# Lichens and associated fungi from Glacier Bay National Park, Alaska

**DOI:** 10.1017/S0024282920000079

**Published:** 2020-03

**Authors:** Toby Spribille, Alan M. Fryday, Sergio Pérez-Ortega, Måns Svensson, Tor Tønsberg, Stefan Ekman, Håkon Holien, Philipp Resl, Kevin Schneider, Edith Stabentheiner, Holger Thüs, Jan Vondrák, Lewis Sharman

**Affiliations:** 1 Department of Biological Sciences, CW405, University of Alberta, Edmonton, Alberta T6G 2R3, Canada; 2 Department of Plant Sciences, Institute of Biology, University of Graz, NAWI Graz, Holteigasse 6, 8010 Graz, Austria; 3 Division of Biological Sciences, University of Montana, 32 Campus Drive, Missoula, Montana 59812, USA; 4 Herbarium, Department of Plant Biology, Michigan State University, East Lansing, Michigan 48824, USA; 5 Real Jardín Botánico (CSIC), Departamento de Micología, Calle Claudio Moyano 1, E-28014 Madrid, Spain; 6 Museum of Evolution, Uppsala University, Norbyvägen 16, SE-75236 Uppsala, Sweden; 7 Department of Natural History, University Museum of Bergen Allégt. 41, P.O. Box 7800, N-5020 Bergen, Norway; 8 Faculty of Bioscience and Aquaculture, Nord University, Box 2501, NO-7729 Steinkjer, Norway; 9 NTNU University Museum, Norwegian University of Science and Technology, NO-7491 Trondheim, Norway; 10 Faculty of Biology, Department I, Systematic Botany and Mycology, University of Munich (LMU), Menzinger Straße 67, 80638 München, Germany; 11 Institute of Biodiversity, Animal Health and Comparative Medicine, College of Medical, Veterinary and Life Sciences, University of Glasgow, Glasgow G12 8QQ, UK; 12 Botany Department, State Museum of Natural History Stuttgart, Rosenstein 1, 70191 Stuttgart, Germany; 13 Natural History Museum, Cromwell Road, London SW7 5BD, UK; 14 Institute of Botany of the Czech Academy of Sciences, Zámek 1, 252 43 Průhonice, Czech Republic; 15 Department of Botany, Faculty of Science, University of South Bohemia, Branišovská 1760, CZ-370 05 České Budějovice, Czech Republic; 16 Glacier Bay National Park & Preserve, P.O. Box 140, Gustavus, Alaska 99826, USA

**Keywords:** biodiversity, evolution, floristics, key, latitudinal diversity gradient, molecular, new species, phylogenetics, symbiosis, taxonomy, temperate rainforest

## Abstract

Lichens are widely acknowledged to be a key component of high latitude ecosystems. However, the time investment needed for full inventories and the lack of taxonomic identification resources for crustose lichen and lichenicolous fungal diversity have hampered efforts to fully gauge the depth of species richness in these ecosystems. Using a combination of classical field inventory and extensive deployment of chemical and molecular analysis, we assessed the diversity of lichens and associated fungi in Glacier Bay National Park, Alaska (USA), a mixed landscape of coastal boreal rainforest and early successional low elevation habitats deglaciated after the Little Ice Age. We collected nearly 5000 specimens and found a total of 947 taxa, including 831 taxa of lichen-forming and 96 taxa of lichenicolous fungi together with 20 taxa of saprotrophic fungi typically included in lichen studies. A total of 98 species (10.3% of those detected) could not be assigned to known species and of those, two genera and 27 species are described here as new to science: *Atrophysma cyanomelanos* gen. et sp. nov., *Bacidina circumpulla*, *Biatora marmorea*, *Carneothele sphagnicola* gen. et sp. nov., *Cirrenalia lichenicola*, *Corticifraga nephromatis*, *Fuscidea muskeg*, *Fuscopannaria dillmaniae*, *Halecania athallina*, *Hydropunctaria alaskana*, *Lambiella aliphatica*, *Lecania hydrophobica*, *Lecanora viridipruinosa*, *Lecidea griseomarginata*, *L. streveleri*, *Miriquidica gyrizans*, *Niesslia peltigerae*, *Ochrolechia cooperi*, *Placynthium glaciale*, *Porpidia seakensis*, *Rhizocarpon haidense*, *Sagiolechia phaeospora*, *Sclerococcum fissurinae*, *Spilonema maritimum*, *Thelocarpon immersum*, *Toensbergia blastidiata* and *Xenonectriella nephromatis*. An additional 71 ‘known unknown’ species are cursorily described. Four new combinations are made: *Lepra subvelata* (G. K. Merr.) T. Sprib., *Ochrolechia minuta* (Degel.) T. Sprib., *Steineropsis laceratula* (Hue) T. Sprib. & Ekman and *Toensbergia geminipara* (Th. Fr.) T. Sprib. & Resl. Thirty-eight taxa are new to North America and 93 additional taxa new to Alaska. We use four to eight DNA loci to validate the placement of ten of the new species in the orders *Baeomycetales*, *Ostropales*, *Lecanorales*, *Peltigerales*, *Pertusariales* and the broader class Lecanoromycetes with maximum likelihood analyses. We present a total of 280 new fungal DNA sequences. The lichen inventory from Glacier Bay National Park represents the second largest number of lichens and associated fungi documented from an area of comparable size and the largest to date in North America. Coming from almost 60°N, these results again underline the potential for high lichen diversity in high latitude ecosystems.

**Table of Contents**

Introduction
The present study
Materials and Methods
Study area
Climate
Glaciation and vegetation history
Stratification of study area into target sampling units
Specimen analysis
Molecular data
Phylogenetic trees
Species delimitation and nomenclature
Presentation of species data
Comparison between sectors and national parks
Results and Discussion
Comparison of sectors within GLBA
Lichen diversity in the national parks of the greater Gulf of Alaska region
Phylogenetic trees
Descriptions of New Genera and Species
*Atrophysma* T. Sprib.
*Atrophysma cyanomelanos* T. Sprib.
*Bacidina circumpulla* S. Ekman
*Biatora marmorea* T. Sprib.
*Carneothele* Fryday, T. Sprib. & M. Svenss.
*Carneothele sphagnicola* Fryday, M. Svenss. & Holien
*Cirrenalia lichenicola* Pérez-Ort.
*Corticifraga nephromatis* Pérez-Ort.
*Fuscidea muskeg* Tønsberg & M. Zahradn.
*Fuscopannaria dillmaniae* T. Sprib.
*Halecania athallina* Fryday
*Hydropunctaria alaskana* Thüs & Pérez-Ort.
*Lambiella aliphatica* T. Sprib. & Resl
*Lecania hydrophobica* T. Sprib. & Fryday
*Lecanora viridipruinosa* M. Svenss. & T. Sprib.
*Lecidea griseomarginata* Fryday
*Lecidea streveleri* T. Sprib.
*Miriquidica gyrizans* Fryday
*Niesslia peltigerae* Pérez-Ort.
*Ochrolechia cooperi* T. Sprib.
*Placynthium glaciale* Fryday & T. Sprib.
*Porpidia seakensis* Fryday
*Rhizocarpon haidense* Brodo & Fryday
*Sagiolechia phaeospora* Fryday & T. Sprib.
*Sclerococcum fissurinae* Pérez-Ort.
*Spilonema maritimum* T. Sprib. & Fryday
*Thelocarpon immersum* Fryday
*Toensbergia blastidiata* T. Sprib. & Tønsberg
*Xenonectriella nephromatis* Pérez-Ort.
Other Species Treated in Detail
*Absconditella rosea* Kalb & Aptroot
*Lecanora alaskensis* H. Magn.
*Lecanora leptacina* Sommerf.
*Lepra subvelata* (G. K. Merr.) T. Sprib. and similar taxa
*Ochrolechia xanthostoma* (Sommerf.) K. Schmitz & Lumbsch and similar taxa
*Steineropsis alaskana* T. Sprib. & Muggia
*Steineropsis laceratula* (Hue) T. Sprib. & S. Ekman
Known Unknowns
Catalogue of All Named Taxa Found
Acknowledgements
References

## Introduction

The landscapes of south-east Alaska are best known for their most striking macrofeatures: snow-capped mountains, misty saltwater fjords and dark coniferous rainforests. Closer examination reveals that the texture of nearly every terrestrial feature in south-east Alaska is, in one way or another, determined at a much smaller scale. Zooming from the landscape view into the canopies of the coastal rainforests and the tapestry of their outcrops and boulder fields reveals a Russian doll of nested ecosystems, one within another, within another. At the scale of an ecosystem a human can hold in her hand, it is fungi and bryophytes that form the building blocks of the multicellular canopy, supporting yet another set of nested dolls of microbial and invertebrate life in their peaks and ravines. It is at this scale, where fungi, algae and bacterial biofilms meet in a permanently wet, cold milieu, that the south-east Alaskan temperate rainforest exhibits peak biodiversity.

Lichens, *s’éixwani* to the Tlingit (Edwards 2009), the indigenous people of south-east Alaska, played a role in traditional food and garment dyeing for the residents of these fjords for thousands of years. In Glacier Bay, the subject of the present paper, lichens are featured in place names and play an outsized role in the recent vegetation history. When the first European collections of lichens were made here, in the framework of the Harriman Expedition (Cummings 1904), Glacier Bay had only recently undergone a massive glacial retreat of over 80 km as a result of saltwater glacial erosion. Only a few years later, the American ecologist William Skinner Cooper arrived in Glacier Bay and began a series of studies that shaped the textbook description of plant succession (Cooper 1923), now the longest-running primary succession plot series in the world (Buma *et al.* 2017). Despite its fame in plant ecology, Glacier Bay was neglected by lichen researchers in the 20th century. Far fewer collectors have worked here compared to other localities in Alaska, for example, the Juneau region (Krog 1968), Sitka or the north end of the Lynn Canal (see e.g. Spribille *et al.* 2010). Between the 1899 Harriman Expedition and the beginning of the present study, we could reconstruct the activity of 17 different collectors or groups of collectors, based on specimens in US, Canadian and Swedish herbaria (Supplementary Material Table S1, available online). Most collected specimens of common macrolichens, with a few notable exceptions. By the end of the 20th century, the recently deglaciated tundra-like pavements visited during the Harriman Expedition had grown into mature forest (Buma *et al.* 2017).

Since the 1990s, attention has been increasingly focused on south-east Alaska as a biodiversity hotspot in conjunction with controversy over commercial logging in the Tongass National Forest (Durbin 1999). In parallel, ecologists have begun to draw attention to the forests of south-east Alaska as a global archetype of ‘temperate rainforest’ (DellaSala *et al.* 2011), highlighted to a significant extent by characteristic lichen assemblages (Goward & Spribille 2005). While some research was conducted on south-east Alaska's lichens in the 1960s (McCullough 1965; Krog 1968), lichens gained significance here from the 1990s onwards, with their use in air quality monitoring (Geiser *et al*. 1994; Derr *et al.* 2007; Derr 2010), the characterization of ecological indicator species (Dillman 2004; Root *et al*. 2014), the drafting of a first lichen list for all of south-east Alaska (Geiser *et al*. 1998) and the first steps to manage National Forest lands for rare and ‘sensitive’ lichens. Considerable work has been carried out in coastal temperate rainforest areas to the south, especially by I. M. Brodo on Haida Gwaii (e.g. Brodo 1995, 2010; Brodo & Ahti 1996; Brodo & Santesson 1997; Brodo & Wirth 1998). Systematic and phylogeographic studies have suggested that outer coastal rainforests bordering the north-eastern Pacific Ocean may have provided Pleistocene refugia to epiphytic lichens (Printzen *et al.* 2003) and, for some taxa, a hotbed of speciation (Brodo 1995; Jørgensen 2005).

Cruise ship tourism has gradually increased since its onset in the late 1960s and concerns about air quality have led to the introduction of lichen-based biomonitoring in Glacier Bay and elsewhere in south-east Alaska. In recent years, *c*. 400 000 people have visited Glacier Bay annually on cruise ships, constituting over 95% of all visitors (Nemeth & Apgar 2010). A cruise ship may spend 9–12 hours in Glacier Bay, with delays in the lower bay to pick up Park rangers and berthing time in front of glaciers in the upper West Arm. Output of pollutants in Glacier Bay has been estimated at 780 mol km^−2^ h^−1^ for SO_2_ in a single season under reported cruise speeds (Mölders *et al*. 2013). Air quality monitoring plots based on lichen community and collection protocols were established as a baseline for the first time in 2008 at Bartlett Cove (at Park Headquarters near Gustavus) and Blue Mouse Cove in the West Arm of Glacier Bay. Monitoring included throughfall deposition analysis and direct measurement of heavy metal concentrations in lichen thalli using inductively coupled plasma mass spectrometry (ICP-MS; Schirokauer *et al*. 2014). Air quality monitoring relies on two approaches in this ongoing long-term study: 1) the propensity of lichens to accumulate heavy metals that can then be quantified using an ICP-MS element analysis protocol; 2) the indicator value of species assemblages rated for sensitivity to nitrogen enrichment and SO_2_. Results to date record an elevated amount of lithium at the Blue Mouse Cove site and elevated N values (*c*. 90% above regional reference thresholds), both attributed to natural factors such as geology and proximity to seawater (Schirokauer *et al*. 2014). However, lichen compositional data were well within the range of reference sites on the adjacent Tongass National Forest (Schirokauer *et al*. 2014).

Several factors make compositional analysis of lichens for air quality monitoring relatively difficult with the knowledge we have to date. First, our baseline knowledge of the lichens has been, until now, rudimentary. As much as 10% of the lichen species in south-east Alaska have yet to be given scientific names (Spribille *et al*. 2010; present study). Second, achieving meaningful levels of biological species monitoring requires factoring in the successional dynamics and high geological and climatic heterogeneity of Glacier Bay itself. Species composition shifts may be as likely to be related to these natural abiotic factors as they are to external stressors such as increased pollutant deposition. Partitioning the signal for natural and anthropogenic factors benefits from increased resolution in lichen taxonomy.

### The present study

The documentation of over 750 lichens and associated fungi in the nearby Klondike Gold Rush National Historical Park (KLGO; Spribille *et al*. 2010) suggested that lichen richness in SE Alaska was even greater than previously estimated. It raised several questions relevant to understanding both regional species richness patterns and the behaviour of meta-regional lichen species assemblages: 1) is such richness generally to be expected in coastal Alaska, or was KLGO exceptionally rich? 2) How specific is regional species composition (i.e. how much ‘turnover’ in species would there be from one fjord to another)? 3) On a gradient from inland to outer coast (increasing oceanicity), how does lichen richness change? These questions were at the core of a proposal funded in 2011 by the US National Park Service to replicate the KLGO study *c*. 80 km to the SSW in Glacier Bay National Park (hereafter referred to by its US National Park Service acronym, GLBA, and not equivalent to ‘Glacier Bay’, which refers to the bay itself). We hypothesized that the high species numbers we detected in KLGO were not unique, but that the infrequency with which such results are reported was rather a reflection of the large investment in effort required to name species in a poorly studied region. We also hypothesized that GLBA would have more species owing to its larger size and greater geological diversity but would largely overlap with the KLGO species pool. Answering questions 1 and 2 above would be possible with an inventory that replicated the style and intensity of the KLGO study; answering question 3 might be more difficult, as many factors covary with climate while, independently, richness can be influenced by geological parent material. We expected this to be the case in GLBA as it is geologically complex, straddling no fewer than three tectonostratigraphic terranes (Perry *et al.* 2009).

Sixty-nine species of lichens had been recorded for GLBA at the time we began our study in 2011 (Bennett & Wetmore 2005). We had two objectives: 1) to acquire a baseline inventory of species in GLBA to support future ecological and monitoring studies; 2) to develop a georeferenced species occurrence database on species pool and turnover (a) along a deglaciation gradient and (b) between geographical sectors and nearby areas (such as KLGO). While imperfect, the resulting data set allows us to make inferences about species richness patterns fjord-to-fjord as well as local and regional gradients. Our results are aggregated into two parts: A) a condensed summary of the species inventory results and caveats, and how these inform our understanding of regional species turnover in SE Alaska; B) a full list of the taxa discovered, including 29 taxonomic novelties (two genera, 27 species) and 71 known unknowns, species which we recognize but the taxonomy of which cannot be resolved at this time.

## Materials and Methods

### Study area

Glacier Bay National Park and Preserve (Fig. 1) is one of the largest national parks in the United States, at 10 849 km^2^ including 10 616 km^2^ in the National Park proper and 233 km^2^ in the Preserve, located in the delta of the Alsek River to the north-west of the park and administered by the park. The current study is concerned only with the National Park and within GLBA with terrestrial and intertidal habitats not currently covered by glaciers. The non-glacier terrestrial land base of GLBA, and thus the study area, currently encompasses *c.* 6023 km^2^. Almost the entire study area is inaccessible by road, the exceptions being the park headquarters area at Bartlett Cove and an access road to the city water supply intake for the town of Gustavus, on Falls Creek. Except for sampling sites in the Bartlett Cove, Tower Road, Gustavus, Falls Creek and Excursion Ridge areas, all sites surveyed were accessed by boat. Landing accessibility, weather and boat scheduling were major factors in planning our sampling.

**Fig. 1.**

A, Alaska and the north-east Pacific showing US national parks in which major lichen inventories have been conducted in the last ten years (outlined); B, Glacier Bay National Park, showing sample sites (black circles) and subdivisions into sectors referred to in the text (separated by black lines). Geographical sectors are indicated as follows (see text for more details): DUN = Dundas, EA = East Arm, EX = Excursion Ridge, GB = Glacier Bay, GUS = Gustavus, WA = West Arm.

### Climate

**Fig. 2.**
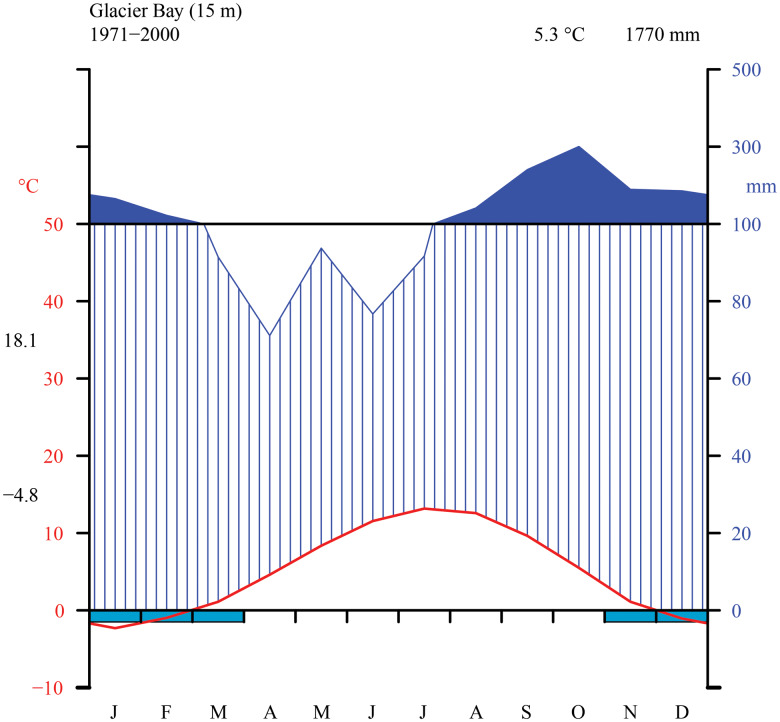
Thirty-year monthly normals of precipitation and temperature near sea level from the station at Glacier Bay (NOAA 2000). Walter-Lieth diagram indicating temperature (°C) on left y-axis and precipitation (mm) on right y-axis (with daily maximum average temperature of the warmest month and daily minimum average temperature of the coldest month in black along left margin), as well as mean annual temperature and precipitation (top right, black).

The Glacier Bay area is dominated by a wet, maritime climate with moderate temperature fluctuations and low overall annual temperature. We generated a Walter-Lieth climate diagram (Fig. 2) with data from NOAA (2000) using the R package climatol v3.1.2 (https://cran.r-project.org/web/packages/climatol/index.html). The mean monthly temperature at Bartlett Cove is 5.3 °C, which is similar to Skagway (5.1 °C) near KLGO, with freezing temperatures common from November to March. The outer, coastal parts of GLBA however are much warmer, with Cape Spencer registering only 70 freezing days per winter over a six-year period (Loewe 1966). The annual precipitation at Bartlett Cove is 1770 mm, nearly three times that of Skagway (666 mm; NOAA 2000) but still considerably less than on Haida Gwaii, British Columbia (2140–2523 mm; Brodo 1995). Variation in precipitation within GLBA is likely to be large. Outside of the long-term sampling at Bartlett Cove, data for Cape Spencer, on the outer coast and near one of our sampling sites in the present study, indicate annual precipitation of 2860 mm, and at Yakutat, which is on the coast 150 km to the north, 3330 mm (Loewe 1966). Values over 2000 mm are probably widespread in Glacier Bay, especially in mountain areas and to the west of the Fairweather Mountains. Preliminary data support the impression that the West Arm might lie in a rain shadow, receive less rain and snow than the East Arm or the main part of Glacier Bay, and be *c.* 1 °C colder than the rest of Glacier Bay (Kopczynski *et al*. 2003; Finnegan *et al*. 2007). Short-term data from climate measurements over several summers at Casement Glacier in the East Arm indicate values similar to those at Gustavus (Loewe 1966).

### Glaciation and vegetation history

The history of deglaciation and post-glacial primary succession in Glacier Bay are well documented in a series of detailed studies beginning with the classical work of Cooper (1923). Though much of the area of Glacier Bay was covered in ice during the Pleistocene, the latest glaciation peaked in the Little Ice Age (*c.* 1300 to 1870 C.E.) and rapidly receded in the early part of the 19th century. By the late 19th century, ice had retreated to near the mouth of the East Arm and the area now known as Muir Point. Glacial retreat proceeded with greater speed in the West Arm than in the East Arm and many studies on succession, including those on vegetation (e.g. Chapin *et al*. 1994) and stream invertebrate community development (e.g. Milner *et al*. 2000), give special attention to the spectacular chronosequence offered in the East Arm. Boggs *et al*. (2008, 2010) provide fine-scale baseline descriptions of current land cover classes and plant associations for the entire park and preserve complex. Cooper (1923) mentioned the presence of abundant *Stereocaulon alpinum* in early successional stages but otherwise lichens have not been treated at the species level in the cited studies.

### Stratification of study area into target sampling units

Following a reconnaissance in September 2011, the 2012 sampling season was laid out to obtain reference species lists for six main geographical sectors (Fig. 1) overlaid with specific abiotic criteria. The geographical targets were A) four main areas glaciated in the Little Ice Age: West Arm Glacier Bay (WA), East Arm Glacier Bay to Muir Point (EA), the main part of Glacier Bay including Geikie Inlet (GB), and the glaciated Gustavus area from Bartlett Cove to the base of Excursion Ridge (GUS); B) two areas not glaciated since the end of the Pleistocene: Excursion Ridge and unglaciated Falls Creek down to the Bear Track Inn (EX) and the Dundas to Taylor Bay area parallel to Icy Straits (DUN). Further potential sampling sectors, such as the outer coast, Deception Hills and the Alsek River outwash plain, were not sampled due to logistical constraints.

The study area harbours large habitat diversity (examples in Fig. 3). For the purposes of lichen sampling, this habitat diversity could be classified in terms of vertical zones (near sea level, mountain slopes to 600 m, subalpine/alpine) and geological parent material (acidic rocks including granite, intermediate pH rocks including argillites, high pH rocks including limestones, and ultramafic rocks including gabbro). If only these seven coarse categories were applied, without reference to topographic aspect and plant community succession, we would have 42 geographical sector/habitat envelopes to survey. Because surveying with this level of stratification was logistically prohibitive, we opted to focus on as many different habitats in as many sectors as was feasible within the allocated sampling period, and given boat time, safety and access constraints. The resulting sampling was biased towards low elevations for all sectors, except EX and DUN, and gave mixed results for major bedrock types. We did not explicitly sample each sector based on surface age since deglaciation, though this is also critical to species composition and was used locally as a sampling criterion in sectors WA and EA. Other factors were considered on a site-by-site basis, such as making an inventory of possible phorophyte substrata (bark of available tree and shrub species). Historical specimens from Glacier Bay in herbaria were not systematically surveyed as the majority of these were of common species and had imprecise locality information; only noteworthy records were checked.

**Fig. 3.**

GLBA landscapes. A, terminus of Riggs Glacier (East Arm) in 2014; B, recently deglaciated *Dryas* mats with numerous *Stereocaulon* species just east of the terminus of Muir Glacier (East Arm) in 2014; C, alder thicket along a jeep trail at Tower Road near the park entrance (Gustavus sector; M. Svensson); D, *Pinus contorta* muskeg in the Falls Creek area, not glaciated during the Little Ice Age (included in the Excursion Ridge sector); E, *Picea sitchensis* rainforest near Bartlett Cove (Gustavus sector); F, alpine meadows and heaths on Excursion Ridge, the richest locality studied for lichens and associated fungi.

Sampling followed an ‘observational feedback’ approach (Spribille *et al*. 2010) and was delimited by neither fixed sampling times nor plots; maximization of species capture within the time we could spend at a site was the sole field objective. GPS waypoint data (Supplementary Material Table S2, available online) were gathered using WGS84 Datum in digital degrees. A total of 349 waypoints were recorded on multiple GPS devices carried by individual researchers. Following deduplication and imposing a 200 × 200 m grid, this translates to 103 unique sites surveyed.

### Specimen analysis

Specimens were examined in the laboratory under a dissecting microscope and pre-sorted for light microscopy or chemical analysis. Specimens were examined with dissecting and compound microscopes with a polarizing light filter and Nomarksi differential interference contrast. The presence or absence of birefringent crystals is noted as POL+/POL−, respectively. Thallus and ascomatal sections were prepared in water and treated with 10% potassium hydroxide (KOH), household bleach (NaOCl, shortened to C according to lichenological convention), *para-*phenylenediamine (C₆H₄(NH₂)₂, abbreviated to PD), nitric acid (HNO_3_; 1% unless otherwise indicated), 1% hydrochloric acid (HCl), Lugol's solution (reported by its full name when referring to the solution, or abbreviated to I when reported as a spot test) or lactophenol cotton blue (LCB; Merck). Pigments are described according to Meyer & Printzen (2000). Images of specimens analyzed by TS and AMF were captured with an Olympus XC50 camera mounted on an Olympus SZX16 dissecting microscope; microphotograph images were captured on a Zeiss Axioskop light microscope. In several cases, images were stacked using CombineZM freeware (https://combinezm.en.lo4d.com/windows). Specimens were mounted in water for photographing unless otherwise specified. Scanning electron microscopy was carried out using an FEI XL-30 scanning electron microscope on gold sputtercoated, dry thalli affixed to aluminium stubs. Ascospore measurements are provided for new taxa as (smallest absolute measurement–) smallest mean – largest mean (–largest absolute measurement) or minimum value – arithmetic mean value ± standard deviation – maximum value; *s* in this case denotes sample standard deviation, *n* denotes sample size; in *Hydropunctaria alaskana* the measurements are (minimum–) [median − 1 *s*] – [median + 1 *s*] (–maximum). Figures in the main species catalogue reflect informal measurements of several ascospores.

Secondary metabolite analysis was carried out using thin-layer chromatography (TLC) techniques for lichens described by Culberson (1972), Culberson *et al*. (1981) and Culberson & Johnson (1982). All analyses employed glass plates (Macherey-Nagel 821 030) to visualize fatty acids. Fatty acids were identified by vertically dipping the fully developed and dried plates into a tank of tap water (in Bergen after application with a fine H_2_O mister) and noting hydrophobic spots in the first 5–10 s while dripping off. Common substances are reported in the text by their acid names and several are abbreviated as follows: atranorin (atr), fumarprotocetraric acid (fpc), protocetraric acid (pc). The presence of satellite substances is denoted with the abbreviation ‘sats’.

Unless otherwise stated, voucher specimens collected for this project are deposited in the herbarium of Michigan State University (MSC). Due to the changing application of National Park Service rules on the deposition of specimens, vouchers that were previously cited as being deposited in other herbaria, especially GZU, by Spribille *et al.* (2014*a*, *b*) and Resl *et al.* (2015, cited in their Supplementary Materials) have been transferred to MSC, except for vouchers that were collected outside the formal park boundaries.

### Molecular data

Molecular (DNA) analysis was carried out on selected specimens using a standardized laboratory pipeline. Ascomata or thallus fragments were pulverized in 1.5 ml Eppendorf tubes using a Retsch cell grinder with a single 3 mm steel bead after freezing at −80 °C. We extracted genomic DNA using the Qiagen DNeasy Plant Mini Kit following the manufacturer's instructions. In the case of sparse material, we used the QIAmp DNA Investigator Kit. We eluted raw nucleic acids in 50–75 μl of elution buffer without RNAse and used the samples undiluted for subsequent PCR reactions. For most samples, we sequenced the internal transcribed spacer (ITS rDNA; internal transcribed spacer regions 1 and 2 as well as the embedded 5.8S region of the ribosomal rDNA) as it is the single most sequenced locus in fungi and widely used as a barcode (Schoch *et al*. 2012). Primers and annealing temperatures follow those outlined in Resl *et al*. (2015). PCR was performed using PuReTaq Ready-To-Go PCR beads (GE Healthcare, Chicago) or the KAPA 3G Plant PCR Kit (KAPA Biosystems). PCR products were sequenced by Microsynth (Switzerland). Newly acquired sequences are listed in Table 1 and for all DNA isolates from which no new sequences are published, in Supplementary Material Table S3 (available online).

**Table 1.** Voucher information and NCBI GenBank Accession numbers for all specimens from which DNA sequences are newly generated for this study. Voucher information and sequence accession numbers for specimens from which no newly generated data are provided here can be found in Supplementary Material Table S3 (available online). A dash (–) indicates no data, an asterisk (*) indicates that the voucher does not appear in any tree in the present paper. GenBank Accessions beginning with letters other than ‘MN’ or ‘MT’ represent sequences generated in other studies.

Used in	Isolates	Species	Voucher	Origin	Publication	ITS	18S	28S	mtSSU	*Mcm7*	*RPB1*	*RPB2*	*EF1a*
*	T1345	*Amygdalaria* sp. AMF10121	*Spribille* 38890 (MSC)	USA: Alaska, Glacier Bay National Park, Excursion Ridge	here	MN483069	–	–	–	–	–	–	–
Fig. 5; Fig. 7	P172	*Arthrorhaphis citrinella*	*Hafellner* 74354 (GZU)	Austria	here	–	MN508042	MN460242	MN508312	MN437631	MN437641	–	MN437649
*	NA	*Atla recondita*	*Fryday* 10302 (MSC)	USA: Alaska, Glacier Bay National Park, Falls Creek, Upper Falls	here	MN483098	–	–	–	–	–	–	–
Fig. 9	T1346	*Atrophysma cyanomelanos*	*Spribille* 39402 (MSC)	USA: Alaska, Hoonah-Angoon District, Glacier Bay National Park, Excursion Ridge	here	MN483104	–	MN460209	MN508262	MN437613	–	–	–
Fig. 9	T1807	*A. cyanomelanos* (holotype)	*Spribille* 39425 (MSC)	USA: Alaska, Hoonah-Angoon District, Glacier Bay National Park, Excursion Ridge	here	MN483105	–	MN460210	MN508263	–	–	–	–
Fig. 10	T621	*Bacidia laurocerasi* subsp. *laurocerasi*	*Spribille* 26334 (KLGO)	USA: Alaska, Klondike Gold Rush National Historical Park, Chilkoot Trail	here	MN483106	–	MN460211	MN508264	–	–	–	–
Fig. 10	T1348	*Biatora marmorea*	*Spribille* 38009 (MSC)	USA: Alaska, Glacier Bay National Park, Marble Mountain	here	MN483107	–	–	MN508265	–	–	–	–
Fig. 5	T1396	*Boreoplaca ultrafrigida*	*Spribille* 31796 (GZU)	Russia: Khabarovskiy Krai, Bureinskiy Zapovednik, upper reach of the Pravaya Bureya River, Tsarskaya Dorogа	here	MN483108	–	MN460212	MN508266	MN437614	–	–	–
*	JV_159	*Caloplaca caesiorufella*	*Spribille* 39314 (MSC)	USA: Alaska, Glacier Bay National Park, Muir Point	here	MN483089	–	–	–	–	–	–	–
*	JV_160	*C. caesiorufella*	*Spribille* 39315 (MSC)	USA: Alaska, Glacier Bay National Park, Muir Point	here	MN483088	–	–	–	–	–	–	–
*	T1244	*C. kamczatica*	*Spribille* 38195 (MSC)	USA: Alaska, Glacier Bay National Park, Fern Harbor	here	MN483091	–	–	–	–	–	–	–
*	T1229	*C. sinapisperma*	*Spribille* 36443 (MSC)	USA: Alaska, Glacier Bay National Park, Oystercatcher Cove	here	MN483095	–	–	–	–	–	–	–
*	T1238	*C. sinapisperma*	*Spribille* 38480 (MSC)	USA: Alaska, Glacier Bay National Park, West Arm, Gloomy Knob	here	MN483090	–	–	–	–	–	–	–
Fig. 5	T1801	*Candelaria concolor*	*Obermayer* 12655 (GZU)	Austria: Steiermark, Oststeirisches Riedelland, 9 km NE of the centre of Graz, Schaftal, Hollergraben	here	MN483109	–	–	MN508267	–	–	–	–
*	T1110	*Carneothele sphagnicola*, (see text for explanation)	*Spribille* 38738 (NY)	USA: Alaska, Glacier Bay National Park, Yellowlegs Muskeg	here	MN483087	–	MN460247	–	–	–	–	–
*	P90	*Cecidonia xenophana*	*Spribille* 38782 (MSC)	USA: Alaska, Glacier Bay National Park, Excursion Ridge	here	–	–	MN460251	–	–	–	–	–
*	T1137	*Chaenotheca* sp. S38739	*Spribille* 38739 (MSC)	USA: Alaska, Glacier Bay National Park, Yellowlegs Muskeg	here	–	–	–	MN508260	–	–	–	–
Fig. 9	L873	*Coccocarpia palmicola*	*Wheeler & Nelson* 103 (CONC)	Chile: Region X, Senda Darwin Biological Research Station	here; Spribille & Muggia 2013	MN483110	–	JX464116	–	–	–	–	–
*	T1284	*Dendriscosticta wrightii*	*Spribille* 36122 (MSC)	USA: Alaska, Glacier Bay National Park, Bartlett Cove	here	MN483092	–	–	MN508337	–	–	–	–
*	T1285	*D. wrightii*	*Spribille* 39269 (MSC)	USA: Alaska, Glacier Bay National Park, Muir Point	here	MN483093	–	–	MN508338	–	–	–	–
Fig. 9	P271	*Fuscopannaria* aff. *sorediata*	*Himelbrant* K04-9-100 (H)	Russia: Kamchatka	here	MN483111	–	–	MN508268	–	–	–	–
Fig. 9	T1214	*F. dillmaniae* (holotype)	*Spribille* 38036 (UPS)	USA: Alaska, Gustavus area, Tower Road	here; Schneider *et al.* 2015	MN483112	–	KP794959	MN508269	–	–	–	–
Fig. 7	P144	*Gyalidea* aff. *lecideopsis* var. *eucarpa*	*Spribille* 39048 (MSC)	USA: Alaska, near Gustavus, Falls Creek near hydro plant	here	MN483071	–	–	–	MN437615	MN437636	MN437643	MN437644
Fig. 21	NA	*Hydropunctaria alaskana*	*Orange* 22769	Canada: British Columbia, Vancouver Island, west of Sooke, Flea Beach	here	MN483172	–	–	–	–	–	–	–
Fig. 21	NA	*H. alaskana*	*Orange* 22768	Canada: British Columbia, Vancouver Island, west of Sooke, Flea Beach	here	MN483171	–	–	–	–	–	–	–
Fig. 21	NA	*H. alaskana*	*Fryday* 10458 (MSC—holotype)	USA: Alaska, Glacier Bay National Park, Taylor Bay	here	MN483166	–	–	–	–	–	–	–
Fig. 21	NA	*H. alaskana*	*Fryday* 10456 (MSC—topotype)	USA: Alaska, Glacier Bay National Park, Taylor Bay	here	MN483165	–	–	–	–	–	–	–
Fig. 21	2880	*H. alaskana*	*Pérez-Ortega* 2045 (MA-Lich)	USA: Alaska, Petersburg, South Mitkof Island, Sumner Strait	here	MN483169	–	–	MN508286	–	–	–	–
Fig. 21	2883	*H. alaskana*	*Pérez-Ortega* 2042 (MA-Lich)	USA: Alaska, Petersburg, South Mitkof Island, Sumner Strait	here	MN483170	–	–	–	–	–	–	–
Fig. 21	NA	*H. rheitrophila*	*Thues* W1288 (BM)	Germany: Baden-Württemberg, Odenwald, Reisenbacher Grund, in the stream Reisenbach *c*. 500m upstream of village	here	MN483167	–	JN573785	EF105159	–	–	–	–
Fig. 21	NA	*H. scabra*	*Thues* W0409 (FR)	Germany: Baden-Württemberg, Black Forest, in the stream St.Wilhelmer Talbach	here	MN483168	–	–	–	–	–	–	–
*	T1315	*Hypogymnia* sp. S38816	*Spribille* 38816 (MSC)	USA: Alaska, Glacier Bay National Park, Excursion Ridge	here	MN483070	–	–	–	–	–	–	–
Fig. 10	T532	*Japewia tornoensis*	*Spribille* 28417 (GZU)	Canada: Yukon, LaBiche River area	here	MN483113	–	–	MN508270	–	–	–	–
Fig. 8	P85	*Lambiella aliphatica*	*Spribille* 38395-B (MSC)	USA: Alaska, Glacier Bay National Park, Excursion Ridge	here	MN483114	–	–	–	–	–	–	–
Fig. 8	P190	*L. hepaticola*	*Pérez-Ortega* nr. 2001 (MA-Lich)	Chile: XII. Region, Tierra del Fuego, Bahia Blanca	here	MN483115	–	–	–	–	–	–	–
Fig. 5	T1721	*Lasallia pustulata*	*Hansen*, Lichenes Danici 778 (GZU)	Denmark: Bornholm, Gudhjem, Jernkås	here	MN483116	–	–	MN508271	–	–	–	–
Fig. 10	T1349	*Lecania hydrophobica*	*Spribille* 39680 (MSC, type material)	USA: Alaska, Glacier Bay National Park, Taylor Bay	here	–	–	–	MN508313	–	–	–	–
*	T1182	*Lecanora alaskensis*	*Tønsberg* 41794 (MSC)	USA: Alaska, Glacier Bay National Park, Fern Harbor	here	–	–	–	MN508326	–	–	–	–
Fig. 10	T1219	*L. leptacina*	*Spribille* 38985 (MSC)	USA: Alaska, Glacier Bay National Park, Dundas Bay	here	MN483118	–	–	MN508273	–	–	–	–
Fig. 10	T1019	*Lecanora* sp.	*Spribille* 28364 (GZU)	Canada: Yukon, Mt. Martin	here	MN483120	–	–	MN508275	–	–	–	–
Fig. 10	T1181	*Lecanora* sp. AMF10122	*Spribille* 38425 (MSC)	USA: Alaska, Glacier Bay National Park, Excursion Ridge	here	MN483121	–	MN460214	MN508276	–	–	–	–
Fig. 10	T1333	*Lecanora* sp. S38412	*Spribille* 38412 (MSC)	USA: Alaska, Glacier Bay National Park, Excursion Ridge	here	MN483117	–	–	MN508272	–	–	–	–
Fig. 10	T1806	*L. viridipruinosa*	*Fryday* 10130 (MSC, type material)	USA: Alaska, Glacier Bay National Park, Excursion Ridge	here	–	–	–	MN508314	–	–	–	–
Fig. 10	MS008	*Lecidea albofuscescens*	*Svensson* 2760 (MSC)	USA: Alaska, Glacier Bay National Park	here	–	–	–	MN508315	–	–	–	–
Fig. 10	T1789	*L. albofuscescens*	*Spribille* 36527 (MSC)	USA: Alaska, Glacier Bay National Park, Seebree Island	here	–	–	–	MN508316	–	–	–	–
Fig. 10	T1790	*L. albofuscescens*	*Tønsberg* 41791 (MSC)	USA: Alaska, Glacier Bay National Park, Fern Harbor area	here	–	–	–	MN508317	–	–	–	–
*	T1195	*L. griseomarginata*	*Fryday* 9937 (MSC)	USA: Alaska, Glacier Bay National Park, Ptarmigan Creek	here	–	–	MN460248	MN508327	MN437633	–	–	–
Fig. 5	T1287	*L. lactea*	*Spribille* s. n. (2010) (GZU)	USA: Alaska, White Pass	here	MN483122	–	MN460215	MN508277	MN437616	–	–	–
Fig. 10	MS007	*L. malmeana*	*Svensson* 2563 (MSC)	USA: Alaska, Glacier Bay National Park, ‘Moose Meadows’ near park entrance	here	–	–	–	MN508318	–	–	–	–
Fig. 9	L761	*Leciophysma saximontanum*	*Spribille* 21173 (GZU, type material)	Canada: British Columbia, Albert River	here; Spribille & Muggia 2013	MN483147	–	JX464119	JX464135	–	–	–	–
Fig. 9	L742	*Leptogidium dendriscum*	*Spribille & Pettitt* 24172 (CANL)	Canada: British Columbia, Penfold River	here; Muggia *et al*. 2011	MN483123	–	JF938137	JF938196	–	–	–	–
Fig. 9	T1731	*Leptogium saturninum* s. lat.	*Spribille* 39308 (MSC)	USA: Alaska, Glacier Bay National Park, Muir Point	here	MN483124	–	–	MN508278	MN437617	–	–	–
*	T1808	Lichinomycete from *Spilonema maritimum*	*Spribille* 39586 (MSC)	USA: Alaska, Glacier Bay National Park, Taylor Bay	here	–	–	–	MN508328	–	–	–	–
Fig. 5	T1403	*Lobaria pulmonaria*	*Spribille* 39224 (MSC)	USA: Alaska, Glacier Bay National Park, Muir Point	here	MN483125	–	MN460216	MN508281	MN437618	–	–	–
*	T1327	*Lopadium disciforme*	*Spribille* 36687 (MSC)	USA: Alaska, Glacier Bay National Park, near Rush Point	here	–	–	–	MN508329	–	–	–	–
*	T1326	*L. pezizoideum*	*Spribille* 38861 (MSC)	USA: Alaska, Glacier Bay National Park, Excursion Ridge	here	–	–	–	MN508330	–	–	–	MN437653
Fig. 10	T604	*Megalaria columbiana*	*Spribille* 18499 (GZU)	USA: California, Humboldt Co., Trinidad Head	here	–	–	–	MN508319	–	–	–	–
Fig. 10	T603	*M. laureri*	*Mayrhofer* 18417 (GZU)	Montenegro: northern part, N of Bistrica, S of the Tara River canyon	here	–	–	–	MN508320	–	–	–	–
Fig. 10	T1196	*Miriquidica gyrizans*	*Fryday* 10175 (MSC)	USA: Alaska, Glacier Bay National Park, Dundas Bay	here	MN483126	–	MN460217	MN508282	–	–	–	–
Fig. 5; Fig. 10	T852	*M. instrata*	*Spribille* s. n., 2010 (GZU)	USA: Montana, Lincoln Co., Whitefish Range, Lewis Creek talus	here; Spribille *et al*. 2011	JN009720	–	MN460241	MN508311	JN009746	–	–	–
Fig. 10	T1185	*Myriolecis schofieldii*	*Spribille* 39188 (MSC)	USA: Alaska, Glacier Bay National Park, Muir Point	here	MN483119	–	MN460213	MN508274	–	–	–	–
Fig. 5; Fig. 9	T1730	*Nephroma helveticum* subsp. *sipeanum*	*Spribille* 39234 (MSC)	USA: Alaska, Glacier Bay National Park, Muir Point	here	MN483127	–	MN460218	MN508279	MN437619	–	–	–
Fig. 6	T1817	*Ochrolechia* aff. *xanthostoma*	*Tønsberg* 46121 (BG)	Norway: Møre og Romsdal, Herøy, island Remøya	here	MN483173	–	–	MN508284	–	–	–	–
Fig. 6	T1299	*Ochrolechia* sp. S38011	*Spribille* 39304 (MSC)	USA: Alaska, Glacier Bay National Park, Muir Point	here	MN483128	–	MN460219	MN508283	–	–	–	–
Fig. 6	T1085	*Ochrolechia* sp. S38011	*Spribille* 38011 (MSC)	USA: Alaska, Glacier Bay National Park, Marble Mountain	here	–	–	MN460243	MN508321	–	–	–	MN437650
Fig. 6	T1341	*Ochrolechia* sp. S38864	*Spribille* 38864 (MSC)	USA: Alaska, Glacier Bay National Park, Excursion Ridge	here	MN483130	–	–	–	–	–	–	–
*	T1338	*Orbiliaceae* from *Spilonema maritimum*	*Spribille* s. n., 21 Sept. 2010 (GZU)	USA: Alaska, Juneau Borough, west side of Douglas Island at Peterson Creek beach access	here	–	–	MN460246	–	–	–	–	–
*	T1335	*Parmelia saxatilis*	*Spribille* 36599 (MSC)	USA: Alaska, Glacier Bay National Park, East Arm, Wolf Point	here	MN483072	–	–	–	–	–	–	–
*	T1336	*Parmelia* sp. S40729	*Spribille* 38051 (MSC)	USA: Alaska, near Gustavus, Tower Road	here	MN483073	–	–	–	–	–	–	–
Fig. 5; Fig. 9	T1216	*Parmeliella triptophylla*	*Spribille* s. n., 29 Sept. 2012 (GZU)	Canada: British Columbia, Incomappleux Canyon	here	MN483131	–	MN460220	MN508285	MN437620	–	–	–
*	T1212	*P. triptophylla*	*Spribille* 36307 (MSC)	USA: Alaska, Glacier Bay National Park, park entrance	here	MN483096	–	–	–	–	–	–	–
*	T1213	*P. triptophylla*	*Spribille* 37502 (MSC)	USA: Alaska, near Gustavus, Tower Road	here	MN483097	–	–	–	–	–	–	–
Fig. 5; Fig. 9	T1727	*Peltigera collina*	*Spribille* 41076 (GZU)	USA: Montana, Sanders Co., Siegel Creek talus	here	MN483132	–	MN460221	MN508280	MN437621	–	–	–
*	P191	*Pertusaria glaucomela*	*Spribille* 36613 (MSC)	USA: Alaska, Glacier Bay National Park, East Arm, Wolf Point	here	–	MN508044	–	MN508331	–	–	–	–
Fig. 9	T1307	*Placynthium* aff. *asperellum*	*Spribille* 38651 (MSC)	USA: Alaska, Glacier Bay National Park, Queen Inlet	here	MN483135	–	–	–	–	–	–	–
Fig. 9	T1306	*P. asperellum*	*Spribille* 38885 (MSC)	USA: Alaska, Glacier Bay National Park, Excursion Ridge	here	MN483134	–	MN460223	–	–	–	–	–
Fig. 9	T1350	*P. flabellosum*	*Spribille* 38956 (MSC)	USA: Alaska, Glacier Bay National Park, Dundas Bay	here	MN483136	–	–	MN508288	–	–	–	–
Fig. 9	T1220	*P. glaciale*	*Fryday* 9786 (MSC)	USA: Alaska, Glacier Bay National Park, near 2011 terminus of Muir Glacier	here	MN483137	–	MN460224	MN508289	–	–	–	–
Fig. 9	KS88	*P. glaciale*	*Spribille* 40765 (MSC)	USA: Alaska, Glacier Bay National Park, near 2011 terminus of Muir Glacier	here	MT041621	–	MT039417	MT039419	–	–	–	–
Fig. 9	T1305	*Placynthium* sp. S38419	*Spribille* 38419 (MSC)	USA: Alaska, Glacier Bay National Park, Excursion Ridge	here	–	–	MN460245	–	–	–	–	–
Fig. 9	T1310	*Placynthium* sp. S38458	*Spribille* 38458 (MSC)	USA: Alaska, Glacier Bay National Park, West Arm, Gloomy Knob	here	MN483138	–	MN460225	MN508290	–	–	–	–
Fig. 9	T1304	*Placynthium* sp. S38458	*K. Dillman* TNF 581 (TNFS)	USA: Alaska, Tongass National Forest	here	MN483133	–	MN460222	MN508287	–	–	–	–
Fig. 9	T1309	*P. subradiatum*	*Spribille* 38476 (MSC)	USA: Alaska, Glacier Bay National Park, West Arm, Gloomy Knob	here	MN483139	–	MN460226	MN508291	–	–	–	–
Fig. 9	T1308	*P.* aff. *tantaleum*	*Spribille* 36386 (MSC)	USA: Alaska, Glacier Bay National Park, Marble Mountain	here	–	–	MN460244	MN508322	–	–	–	–
Fig. 9	T1183	*P. tantaleum*	*Spribille* 39974 (GZU)	USA: Montana, Flathead Co., Whitefish Range, Trail Creek	here; Schneider *et al.* 2015	MN483140	–	KP794956	MN508292	–	–	–	–
Fig. 5	T1321	*Pleopsidium chlorophanum*	*Spribille* 40380 (GZU)	USA: Montana, Missoula Co., Finlay Lakes trail	here	–	–	KP794962	MN508323	MN437632	–	–	MN437651
*	T1332	*Polycauliona pollinarioides*	*Spribille* s. n., 2012	USA: Alaska, S end of Mitkof Island	here	MN483074	–	–	–	–	–	–	–
*	KS91	*P. pollinarioides*	*Björk* (UBC)	Canada: British Columbia, Albert Head	here	MN483075	–	–	MN508332	–	–	–	–
*	T1317	*P. polycarpa*	*Spribille* 37965 (MSC)	USA: Alaska, Glacier Bay National Park, E side Russell Island	here	MN483076	–	–	–	–	–	–	–
*	KS140	*Polycauliona* sp. S39572	*Fryday* 10661 (MSC)	USA: Alaska, Petersburg Borough, Mitkof Island, Ideal Cove	here	MN483077	–	–	MN508333	–	–	–	–
*	T1301	*Polycauliona* sp. S39572	*Spribille* 39573 (MSC)	USA: Alaska, Glacier Bay National Park, Taylor Bay	here	MN483078	–	–	–	–	–	–	–
*	T1194	*Porpidia seakensis*	*Fryday* 9626 (MSC)	USA: Alaska, Glacier Bay National Park, Bartlett Lake trail	here	–	–	MN460249	–	MN437634	–	–	–
*	T1347	*Psoroma hypnorum* s. lat.	*Spribille* 39296 (MSC)	USA: Alaska, Glacier Bay National Park, Muir Point	here	MN483079	–	–	–	–	–	–	–
Fig. 5	T1800	*Pycnora sorophora*	*Sebernegg* s. n. *& Mayrhofer* 05 May 2011 (GZU)	Austria: Steiermark, Schladminger Tauern, Unterer Zwieflersee, NW Uferbereich	here	MN483141	–	–	MN508293	–	–	–	–
Fig. 5	T990	*Ramalina almquistii*	*Talbot* AML008-X-01A (GZU)	USA: Amlia Island, Aleutians,	here	MT041620	–	MT039416	MT039418	MT041632	–	–	–
Fig. 10	T770	*Ramalina dilacerata*	*Spribille* 0671-B (GZU)	Russia: Khabarovskiy Krai	here; Schneider *et al*. 2015	MN483142	–	KP794953	MN508294	–	–	–	–
Fig. 5; Fig. 10	T624	*Ramboldia cinnabarina*	*Spribille* 21549 (GZU)	Canada: British Columbia, Selkirk Mtns, Badshot Range, Healy/Hall divide	here, Resl *et al.* 2015	KR017140	KR017281	KR017229	–	MN437630	–	–	–
Fig. 5; Fig. 10	T1799	*Rhizocarpon haidense*	*Fryday* 10680 (MSC)	USA: Alaska, Kupreanof Island, Little Duncan Bay	here	MN483143	–	MN460227	MN508295	–	–	–	–
Fig. 5; Fig. 10	T1071	*R. oederi*	*Spribille* 36629 (MSC)	USA: Alaska, East Arm of Glacier Bay, Muir Inlet, Wolf Point	here	MN483144	–	MN460228	MN508296	MN437622	–	–	–
Fig. 7	T1184	*Sagiolechia phaeospora*	*Spribille* 38406 (MSC)	USA: Alaska, Glacier Bay National Park, Excursion Ridge	here	MN483145	–	MN460229	MN508297	MN437623	–	–	MN437645
Fig. 9	T1221	‘*Santessoniella*’ *arctophila*	*Spribille* 36663 (MSC)	USA: Alaska, Glacier Bay, near Rush Point	here	MN483146	–	MN460230	MN508298	MN437624	–	–	–
Fig. 5	P197	*Schaereria dolodes*	*Lendemer* 19748 (GZU)	USA	here	MN483148	MN508038	MN460231	MN508299	MN437625	–	–	–
Fig. 5	T1290	*S. dolodes*	*Spribille* 40649 (GZU)	USA: Montana, Sanders Co., lower Siegel Creek	here, Resl *et al.* 2015 (as ‘s. n.’)	KR017136	–	KR017224	KR017383	MN437629	KR017466	KR017524	KR017630
Fig. 5; Fig. 10	T1649	*Sphaerophorus globosus*	*Spribille* 41201 *& Holien* (GZU)	Norway: Nord-Trøndelag, Flatanger	here	MN483149	–	MN460232	MN508300	MN437626	–	–	–
Fig. 9	T1338	*Spilonema maritimum* (isotype material)	*Spribille* s. n., 21 Sept. 2010 (GZU)	USA: Alaska, Juneau Borough, west side of Douglas Island at Peterson Creek beach	here	–	–	–	MN508324	–	–	–	–
Fig. 9	L1727	*S. revertens*	*Wheeler* 3798a (GZU)	USA: Montana	here; Spribille *et al.* 2014*a*	MN483150	–	KC893667	KC893678	–	–	–	–
Fig. 5	T1393	*Sporastatia testudinea*	*Spribille* 27961	USA: Alaska, Mt Healy	here	MN483151	–	MN460233	–	–	–	–	–
*	NA	*Sporodictyon schaererianum*	*Fryday* 9977 (MSC)	USA: Alaska, Glacier Bay National Park, Queen Inlet	here	MN483099	–	–	–	–	–	–	–
*	NA	*Staurothele septentrionalis*	*Fryday* 9784 (MSC)	USA: Alaska, Glacier Bay National Park, Upper Muir Inlet	here	MN483100	–	–	–	–	–	–	–
*	NA	*S. septentrionalis*	*Fryday* 9794 (MSC)	USA: Alaska, Glacier Bay National Park, Upper Muir Inlet	here	MN483101	–	–	–	–	–	–	–
Fig. 9	T1187	*Steineropsis alaskana*	*Spribille* 38953 (MSC)	USA: Alaska, Glacier Bay National Park, Dundas Bay	here; Schneider *et al.* 2015	MN483152	–	KP794957	MN508301	–	–	–	–
Fig. 9	T1188	*S. laceratula*	*Spribille* 39570 (MSC)	USA: Alaska, Glacier Bay National Park, Taylor Bay	here; Schneider *et al*. 2015	MN483153	–	KP794958	MN508302	–	–	–	–
Fig. 5; Fig. 10	KS122	*Stereocaulon* sp.	*Resl* s. n. (2014) (GZU)	Iceland: Suðurland, highland W of Hofsjökull Glacier	here	MN483154	–	MN460234	MN508303	MN437627	–	–	–
*	T1334	*Stereocaulon* sp. S39567	*Spribille* 39567 (MSC)	USA: Alaska, Glacier Bay National Park, Taylor Bay	here	MN483080	–	–	–	–	–	–	–
*	T1280	*Sticta rhizinata*	*Spribille* 36814 (MSC)	USA: Alaska, Glacier Bay National Park, edge of Crane Flats	here	MN483081	–	–	MN508334	–	–	–	–
*	T1344	*Thelotrema lepadinum*	*Spribille* 39635 (MSC)	USA: Alaska, Glacier Bay National Park, Taylor Bay	here	MN483082	–	–	–	–	–	–	–
*	T1189	*Tingiopsidium* sp. AMF9804	*Fryday* 9805 (MSC)	USA: Alaska, Glacier Bay National Park, East Arm, Wolf Point	here	MN483083	–	MN460250	MN508335	MN437635	–	–	–
Fig. 5	T1294	*Toensbergia blastidiata*	*Tønsberg* 41670 (MSC—holotype)	USA: Alaska, Glacier Bay National Park, base of Marble Mountain	here	MN483155	–	MN460235	MN508304	–	–	–	–
Fig. 5	P132	*T. geminipara*	*Spribille* 38484 (MSC)	USA: Alaska, Glacier Bay National Park, West Arm, Ptarmigan Creek	here	–	MN508043	–	MN508325	–	–	MN437642	MN437652
Fig. 8	P151	*Trapelia coarctata*	*Aptroot* 66551 (hb. Aptroot)	St. Helena: High Peak, basalt cliff with trees	here	MN483156	–	MN460236	–	–	–	–	KU844403
Fig. 8	CP943	*Trapeliopsis flexuosa*	*Palice* 8079 *& O. Peksa* (hb. Palice; PRA)	Czech Republic: East Bohemia	here; Resl *et al*. 2015	KR017146	–	KR017232	KR017399	–	MN437640	KR017535	KR017596
Fig. 8	CP4286	*T. granulosa*	*J. Schön & C. Printzen* (FR)	Germany: Hessen, Stadt Frankfurt	here	MN483158	–	MN460238	MN508306	–	MN437637	–	–
Fig. 8	T1112	*T. granulosa*	*V. Wagner* plot 28.07.06/2 (UBC)	Canada: British Columbia, Mt. Revelstoke	here	MN483159	–	–	MN508307	–	–	–	–
Fig. 8	KS76	*T. granulosa*	*Resl* 1153 (GZU)	USA: Montana, Gallatin Co., Hyalite Canyon	Resl *et al.* 2015	KR017079	–	–	KR017315	–	–	–	KR017591
Fig. 8	P243	*T. gymnidiata*	*Ertz* 16241 (BR)	Spain: Canary Islands	here	MN483160	–	–	MN508261	–	–	–	–
Fig. 8	KS97	*Trapeliopsis* sp.	*Spribille* 40883 (GZU)	Canada: British Columbia, ‘The Kettle’ rapids, Clearwater River, N of Clearwater town	here	MN483162	MN508040	MN460239	MN508309	–	MN437638	–	MN437647
Fig. 8	KS87	*Trapeliopsis* sp. S40723	*Spribille* 40723 (MSC)	USA: Alaska, Glacier Bay National Park, Bartlett Cove	here	MN483161	MN508039	–	MN508308	MN437628	–	–	MN437646
*	T1081	*Tuckermannopsis chlorophylla*	*Spribille* 39112 (MSC)	USA: Alaska, Glacier Bay National Park, Bartlett Cove	here	MN483084	–	–	–	–	–	–	–
*	T1096	*T. chlorophylla*	*Spribille* 38192 (MSC)	USA: Alaska, Glacier Bay National Park, Fern Harbor	here	–	–	–	MN508339	–	–	–	–
*	T1080	*T. chlorophylla*	*Spribille* 38764 (MSC)	USA: Alaska, Glacier Bay National Park, Excursion Ridge	here	MN483085	–	–	–	–	–	–	–
*	T1211	*T. chlorophylla*	*Spribille* 36341 (MSC)	USA: Alaska, Glacier Bay National Park, Marble Mountain	here	MN483086	–	–	–	–	–	–	–
Fig. 5	T1324	*Umbilicaria polyphylla*	*Spribille* 40461 (GZU)	Austria: Styria, Zirbitzkogel, Großer Winterleitensee	here; Resl *et al.* 2015	MN483163	KR017276	KP794976	KR017390	–	–	–	KR017592
*	T1192	Unknown genus AMF10343	*Fryday* 10343 (MSC)	USA: Alaska, Glacier Bay National Park, Excursion Ridge	here	–	–	–	MN508336	–	–	–	–
Fig. 5; Fig. 6	P169	*Varicellaria rhodocarpa*	*Spribille* 29691 (GZU)	Canada: British Columbia, Muncho Lake, Alaska Hwy	here	MN483164	MN508041	MN460240	MN508310	–	MN437639	–	MN437648
Fig. 6	T1342	*Varicellaria* sp. S38337	*Spribille* 38337 (MSC)	USA: Alaska, Glacier Bay National Park, Excursion Ridge	here	MN483129	–	–	–	–	–	–	–
*	NA	*Verrucaria anziana*	*Fryday* 10286 (MSC)	USA: Alaska, Glacier Bay National Park, Falls Creek	here	MN483102	–	–	–	–	–	–	–
*	NA	*V. anziana*	*Fryday* 10455b (MSC)	USA: Alaska, Glacier Bay National Park, Taylor Bay	here	MN483103	–	–	–	–	–	–	–

### Phylogenetic trees

Phylogenetic analyses were used strictly to place newly described or remarkable species in larger groups, not to test species delimitations. We amplified DNA sequences from a total of 136 specimens for this study, including 83 collected in GLBA and adjacent areas (Table 1). We also used gene data from over 440 voucher specimens extracted for previous studies, as well as published genome projects available on the Joint Genome Institute MycoCosm website (https://genome.jgi.doe.gov/programs/fungi/index.jsf). Depending on the species, we included up to eight fungal loci, including ribosomal loci of the nucleus (ITS, 18S, 28S) and mitochondrion (12S) and nuclear protein-coding loci (*Mcm7*, *RPB1*, *RPB2*, *EF1a*). For newly generated sequences, primers, PCR conditions and locus abbreviations follow Resl *et al.* (2015) and Schneider *et al.* (2015). The decision on how many loci to sequence was informed by the available ‘background’ data in the NCBI nt database (‘GenBank’) for the larger taxonomic group in question. We assembled a private database of DNA sequences from GenBank and added an identifier code to each sequence to indicate the voucher it was derived from (typically a letter followed by three or four numbers, such as X123). Sequences from multiple loci, but one voucher, can thus be tied together and automatically called up for use in a tree. We combined this with MAFFT v7 (Katoh & Standley 2013) alignment and automated concatenation in the python-based phyloscripts pipeline (Resl 2015). Concatenation of DNA specimen data from different specimen vouchers was thereby eliminated. Automated concatenation based on an identifier code enabled quick testing of taxon samples for phylogenetic analysis. We included multiple samples of a taxon or group of closely related taxa if they had a ‘bridging’ locus in common, as this increased the number of loci represented for the resulting clade.

We further screened sequences with BLAST searches against the NCBI nt database to identify potential sequences from non-target fungi, even from already published sequence data. Based on this, we removed seven sequences from our data set: *Lecania atrynoides* 28S (AY756352) is identical to *Bryobilimbia hypnorum*; *Candelariella terrigena* mitochondrial 12S (DQ986884) appears to derive from a member of *Gyalectales* close to *Porina*; and *Micarea* (*Leimonis*) *erratica* 18S (KJ766742), 28S (KJ766591) and mitochondrial 12S (KJ766425) belong to an unknown member of *Lecideales*, not *M. erratica* (which is represented by other sequences in NCBI nt). *Lecanora achroa* 28S sequence JN939502 (Zhao *et al.* 2015) is a chimeric duplicate sequence of itself following ~position 651; because of uncertainty regarding the sequence identity, the entire sequence from this locus was deleted. Similarly, sequence HM576929 deposited by Zhao *et al.* (2015) as *Rhizoplaca shushanii Mcm7* protein in fact derives from the β-tubulin locus and was therefore not used. Major data sources and their underlying voucher specimens are listed in Table 1 and Supplementary Table S3 (available online).

Upon data set selection, we visually examined each alignment. For three sequences (P172, P173, T764) we removed several hundred base pairs from the 3′ end of the 28S sequence that was unalignable due to long introns and *c.* 150 bp of *Ramalina dilacerata* KP794953 due to poor quality. For the *Lecanorales* alignment, MAFFT failed to align a major 28S intron starting at position 932 of *Ramboldia arandensis* DQ431919; 24 of the 116 taxa in the 28S *Lecanorales* alignment possessed this homologous intron, the only section of any alignment that could not be handled by MAFFT and required manual adjustment. We then trimmed all sites from the alignment present in 10% or fewer sequences and subjected the trimmed alignment to a partition search using PartitionFinder v1.1.1 (Lanfear *et al.* 2012, 2016; v2.1.1 for the *Ostropales*/*Gyalectales* and *Sticta* data sets), using linked branch lengths, all available models, a ‘greedy’ search scheme, and the Bayesian Information Criterion for evaluating best model fit. The alignments were then used for maximum likelihood analyses using RAxML-HPC v8.0.0 (v.7.2.8 for *Pertusariales*) with 1000 bootstrap replicates and the GTRGAMMA model of nucleotide substitution for each partition (Stamatakis 2014).

### Species delimitation and nomenclature

As in the KLGO study, we based species identification more on systematic observation than on the *a priori* use of keys, that is, we sorted specimens into ‘morphospecies’ based on chemical and morphological characters *in statu symbiotico* and only then looked for applicable names in a global literature set. We continue to track ‘phantom phenotypes’ (Spribille 2018), distinct lichen symbiotic outcomes that may not be supported at the present time by DNA data from a small number of fungal gene loci. The reasons for this can be exemplified by the members of the *Bryoria implexa* group. Based on five gene loci and 18 microsatellite markers, Boluda *et al.* (2018) concluded that historically recognized members of this group are formed by one fungal species and thus, according to the *International Code of Nomenclature for Algae, Fungi and Plants* (Turland *et al.* 2018), the oldest valid name of this fungus should be used for all these lichens. We consider such a move premature, and the null hypothesis of genetic distinctness of these putative species impossible to reject at the current time, for the following three reasons. First, the existence of distinct multistate phenotypes, especially those that have been tracked with little controversy for over a century, is in itself evidence for genetically encoded biological phenomena; second, the biological basis for the formation of the phenotypes has neither been explained nor, to our knowledge, studied; third, the absence of evidence must not be confused with evidence of absence, in this case of phylogenetic signal in the ascomycete genome. Five loci represent less than 0.05% of the 10 000+ protein-coding genes that can be expected on a lecanoromycete genome (compare Armaleo *et al.* 2019).

Nomenclature of lichens and lichen-associated fungi largely follows Esslinger (2019) and Diederich *et al*. (2018), though two special cases merit comment: 1) we accept the need for segregate genera of *Caloplaca* and *Xanthoria* in *Teloschistaceae*, as outlined by Arup *et al.* (2013), but retain *Caloplaca* here in the broad sense with segregate names in parentheses since a) the combinations have not been made for approximately half of the taxa found in GLBA, and b) the names are not familiar to many users and we wish to avoid the confusion caused by moving closely related taxa to different parts of the main list; 2) we agree with Esslinger (2019) and do not follow the circumscription of cetrarioid genera derived from ‘temporal banding’ (Kraichak *et al.* 2017), for two reasons. First, temporal banding assumes that rates of phenotype evolution are linearly linked to rates of molecular evolution, but this is obviously not true across the tree of life or we would see as much phenotypic diversity in protists as we do in mammals (though extant members of both are at an equal distance to the most recent common ancestor in evolution). Second, unlike species, which are biological entities, genera are groupings of species that are alike from the human point of view, in recent years informed by what we have learned about common descent (monophyly). No imperative exists for these groupings to be equally old, nor does there exist a consensus on whether such an imperative would be desirable. Numerous other arguments against the adoption of temporal banding have been advanced by Lücking (2019). Our approach may be conservative, but it does not preclude rigorous hypothesis testing and the exploration of alternative nomenclatural solutions in the future.

Nomenclature of vascular plants follows *Flora of North America* (online treatments: http://www.efloras.org/flora_page.aspx?flora_id=1) with the exception of *Cupressus nootkatensis* (D. Don) Spach, which follows Gadek *et al.* (2000).

### Presentation of species data

Not all collections could be confidently assigned to a known species. The reasons for this are often complex and the story behind each ‘problem species’ reveals the challenges of working in poorly studied regions, as well as the interconnectedness of local taxonomic issues to broader global-level systematics. Replicating our approach in KLGO (Spribille *et al.* 2010), we present species in a hierarchical fashion here to allow the reader to navigate the results from the standpoint of their relative novelty and certainty. The results are presented in three groups: 1) taxa for which we have invested considerable effort to resolve their underlying systematic relationships, including species new to science; 2) ‘known unknowns’, putative species which we can characterize but for which we can neither find unambiguously applicable names nor assert with confidence that they are new species, or for which material is insufficient for a formal description; 3) lichen-forming and lichenicolous fungi for which we are more or less certain we can apply existing names (but see below). Unlike ‘known unknowns’, these latter species can be connected to a species name, even if this is done with caveats. Communicating to land managers, funders and other scientists the distinction between these types of taxonomic problems and the work they require is essential to building an appreciation of the role of systematics in the lichen inventory of poorly studied regions. We also consider it essential to report species of uncertain status, so the biodiversity of an area can be properly recorded, and other lichenologists can be alerted to their existence. We also hope this flags specimens from our study area to be included in other research, either current or in the future.

The list of taxa with names also includes some species for which the application of a name is uncertain. These are denoted with ‘cf.’ (for *confer*, the Latin imperative to compare) in cases where further studies, especially comparison with type material, would be advantageous; or ‘aff.’ (Latin: *ex affinitatis*) in cases where we or consulted experts have performed such studies and conclude that the species in question is in close affinity with, but not identical to, the type material. Of fungi, we exclude only yeast-forming microfungi associated with the lichen cortex, several of which have been detected in macrolichen samples from GLBA (Spribille *et al.* 2016), because surveying for these species requires special techniques and is beyond the scope of the present study.

After the species name, a brief summary of its observed ecological and elevational range in GLBA is provided, followed by an abbreviated list of specimens seen. Sector abbreviations are as noted above and waypoints are listed in Supplementary Material Table S2 (available online). Collection numbers reflect individual collectors based on initials: F = A. Fryday, M = M. Svensson, P = S. Pérez-Ortega, S = T. Spribille and T = T. Tønsberg. Records presented in the main and known unknown lists in square brackets (e.g. […]), denote localities outside the formal GLBA boundaries (most are within a few hundred metres of the formal park boundary). New species for Alaska are denoted with an asterisk (*) and for North America by a double asterisk (**); a hash symbol (#) denotes putatively lichenicolous fungi and a plus sign (+) putative saprobic fungi (we refer to these as ‘putative’ because our knowledge of their nutritional mode is derived from observations of fertile structures, not the whole mycelium or yeast stages).

### Comparison between sectors and national parks

To compare lichen composition of different areas, we constructed Venn diagrams using the R packages venn (https://cran.r-project.org/web/packages/venn/venn.pdf) and VennDiagram (Chen & Boutros 2011). We used species lists from McCune *et al.* (2018) for Katmai and Lake Clark National Parks and Preserves, and Spribille *et al.* (2010) for Klondike Gold Rush National Historical Park. Species lists were synonymized based on comparison of the application of names in the four studies and final reported numbers differ slightly from those in the original publications owing to deduplication of names in McCune *et al.* (2018) and follow-up studies since Spribille *et al.* (2010). The underlying matrix is presented in Supplementary Material Table S4 (available online). Maps to show park locations were generated using QGIS 3.10 (www.qgis.org), based on shapefiles downloaded from www.naturalearthdata.com and https://nrdata.nps.gov/programs/Lands/.

## Results and Discussion

We found a total of 947 species from the 4741 specimens collected. Ninety-eight could not be assigned to any named species. Of these 98, we have enough data to describe 27 as new to science. The remaining 71 species are reported as ‘known unknowns’. Of the 947 species reported, 831 are lichens, 96 are assumed non-mutualistic lichen-associated (lichenicolous) fungi and 20 are assumed non-mutualistic saprotrophic fungi. Thirty-eight previously described taxa are reported here for North America for the first time, and an additional 93 taxa are new reports for Alaska. The addition of 158 named taxa (27 + 38 + 93) to the known lichens and lichen-associated fungi of Alaska represents approximately a 9% increase in the collective Alaskan lichen-associated biota, which until now was estimated to contain *c*. 1750 taxa (unpublished data). All but 11 species (indicated in the main list in brackets) were found within the official GLBA boundaries, the others occurring on lands near the town of Gustavus. The survey accomplishes our twin goals of establishing a baseline inventory for GLBA and providing a georeferenced occurrence database for every species, which we analyze below at the level of park sectors. The number of lichen and associated fungal taxa we recorded in GLBA exceeds that of any US national park in the review of Spribille *et al.* (2010) or published since, and for the total number of taxa in study areas under 10 000 km^2^ worldwide, it is second only to the 1061 taxa found in Parc national des Cévennes, France (Roux *et al.* 2008), an area with decades of study investment.

### Comparison of sectors within GLBA

Individual sectors of GLBA differ greatly in their species composition (Fig. 4A; Supplementary material Table S4A, available online). The richest sector is Excursion Ridge with 438 taxa, followed by Gustavus and Dundas (both with 326), West Arm (248), Glacier Bay (232) and East Arm (189). Only 14 taxa were found in all sectors. The Excursion Ridge and Dundas sectors, which escaped glaciation in the Little Ice Age, together harbour 615 species, while all four glaciated sectors together harbour 607. If the Gustavus sector is instead lumped in with the unglaciated sectors, the first number climbs to 750 and the remaining unglaciated sectors drop to 452. This explains why parts of the Venn diagram (Fig. 4A) that exclude these three sectors, and display species found only in one or more of the remaining sectors, contain so few species. Excursion Ridge harbours the greatest number of unique species (i.e. species found only in one sector) with 198, while Glacier Bay (43) and East Arm (40) harbour the fewest. Collectively, the two unglaciated sectors hold 339 species not found in any glaciated sector, whilst glaciated sectors harbour 331 species not found in any unglaciated sector; again, the first number rises to 494 species if the Gustavus sector is instead lumped in with the unglaciated sectors, whilst the remaining glaciated sectors minus Gustavus have only 196 species. Why the Glacier Bay, West Arm and East Arm sectors harbour so few unique species, individually and collectively, cannot be directly determined from our data. These sectors account for the most recently deglaciated surfaces in GLBA but at the same time they were also the most remote and difficult to access during this survey. In contrast to the other three glaciated sectors, the Gustavas sector shares a long boundary with the unglaciated Excursion Ridge sector. Such proximity, providing easier opportunities for recolonization, could help explain the much higher species richness of the Gustavus sector compared to other glaciated sectors. That being said, the Gustavus sector was also easier to access.

**Fig. 4.**

A, Venn diagram of species occurrence within the six sectors of GLBA. Numbers do not add up to 947 because one species (*Melanohalea olivacea*) could not be assigned to any one sector due to a lack of site data. All species of lichens and lichen-associated fungi, including ‘known unknowns’, are included in this diagram. Where a number is absent from a segment, the value is zero; B, occurrence of named lichen species across four national parks and preserves in the Gulf of Alaska region (lichen-associated fungi and ‘known unknowns’ not included). Data is based on the present paper (Supplementary Material Table S4A & B, available online), Spribille *et al.* (2010) and McCune *et al.* (2018).

While numbers per sector will increase with further surveys, so too should the number of singleton species (those represented by only one specimen); we suspect the dissimilarity recorded between the sectors is real. However, results are skewed based on the kinds of sites that were accessible. The argillite outcrops of Excursion Ridge contained by far the richest sites found anywhere in GLBA. The sampling of such a site elsewhere in GLBA, if accessible, could lead to a significant rearrangement in the Venn diagram. We hypothesize that many factors (glacial history, vegetation succession and associated substratum availability and geological bedrock) drive richness patterns but inclusion of diverse sites within a sector would certainly affect the perceived richness distribution. Though our study was not designed to detect the impacts of air quality, we do not suspect a role for cruise ship emissions in the observed richness patterns. Cruise ship exhaust, to the extent it was observed, appears to linger in narrow passages of the West Arm in elevational belts well above sea level, sites inaccessible during the present survey.

### Lichen diversity in the national parks of the greater Gulf of Alaska region

Three other national parks in the greater Gulf of Alaska region (Fig. 1A) have been intensively surveyed for lichens in recent years: Klondike Gold Rush National Historical Park (KLGO: Spribille *et al.* 2010) and Katmai and Lake Clark National Parks and Preserves (McCune *et al.* 2018). Our collated lists of lichens and associated fungi from those parks, including revisions undertaken since (for KLGO), give total numbers of 757 (KLGO), 589 (Katmai) and 722 species (Lake Clark; lists in Supplementary Material Table S4B, available online). A four-way comparison of these national parks (Fig. 4B) provides an overview of the known collective lichen species pool and the species turnover along a 1000 km segment of the mountain chain that borders the Gulf of Alaska from Cook Inlet to the Icy Straits. A cumulative 1341 named lichen taxa occur in the four parks (GLBA 773, KLGO 604, Katmai 568 and Lake Clark 691; Fig. 4B). Comparisons for lichenicolous fungi and saprobic fungi and ‘known unknown’ lichens are not included above or in Fig. 4B because the first two groups were not specifically targeted in surveys of Katmai or Lake Clark (lichenicolous fungi: Katmai 9, Lake Clark 6; saprobic fungi: Katmai 2, Lake Clark 7) and the latter group is comparable only between GLBA and KLGO (though 10 ‘known unknown’ lichen species were reported from Katmai and 18 from Lake Clark). The cumulative number of lichenicolous fungal species in GLBA and KLGO is 147, and for ‘known unknown’ lichens 111 (all summary data in Supplementary Material Table S4B, available online).

Many taxa (617/46% of the 1341 named taxa) are found in only one park. GLBA has by far the highest number (248 taxa) followed by Lake Clark (160 taxa). This might reflect the relatively southern position of GLBA at the edge of the large temperate rainforest formation of south-east Alaska, and the position of Lake Clark on the opposite end of the northwest-southeast gradient. By contrast, only 192 (14%) of named taxa are found in all four parks (Fig. 4B). The large percentage of singletons—taxa found in only one park—underlines the importance of these protected areas in providing non-redundant habitat for lichen species. It also raises the question of how many species occur in natural landscapes of the Gulf of Alaska region that are not under any current form of protection.

### Phylogenetic trees

We obtained 280 new DNA sequences from the ascomycete fungal symbiont for specimens used in this study, most of them from specimens collected in GLBA (Table 1). A total of 223 were used in calculating phylogenetic trees together with previously published data. We calculated seven phylogenetic trees to provide context for placement of new species and ‘known unknowns’ when DNA data could be acquired. The taxon sample of each tree was designed to allow the exploration of placement of a sequence set across a broad cross-section of fungal evolution. In some cases, these are the first phylogenetic analyses to incorporate previously published, disparate data sets, and as a result, some new patterns emerge. Relationships specific to newly described species or ‘known unknowns’ are discussed under the treatments of those species but we highlight some of the broad patterns here, except for the *Hydropunctaria* tree which is discussed under the description of *Hydropunctaria alaskana*.

**Fig. 5.**
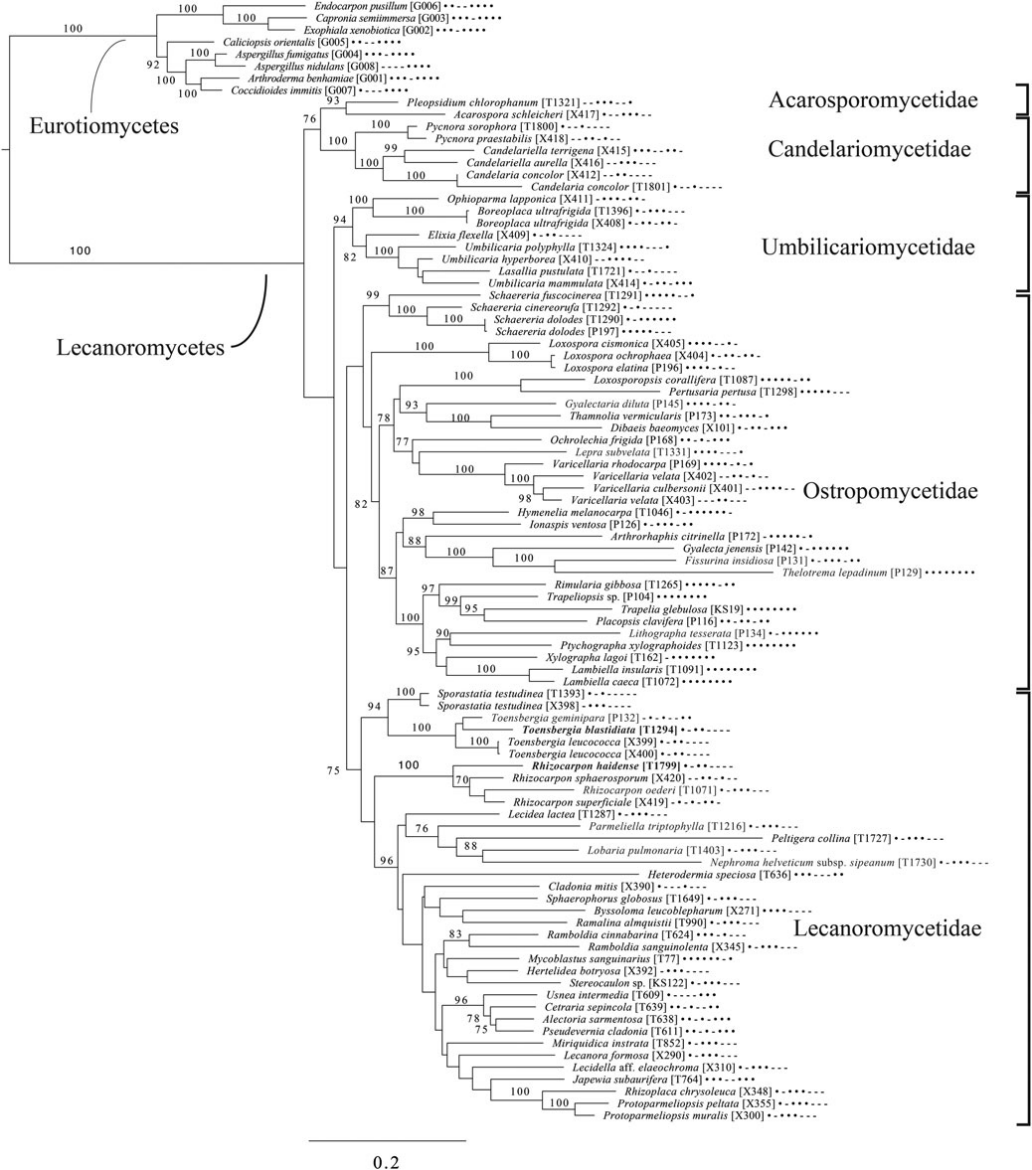
Majority-rule consensus tree of the class Lecanoromycetes, showing the placement of two new species (in bold) using selected voucher specimens and eight loci. Dots and dashes to the right of tip names indicate presence and absence of loci, respectively, in the following order: ITS, 18S, 28S, mtSSU, *Mcm7*, *RPB1*, *RPB2*, *EF1a*. Values indicate percent bootstrap support. Alphanumeric codes in brackets are identifiers unique to this study. Voucher information and GenBank Accession numbers are outlined in Table 1 and Supplementary Material Table S3 (available online).

**Fig. 6.**
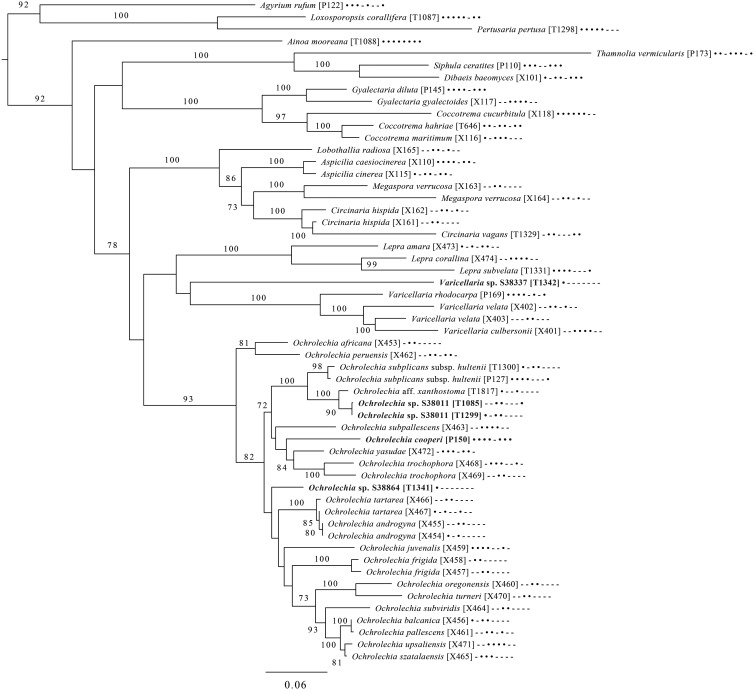
Majority-rule consensus tree of the order *Pertusariales* (subclass Ostropomycetidae) based on eight loci. Dots and dashes to the right of tip names indicate presence and absence of loci, respectively, in the following order: ITS, 18S, 28S, mtSSU, *Mcm7*, *RPB1*, *RPB2*, *EF1a*. Values indicate percent bootstrap support. Novel taxa are in bold. Alphanumeric codes in brackets are identifiers unique to this study. Voucher information and GenBank Accession numbers are outlined in Table 1 and Supplementary Material Table S3 (available online).

**Fig. 7.**
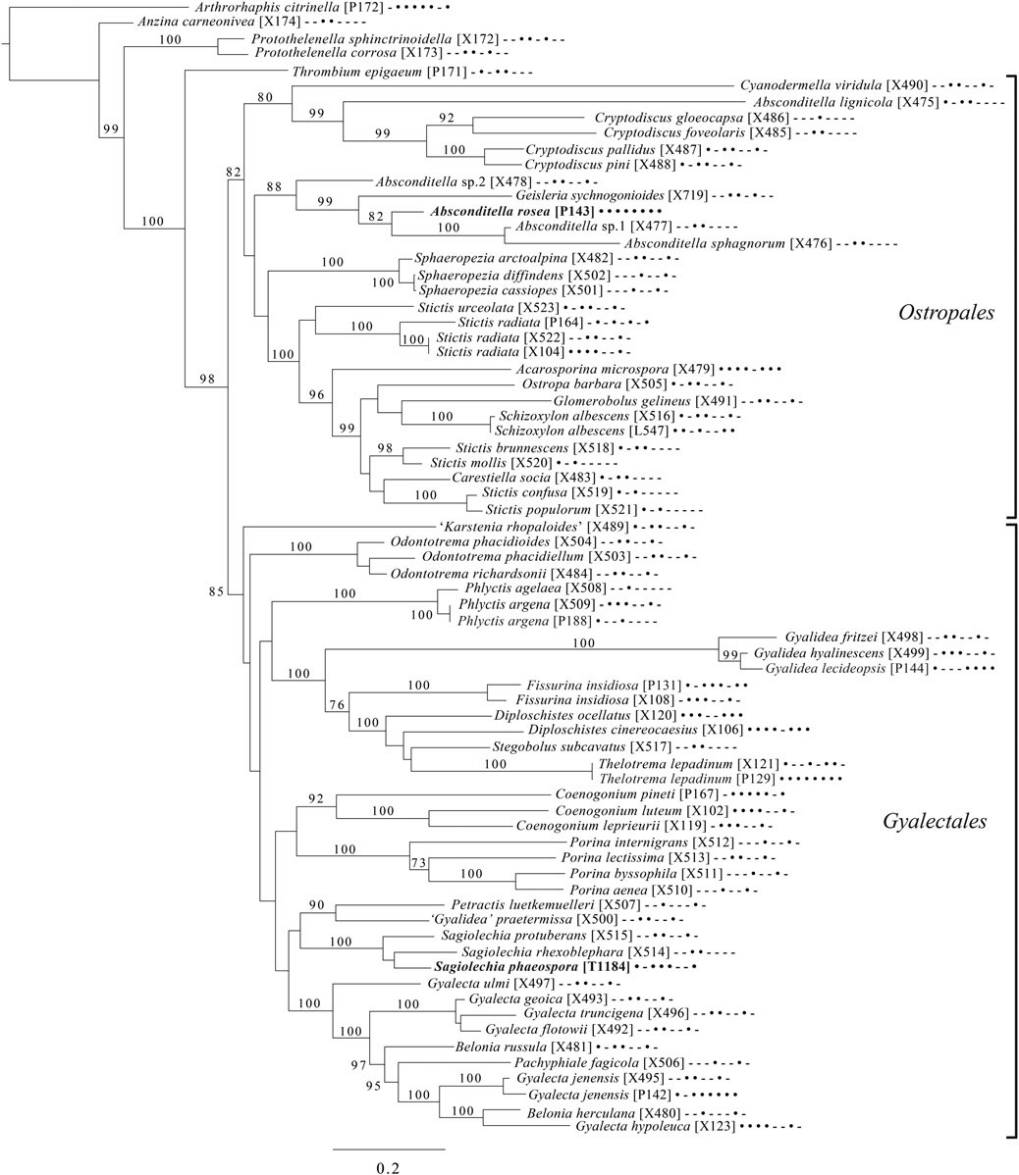
Majority-rule consensus tree of the orders *Ostropales* and *Gyalectales* (subclass Ostropomycetidae) showing placement of two new species (in bold) based on eight loci. Dots and dashes to the right of tip names indicate presence and absence of loci, respectively, in the following order: ITS, 18S, 28S, mtSSU, *Mcm7*, *RPB1*, *RPB2*, *EF1a*. Values indicate percent bootstrap support. Alphanumeric codes in brackets are identifiers unique to this study. Voucher information and GenBank Accession numbers are outlined in Table 1 and Supplementary Material Table S3 (available online).

**Fig. 8.**
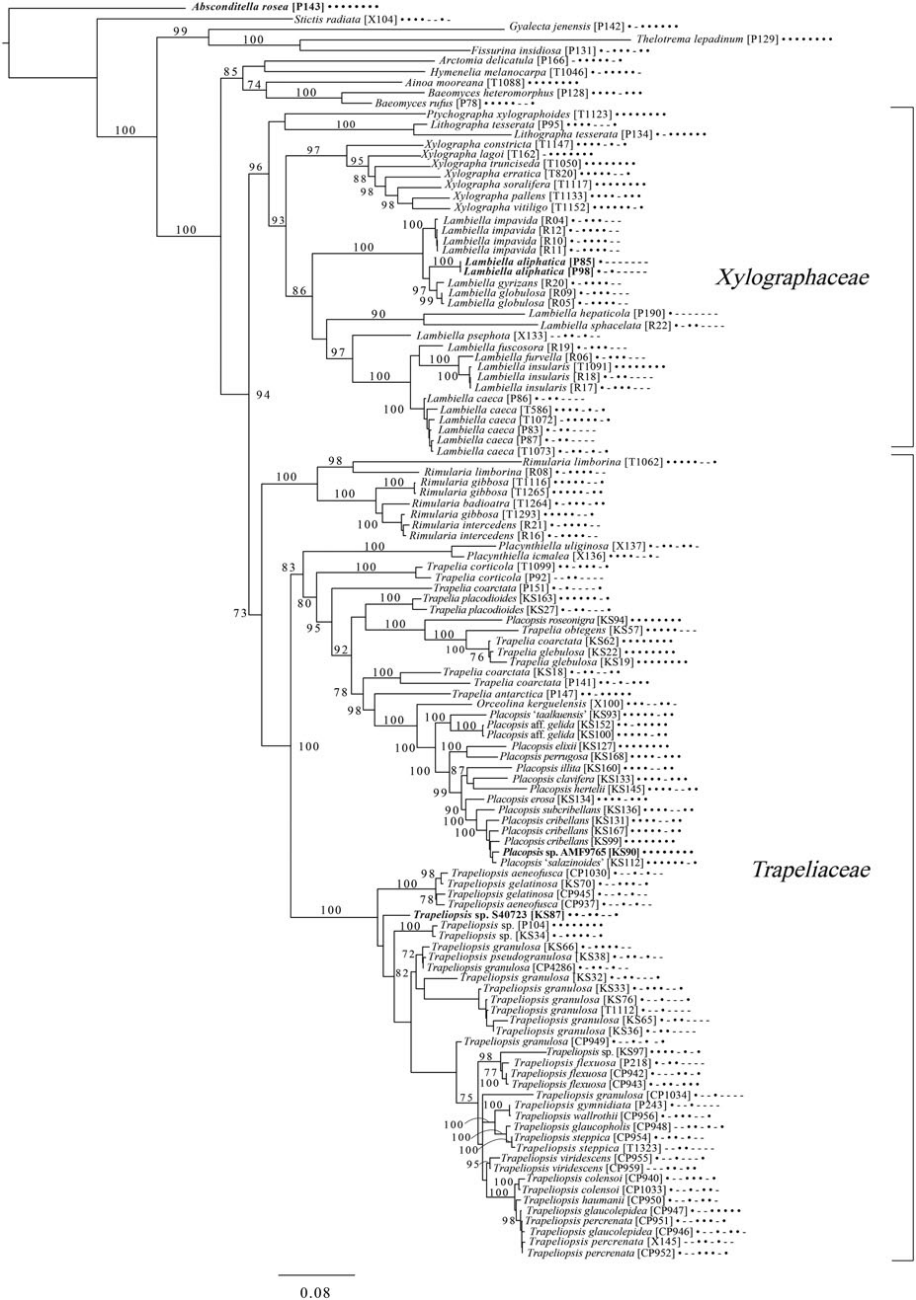
Majority-rule consensus tree of the order *Baeomycetales* (subclass Ostropomycetidae) based on eight loci. Dots and dashes to the right of tip names indicate presence and absence of loci, respectively, in the following order: ITS, 18S, 28S, mtSSU, *Mcm7*, *RPB1*, *RPB2*, *EF1a*. Values indicate percent bootstrap support. Novel taxa are in bold. Alphanumeric codes in brackets are identifiers unique to this study. Voucher information and GenBank Accession numbers are outlined in Table 1 and Supplementary Material Table S3 (available online).

**Fig. 9.**
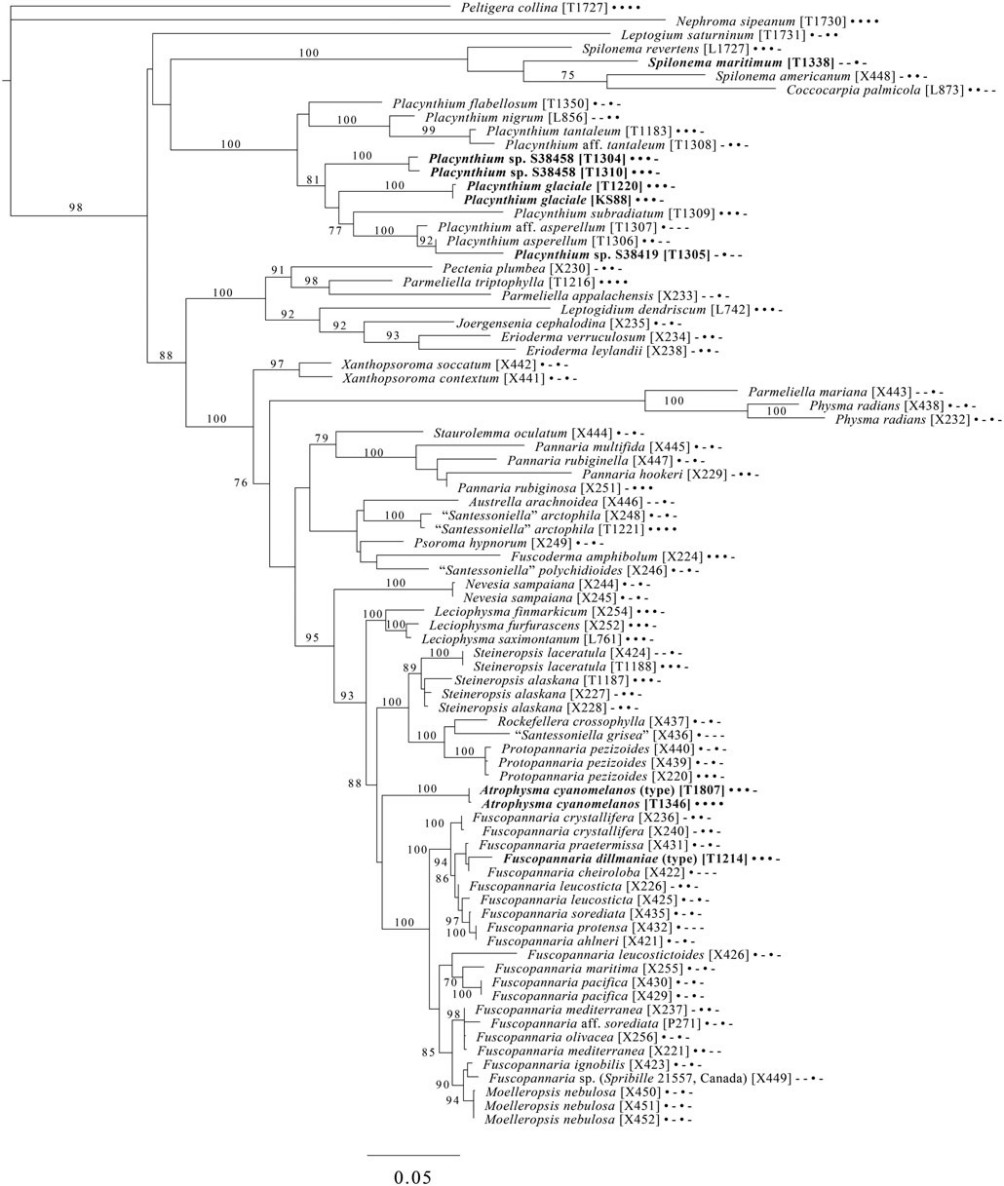
Majority-rule consensus tree of the suborder *Collematineae* (order *Peltigerales*) showing placement (in bold) of four new species and a ‘known unknown’ based on four loci. Dots and dashes to the right of tip names indicate presence and absence of loci, respectively, in the following order: ITS, 28S, mtSSU, *Mcm7*. Values indicate percent bootstrap support. Alphanumeric codes in brackets are identifiers unique to this study. Voucher information and GenBank Accession numbers are outlined in Table 1 and Supplementary Material Table S3 (available online).

**Fig. 10.**
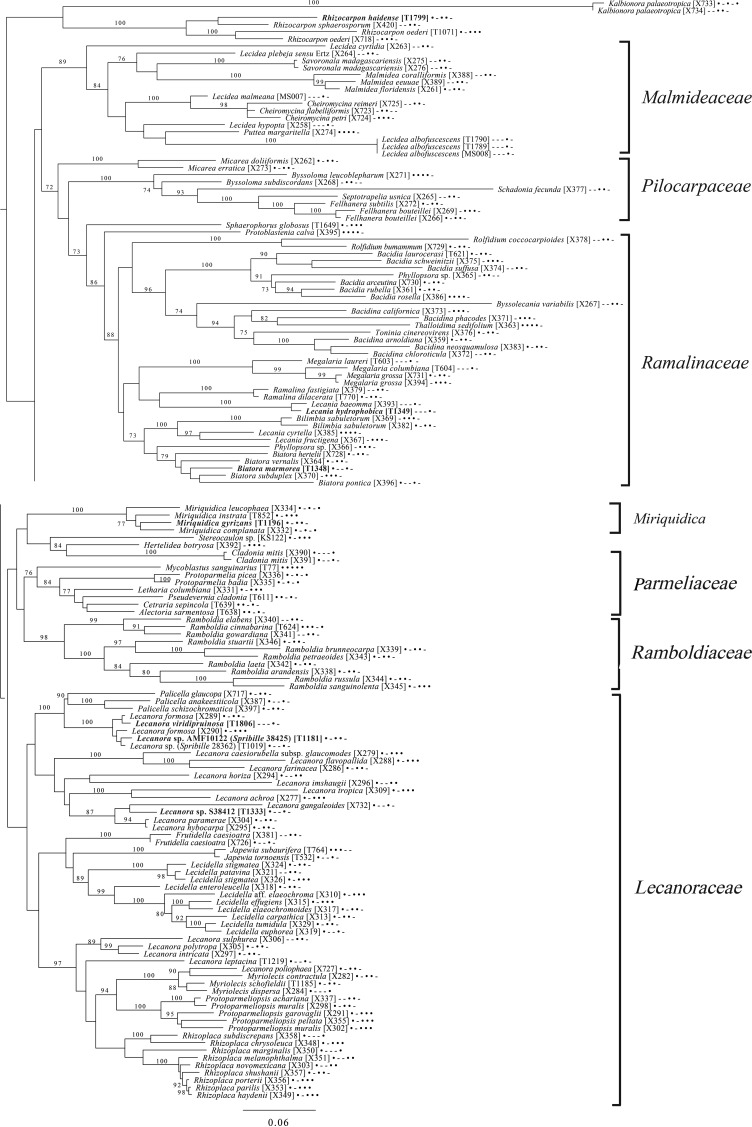
Majority-rule consensus tree of the order *Lecanorales* (subclass Lecanoromycetidae) showing placement (in bold) of three new species and several ‘known unknowns’ or previously poorly understood taxa, based on five loci. Dots and dashes to the right of tip names indicate presence and absence of loci, respectively, in the following order: ITS, 18S, 28S, mtSSU, *Mcm7*. Values indicate percent bootstrap support. Alphanumeric codes in brackets are identifiers unique to this study. Voucher information and GenBank Accession numbers are outlined in Table 1 and Supplementary Material Table S3 (available online).

The broadest evolutionary taxon sample includes representatives of the entire class Lecanoromycetes with Eurotiomycetes as an outgroup (Fig. 5) based on eight loci. This provides context for five of the remaining phylogenetic trees (Figs 6–10) as well as several clades not treated in those analyses. The overall topology largely recapitulates known relationships but provides for the placement of two species placed here in the hitherto monotypic genus *Toensbergia* (*Sporastatiaceae*), a relationship that had not been suspected based on morphological data.

An eight-locus phylogenetic tree of *Pertusariales* (Fig. 6) includes representatives of major genera that have been previously sampled, as well as representatives of the main groups within the genus *Ochrolechia*. This analysis places a sterile ‘known unknown’ in the *Lepra-Varicellaria* clade (*Varicellaria* sp. S38337) and another sterile sample in *Ochrolechia* (*Ochrolechia* sp. S38864). It places the newly described *Ochrolechia cooperi* relative to other species in that genus, provides evidence for the close relationship of the putatively undescribed *Ochrolechia* sp. S38011 to the alectoronic acid-containing species of *Ochrolechia* with closed ascomata (*O. subplicans*, *O. xanthostoma*), and finally provides evidence for the monophyly of that species group and its position within, not outside of, *Ochrolechia* as currently circumscribed.

An eight-locus phylogenetic tree (Fig. 7) of the clade of Ostropomycetidae circumscribed as the order *Ostropales* s. lat. includes many of the species sampled by Baloch *et al.* (2010), augmented with data from Resl *et al.* (2015), Schneider *et al.* (2016) and new data. It places *Absconditella rosea* in the *Absconditella* clade (as opposed to *Cryptodiscus*) and the new species *Sagiolechia phaeospora* in a clade with *S. protuberans* and *S. rhexoblephara*. The expanded locus and taxon sampling recovers reciprocal monophyly of a clade of predominantly saprotrophic genera that include *Ostropa barbara* on the one hand, and a clade of mainly lichen-forming groups including the well-studied families *Graphidaceae*, *Gyalectaceae* and *Porinaceae* on the other. The second clade encompasses many of the same genera placed in *Gyalectaceae* and the order *Gyalectales* (Hawksworth & Eriksson 1986; see also the overview by Gagarina 2015) as well as the *Graphidales*. The split we found is better resolved than in previous studies (Kauff & Lutzoni 2002; Baloch *et al.* 2010) and could be taken as support for the recognition of a single order including the families *Coenogoniaceae*, *Graphidaceae*, *Gyalectaceae, Porinaceae* and *Phlyctidaceae*, and the *Odontotrema* clade of *Ostropaceae*. Our analysis provides a larger sample of *Ostropales* and *Gyalectales* than the recently published five-locus data set of Kraichak *et al.* (2018), but we recover a broadly similar topology. Kraichak *et al.* (2018) included a considerably larger taxon sample of *Diploschistaceae, Fissurinaceae*, *Graphidaceae* and *Thelotremataceae*, which they recognize as constituting an order of their own (*Graphidales*).

An eight-locus phylogenetic tree of *Baeomycetales* (Fig. 8) relies heavily on data generated by Resl *et al.* (2015, 2018) and Schneider *et al.* (2016) and recovers almost the same topology as the first study. It places the newly described *Lambiella aliphatica* as well as a previously unpublished sequence from a Chilean specimen of the otherwise Australasian *Lambiella hepaticola* and ‘known unknowns’ from genera *Placopsis* and *Trapeliopsis*.

A five-locus phylogenetic tree of the *Peltigerales* suborder Collematineae (Fig. 9) relies heavily on data from Ekman *et al.* (2014) but is expanded to include newly generated, as well as published, sequences from *Coccocarpiaceae* and *Placynthiaceae* (Spribille & Muggia 2013; Spribille *et al.* 2014*a*). The tree places the newly described *Spilonema maritimum* in a monophyletic clade with *Coccocarpia* and *Spilonema*, suggesting that more work is needed on the relationships between the lecanoromycete mycobionts in those lichens. The newly described *Placynthium glaciale* is recovered within a strongly supported *Placynthium* clade despite its muriform ascospores, a first for that genus. The sampling also enables us to place the new genus *Atrophysma* as a distinct clade among previously sampled members of *Pannariaceae* and the newly described *Fuscopannaria dillmaniae* in the genus *Fuscopannaria*.

The final lecanoromycete tree is of the order *Lecanorales* and is based on five loci (Fig. 10). To construct this tree, we screened GenBank for sequences from *Lecanoraceae, Malmideaceae, Pilocarpaceae* and *Ramalinaceae* and chose taxa that, either alone or in combination with multiple replicates of the same or closely related taxa, covered as many of the five loci as possible. The objective was to build a topology that covered phylogenetic structure in all the main groups of *Lecanorales* and explored the relationships of several sequence sets we recovered from GLBA. The taxon sample, especially for *Lecanoraceae*, relied heavily on published sequences from Zhao *et al.* (2015). Because of our interest in the placement of the *Lecidea albofuscescens* group and a suspected relationship with *Malmideaceae*, we included as much multilocus data from that family as was available. Despite the lacunose sequence coverage, we recovered the *Malmideaceae* as a well-supported, monophyletic clade including *L. albofuscescens* as well as *Lecidea malmeana*, a polysporous species collected in GLBA. Of species that have been previously reported to belong to *Malmideaceae*, only *Kalbionora* (Sodamuk *et al.* 2017) was not recovered within this clade, instead grouping in an unsupported relationship with the outgroup *Rhizocarpon*. We refrain from undertaking any nomenclatural changes because of poor internal support within *Malmideaceae*, and ongoing studies.

Also in *Lecanorales*, the newly described species *Lecania hydrophobica*, *Biatora marmorea* and *Miriquidica gyrizans* grouped with *Lecania baeomma* and the genera *Biatora* and *Miriquidica*, respectively, as expected based on morphological analyses (Fig. 10). Zhao *et al.* (2015) did not include the recently described genus *Palicella* in their *Lecanoraceae* analyses, but in our topology, the species referred here are recovered as a strongly supported monophyletic group within a poorly supported *Lecanoraceae*. Our analysis confirms the recent result of Kondratyuk *et al.* (2019), in that even the narrow *Palicella* clade encompasses a saxicolous taxon (*Palicella anakeestiicola* S.Y. Kondr. *et al*.) and is sister to a clade of saxicolous species with similar chemistry and pigments, until now called the *Lecanora formosa* group. One of our newly described species, *Lecanora viridipruinosa*, and one ‘known unknown’ (*Lecanora* sp. F10122) resolve within this group. We refrain from making any new combinations because we could not find accessions with enough loci to represent the *Lecanora varia* group, an important group of species with an older genus name (*Straminella* Choisy), and thus cannot eliminate the possibility that some or more of these species may be assignable there. Another ‘known unknown’, *Lecanora* sp. S38412, resolves within a supported clade referable to *Lecanora* s. str., in proximity to *Lecanora gangaleoides*, as expected by morphochemical analysis. *Lecanora leptacina* is recovered on its own branch in a clade that includes the *Lecanora polytropa* group, *Myriolecis* (now treated in part as *Polyozosia*; Kondratyuk *et al.* 2019), *Protoparmeliopsis* and *Rhizoplaca*. Finally, a new sequence set from *Myriolecis schofieldii* resolves as expected within that clade, as well as a recently published sequence of *Lecanora poliophaea* (Kistenich *et al.* 2018).

A further 57 DNA sequences were generated for species not included in any phylogenetic analysis here, in most cases because we were unsuccessful in obtaining multiple loci and no meaningful analysis could be conducted with a single locus in conjunction with published data. We publish the sequences (Table 1) because single locus data published here might match up against future sequences from multilocus and barcoding data sets.

## Descriptions of New Genera and Species

### Atrophysma T. Sprib. gen. nov.

MycoBank No.: MB 830090

A cyanolichen with minutely coralloid, finger-like lobes over a black hypothallus, similar to *Placynthium* but ascospores are simple, similar to *Leciophysma* but with dark blue-black pigments in the apothecium; asci lacking an amyloid apical tube.

Type: *Atrophysma cyanomelanos* T. Sprib. (below).

#### Etymology

The genus name comes from *atra* (black), a reference to its colour impression in the field, and –*physma*, thought to derive from the Greek verb *physao*, to blow up or distend, and the suffix –*ma*, indicating a completed action (Verdon 1992).

### Atrophysma cyanomelanos T. Sprib. sp. nov.

MycoBank No.: MB 830091

A cyanolichen with minutely coralloid, finger-like lobes over a black hypothallus, black apothecia, internally with a black pigment, reversibly HNO_3_+ mauve, KOH+ remaining blackish but weakly greenish tinged, and simple ascospores, 11.0–16.0 × 7.1–8.1 μm, frequently with a warted gelatinous epispore.

Type: USA, Alaska, Hoonah-Angoon Census Area, Glacier Bay National Park, Excursion Ridge, ridgetop, 58.46503°N, 135.55757°W, 903 m, saxicolous on argillite slabs on alpine ridgetop covered by deep snow much of the year, 1 August 2012, *Spribille* 39425 (MSC—holotype; NY—isotype).

(Fig. 11)

**Fig. 11.**

*Atrophysma cyanomelanos* (holotype). A & B, habit; C, habit with SEM; D, broken thallus lobe with SEM; E, broken thallus lobe in brightfield microscopy; F, ascoma section (composite image); G, ascus stained in Lugol's solution. Scales: A = 1 mm; B & C = 0.5 mm; D & F = 50 μm; E & G = 10 μm.

*Thallus* a sprawling crust up to 7 cm diam., becoming confluent with adjacent thalli, olivaceous brown, consisting of minute coralloid fingers 70–150 μm diam.; *hypothallus* present as a black base to the coralloid fingers, but not extending beyond the perimeter of the main thallus, coloured with the same pigment as the apothecia (see below); individual lobes consisting of tightly packed *Nostoc*-like cyanobacterial cells with fungal hyphae sheathed in a gelatinous cortex-like layer, a cellular cortex lacking.

*Ascomata* apothecia, round, sometimes flexuose, single or grouped, (0.25–)0.5–0.6(–1.3) mm diam., often absent; *disc* flat to more often convex, jet black, matt, sometimes hollowed out (herbivory?) leaving concave shells; *proper margin* prominent, receding with age but not disappearing, matt. *Excipulum* 60–90 μm wide laterally, to 40 μm wide basally, composed of radiating, anastomosing hyphae that widen towards the tips, up to 8–9 μm diam. with lumina to 3–5 μm, streaked with black pigments externally, POL+ crystals not seen. *Hymenium* (60–)70–90 μm tall, hazy reddish brown, I+ wine red before KOH treatment, uppermost part (‘epihymenium’) lacking crystals, heavily black-pigmented, the pigment reversibly HNO_3_+ mauve, KOH+ remaining blackish but weakly greenish tinged, similar to the ascomatal pigments in *Farnoldia* and the hypothallus pigments in *Placynthium*; *paraphyses* mostly simple, 2–4 μm wide at midpoint, not widened apically, moniliform. *Hypothecium* to 200 μm thick, hyaline or light reddish brown, grading in the lowermost 50–150 μm to deep brown, I+ wine red. *Asci* 8-spored, widely flask-shaped, lightly amyloid externally, I− internally, lacking an amyloid apical tube or tholus; *ascospores* simple, broadly ellipsoid, (10–)11.0–16.0(–19) × (5.5–)7.1–8.1(–9.5) μm, frequently with a warted gelatinous epispore (*n* = 60, from six specimens).

*Pycnidia* not observed.

#### Chemistry

No secondary substances detected.

#### Etymology

From *kyanos* (blue) and *melas* (black), referring to the characteristic contrasting colours of the ascomata and thallus upon close examination.

#### Habitat

On rock, apparently preferring weakly calcareous rock (in GLBA, argillite) in subalpine and alpine habitats.

#### Notes

We first encountered this species working in KLGO and tentatively assigned it, as a ‘known unknown’, to the genus *Santessoniella* Henssen (Spribille *et al.* 2010, as *Santessoniella* sp. 24535). The GLBA material is much richer and gave us a broader basis for morphological as well as DNA analysis but placing the new species into an existing genus proved impossible. Ekman *et al.* (2014) showed that *Santessoniella* as circumscribed by Henssen (1997) is polyphyletic and its characteristic thallus morphology evolved independently. In addition, the type of the genus, *S. polychidioides* (Zahlbr.) Henssen, has been recovered within *Psoroma* Ach. ex Michx. with moderate support, suggesting that the former genus will be lost to synonymy. We also suspected a relationship to *Leciophysma* Th. Fr., but species of that genus have a distinct I+ apical tube in the ascus (such as in *L. saximontana* (T. Sprib. *et al.*) P. M. Jørg. *et al.*, a species initially described in *Santessoniella* but later placed in *Leciophysma*; Spribille *et al.* 2007; Ekman *et al.* 2014). We also know of no species of *Pannariaceae* with the black pigments of this species, which recall those in *Placynthiaceae*. We considered a possible placement in the latter family but no *Placynthiaceae* are known to have simple ascospores. Multilocus DNA sampling from the apothecia of the new species placed it in the *Pannariaceae* (Fig. 9). A continued discussion of species formerly placed in *Santessoniella* can be found under the treatment of *Fuscopannaria dillmaniae* later in this paper.

We observed a wide variation in ascospore sizes within the limited material available to us, so much so that we initially suspected we might have two species. To at least cursorily test this, we sequenced ITS rDNA from both a specimen with large ascospores and one with small (the type) and found no difference between the two.

*Atrophysma cyanomelanos* is currently known only from Alaska. We have also seen a specimen from the Brooks Range in northern Alaska (below).

#### Additional specimens examined

**USA:** *Alaska*: Klondike Gold Rush National Historical Park, 2007, *Spribille* 24535 (KLGO); west side of White Pass, 2008, *Spribille* 26967 (KLGO, L853), 26968 (KLGO, voucher L947); Hoonah-Angoon Census Area, Glacier Bay National Park, Excursion Ridge, 58.46274°N, 135.55288°W, 919 m, saxicolous on soft argillite, 2012, *Spribille* 38414 (MSC); *ibid.*, 58.46222°N, 135.55954°W, 883 m, small rock in tundra, 2012, *Spribille* 38770 (MSC); *ibid.*, 58°27.810′N, 135°33.485′W, 2012, *Svensson* 2660 (MSC); *ibid*., 58.46503°N, 135.55757°W, 2012, *Spribille* 39435 (MSC); *ibid.*, 58.46469°N, 135.55736°W, 918 m, saxicolous, 2012, *Fryday* 10338 (MSC, topotype); *ibid.*, *Spribille* 39384 (MSC), 39388 (MSC), 39402 (NY; DNA voucher 1346); Gates of the Arctic National Park, northern Brooks Range, Summit Lake, 68.0495226°N, 150.5257256°W, 1140 m, saxicolous on sandstone/quartzite cobbles, 2012, *T. Wheeler* 4271 (hb. Wheeler).

### Bacidina circumpulla S. Ekman sp. nov.

MycoBank No.: MB 830092

Thallus of ± placodioid, pale greyish, yellowish or brownish squamules that never form goniocysts or soralia. Apothecia biatorine, mostly flat, with a pinkish, beige, ±brown, greyish to almost black and often piebald disc and a ± greyish black and slightly shiny margin. Proper exciple thin, paraplectenchymatous, diffusely reddish brown and/or dirty green in at least the uppermost part. Hymenium colourless in lower part, diffusely and unevenly reddish brown and dirty green in upper part and in scattered vertical streaks. Hypothecium colourless to pale yellowish. Ascospores straight, curved to shallowly helical, acicular, mostly with 3–5 thin septa.

Type: USA, Alaska, Hoonah-Angoon Census Area, Glacier Bay National Park, Queen Inlet, shoreline, 58.88500°N, 136.50838°W, 0–5 m, on rotting driftwood log, 19 July 2012, *Svensson* 2540 (NY—holotype; MSC—isotype).

(Fig. 12)

**Fig. 12.**

*Bacidina circumpulla*. A, part of thallus with pale and medium dark apothecia (holotype); B, thallus with dark-pigmented apothecia (*Fryday* 10017); C, section of relatively pale apothecium, with brown pigment in upper part of proper exciple and irregularly in hymenium (holotype); D, section of dark apothecium with more pigment in exciple and hymenium, including some green pigment in upper part of exciple (mixed with the brown) (holotype). Scales: A & B = 0.5 mm; C & D = 50 μm.

*Thallus* crustose, consisting of firm, ±placodioid, discrete, contiguous, or overlapping, sometimes imbricate, squamules. *Squamules* up to 350 μm wide, adnate and flattened or somewhat raised when overlapping, pale greyish, yellowish, or brownish, matt, not forming goniocysts or soralia. *Prothallus* thin and endosubstratal, whitish, present along edge of thallus or lacking. *Photobiont* chlorococcoid, cells rounded to ellipsoidal, 8–18 μm long, single or in clusters.

*Apothecia* scattered over thallus or aggregated, biatorine, broadly sessile, 0.2–0.4–0.7 mm diam. (*s* = 0.1, *n* = 40), flat, remaining so or becoming convex with age, without pruina, often strikingly variable in colour within the same thallus; *disc* dirty pinkish or pale beige to dark reddish or olive-brown to dark pinkish grey to almost black, often piebald; *proper margin* with dark pigment (appearing greyish black) in a ring around the paler disc, otherwise with colours similar to the disc, somewhat shiny, distinct and raised in young apothecia, soon level with the disc, ±persistent or later partially excluded in convex apothecia. *Proper exciple* 30–35 μm thick, without crystals, paraplectenchymatous, diffusely reddish brown and/or dirty green in at least the uppermost part, in dark apothecia with reddish brown pigment also along the edge and in the innermost part bordering the hypothecium, otherwise ±colourless, composed of radiating, dichotomously branched hyphae with moderately gelatinized walls; cell lumina in upper part of exciple narrowly ellipsoid (up to 9 μm long and 3 μm wide), wider and ±ellipsoid in lower part (up to 14 μm long and 6 μm wide), sometimes somewhat expanding terminally. *Hymenium* 49–54–59 μm tall (*s* = 3, *n* = 20), colourless in lower part, diffusely and unevenly reddish brown and dirty green in upper part and in scattered vertical streaks, pigment mostly concentrated around groups of paraphyses and young asci; *paraphyses* fairly abundant, in approximately equal proportion to number of asci, 1.5–2.3 μm wide in mid-hymenium, unbranched or sparingly branched in upper part; apices ±clavate, 2.3–3.6–5.4 μm wide (*s* = 0.8, *n* = 70), without gelatinous cap or internal pigment. *Hypothecium* colourless to pale yellowish. *Asci* clavate, 8-spored, approximately of *Bacidia* type *sensu* Hafellner (1984); young spore mass not forming ocular chamber, apex above young spore mass staining dark blue in IKI with a widely and bluntly conical axial body staining pale blue; *ascospores* colourless, without perispore or ornamentation, acicular, straight, curved or shallowly helical, 26–37–54 μm long (*s* = 6, *n* = 70), 1.6–2.2–3.1 μm wide (*s* = 0.3, *n* = 70), with (0–)3–5(–7) thin septa.

*Pycnidia* scattered, immersed in thallus with protruding ostiole, globose, unpigmented except for a dark ring of reddish brown pigment around the ostiole, 60–100 μm diam., unilocular; *conidiophores* lining inside of cavity, terminated by cylindrical to narrowly clavate conidiogenous cells, 3.5–6.0 × 1.5–2.3 μm. *Conidia* acrogenously formed, filiform, curved (but not hooked), non-septate, 7–13 × 0.7–1.0 μm.

#### Chemistry

All spot tests negative. No substances detected by HPTLC (Arup *et al.* 1993).

#### Pigments

Laurocerasi-brown (reddish brown in H_2_O, KOH+ purplish, N+ orange-red) in proper exciple, hymenium and pycnidial wall, Bagliettoana-green (green in H_2_O, KOH− then HCl+ purple, HNO_3_+ purple) in hymenium and uppermost part of proper exciple (Meyer & Printzen 2000), and possibly sometimes small amounts of Rubella-orange (yellow to orange in H_2_O, KOH+ intensifying, HNO_3_+ intensifying) in hypothecium (Ekman 1996).

#### Etymology

The epithet *circumpulla* (nominative singular *circumpullus*) alludes to the shiny black ring formed by pigment in the uppermost part of the apothecial margin, surrounding the often paler disc.

#### Habitat

Known from two localities in the western United States and Canada: one in inland British Columbia and one in GLBA. At the first locality, it was found overgrowing a decaying polypore in a swamp and at the other locality the exposed, soft wood of a log near the seashore.

#### Notes

*Bacidina circumpulla* is readily distinguished from all other species of the genus by its thallus, resembling a flattened miniature version of *Bilimbia lobulata* (Sommerf.) Hafellner & Coppins, and the apothecia that are superficially similar to the apothecia of *Cliostomum griffithii* (Sm.) Coppins, including the striking colour variation even within the same thallus. Unlike many members of the genus, the thallus never dissolves into goniocysts and generally lacks greenish hues when dry. *Bacidina circumpulla* shares the microsquamulose habit and the mixture of brown and green apothecial pigments with *B. neosquamulosa* (Aptroot & van Herk 1999) which, however, possesses a thicker apothecial margin and a greenish thallus composed of deeply incised microsquamules that sometimes disintegrate to form patches with goniocysts.

‘*Bacidina circumpulla* Ekman & Spribille ined.’ reported by McCune *et al.* (2018) does not belong here but rather to the taxon named *Bacidia friesiana* by Ekman (1996).

#### Additional specimens examined

**Canada:** *British Columbia*: Clearwater Valley, ‘Edgewood West’, 51°52.0′N, 120°01.8′W, overgrowing polypore fungus in swamp forest, 2006, *Björk* 13219 (UBC).—**USA:** *Alaska*: Hoonah-Angoon Census Area, Glacier Bay National Park, Queen Inlet, shoreline, 58.8770°N, 136.5060°W, 0–5 m, rotting log, 2012, *Fryday* 10016, 10017 (MSC—topotypes).

### Biatora marmorea T. Sprib. sp. nov.

MycoBank No.: MB 830093

Similar to *Biatora sphaeroidiza* but differing in the deposition of pigment as distinct granules around the tips of the paraphyses, by the presence of a prominent dark hypothallus, and by the apothecial margin which remains white, prominent and not excluded at maturity.

Type: USA, Alaska, Hoonah-Angoon Census Area, Glacier Bay National Park, west side of Glacier Bay, base of Marble Mountain directly opposite Drake Island, 58°37.894′N, 136°14.639′W, corticolous on large, old *Oplopanax horridus* in dense beach fringe thicket of *Alnus incana*, just above sea level, 3 July 2012, *Spribille* 38009, *Pérez-Ortega & Tønsberg* (MSC—holotype; NY—isotype).

(Fig. 13)

**Fig. 13.**

*Biatora marmorea* (holotype). A & B, habitus; C, section through hymenium and hypothecium, arrows indicate pigment incrustations on paraphysis tips; D, section through excipulum; E, asci and ascospores in Lugol's solution. Scales: A = 1 mm; B = 100 μm; C–E = 10 μm.

*Thallus* crustose, rimose to weakly areolate at the thallus margin, smooth, the individual areoles flat to weakly convex, creamish white, 0.2–0.4 mm diam.; thin, 50–120 μm in section, weakly stratified, with medullary hyphae strongly birefringent under polarized light, cortex scarcely differentiated, <15 μm thick, biofilm-like; sterile thalline hyphae I+ gold; *hypothallus* prominent, dark bluish to blue-black. *Photobiont* chlorococcoid, abundant; *cells* 6–10 μm diam.

*Ascomata* apothecia, round, single or in pairs, (0.3–)0.4–0.5(–0.7) mm diam.; *disc* convex, variably creamish white, aeruginose, blue-black to black depending on the exposure of the apothecia and intensity of pigment, with light-exposed sides of apothecia darker, matt; *proper margin* prominent, biatoroid, white to whitish to pale grey, becoming ±excluded as viewed from above, matt. *Excipulum* 35–40 μm wide laterally and 60–65 μm wide basally, composed of radiating, thick-walled hyphae with locally broadened lumina (Fig. 13D), embedded in a heavily gelatinized layer extending up to 10 μm beyond the hyphal tips, lacking crystals as viewed in polarized light. *Hymenium* hyaline except for the paraphysis tips, 45–60 μm tall, I+ blue changing to rust red with increasing iodine concentration, I+ blue following pretreatment with KOH, lacking crystals in polarized light; *paraphyses* straight, simple to weakly branched, 2 μm wide medianly, gradually widening to 3.5 μm apically, encrusted with bluish black pigment granules (Fig. 13C), these HCl+ blue, reversibly HNO_3_+ mauve ↔ KOH+ greenish, sequentially HNO_3_+ mauve −> HCl+ unchanged −> KOH dissolving yellowish, and HNO_3_+ mauve −> KOH+ strong green fading to yellowish −> HCl+ unchanged (similar to Cinereorufa-green, Printzen & Tønsberg (2003)). *Hypothecium* 130–150 μm tall, with algal cells frequently wedged between hypothecium and lower inner excipulum, giving the section a lecanorine appearance, hyaline above with a very pale brownish subhymenial layer visible *c.* 50–60 μm thick below. *Asci Bacidia*-type to *Biatora*-type (Fig. 13E), 8-spored, 25–27 × 6–12 μm; *ascospores* simple, narrowly ellipsoid, (8.0–)9.8–10.7(–12.5) × (2.5–)2.9–3.2(–4.5) μm (*n* = 23 over two collections).

*Conidiomata* not seen.

#### Chemistry

Thallus C−, KOH−, Pd−, UV+ pale orange. A dominant xanthone, perhaps thiophanic acid, with a secondary unidentified xanthone, detected by TLC.

#### Etymology

Named after the type locality and, so far, only known location, Marble Mountain (Latin ‘*marmor*’, marble). Also a fanciful reference to the marbled pigmentation of the apothecial discs.

#### Habitat

Corticolous on the bases of *Oplopanax horridus* (Smith) Miquel and bark of *Alnus incana* (L.) Moench subsp. *tenuifolia* (Nutt.) Breitung.

#### Notes

We were initially unsure of the genus assignment of this species because of the presence of algal cells between the lower hypothecium and lower excipulum in many sections, suggesting an affinity to *Lecania* A. Massal. s. lat. *Biatora marmorea* has an ascomatal habit (Fig. 13B) resembling *Myrionora* R. C. Harris (Palice *et al.* 2013; now also included in *Biatora*, Kistenich *et al.* 2018) or species of the *B. beckhausii* group (Printzen 2014), and DNA sequences confirmed its placement in *Biatora* (Fig. 10). *Biatora marmorea* appears to be closest, at least morphologically and chemically, to *B. sphaeroidiza* Printzen & Holien (Printzen 1995). It shares the chemical profile of *B. sphaeroidiza* in TLC but does not closely resemble the species in habit. In *B. sphaeroidiza*, the apothecia quickly become convex and emarginate with age, but in *B. marmorea* they maintain a prominent whitish margin which contrasts strongly with the pigmented disc (Fig. 13B). Most striking in the field is the presence of a dark blue-black hypothallus (Fig. 13A), which *B. sphaeroidiza* lacks. At the microscopic level, *B. marmorea* contains a pigment similar in reaction type to that present in *B. sphaeroidiza*, but it is concentrated in granules around the tips of the paraphyses that lead the apothecia to appear greenish speckled from the outside, although blackish under the light microscope. In addition, it has a thicker hymenium (45–60 μm) than *B. sphaeroidiza* (30–40 μm). The species is also genetically distinct from *B. sphaeroidiza* in a test phylogeny of only *Biatora* species (C. Printzen, personal communication 2017).

Oddly, despite the heavy collecting on *Alnus* and *Oplopanax* elsewhere in GLBA, this species was found in only one place, on the shoreline at the base of Marble Mountain. Here, however, it was abundant.

#### Additional specimens examined

**USA:** *Alaska*: Hoonah-Angoon Census Area, Glacier Bay National Park, west side of Glacier Bay, base of Marble Mtn directly opposite Drake Island, 58°37.894′N, 136°14.639′W, corticolous on *Oplopanax horridus*, 2011, *Spribille* 36364 *& Fryday* (MSC, topotype); *ibid.*, corticolous on *Alnus incana* subsp. *tenuifolia*, sea level, 2012, *Spribille* 38015, *Pérez-Ortega & Tønsberg* (MSC, topotype); *ibid*., corticolous on *Oplopanax horridus*, 2012, *Tønsberg* 41650 (MSC, topotype).

### Carneothele Fryday, T. Sprib. & M. Svenss. gen. nov.

MycoBank No.: MB 830094

Similar to *Thelocarpon* but with red-brown ascomata, the wall pigment forming magenta crystals in 10% KOH.

Type: *Carneothele sphagnicola* Fryday, M. Svenss. & Holien (see below).

#### Etymology

From the Latin *carnalis* (‘of the flesh’) and –*thele* (Gr.: nipple), a reference to the shape and colour of the ascomata.

### Carneothele sphagnicola Fryday, M. Svenss. & Holien sp. nov.

MycoBank No.: MB 830095

Thallus biofilm-like, coating *Sphagnum* mosses; ascomata red-brown, perithecioid, the wall pigment forming magenta crystals in 10% KOH, attenuating to a narrow ostiole, with asci 220–250 × 30–35 μm, polysporous, containing ellipsoid ascospores.

Type: USA, Alaska, Petersburg Borough, Mitkof Island, ‘Towers muskeg’, 56.672750°N, 132.918500°W, 10 m, *Sphagnum* bog (muskeg) with *Oxycoccus oxycoccos*, 1 September 2014, *Fryday* 10667, *K. Dillman & Spribille* (MSC—holotype, E—isotype).

(Fig. 14)

**Fig. 14.**

*Carneothele sphagnicola* (A & B from *Spribille* 40821; C–F from *Spribille* 40824). A & B, habitus; C, section through ascoma; D, ascomatal section in Lugol's solution, showing deeply amyloid hymenial region and adjacent non-amyloid zone; E, ascoma in K, demonstrating dendritic crystals; F, ascospores in Lugol's solution, showing characteristic amyloid reaction. Scales: A & B = 0.5 mm; C–E = 50 μm; F = 10 μm.

*Thallus* biofilm-like. *Photobiont* present as scattered bundles of green algal cells, 20–60 μm across, present around the base of the perithecia; individual cells orbicular, 5–9 μm diam.

*Ascomata* perithecioid, scattered, brick red, occasionally with light yellow pruina around the ostiole, becoming pale brown in the herbarium, flask-shaped, 0.20–0.25–0.30 mm diam., 0.4–0.5 mm tall, ½ to ⅔ immersed in substratum. *Ascomatal wall* hyaline (blue in 10% HCl), composed of longitudinally arranged hyphae 2–2.5 μm thick but with numerous, minute, golden brown crystals that dissolve in KOH to give a fleeting magenta solution followed by the formation of ± rectangular or dendroid magenta crystals, mostly *c*. 10–18 μm across but up to 30 × 10 μm, crystals not dissolving in 10% HCl or 50% HNO_3_, but becoming golden brown. *Hamathecium* composed of numerous, slender (*c*. 1 μm thick), lax, unbranched filaments, I−. *Asci* 220–250 × 30–35 μm, slightly clavate at base, gradually tapering to a narrow apex, wall I+ blue, tholus I+ blue with a narrow, I− ocular chamber; *ascospores* unicellular, numerous (>200) per ascus, broadly ellipsoid with pointed ends, 9–10 × 4–5 μm, amyloid (IKI+ blue).

#### Chemistry

Thallus spot tests negative, ascomatal wall with KOH+ magenta crystals (see below), HNO_3_− (50% solution), HCl− (15% solution). No substances detected by TLC.

#### Etymology

A reference to the apparently obligate occurrence on the tops of *Sphagnum* hummocks.

#### Habitat

Found only on the dry tops of *Sphagnum* hummocks, apparently most frequent on *S. fuscum* (Schimp.) Klinggr. At the type locality it occurs in a distinctive community with *Absconditella sphagnorum* Vězda & Poelt, as well as an undescribed ascomycete superficially resembling *Geltingia associata* (Th. Fr.) Alstrup & D. Hawksw. and *Epibryon* sp.

#### Notes

*Carneothele sphagnicola* is a highly distinctive species that defied placement in any known genera. It is apparently close to *Thelocarpon*, with which it shares the minute ascomata on organic substrata with the occasional presence of a yellow pruina, plus the multi-spored asci that gradually taper to a narrow apex. However, it differs from that genus in the more robust red-brown ascomata with the wall pigment forming magenta crystals in 10% KOH.

*Carneothele sphagnicola* is readily recognizable in the field on account of its ascomata that, when wet, resemble little pointed pieces of raw red meat as viewed through a hand lens (Fig. 14B), and which dot patches of moribund *Sphagnum*. Microscopically it is characterized by its multi-spored asci and the ascomatal wall containing small orange crystals that dissolve in KOH to form different, magenta crystals. Using standard 10% KOH these crystals are ±rectangular (*c*. 20–30 × 5–10 μm) but with a higher concentration of KOH, long, branching (dendroid) needle-shaped crystals are formed (Fig. 14E).

In our efforts to find an existing genus in which to place the new species, we shared material and photographs with several specialists (O. Eriksson, Sweden; P. Döbbeler, Germany; B. Coppins, UK; A. Rossman, USA). One of us (HH) recognized the species from material collected in oceanic bogs in Trøndelag, Norway. We have since found the species outside of GLBA in muskeg on Mitkof Island, which we have designated the type locality based on the greater abundance of material collected from this site. We expect it to be widespread in such habitats in SE Alaska and possibly in other temperate rainforest regions of the world. Indeed, the associated ascomycete resembling *Geltingia associata* and the *Epibryon* sp. have recently been collected from a similar habitat in Newfoundland, NE Canada, although *C. sphagnicola* was not seen (J. M. McCarthy, personal communication).

Attempts to establish the evolutionary relationships of *Carneothele sphagnicola* using DNA have so far not been successful. The one extraction that has yielded usable DNA produced ITS and 28S rDNA sequences with affinities to Dothideomycetes and *Sarea*, respectively (isolate T1110, Table 1), but it is not certain whether these sequences derive from the fungus that forms the fruiting bodies described here.

#### Additional specimens examined

**USA:** *Alaska*: Hoonah-Angoon Census Area, Glacier Bay National Park, NE of Gustavus, Falls Creek area, ‘Yellowlegs Savanna’ muskeg, 58.44367°N, 135.60583°W, terricolous on wet ground next to flark, 250 m, 2012, *Spribille* 38741 (MSC), *Fryday* 10046 (MSC), *Svensson* 2577 (MSC); *ibid.*, 58.44742°N, 135.60593°W, 245 m, *Spribille* 38738 (MSC, NY); Petersburg Borough, Mitkof Island, muskeg *c.* 0.6 km S of Papkes Landing Road, W side of highway behind radio towers, 56.673015°N, 132.916128°W, 9 m, 2014, *Spribille* 40824, *Fryday & Dillman* (GZU, UPS, topotypes); *ibid.*, Twin Creek muskeg, 56.723250°N, 132.905500°W, 2014, *Spribille* 40821, *Fryday & Dillman* (H).—**Norway:** *S-Trøndelag*: Åfjord, by Lake Måmyrvatnet, Nesodden, 64.09798°N, 10.54964°E, 260 m, on dead *Sphagnum fuscum* in ombrotrophic mire, 2016, *Holien* 15270 & 15318 (TRH). *N-Trøndelag*: Steinkjer, W of lakelet Svarttjønna, Jernblaestermyra, 64°03.01′N, 11°31.61′E, 200 m, on dead *Sphagnum* in ombrotrophic mire, somewhat eroded and trampled patches, 2008, *Holien* 11927 (TRH); *ibid*., 2012, *Holien* 13916 (TRH), 13917 (MSC); Meråker, N of Sulåmoen, E of Litlåa, 63.5649°N, 11.9442°E, 440 m, on dead *Sphagnum fuscum* in ombrotrophic mire, 2013, *Holien* 14221 (TRH).

### Cirrenalia lichenicola Pérez-Ort. sp. nov.

MycoBank No.: MB 830096

Similar to *Cirrenalia caffra* Matsush. but differing in the thinner diameter of the filaments, conidia with fewer average number of septa, and different substratum.

Type: USA, Alaska, Hoonah-Angoon Census Area, Glacier Bay National Park and Preserve, along the trail from Bartlett Cove to Point Gustavus, near campground, 58°26′43″N, 135°53′05″W, on sterile unidentified corticolous crust on *Alnus*, 7 July 2012, *Pérez-Ortega* 2284 (US–holotype).

(Fig. 15)

**Fig. 15.**

*Cirrenalia lichenicola* (holotype). A, sporodochia; B, conidia; C, detail of a coiled conidium (B & C in water, using DIC microscopy). Scales: A = 100 μm; B = 20 μm; C = 5 μm.

Colonies growing on a sorediate crustose lichen, forming groups of sporodochia, black, up to 100 μm diam. *Mycelium* scanty, superficial or immersed in the host, hyaline or slightly brown. *Conidiophores* micronematous or semi-macronematous, short, acrogenous or more rarely arising laterally on hyphae, simple or very rarely dichotomously branched, straight or more rarely flexuous, pale to dark brown, smooth, 4–6 μm wide; *conidiogenous cells* monoblastic, terminal; *conidia* acrogenous, solitary, dry, helicoid, contorted one time, smooth-walled, dark brown when mature, light brown when young, septa 4–7, not or slightly constricted at the septa, apex obtuse, basal parts usually tapering, 8–12 μm, filaments 2.5–4.5(–6.0) μm wide (*n* = 24).

#### Etymology

Named for its lichenicolous occurrence.

#### Habitat

On an unidentified whitish sorediate crustose lichen; material too scant for chemical tests.

#### Notes

The genus *Cirrenalia* Meyers & R. T. Moore has been used for species of dematiaceous fungi characterized by helicoid conidia, usually constricted at the septa (Zhao & Liu 2005). Initially, the genus contained only marine species but it was expanded (Sutton 1973) to include terrestrial species and the number of accepted terrestrial and marine species is now similar. The genus has been recently studied using molecular markers and been shown to be highly polyphyletic (Abdel-Wahab *et al.* 2010). Compared to species treated in the overview provided by Zhao & Liu (2005), *Cirrenalia lichenicola* is characterized by the small size of the conidia and the narrow filaments. *Cirrenalia caffra* Matsush. is similar to the new species although the filament is slightly wider and the conidia are also larger. *Cirrenalia lignicola* differs in having more coiled conidia and the presence of up to 12 septa. *Cirrenalia lichenicola* is known only from the type specimen (Fig. 15).

### Corticifraga nephromatis Pérez-Ort. sp. nov.

MycoBank No.: MB 830097

Lichenicolous on *Nephroma bellum*. Differing from morphologically similar species by the presence of non-septate ascospores that are ellipsoid with acute ends to teardrop-shaped (dacryoid).

Type: USA, Alaska, Hoonah-Angoon Census Area, East Arm of Glacier Bay, mouth of unnamed creek E of Muir Point, 58.83642°N, 136.05313°W, 8 m, 30 July 2012, *Spribille* 39257 (US—holotype).

(Fig. 16)

**Fig. 16.**
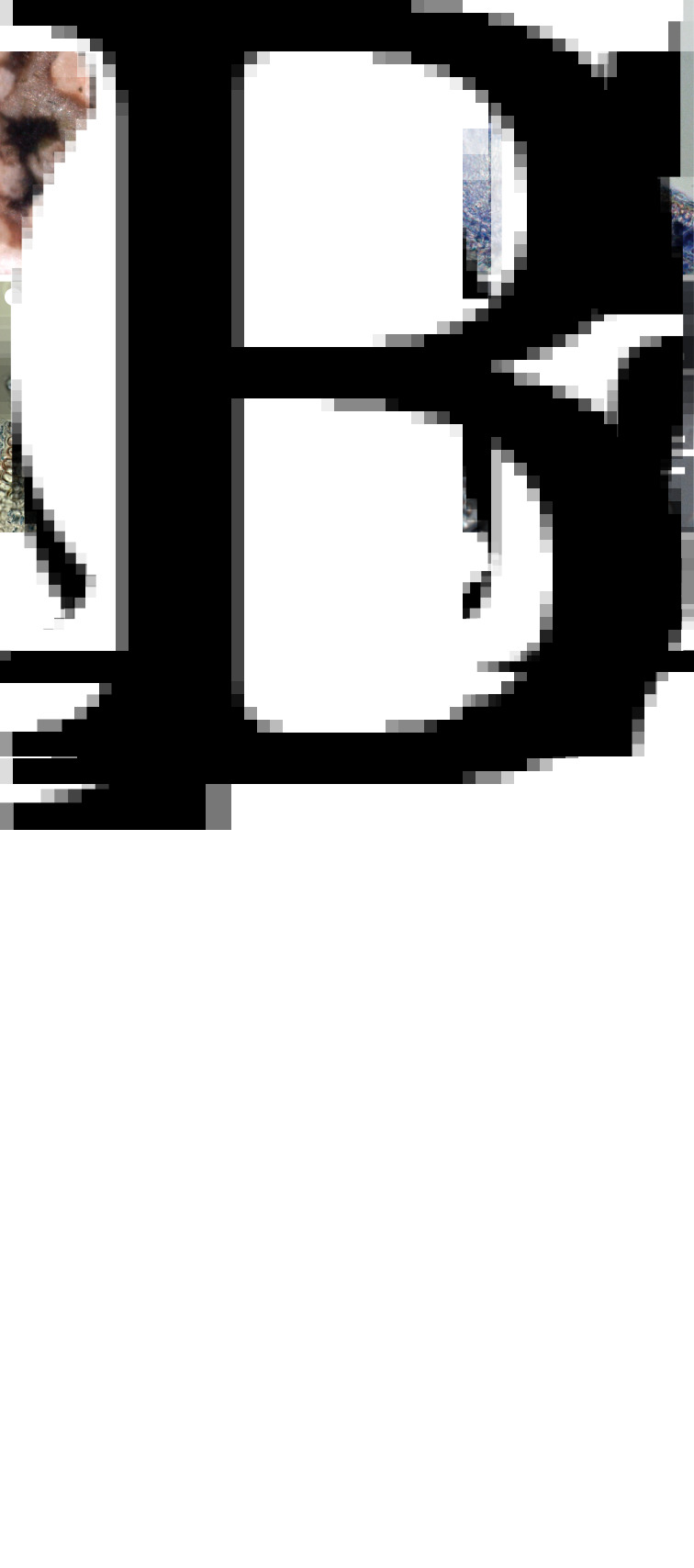
*Corticifraga nephromatis* (holotype). A, ascomata; B, transverse section of ascoma; C, detail of hymenium and excipulum; D, paraphyses; E, young ascus; F, mature ascus; G–I, ascospores (B & C in lactophenol blue; D–I in water using DIC microscopy). Scales: A = 200 μm; B = 50 μm; C = 25 μm; D = 10 μm; E–I = 5 μm.

*Apothecia* delimited, scattered or more rarely confluent, originating from splits in the host cortex, irregularly roundish to angular, 0.10–0.25 mm diam.; *disc* usually at the same level as the thallus surface or slightly raised, cream-coloured with the exciple usually lighter than the disc. *Exciple* colourless, up to 25 μm wide but usually reduced. *Hymenium* colourless, 50–70 μm tall, I−, KI−; *paraphyses* straight, not branched, 2–3 μm wide, not capitate but increasing in diameter, gradually reaching up to 5 μm at the apex. *Hypothecium* hyaline, up to 85 μm tall. *Asci* bitunicate, clavate to subcylindrical, with a small ocular chamber seen in immature asci, 25–34 × 5–8 μm (*n* = 10), 8-spored, I−, KI−; *ascospores* simple, from ellipsoid with acute ends to teardrop-shaped (dacryoid), colourless, smooth-walled, (10–)11–12 × 3–4(–5) μm (*n* = 25).

#### Etymology

Named for its occurrence on *Nephroma*.

#### Habitat

On *Nephroma bellum* (Spreng.) Tuck.

#### Notes

Four species of *Corticifraga* D. Hawksw. & R. Sant. are known from SE Alaska. *Corticifraga fuckelii* (Rehm) D. Hawksw. & R. Sant. and *C. peltigerae* (Fuckel) D. Hawksw. & R. Sant. are common on species of *Peltigera*, and recently *C. scrobiculatae* Pérez-Ort. was described from *Lobarina scrobiculata* (Spribille *et al.* 2010) and discovered in GLBA during the present survey. *Corticifraga chugachiana* Zhurb. was described from the Chugach National Forest as one of the few lichenicolous fungi to occur on *Lobaria oregana* (Zhurbenko 2007). *Corticifraga nephromatis* (Fig. 16) is the second species of the genus growing on *Nephroma* Ach. *Corticifraga santessonii* Zhurb. may grow on *Nephroma occultum*, but it is readily differentiated from *C. nephromatis* by its dark-coloured ascomata and 3-septate ascospores. Furthermore, the new taxon is distinguished from other species in the genus by the presence of ellipsoid to dacryoid, simple ascospores and broader paraphyses. The recently described genus *Taitaia* Suija *et al*. shows clear affinities with *Corticifraga*, from which it is separated based on molecular data and the typically aggregated apothecia (Suija *et al.* 2018). *Taitaia aurea* Suija *et al*. possesses apothecia of a similar colour to those of *C. nephromatis*. It differs, however, in the presence of 1-septate ascospores and the aggregated apothecia, as well as the occurrence on *Crocodia* cf. *clathrata* (DeNot.) Trev.

#### Additional specimen examined

**USA:** *Alaska*: Hoonah-Angoon Census Area, East Arm of Glacier Bay, mouth of unnamed creek E of Muir Point, 58.83642°N, 136.05313°W, 8 m, 2012, *Spribille* 39259 (MSC—topotype).

### Fuscidea muskeg Tønsberg & M. Zahradn. sp. nov.

MycoBank No.: MB 830098

Similar to *Fuscidea praeruptorum* (Du Rietz & H. Magn.) V. Wirth & Vězda in being sorediate and producing alectorialic acid, but distinct from that species by the medianly constricted, shorter (7–10 μm) ascospores (vs bean-shaped and slightly longer (9.5–12 μm) in *F. praeruptorum*) and the corticolous habit (vs saxicolous).

Type: USA, Alaska, Hoonah-Angoon Census Area, along and N of road from Gustavus to Bartlett Cove, near entrance sign for Glacier Bay National Park and Preserve, 58°27.19′N, 135°46.64′W, corticolous on branches of *Pinus contorta* Dougl. ex Loudon subsp. *contorta* in muskeg, 1 July 2012, *Tønsberg* 41576 (MSC–holotype; NY–isotypes).

(Fig. 17)

**Fig. 17.**

*Fuscidea muskeg* (holotype). A, habitus; B, young apothecia with SEM, showing surface of surrounding thallus; C, surface of thallus wart from (B) with SEM; D, surface of excipulum from (B) with SEM, showing short hyphal ‘spines’; E, section of apothecium; F, ascus and ascospores (DIC). Scales: A = 2 mm; B = 200 μm; C & E = 50 μm; D = 20 μm; F = 10 μm.

*Thallus* crustose, areolate, usually forming rounded patches to a few cm diam., rarely larger, to 0.5 mm thick. *Hypothallus* often distinct as ramifying hyphae bordering the thallus and also evident as pale brown pigmentation of the substratum between thallus areoles. *Esorediate areoles* discrete, pale greenish to greenish with a yellowish tinge (becoming brown or pinkish in the herbarium), convex, 0.12–0.20 mm diam., soon bursting apically to form soralia. *Soralia* concolorous with esorediate parts, discrete at first, later some becoming confluent, rarely forming a leprose crust throughout. *Soredia* mostly aggregated in irregularly rounded consoredia, 14–55 μm wide. *Photobiont* trebouxioid, cells (8–)11–15(–18) μm diam.

*Apothecia* often absent, occasionally abundant, pale to dark brown, discrete, regularly to irregularly rounded, to 1.0 mm diam., attached to the thallus only at the centre of the base; *disc* pale to dark brown, more or less flat; *proper margin* concolorous with disc or, sometimes, paler brown than disc or pale greenish brown, raised above the disc, flexuose, ±involute, 0.06–0.08 mm wide, bearing spiny protrusions visible in SEM (Fig. 17D). *Excipulum* rim 60–65 μm wide, brown along the edges, colourless inside; *cells* rounded and thin-walled; *walls* brown in pigmented parts; lumina wide, 7–10 × 6–7 μm. *Hymenium* colourless or, rarely, brownish, 48–65 μm tall; *paraphyses* sparingly anastomosed and, especially in upper part, branched, 1.5–2 μm wide; apical cells enlarged, to (2–)3(–4) μm, with a pigmented cap. *Hypothecium* colourless, 25–52 μm tall. *Asci* clavate, 27–45 × 10–12 μm; *ascospores* 7–10 × (3–)4–5 μm (*n* = 22), simple or rarely 1-septate, medianly constricted (Fig. 17F).

*Pycnidia* brown, convex, 0.06–0.12 mm wide; *conidia* dacryoid, 3 × 1.5–2 μm.

#### Chemistry

Thallus PD+ yellow, C+ red; alectorialic acid with satellite(s) by TLC.

#### Etymology

From *muskeg,* the Algonquin name for a blanket bog (USDA Forest Service 1990).

#### Habitat

In Alaska, *Fuscidea muskeg* occurs in muskeg (a blanket bog), a habitat closely associated with cool summers and abundant rain. In these habitats in GLBA it grows mainly on branches of *Pinus contorta* but also on *Picea sitchensis* (Bong.) Carrière, *Alnus* and *Vaccinium ovalifolium* Sm.

#### Notes

In the past (Fryday 2008*a*), *F. muskeg* has been mistaken for a corticolous form of *F. praeruptorum*, which is chemically identical. However, recent examination of richly fertile, corticolous material has revealed medianly constricted and rather short ascospores, 7–10 μm. In *F. praeruptorum* the ascospores are bean-shaped and slightly longer (9.5–12 μm), the areoles are smaller and more scattered than in *F. muskeg*, and the brownish or blackish hypothallus is more conspicuous. The overall colour of fresh material of *F. muskeg* is light green or green with a yellowish tinge on a pale brownish background, whereas *F. praeruptorum* has a distinct brown or black overall colour. In old herbarium material, both species are brown to pinkish. Within *Fuscidea*, the combination of medianly constricted ascospores and production of alectorialic acid distinguishes *F. muskeg* as a distinct species. *Fuscidea arboricola* Coppins & Tønsberg and *F. lightfootii* (Sm.) Coppins & P. James are also sorediate and have medianly constricted ascospores (see Gilbert *et al.* 2009), but those species are chemically different from *F. muskeg* in producing fumarprotocetraric acid and divaricatic acid, respectively. *Fuscidea lightfootii* was reported as new to North America by Aptroot (1996) but this was shown by Fryday (2008*a*) to be an error; it is not currently known to occur in North America.

*Fuscidea muskeg* is a widely distributed species in North America. Its phylogenetic position will be discussed by M. Zahradniková *et al.* (unpublished data).

#### Additional specimens examined

**USA:** *Alaska*: Glacier Bay National Park and Preserve, along and N of road from Gustavus to Bartlett Cove, near park entrance, 58°27.087′N, 135°47.284′W, 32 m, corticolous on *Pinus contorta* in glacial outwash plain, 2011, *Spribille* 36302, 36306 *& Fryday* (MSC, topotypes); *ibid*., 58°27.153′N, 135°46.604′W, corticolous on branches of *Pinus contorta* in muskeg, 2012, *Tønsberg* 41567 (MSC, topotype); *ibid*., 58°27.185′N, 135°46.643′W, corticolous on branches of *Pinus contorta* in muskeg [with *Equisetum*], *Tønsberg* 41577 (MSC, topotype); *ibid.*, 58.45115°N, 135.79013°W, corticolous on *Pinus contorta* branches, 2012, *Spribille* 38698 (MSC, topotype), *Svensson* 2561 (MSC); Bartlett Lake trailhead, 58°27.292′N, 135°52.240′W, 48 m, corticolous on *Vaccinium ovalifolium*, 2011, *Spribille* 36035 (MSC); E of Bartlett Cove along road, Crane Flats, just S of road along edge of meadows, 58.450110°N, 135.841521°W, 33 m, corticolous on *Alnus*, 2011, *Spribille* 36803 (MSC); ridge above Fern Harbor, at and E of the pond, 58°18.803′N, 136°27.635′W, 235 m, corticolous on branches/twigs of *Pinus contorta* in muskeg, 2012, *Tønsberg* 41771, 41773 (MSC); east shore of mouth of Glacier Bay, small point 5.5 km N of Point Gustavus and 2.8 km S of Bartlett Cove NPS compound, along seashore, 58.43005°N, 135.90211°W, 4 m, corticolous on *Picea sitchensis*, 2012, *Spribille* 39118 (MSC); *ibid.*, 3.8 km N of Point Gustavus and 4.4 km S of Bartlett Cove NPS compound, along seashore, 58.41458°N, 135.89868°W, 4 m, corticolous on *Picea sitchensis*, 2012, *Spribille* 39154 (MSC).

### Fuscopannaria dillmaniae T. Sprib. sp. nov.

MycoBank No.: MB 830099

Differing from all other *Fuscopannaria* species and *Moelleropsis nebulosa* in the thallus consisting entirely of corticate, isidioid granules 20–50 µm in diam., these usually branching, olivaceous brown; differing from *Leciophysma furfurascens* in the presence of a robust, black hypothallus.

Type: USA, Alaska, Hoonah-Angoon Census Area, just outside of boundaries of Glacier Bay National Park, NW of Gustavus, Tower Road, 58.46253°N, 135.77430°W, corticolous on *Alnus viridis* subsp. *crispa* (Aiton) Turrill, 33 m, 4 July 2012, *Spribille 38036*, *Tønsberg & Pérez-Ortega* (UPS—holotype; BG—isotype).

(Fig. 18)

**Fig. 18.**

*Fuscopannaria dillmaniae* and species that it may be confused with. A & B, *F. dillmaniae*, habitus of holotype, from which a DNA sequence was reported as *Santessoniella grisea* by Schneider *et al.* (2016); C, a specimen (*Tønsberg* 24918, BG) reported as *Santessoniella grisea* from Washington State, USA by Tønsberg & Henssen (1999), and later as a DNA voucher by Ekman *et al.* (2014), which appears to belong in the genus *Rockefellera*; D, a specimen (*Tønsberg* 32520) identified as *S. grisea* from Alaska (P.M. Jørgensen, unpublished data); E, isotype specimen of *Placynthium griseum* (W); F, holotype specimen of *Pannaria furfurascens* (H). Scales: A = 2 mm; B & C = 0.5 mm; D & F = 1 mm; E, mobile phone photograph, no scale bar available.

*Thallus* a sprawling crust, irregularly oval, 2–4.5 cm long and 1.5–2.5 cm wide, composed of minute isidioid growths on a black hypothallus, these cracking into areole-like patches when dry, the areoles 0.8–1 mm diam.; thalline isidioid outgrowths olivaceous brown, irregularly branched, not opuntioid, individual fingers 20–50 μm diam., consisting of tightly packed *Nostoc*-like cyanobacterial cells with fungal hyphae sheathed in a gelatinous cortex-like layer, a cellular cortex lacking.

*Ascomata* apothecia, round, single, 0.4–0.9 mm diam.; *disc* flat to strongly convex, reddish brown to dark red-brown, matt; *proper margin* soon receding, pale brown, matt. *Excipulum* to 62 μm wide laterally and 50–55 μm wide basally, composed of radiating, moniliform hyphae, up to 6 μm diam., lacking apparent pigments or crystals, but with thin pigment line occasionally separating excipular region from hymenium, I−. *Hymenium* 100–130 μm tall, hyaline, asci and surroundings I+ blue before KOH treatment, uppermost part of hymenium (‘epihymenium’) with mauve-brown pigments between the tips of the paraphyses, crystals lacking; *paraphyses* moniliform, weakly branched to branched and anastomosing, tips 2–3 μm wide, with KOH+ dirty green wall pigment. *Hypothecium* 150–200 μm thick, hazy hyaline, KOH+ slightly creamish, of tightly woven hyphae, lumina not >2 μm. *Asci* widely flask-shaped, *c*. 75 × 17 μm, 5–6 ascospores observed but probably 8 per ascus, lightly amyloid externally, with a strongly I+ apical tholus pierced by a distinct apical tube; *ascospores* simple, broadly ellipsoid, (11.5–)16.0–16.3(–22) × (7.5–)8.0–9.5(–11) μm, sometimes with apical end thickenings, lacking ornamented perispore (*n* = 18 from three specimens).

*Pycnidia* not observed.

#### Chemistry

All thallus spot tests negative; no substances detected by TLC.

#### Etymology

Named to honour the outstanding contributions of Karen Dillman in documenting the lichen biota of south-east Alaska.

#### Habitat

On the bark of *Alnus* and *Populus* in lowland temperate rainforests, so far known only from south-east Alaska. *Fuscopannaria dillmaniae* has been found at three sites in GLBA and on the nearby mainland near Juneau. It is probably more widespread in south-east Alaska than the few records currently suggest.

#### Notes

Minutely fruticose pannarioid cyanolichens have caused considerable confusion amongst taxonomists. Closely related species were described in the genera *Pannaria* Delise ex Bory, *Lemmopsis* (Vain.) Zahlbr. and *Placynthium* (Ach.) Gray, but Henssen (1997) united them into a new genus, *Santessoniella*, based mainly on thallus architecture. Molecular studies have since revealed the profuse, branched, finger-like thallus projections, characteristic of the morphologically defined *Santessoniella*, to be a product of convergent evolution of morphology that appears to have arisen at least three times in the *Pannariaceae* (Ekman *et al.* 2014; four if one counts the genus *Atrophysma*, described in this paper).

*Fuscopannaria dillmaniae* (Fig. 18A & B) is most similar to *Leciophysma furfurascens* (Nyl.) Gyeln. (Fig. 18F) in overall habit but differs in possessing a well-developed black hypothallus. Based on Henssen's work and subsequent keys to pannarioid lichens (Jørgensen 2000, 2005), the new species could also be assumed to belong to *Santessoniella grisea* (Hue) Henssen and this led one of us (TS, in Schneider *et al.* 2015) to incorrectly report an LSU rDNA sequence from the type of *F. dillmaniae* as *S. grisea*. *Santessoniella grisea* was reported from Washington State (Tønsberg & Henssen 1999) and later Alaska and Mexico (Jørgensen 2000); the Washington State material is also the basis for an ITS rDNA NCBI nucleotide database voucher (Ekman *et al.* 2014). Neither the Washington State specimen (Fig. 18C) nor another specimen subsequently collected from Alaska and named as *S. grisea* by P. M. Jørgensen (unpublished data) (Fig. 18D), closely resemble the isotype of *Placynthium griseum* Hue ≡ *Santessoniella grisea* (Hue) Henssen (W!, Fig. 18E). The DNA sequences of the Washington State specimen place it in the wider *Protopannaria* clade (Fig. 9), in an unsupported subclade with the eastern North American species *Rockefellera crossophylla* (Lendemer *et al*. 2017).

Another species with which *Fuscopannaria dillmaniae* could understandably be confused, based on the key and thallus dimensions in Jørgensen (2000), is *F. coralloidea* P. M. Jørg. Jørgensen reported for this species ‘thallus to 150 μm thick’ but did not provide measurements for individual coralloid lobes. Based on our study of the holotype (USA, California, *Sharnoff & Sharnoff* 1323.23, CANL–119402!), the thallus of *F. coralloidea* is 2–3 mm thick, and compared to *F. dillmaniae* has a much coarser thallus architecture, with fruticose branches an order of magnitude thicker (210–270 μm, flaring to 410 μm wide, vs 20–50 μm in *F. dillmaniae*). It grows on soil in California and Oregon.

Perhaps the *Fuscopannaria* species most similar to *F. dillmaniae* is *Moelleropsis nebulosa* (Hoffm.) Gyeln. *Moelleropsis* is clearly nested within *Fuscopannaria* (Fig. 9), a result that has been obtained before (see also Ekman *et al*. 2014) but is the older generic name. A proposal to conserve *Fuscopannaria* over *Moelleropsis* is pending (Jørgensen *et al*. 2013). *Fuscopannaria dillmaniae* possesses more structured, coralloid isidioid growths, compared to the loose granules that characterize *M. nebulosa*, and differs from the latter in its prominent black hypothallus.

#### Additional specimens examined

**USA:** *Alaska*: Glacier Bay National Park and Preserve, East Arm of Glacier Bay, mouth of unnamed creek E of Muir Point, 58.83642°N, 136.05313°W, corticolous on *Populus balsamifera*, with *Rostania occultata*, 8 m, 2012, *Spribille* 39251 (MSC); *ibid*., 58.83477°N, 136.05717°W, on *Alnus*, 2012, *Svensson* 2780 (UPS); W side of Glacier Bay, Shag Cove, 58°37.924′N, 136°19.715′W, corticolous on *Alnus*, sea level, 2011, *Spribille* 36401 (MSC); Juneau City and Borough, Montana Creek, 58°25.567′N, 134°37.979′W, on *Alnus*, 77 m, ix 2010, *Spribille* s. n., *Hampton-Miller & Taurer* (ALTA, BG, NY, UBC, UPS).

### Halecania athallina Fryday sp. nov.

MycoBank No.: MB 830100

Similar to *Halecania rhypodiza* (Nyl.) Coppins but with smaller ascospores and lacking any visible epilithic thallus.

Type: USA, Alaska, Hoonah-Angoon Census Area, Glacier Bay National Park, Excursion Ridge, 58.46349°N, 135.55807°W, 922 m, alpine heath with rock outcrops, alkaline argillite, 22 July 2012, *Fryday* 10114, *Spribille & Svensson* (MSC—holotype).

(Fig. 19)

**Fig. 19.**

*Halecania athallina* (holotype). A & B, habitus; C, section of apothecium; D, detail showing paraphyses; E–H, ascospores. Scales: A = 1 mm; B = 0.2 mm; C = 50 μm; D–H = 10 μm (scale bar provided only in H).

*Thallus* completely immersed in substratum. *Photobiont* chlorococcoid, cells 6‒10 μm diam.

*Apothecia* scattered, dark reddish brown, lecideine, (0.2‒)0.3‒0.4(‒6) mm diam.; *disc* flat to slightly convex when mature, with a persistent, slightly raised proper margin, 0.05 mm wide. *Excipulum* internally hyaline composed of narrow, branched and anastomosing hyphae *c.* 1.0 μm wide, outer 10 μm brown, with cortical cells 3‒4 μm wide with dark brown cap (similar to paraphyses). *Hymenium* 35‒40 μm tall; *epihymenium* brown, 5‒10 μm wide; *paraphyses* simple, 1‒1.5 μm wide, abruptly thickening at apex to 3‒4 μm with a brown cap. *Hypothecium c.* 30 μm tall, hyaline, composed of randomly orientated hyphae 2‒3 μm wide. *Asci Catillaria*-type, slightly clavate to clavate, 30‒35 × 12‒18 μm; *ascospores* hyaline, 1-septate, (9.5–)11.8 ± 1.9(–14.5) × (3.5–)4.7 ± 0.4(–5.5) μm, l/w ratio (2.2–)2.5 ± 0.4(–3.0), *n* = 10, with thin perispore.

*Conidiomata* not observed.

#### Chemistry

Apothecial section and adjacent thalline material KOH−, C−, PD−; unidentified substance at *R*_f_ classes A4, B4, C4 by TLC.

#### Etymology

A reference to the lack of thallus.

#### Habitat

On mildly basic argillitic rock in the alpine zone.

#### Notes

This is an inconspicuous but easily identified species on what appear to be almost uncolonized surfaces of argillitic rock (Fig. 19A). Attempts to amplify DNA were not successful.

The genus *Halecania* was erected by Mayrhofer (1987) for a group of six species previously included in *Lecania*, from which they differed in having *Catillaria*-type asci, distinctly capitate paraphyses and halonate ascospores. Additional species have subsequently been added (e.g. Coppins 1989*a*; Fryday & Coppins 1996; van den Boom & Elix 2005; van den Boom 2009) so that the genus now includes over 20 species (Index Fungorum 2019). Many species are saxicolous but the genus also includes corticolous, muscicolous and lichenicolous species.

The new species differs from all previously described species by the almost complete absence of an epilithic thallus. It most closely resembles *Halecania rhypodiza* but that species differs in having a distinct, dark brown, granular thallus and larger ascospores (12–15 × 4.5–6 μm; Fletcher & Coppins 2009).

#### Additional specimen examined

**USA:** *Alaska*: Hoonah-Angoon Census Area, Glacier Bay National Park, Excursion Ridge, 58.46349°N, 135.55807°W, 922 m, alpine heath with rock outcrops, alkaline argillite, 2012, *Fryday* 10130, *Spribille & Svensson* (MSC, sub *Lecidella patavina*).

### Hydropunctaria alaskana Thüs & Pérez-Ort. sp. nov.

MycoBank No.: MB 830101

Similar to *Hydropunctaria oceanica* Orange but differing in the shorter ascospores, distinctly fimbriate prothallus in some specimens, and nuclear ITS and mitochondrial SSU sequence data.

Type: USA, Alaska, Hoonah-Angoon Census Area, Glacier Bay National Park, Taylor Bay, 58.2549°N, 136.5675°W, 0–5 m, on metamorphic rocks (hornblende augen gneiss) beside creek with *Buellia coniops* and *Verrucaria aethiobola*, 9 August 2012, *Fryday* 10458 *& Spribille* (MSC—holotype).

(Fig. 20)

**Fig. 20.**

*Hydropunctaria alaskana*. A–C, habitus (A from holotype, B & C from isotype specimens); D, section of perithecium from holotype. Scales: A–C = 1 mm; D = 50 μm.

*Thallus* episubstratal, thin to moderately thick, sterile areas *c*. 40–115 μm thick (*n* = 7), cracks numerous, creating small areoles, continuous areas without cracks absent or restricted to the thallus margin, fertile areoles up to 400–600 μm diam., *c.* 2–3 times larger than sterile ones (25 largest measured on four thalli); surface of infertile areoles in the type material smooth, except on the thallus margin where jugae are protruding over the surrounding thallus and become visible as black dots; development of jugae is more variable in specimens from coastal rocks. Thallus surface brown to black-brown but occasionally rusty reddish tinged due to mineral particles deposited on the thallus, without any green component, not subgelatinous (remaining opaque when wet). *Pseudocortex* absent to max. 5–8 μm high, with faint brown pigmentation or hyaline, KOH−. *Prothallus* cream-coloured to clear white, distinctly fimbriate when well developed, but more often thin or absent. *Photobiont* cells mostly irregularly arranged, rarely in short vertical chains, (6.6–)7–8(–10.4) × (3.9–)6.1–6.7(–7) μm (*n* = 30), in section mostly ± cuboid (rarely orbicular), interspersed with some more elongated cells with length/width ratio of *c.* 2.1:1. *Medulla* brown-black in upper parts (‘black basal layer’), in parts fading to colourless at the base, fading more frequently observed under the perithecia, KOH−. Black protrusions (jugae) of up to 35 μm diam. frequent, in central parts of the thallus mostly emerging from black basal layer, some also disconnected from basal layer and initiating from a zone at approximately half the height of the thallus but usually not penetrating the pseudocortex, except on the flanks and in the direct vicinity (same areole) of the perithecia and on the edge of the thallus.

*Perithecia* forming flat projections, ±raised above the surrounding thallus (ratio perithecia/thallus height *c.* 1.5:1–4:1), crowded to widely-spaced and isolated (variation within a single thallus). *Involucrellum* conical, enveloping the exciple and merging laterally with black basal layer. *Exciple* 150–185(–230) μm wide (*n* = 5), with brown-black pigmentation from top to bottom. *Periphyses* 22–35 × 1–2 μm (*n* = 4). Interascal filaments quickly gelatinized in ontogeny. *Asci* 8-spored, 30–51 × 14–15.7 μm (*n* = 10). *Ascospores* (10.8–)12.2–15.0(–17.9) × (4.7–)5.3–7.1(–9.2) μm (*n* = 152), l/w ratio (1.6–)1.8–2.4(–3.4), without halonate perispore.

#### Chemistry

Pseudocortex pigment KOH−, brown pigment in basal layer KOH−. Hymenial gel I+ red, K/I+ blue. No metabolites detected by TLC.

#### Etymology

Named for its discovery in Alaska.

#### Habitat

The type collection is from a small stream close to the coastline, not inundated at the time of collection, where the species is locally frequent. The associated species (e.g. *Buellia coniops*) indicate at least the temporary influence of saline spray from the nearby seashore. Two other populations are known from the upper littoral zone on coastal rocks (e.g. with *Mastodia tessellata*) along the NW North American coast, on Mitkof Island (Alaska) and Vancouver Island (British Columbia).

#### Notes

**Fig. 21.**
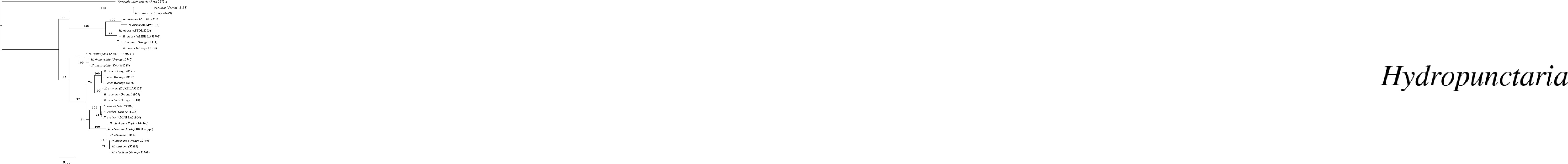
Majority-rule consensus tree of the genus *Hydropunctaria* (Eurotiomycetes) based on ITS and mtSSU loci, showing placement of the new species *H. alaskana* (bold) relative to previously known species. Values indicate percent bootstrap support. Further voucher information and GenBank Accession numbers are outlined in Table 1 and Supplementary Material Table S3 (available online).

This new species is morphologically most similar to *Hydropunctaria oceanica*, *H. aractina* (Wahlenb.) Orange, *H*. *maura* (Wahlenb.) C. Keller *et al.* and *H. orae* Orange. It differs in having, on average, shorter ascospores (12.5–15.0–18.0 μm in *H. oceanica*, 14.5–16.6–19.0 μm in *H. maura*, 13.0–16.1–19.5 μm in *H. orae*) and the presence of a fimbriate white prothallus in some specimens. The thallus is much thinner compared to the sun-exposed thalli of *H. maura* (40–115 μm vs 60–300 μm). From *H. orae* and *H. aractina* it also differs by the lack of green pigmentation in the cortex and brown-black colour in thallus surface view. There is an overlap with some forms of these species and identification of taxa from this genus in coastal areas (where occurrences of freshwater and salt-tolerant species can intermingle) can remain ambiguous if not supported by sequencing of the ITS region (Orange 2012). Two ITS sequences of the new species place *H. alaskana* apart from its morphologically most similar lookalike, *H. oceanica*, and in proximity to the freshwater taxon *H. scabra* (Vězda) C. Keller *et al*. (Fig. 21). It differs from *H. scabra*, most markedly, in its regularly cracked-areolate thallus with a generally smooth surface of the infertile areoles in the centre of the thallus, a non-subgelatinous structure with the photobiont cells in an irregular arrangement, on average slightly smaller ascospores and the absence of green colour components in the pseudocortex. Occasionally occurring cracks in *H. scabra* (particularly in older herbarium specimens) are thinner and usually clustered in the vicinity of fruiting bodies, but not throughout the entire thallus as in *H. alaskana* (and *H. oceanica*). In addition, the fimbriate prothallus as observed in parts of one of the sequenced topotypes of *H. alaskana* (Fig. 20B) has never been seen in *H. scabra* or any other freshwater *Hydropunctaria.* These differences are based on comparison with sequenced specimens of *H. scabra* from Europe and two North American vouchers from Alaska and Montana (*McCune* 32162, 32163 [OSC]) for which ITS sequencing failed. Among the coastal species, a white prothallus is often seen in *H. oceanica* but it is never fimbriate in that species and only *H. maura* is known to occasionally develop a scarcely fimbriate prothallus.

#### Additional specimens examined

**Canada:** *British Columbia*: Vancouver Island, W of Sooke, Flea Beach, 48°22′54.84″N, 123°55′55.92″W, on N-facing rocks at top of seashore, in slight shade, 2015, *A. Orange* 22768 (NMW, specimen not seen).—**USA:** *Alaska*: Hoonah-Angoon Census Area, Glacier Bay National Park, Taylor Bay, 58.2549°N, 136.5675°W, 0–5 m, *Fryday* 10455, 10456 (MSC—topotypes); Petersburg, South Mitkof Island, Sumner Strait, seashore of sedimentary rocks, 56°33′10″N, 132°38′41″W, 0–5 m, 2012, *Pérez-Ortega* 2042, *K. Dillman & Spribille* (MA-Lich); *ibid*., *Pérez-Ortega* 2045, *K. Dillman & Spribille* (MA-Lich).

### Lambiella aliphatica T. Sprib. & Resl sp. nov.

MycoBank No.: MB 830102

Similar to *Lambiella globulosa* but with fatty acids instead of stictic acid.

Type: USA, Alaska, Hoonah-Angoon Census Area, Glacier Bay National Park, Excursion Ridge, 58.46233°N, 135.55349°W, 907 m, saxicolous on argillite in alpine talus, 14 July 2012, *Spribille* 38388 (MSC—holotype).

(Fig. 22)

**Fig. 22.**

*Lambiella aliphatica* (holotype). A & B, habitus; C–E, SEM images of the surface of the apothecium, showing the umbo (C) and perforations in the surface of the umbo (D & E); F, section through apothecium; G, asci, in Lugol's solution; H, thallus cross-section. Scales: A = 1 mm; B = 0.2 mm; C = 100 μm; D = 20 μm; E, G & H = 10 μm; F = 50 μm.

*Thallus* crustose, forming patches to 3.5 cm across; areolate, the individual areoles 0.2–0.5 mm diam., bicoloured, with a dark grey edge and light grey centre; in section not stratified, differentiated cortex absent, but upper 10 μm of thallus pigmented grey; sterile hyphae non-amyloid. *Photobiont* chlorococcoid, roundish, cells 5–10 μm diam.

*Ascomata* apothecia, rounded to angular or even forming ‘U’ or ‘C’ shapes, (0.25–)0.3–0.5(–0.7) mm diam., single or forming groups of 2–3, or in tubercles to 0.8 mm diam., with a distinct umbo; *disc* deeply concave, black, matt to slightly lustrous, with a prominent black margin, the umbo with a sieve-like pattern visible in SEM (Fig. 22D & E). *Excipulum* 55–95 μm wide laterally, brown internally, outer 20–40 μm jet black (‘carbonized’), composed of hyphae to 4 μm diam., swelling to 6 μm in KOH, non-amyloid, excipulum but not epihymenium almost covered by bacterial colonies (stained in LCB). *Hymenium* 65–90 μm tall, pale yellow-brown to hyaline, strongly amyloid (I+ wine red, I+ blue after KOH), full of lipid bodies in lower half, in upper part pigmented brown-olivaceous; *paraphyses* branched and anastomosing, with kinks, separating in KOH, 1.8–2 μm wide medianly, to 4 μm wide apically, with internal terminal brown pigmentation. *Hypothecium* 70–90 μm tall, pale brown-yellow to brown, composed of heavily pigmented, thick-walled hyphae to 3 μm diam. *Asci c*. 42 × 15–18 μm, 8-spored; *ascospores* simple, ovoid or broadly ellipsoid, (6.5–)8.2–9.3(–12) × (4.5–)5.5–6.3(–7) μm (*n* = 28 over three specimens).

*Conidiomata* not observed.

#### Chemistry

All thallus spot tests negative; two unidentified fatty acids with *R*_f_ classes A2 and A3, B4, C3 and C4 by TLC.

#### Etymology

A reference to the presence of aliphatic (fatty) acids that characterize this species.

#### Habitat

On argillite rock on alpine ridgeline and in talus.

#### Notes

*Lambiella aliphatica* (Fig. 22) is the first member of the genus to be described that contains primarily fatty acids in the thallus. The genus *Lambiella* Hertel was recently treated in some detail, as a split from *Rimularia* Nyl., in a phylogenetic study by Resl *et al.* (2015). DNA sequences from *L. aliphatica* (first published by Resl *et al.* 2018) clearly place it in the *L. impavida* group as sister to a clade that includes *L. globulosa* (Coppins) M. Westb. & Resl and *L. gyrizans* (Nyl.) M. Westb. & Resl (Fig. 8). It is similar to *L. globulosa*, a species described from Scotland (Coppins & Kantvilas 2001), especially on account of its near-globose ascospores, but it differs from that species and *L. gyrizans* in secondary metabolite chemistry as both possess thalli that are K+ yellow to orange and contain the stictic acid complex (Hertel & Rambold 1990; Coppins & Kantvilas 2001). It is so far known only from the type locality but is an inconspicuous species and is likely to occur elsewhere in the mountains around the Gulf of Alaska, though it may be restricted to the few regions with argillite or slightly basic rocks.

#### Additional specimens examined

**USA:** *Alaska*: Hoonah-Angoon Census Area, Glacier Bay National Park, Excursion Ridge, 58.46233°N, 135.55349°W, 907 m, saxicolous on argillite in alpine talus, 2012, *Spribille* 38392 (sub *Amygdalaria subdissentiens*), 38395-B (MSC—topotypes).

### Lecania hydrophobica T. Sprib. & Fryday sp. nov.

MycoBank No.: MB 830103

Similar to *Lecania baeomma* but richly fertile and lacking soredia/soralia.

Type: USA, Alaska, Hoonah-Angoon Census Area, Glacier Bay National Park, Cross Sound, Taylor Bay at ‘campsite cove’, 58.25467°N, 136.56860°W, saxicolous on vertical shale outcrop W of camp, 9 m, 8 August 2012, *Spribille* 39680 & *Fryday* (MSC—holotype; NY—isotype).

(Fig. 23)

**Fig. 23.**
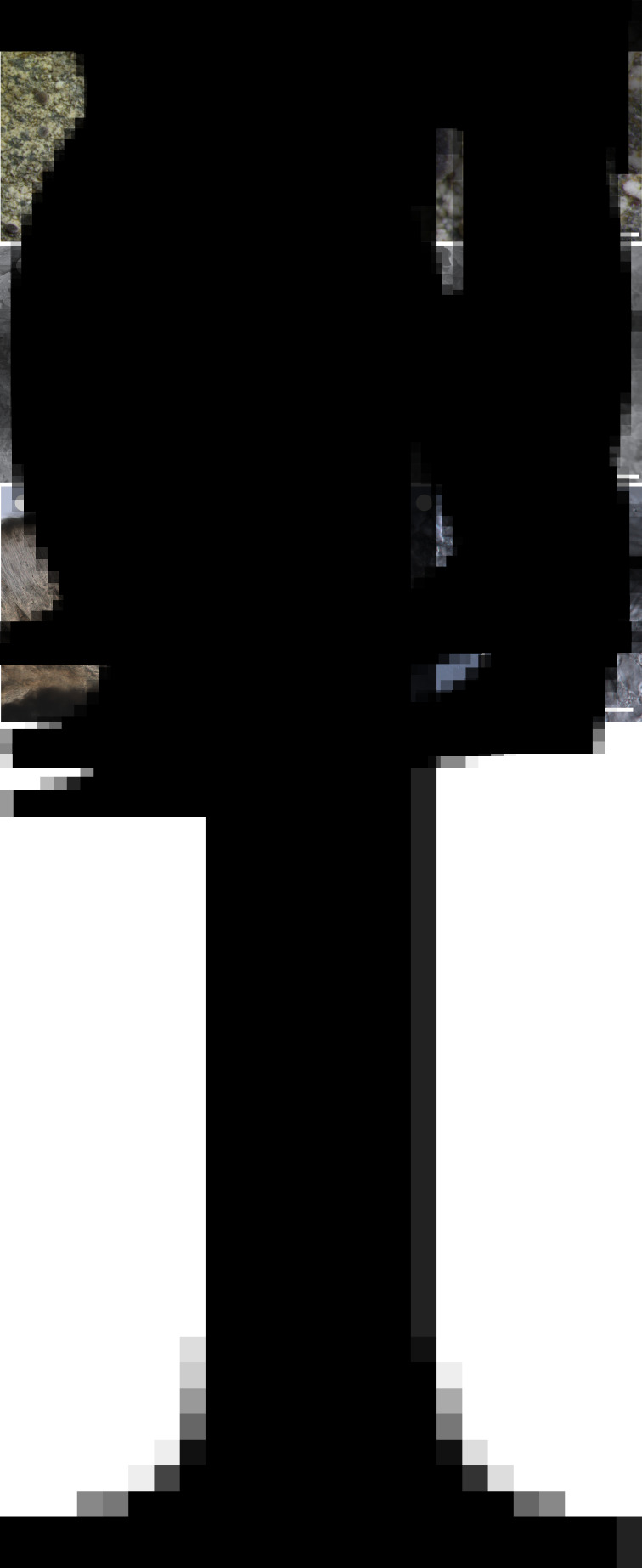
*Lecania hydrophobica* (holotype). A & B, habitus; C & D, SEM images of thallus showing (C) film-like covering of medullary hyphae and (D) hyphal coating of thin (<100 nm in diam.) spaghetti-like (wax?) fibrils; E, section through apothecium; F–I, ascospores. Scales: A = 1 mm; B = 0.2 mm; C, F–I = 10 μm; D = 5 μm; E = 20 μm.

*Thallus* crustose, composed of small convex areoles, ±bullate, becoming almost granular, creamish white to ochre-yellowish, hydrophobic; *individual areoles* 0.15–0.45 mm diam., thallus scarcely stratified, internally with wefts of hyphae, these covered by a thin, biofilm-like cortex (Fig. 23C); *sterile hyphae* appearing ornamented or papillate at × 1000 magnification, this probably owing to thick layers of spaghetti-like filaments <100 nm in diam. throughout the internal thallus (Fig. 23D), that resemble certain plant waxes, for example in Barthlott *et al.* (1998: Fig. 19A). *Photobiont* chlorococcoid, cells 6–17 μm diam.

*Ascomata* apothecia, round, well-spaced, single, flat to convex, becoming tuberculate, (0.5–)0.75–0.87(–1.6) mm diam.; *disc* reddish brown to greyish brown to piebald, matt, apparently epruinose or with a light appearance of pruina, sometimes heavily pruinose; *proper margin* very prominent in youngest ascomata, the disc emerging through a ‘doughnut hole’, ‘pruinose’ on account of heavy layering of wax-like filaments, margin becoming thin, brown, giving a ‘lecanorine’ impression, eventually becoming excluded with age. *Excipulum* 60–100 μm wide laterally, composed of radiating thick-walled hyphae to 7 μm diam., not amyloid, full of crystals as viewed under polarized light; *hymenium* 60–120 μm tall, hyaline to hazy yellowish, IKI+ blue both before and after treatment with KOH; *paraphyses* strongly conglutinated, even after treatment with KOH, not widened apically; *epihymenium* full of POL+ crystals, these golden brown in transmitted light. *Hypothecium* hyaline or pale yellowish brown, 50–150 μm tall, full of grana, apparently full of old asci (Fig. 23E) as if apothecium builds upwards upon older hymenia; not amyloid, containing interspersed POL+ crystals, some of the hyphae thin, <2 μm diam., containing a very narrow cytoplasm. *Asci Bacidia*-type, 8-spored, difficult to visualize due to hamathecial conglutination, many containing yellowish grana, *c*. 50 × 10 μm; *ascospores* 1-septate, broadly ellipsoid, (10.5–)12.4–13.8(–18) × (3.5–)4.3–4.8(–5.5) μm (*n* = 35, three specimens used).

#### Chemistry

Thallus all spot tests negative, except faintly UV+ yellowish; atranorin and gangaleoidin detected by TLC.

#### Etymology

Named for its highly hydrophobic properties, perhaps on account of its unusual wax-like filament structures.

#### Habitat

Saxicolous on rain-sheltered underhangs of shale rocks near sea level.

#### Notes

This species was first reported for North America from British Columbia by Brodo (1976) as *Catillaria biformigera* (Leight.) P. James ( = *Tylothallia biformigera* (Leight.) P. James & H. Kilias) and distributed by him as *Lichenes Canadenses Exsiccati* 93. In his notes on that exsiccatae, Brodo (1976) discussed his collection in relation to *Catillaria baeomma* (Nyl.) Zahlbr. (=*Lecania baeomma* (Nyl.) P. James & J. R. Laundon) and concluded that, because the original material of that species examined by him lacked soralia, James (1965) was correct in including the species in the synonymy of *C. biformigera*. However, the current concept of *L. baeomma* is of a species with diffuse blue-grey soralia (Reese Næsborg 2008; Fletcher *et al.* 2009*a*) and the situation is further complicated by the fact that James (1970) originally named this taxon *Lecania rupicola* (Nyl.) P. James and only later, without explanation, synonymized that name with the earlier *L. baeomma* (Hawksworth *et al*. 1980).

Our phylogeny confirmed the close relationship of our collection with *L. baeomma* (Fig. 10). To ascertain whether our species was synonymous with *L. baeomma* s. str., which according to Brodo (1976) was also esorediate, we obtained from H-Nyl and BM the type material and other original collections of *Lecanora baeomma* Nyl. and its synonyms (viz. *Lecanora caesia* W. Johnson, *L. caesiolepra* Nyl., *L. glaucocarnea* Nyl. and *Lecidea rupicola* Nyl.), all of which, apart from *L. caesia*, were collected by Larbalestier from Connemara in the west of Ireland. Although all these collections were small, it was immediately apparent that *L. caesia, L. caesiolepra* and *L. rupicola* were synonymous with the current concept of *L. baeomma* as a species with diffuse, blue-grey soredia and were not conspecific with our species. Furthermore, close examination of the type collection of *L. baeomma* showed that it was also sorediate, but the soralia were small (*c*. 0.3 mm diam.) and white-coloured and easily overlooked. Clearly our species was also not conspecific with the type of *L. baeomma.* The apothecia of all four of these taxa had a thick, non-corticate thalline margin that persisted into mature apothecia, unlike that of our new species in which only young apothecia have a thalline margin and mature apothecia are apparently lecideine. This left *L. glaucocarnea* as the only possible species that might represent a previously published name for our species but, although the type collection lacked soredia, it was otherwise quite different, having a thick, areolate thallus and apothecia that lacked any sign of a thalline margin, even when young. To confirm these conclusions, we compared apothecial sections of our new species with those of representative specimens of *L. baeomma*, *L. glaucocarnea* and *L. rupicola.* Although all were clearly similar and all had inspersed crystals, they differed in the position, size and solubility of these crystals; specifically, whereas all sections had crystals in the subhymenium and exciple, the hymenium of our new species contained large crystals that did not dissolve in K. The hymenium of the other three taxa, by contrast, either lacked crystals or contained small crystals that dissolved in K.

Although not required to justify describing our collection as a new species, we were curious whether *L. baeomma* and *L. rupicola* were indeed synonyms. Two collections helped us decide this. The first was a collection from H labelled ‘Larbalestier's Lichen Herbarium (*Exsiccatum britannicum*) # 26 *Lecanora baeomma*’ that had diffuse blue soralia, indicating that Larbalestier thought *L. rupicola* and *L. baeomma* were conspecific. The second collection was a specimen in BM (BM000975337) that had both diffuse, blue-grey and small, white soralia on the same specimen, clearly indicating that only one species was involved. The only remaining issue was the identity of *L. glaucocarnea*, which appeared quite different from *L. baeomma* and possibly represents a distinct species, but the resolution of this was beyond the scope of the current study.

Reese Næsborg (2008) indicated that ‘the proper taxonomic placement of *Lecania baeomma* remains uncertain’. Our phylogenetic reconstruction with only one locus (mtSSU) suggests, as did the original work of Reese Næsborg, a close relationship to *Ramalina* Ach. (Fig. 10); it is certainly not a *Lecania* in the sense of the type of that genus (*L. fuscella* (Schaer.) A. Massal.). Exploring the proper placement with more loci is not possible at this time.

*Lecania hydrophobica* was collected once in GLBA near the outer coast where it was abundant on sheltered rock underhangs. It is otherwise known from collections outside and to the south of GLBA by Mary Stensvold (née Muller) on southern Baranof Island and by Irwin Brodo from British Columbia.

#### Additional specimens examined

**Canada:** *British Columbia*: Haida Gwaii (= ‘Queen Charlotte Islands’), Graham Island 1967, *Brodo* 10165, *Lichens Canadenses Exsiccati* 93 (MSC, as *Catillaria biformigera* (Leight.) P. James).—**USA:** *Alaska*: Sitka Borough, Baranof Island, west coast, point of land W of Yamani Islets, N side at the mouth of Neker Bay, 56.67°N, 135.20°W, E-facing cliff of Sitka graywacke and adjacent area, 1992, *M. C. Muller* 5716 (TNFS-3117); *ibid.*, narrow marine passageway connecting Redfish Bay and Big Branch Bay, 56.33°N, 134.86°W, rocky exposure just above high tide line, 1992, *M. C. Muller* 5721 (TNFS-3119).

### Lecanora viridipruinosa M. Svenss. & T. Sprib. sp. nov.

MycoBank No.: MB 830104

Distinguished by the thallus of scattered, ±adnate areoles, the lecideine, greenish pruinose (C−, KC−) apothecia and by the thallus containing atranorin and zeorin.

Type: USA, Alaska, Hoonah-Angoon Census Area, Glacier Bay National Park, Excursion Ridge, 58.46349°N, 135.55807°W, 920 m, alpine heath with rock outcrops, on exposed argillite rock, 22 July 2012, *Svensson* 2626, *Fryday & Spribille* (MSC—holotype)

(Fig. 24)

**Fig. 24.**

A, *Lecanora viridipruinosa*, habitus of *Spribille* 38419; B, an undescribed *Lecanora* from the *L. formosa* group found at the type locality of *L. viridipruinosa* (*Spribille* 38425). Both specimens are represented in the phylogenetic tree in Fig. 10. Scales: A = 2 mm; B = 1 mm.

*Thallus* of dispersed, discrete, ±adnate areoles, 0.5–1.5 mm diam., dull white; *hypothallus* not apparent, but edges of areoles sometimes encrusted in a black mat of cyanobacteria. *Photobiont* chlorococcoid, cells 8–15 μm diam.

*Apothecia* semi-immersed to sessile, scattered on areoles, (0.35–)0.5–0.8(–1.5) mm diam., lecideine, ±round to somewhat irregular, at first with a flat, black disc and a thin, rarely flexuose margin concolorous with the disc, but soon becoming convex; *disc* usually with a faint, greenish pruina (C−, KOH+ dissolves, KC−, N−). *Excipulum* 80−120 μm wide, greenish black, without photobiont cells. *Hymenium* 45–70 μm high; *epihymenium* green-aeruginose, 5–15 μm high; *paraphyses* numerous, branched and anastomosing, 1.0–1.5 μm thick, not or slightly thickened (to 2(–4) μm) at the apex, with a green pigment cap. *Hypothecium* pale though sometimes discoloured orange-brown by mineral particles, occasionally with a small number of photobiont cells basally. *Ascus Lecanora*-type, 8-spored, broadly clavate, 30–50 × (9–)12–15 μm; *ascospores* simple, hyaline, thick-walled (to 1 μm), broadly ellipsoid, (7–)10(–14) × (4–)5(–7) μm (*n* = 22).

*Conidiomata* not observed.

#### Chemistry

Thallus C−, KOH+ yellow, PD−, UV−; apothecial section C−, KOH−, HNO_3_+ red; atranorin and zeorin by TLC.

#### Etymology

A reference to the greenish pruina on the apothecial discs.

#### Habitat

On argillite rocks in the alpine zone, so far known only from the type locality.

#### Notes

*Lecanora viridipruinosa* belongs to a group of black-fruited, saxicolous species that are probably closely related to each other and the genus *Palicella* Rodr. Flakus & Printzen (Fig. 10), but not to *Lecanora* Ach. s. str. They are still generally retained in *Lecanora* pending a systematic revision of lecanoroid genera. Several of these, such as *L. viridipruinosa*, contain atranorin and zeorin: *Lecanora formosa* (Bagl. & Carestia) Knoph & Leuckert has a more well-developed thallus of convex ± bullate areoles, greyish-pruinose apothecia and contains ± psoromic acid in addition to atranorin and zeorin (Edwards *et al.* 2009). *Lecanora sulphurea* (Hoffm.) Ach. has a more well-developed, yellowish thallus, larger (to 2.5 mm) apothecia with grey pruina and contains gangaleodin, α-collatolic acid and usnic acid in addition to atranorin and zeorin (Edwards *et al.* 2009). *Lecanora griseofulva* Elix & Øvstedal has epruinose apothecia and contains griseofulvin as a major substance in addition to atranorin and zeorin (Elix & Øvstedal 2004). Species with other chemistries might also belong here. *Lecanora atromarginata* (H. Magn.) Hertel & Rambold has a more well-developed, yellowish thallus with a prominent, pale prothallus, larger (to 2.5 mm), epruinose apothecia and contains usnic and stictic acids (Edwards *et al.* 2009). *Lecanora atrosulphurea* (Wahlenb.) Ach. has larger apothecia (to 2.5 mm) with both aeruginose and brown pigments and contains usnic acid, ±norstictic acid, and ±xanthones (Edwards *et al.* 2009). *Lecanora caesiosulphurea* Vain. has a sulphur yellow thallus, an HNO_3_ violet epihymenium, and bluish or lead-coloured pruina that react KC+ red (Thomson 1997). *Lecanora marginata* (Schaer.) Hertel & Rambold has a better developed, yellowish thallus (except whitish in subsp. *elata* (Schaer.) Clauzade & Cl. Roux), epruinose apothecia and contains atranorin and ±usnic acid (Edwards *et al.* 2009). *Lecanora scrobiculata* (Th. Fr.) Elix & Øvstedal has epruinose apothecia and contains psoromic acid in addition to atranorin (Elix & Øvstedal 2004). *Lecanora viridiatra* (Stehn.) Zahlbr. has a yellowish green thallus and contains usnic acid (Aptroot *et al.* 2009 as *Lecidea luteoatra* Nyl.). *Lecidella viridans* (Flot.) Körb. could be confused with *L*. *viridipruinosa* owing to the black, greenish pruinose apothecia and similar-sized ascospores, but it contains arthothelin, thiophanic acid and 4,5-dichloro-norlichexanthone (C+ orange, UV+ orange; Fletcher *et al.* 2009*b*).

Material from GLBA includes two thallus forms, one with thick, roundish areoles (Fig. 24A) represented by *Spribille* 38419 and *Fryday* 10130, and another with a thin, non-areolate thallus (Fig. 24B) exemplified by *Spribille* 38425. These initially appeared to be extreme ends of thallus variation within a single population, and grew intermixed on the same mountain top, but DNA data, from one specimen each, suggest they are different species, both from the *Lecanora formosa* group. The only overlapping locus obtained, mtSSU, differs at 10 shared positions for the amplicons obtained for *L. viridipruinosa* (*Fryday* 10130, voucher T1806) and the additional species (*Spribille* 38425, voucher T1181), as well as *L. viridipruinosa* lacking a 10 bp insertion present in the latter. The second species contains atranorin but lacks zeorin, and may be undescribed. It is probably conspecific with a collection from the Yukon (*Spribille* 28362, GZU; T1019 in Fig. 10).

#### Additional specimens examined

**USA:** *Alaska*: Hoonah-Angoon Census Area, Glacier Bay National Park, Excursion Ridge, sharp point on eastern side of ridge, 58.46304°N, 135.55062°W, 936 m, saxicolous on argillite, 2012, *Spribille* 38419 (MSC); *ibid.*, 58.46349°N, 135.55807°W, 920 m, alpine heath with rock outcrops, on exposed rock, 2012, *Svensson* 2603, *Fryday & Spribille* (MSC); *ibid.*, *Svensson* 2604, *Fryday & Spribille* (MSC); *ibid*., *Svensson* 2638, *Fryday, & Spribille* (MSC); *ibid*., *Spribille* 38828 (MSC, sub *Lecidea lapicida*); *ibid.*, *Fryday* 10130 (MSC, sub *Halecania athallina*).

#### Other specimens examined (*Lecanora* aff. *viridipruinosa*)

**Canada:** *Yukon Territory*: Mt Martin, *Spribille* 28362 (UPS).—**USA:** *Alaska*: Hoonah-Angoon Census Area, Glacier Bay National Park, Excursion Ridge, sharp point on eastern side of ridge, 58.46304°N, 135.55062°W, 936 m, saxicolous on argillite, 2012, *Spribille* 38425 (MSC).

### Lecidea griseomarginata Fryday sp. nov.

MycoBank No.: MB 830109

Similar to *Lecidea lapicida* but separated from that species and all other species of the genus by the broad, grey proper margin.

Type: USA, Alaska, Hoonah-Angoon Census Area, Glacier Bay National Park, Ptarmigan Creek, along shore N of creek, 58.8890°N, 136.8970°W, 0‒10 m, shoreline granitic rock, 15 July 2012, *Fryday* 9938, *Spribille & Svensson* (MSC—holotype).

(Fig. 25)

**Fig. 25.**

*Lecidea griseomarginata* (*Fryday* 9937). A, habitus; B, apothecial section; C, section through hymenium, with asci and ascospores. Scales: A = 0.5 mm; B = 100 μm; C = 10 μm.

*Thallus* effuse, mostly immersed in the substratum, visible only as a black hypothallus between the rock granules and rarely as small patches of a thin grey, cracked-areolate thallus; *medulla* I+ blue. *Photobiont* chlorococcoid, cells 9‒15 μm diam.

*Apothecia* scattered, 0.6‒1.0 mm diam., lecideine, ±orbicular with a flat, black disc and a wide grey margin 0.5 mm across that is barely raised above the level of the disc. *Excipulum* internally hyaline with mottled brown patches composed of radiating hyphae with cells 4‒5 μm wide, outer 35‒50 μm similar but with additional blue-black (HNO_3_+ red) pigment; extending below the hypothecium where it is composed of narrower, randomly orientated hyphae, this structure sometimes extending into the lateral section. *Hymenium* 75‒80 μm high; *epihymenium* bright aeruginose, 10‒12 μm thick; *paraphyses* simple, 1.5‒2.0 μm thick, widening slightly at the apex up to 3 μm, with a blue-black (HNO_3_+ red) cap, septate; lax in KOH. *Hypothecium* brown, up to 175 μm thick at centre of apothecium narrowing to nothing at the excipulum. *Ascus Lecidea*-type, slightly clavate, 40‒50 × 12‒15 μm; *ascospores* simple hyaline, broadly ellipsoid, (10.5–)12.6 ± 1.3(–14.5) × (5.5–)6.5 ± 0.7(–8.0) μm, l/w ratio (1.6–)1.9 ± 0.2(–2.3), *n* = 18.

*Conidiomata* not observed.

#### Etymology

The name refers to the wide, grey proper margin.

#### Habitat

On recently deglaciated granitic rock at sea level.

#### Chemistry

Apothecial section C−, KOH+ yellow solution; stictic acid by TLC.

#### Notes

Known only from the type locality, where it occurs near the shore but near the end of a sheltered fjord and probably little affected by maritime influences. *Lecidea griseomarginata* belongs to the *Lecidea lapicida* group, which includes species of *Lecidea* s. str. with relatively broad ascospores (>5 μm) and a thallus with an amyloid medulla but lacking an epinecral layer (i.e. not atrobrunnea-type). Hertel (1995) recognized three species in the group: *L. ecrustacea* (Anzi ex Arnold) Arnold, which lacks an epilithic thallus, and *L. lapicida* (Ach.) Ach. and *L. swartzoidea* Nyl. with an epilithic thallus. He separated the last two species by hypothecium colour; hyaline to pale brown in *L. lapicida* but dark brown in *L. swartzoidea*. He further recognized two varieties of *L. lapicida*: var. *lapicida* (stictic acid or no substances) and var. *pantherina* (DC.) Ach. (norstictic acid). However, two morphologically distinct entities exist with an epilithic thallus containing norstictic acid: a norstictic acid-containing variety of *L. lapicida*, and a distinct species with a thick, white thallus and ±immersed apothecia that is usually referred to as *L. lactea* Flörke ex Schaer. (e.g. Aptroot *et al*. 2009), sequences of which are published here (Table 1; from a KLGO specimen). The taxonomy of the group is highly confused and much in need of a thorough modern revision, along with the rest of *Lecidea* s. str.

*Lecidea griseomarginata* differs from all the above-mentioned taxa, and other species of *Lecidea* s. str., most noticeably in its thick, grey proper margin (Fig. 25A). The lack of an epilithic thallus further distinguishes it from all the above species except *L. ecrustacea*, from which it differs in its distinctive exciple structure and by lacking norstictic acid. The new species is just one of the distinct morphotypes in the *L. lapicida* group that we are aware of in the North American Arctic.

We obtained fungal DNA sequences of *Lecidea griseomarginata* from three loci, namely 28S, mtSSU and *Mcm7*. However, so few other *Lecidea* species have published sequences from these loci that building a tree from the available data would be uninformative. We publish the sequences here for future reference (Table 1).

#### Additional specimen examined

**USA:** *Alaska*: Hoonah-Angoon Census Area, Glacier Bay National Park, Ptarmigan Creek, along shore N of creek, 58.8890°N, 136.8970°W, 0‒10 m, shoreline granitic rock, 2012, *Fryday* 9937, *Spribille & Svensson* (MSC—topotype; sub *Rhizocarpon lecanorinum*).

### Lecidea streveleri T. Sprib. sp. nov.

MycoBank No.: MB 830110

Thallus whitish with dark chocolate brown, flattened apothecia with ascospores 8–12(–18) × 3–4(–5) μm. Differing from *Lecidea albofuscescens* Nyl. and *L. lesdainii* Zahlbr. in the narrower ascospores, lack of ascospore ornamentation, flattened apothecia and thin, filmy thallus.

Type: USA, Alaska, Hoonah-Angoon Census Area, Glacier Bay National Park, steep slopes above unnamed lake in basin on west side of Dundas Bay, 58.34713°N, 136.39937°W, 180 m, corticolous on *Alnus* along mountain stream, 25 July 2012, *Spribille* 39030 (US—holotype; MSC—isotype).

(Figs 26 & 27)

**Fig. 26.**

*Lecidea streveleri* (and putatively related species). A & B, *Lecidea streveleri* (holotype); C, habitus, *Lecidea albofuscescens*, (holotype, H-Nyl-20725); D, habitus, *Lecidea lesdainii* (holotype of *Helocarpon corticola*, LI-271019); E–G, SEM images of ascospores of (E) *L. streveleri* (from holotype), (F) *L. lesdainii* (from holotype of *Helocarpon corticola*) and (G) *L. albofuscescens* (from *Spribille* 36527). Scales: A = 1 mm; B = 200 μm; C & D = 2 mm; E & G = 2 μm; F = 5 μm.

**Fig. 27.**

*Lecidea streveleri* (anatomical details of apothecium). A, section of apothecium (*Spribille* 39197); B, z-stack of apothecial section in lactophenol cotton blue, showing putative bacteria in epihymenium; white line indicates break between vertical and perpendicular surfaces (*Spribille* 39707); C & D, hypothecium in brightfield (C) and polarized (D) light showing upwards contortion and integration of outermost layers of *Alnus* periderm into the apothecium (arrowed) (*Spribille* 39707); E–G, asci, in Lugol's solution (E & F, holotype; G, *Brodo* 11042 [GZU]); H, ascospores (*Spribille* 39197). Scales: A = 100 μm; B, E–H = 10 μm; C & D = 50 μm.

*Thallus* smooth, rimose, not clearly areolate but algal cells aggregated in clumps beneath the thallus surface, giving a mottled appearance when moist; thin, up to 100 μm in section, greyish white to greenish white; forming patches 0.5–4 cm diam.; stratification weak, but cortical polysaccharide layer 10–15 μm thick. *Photobiont* chlorococcoid, cells 7–9 μm diam.

*Ascomata* apothecia, round, (0.25–)0.5–0.67(–0.9) mm, single or in small groups, weakly convex or appearing nearly flattened, sometimes ±tuberculate, medium brown, matt to weakly shiny, strictly epruinose; *proper margin* prominent, not excluded when old, shiny, black to brown or rarely pigment-deficient, swelling and translucent when wet. *Exciple* 40–60 μm wide laterally, 30–60 μm wide basally, streaked reddish to medium brown internally, lacking visible crystals in polarized light, I−, composed of radiating hyphae with apparently papillate walls, hyphae embedded in a bacteria-containing gelatinous layer that can extend for 6–10 μm beyond the ends of the hyphal tips. *Hymenium* pale brown, brownish streaked to almost hyaline, 48–60 μm tall, I+ deep blue, sometimes with apparent lipid accumulations in lower hymenium; *epihymenium* not or weakly developed, structures similar to bacilli visible in LCB staining, but gelatinous, epipsammoid layer and crystals lacking (POL−) (Fig. 27B), pigment accumulations brownish, KOH−; *paraphyses* simple or weakly branched, 4–5 μm diam. apically, with wall to 1.5 μm, mostly not capitate although a few capitate hyphae seen (belonging to *L. streveleri*?). *Hypothecium* dark reddish brown, 110–200(–250) μm tall, I−, lacking visible crystals in polarized light, composed of strikingly thick-walled hyphae 7–9 μm diam., with lumen *c*. 1 μm diam., anchored directly in the substrate phloem (Figs 27C & D). *Asci* clavate (Figs 27E–G), *Bacidia*-type, 34–35 × 6–8 μm, with 8 ascospores; *ascospores* narrowly ellipsoid, (7.0–)9.5–10.5(–13) × (2.7–)3.0–3.5(–4.0) μm (*n* = 52), not ornamented (Figs 26E & 27H).

#### Chemistry

Thallus all spot tests negative; no substances detected by TLC.

#### Etymology

Named in honour of Dr Gregory P. Streveler, an extraordinary naturalist and polymath, and author of numerous scientific papers, who has dedicated much of his life to understanding the natural history of Glacier Bay.

#### Habitat

On bark of *Alnus* and *Populus balsamifera*. In addition to its occurrence in Glacier Bay, an exsiccatae from Haida Gwaii, British Columbia, also from *Alnus*, has been widely distributed under the name *Lecidea albofuscescens* (Brodo 1971).

#### Notes

*Lecidea streveleri* is similar to *L. albofuscescens* Nyl., a widespread species of boreal-montane forests of the Northern Hemisphere, and *L. lesdainii* Zahlbr., a species described from Macaronesia and disjunct along the North American Pacific Coast (Breuss 1990, 2001). It differs from both species, however, in its consistently narrower ascospores (3.3 ± 0.36 μm (*n* = 52) in *L. streveleri* vs 5.0 ± 0.64 μm (*n* = 71) in *L. albofuscescens*, *P* = <0.001; ascospores are even wider in *L. lesdainii*, see Breuss (1990)). *Lecidea streveleri* can further be distinguished from both of these species by the lack of ascospore ornamentation (Fig. 26E); in *L. albofuscescens* and *L. lesdainii* the ascospores are almost always clearly warted (compare Figs 27F & G), a feature that can usually also be observed as an irregular ascospore surface in microscopic water mounts. In addition, *L. streveleri* forms smooth, rimose, whitish thalli, whereas *L. albofuscescens* and *L. lesdainii* both form minutely granular to granular-areolate thalli.

*Lecidea streveleri* is superficially similar to other crustose lichens that possess apothecia with dark, brownish hypothecia and one-celled ascospores. One group of species that it could, in theory, be confused with is the so-called ‘*Lecidea plebeja* group’ (Palice *et al.* 2018), likely including the eastern North American *Biatora peliaspis* Tuck. However, members of this group possess apothecial discs with epithecial crystals that are soluble in KOH, giving the apothecial disc a grainy appearance, unlike in *L. streveleri* and other members of the *L. albofuscescens* group which lack such crystals and possess glossy apothecia. Probably also related to both *L. albofuscescens* and the ‘*L. plebeja* group’ are members of the ‘*Lecidea malmeana* group’, also discussed by Palice *et al.* (2018), which differ in possessing polysporous asci (in low multiples of eight, ‘plusiosporic’ *sensu* Palice *et al.* (2018)), and thus are less likely to be confused with *L. streveleri*.

Attempts to amplify DNA from *L. streveleri* for this study were unfortunately not successful. However, we were able to obtain DNA sequences from *L. albofuscescens*, including three specimens from GLBA, albeit from only two loci. Analyzed within the broader context of the order *Lecanorales*, these sequences resolve within the family *Malmideaceae*, in the vicinity of genera such as *Cheiromycina* B. Sutton, *Malmidea* Kalb *et al.*, *Puttea* S. Stenroos & Huhtinen, and *Savoronala* Ertz *et al*. (Fig. 10), as well as a specimen of *Lecidea malmeana* from GLBA. However, the genus *Kalbionora*, which has been postulated to belong to *Malmideaceae* (Sodamuk *et al.* 2017), did not cluster with this family in our phylogenetic analyses. Many more lichen-forming genera and species need to be sampled to paint a more complete picture of phylogenetic relationships in *Malmideaceae*. Several generic names are thus in contention and, given the poor support for the ingroup phylogeny and the poor taxon sampling for our phylogenetic tree, we refrain from describing a new genus for the *L. albofuscescens* group at this time.

#### Exsiccatae (*L. streveleri*)

**Canada:** *British Columbia*: Queen Charlotte Islands [= Haida Gwaii], Moresby Island, Takakia Lake, 1967, *Brodo* 11042, *Lichenes Canadenses Exsiccati* 38 (GZU, as *L. albofuscescens*).

#### Additional specimens examined *(L. streveleri)*

**USA:** *Alaska*: Hoonah-Angoon Census Area, Glacier Bay National Park, Geikie Inlet, Shag Cove, 58°37.924′N, 136°19.715′W, corticolous on *Alnus* just above sea level, 2011, *Spribille* 36400 (MSC); *ibid.*, Bartlett Cove, 58.44557°N, 135.89712°W, just above sea level, corticolous on *Populus balsamifera*, 2012, *Spribille* 38244 (MSC); *ibid.*, East Arm of Glacier Bay, mouth of unnamed creek E of Muir Point, 58.83642°N, 136.05313°W, *c*. 8 m, corticolous on *Alnus*, 2012, *Spribille* 39197 (MSC); *ibid.*, Cross Sound, Taylor Bay at ‘campsite cove’, 58.25467°N, 136.56860°W, 4 m, corticolous on *Alnus*, 2012, *Spribille* 39707 (MSC).

#### Comparison material examined (*L. albofuscescens*; see also GLBA vouchers cited in ‘Catalogue of All Named Taxa Found’ (below))

**Austria:** *Styria*: *Mühlbacher* 230 (GZU); *ibid.*, 24 x 1962, *Schauer* s. n. (GZU).—**Canada:** *British Columbia*: Queen Charlotte Islands, *Brodo* 9723 (*Lich. Canadenses Exs*. 127, GZU).—**Finland:** Evois, ad corticem *Abies*, 1866, *Norrlin* s. n. (H-NYL 20725—holotype).—**Russia:** *Khabarovskiy Krai*: 33.7 km (air line) due W of Lazarev, up small side road in Sredniy Khrebet Mountains, between Studeniy and Zvuchnaya streams, 52°13.451′N, 141°00.428′E, corticolous on *Abies nephrolepis*, 56 m, 2009, *Spribille* 30886 (GZU); *ibid*., Sredniy Khrebet Mountains, Polоsataya Mountain, 45.5 km (air line) NW of Lazarev, between Pravaya Tumi River and Krutoberezniy stream, 52°22.779′N, 140°53.503′E, corticolous on *Abies nephrolepis* trunk, 212 m, 2009, *Spribille* 31024 (GZU); *ibid*., De Kastri-Komsomolsk route, 30 km (air line) WSW of De Kastri, near watershed divide between Chistiy and Khanda River watersheds, 51°23.260′N, 140°21.758′E, corticolous on *Picea jezoensis*, 135 m, 2009, *Spribille* 31147 (GZU).

#### Comparison material examined (*L. lesdainii*)

**Spain:** *Tenerife*: Macizo de Teno, laurisilva oberhalb von Los Silos an der Forststraße von Erjos del Tanque nach El Palmar (Monte del Agua), 900–1000 m, auf Stämmen von *Laurus*, 1984, *O. Breuss* 3680 (LI—holotype of *Helocarpon corticola* Breuss).

### Miriquidica gyrizans Fryday sp. nov.

MycoBank No.: MB 830111

Distinguished from all other species of the genus by the gyrose apothecia. Further characterized by the subsquamulose thallus and the dark hypothecium that reacts KOH+ magenta.

Type: USA, Alaska, Hoonah-Angoon Census Area, Glacier Bay National Park, Dundas Bay, unnamed mountain W of bay, 58.3421°N, 136.4015°W, 460 m, upper surface of large, flat granitic boulder on alpine ridge, 25 July 2012, *Fryday* 10175, *Spribille & Svensson* (MSC—holotype).

(Fig. 28)

*Thallus* effuse, composed of dispersed or ±contiguous areoles, 0.2‒1.0 mm across, pale brown but with an irregular layer of dead fungal cells giving them a grey appearance, areoles always associated with cyanobacteria (*Gloeocapsa*; cells 7.5‒12.5 μm diam., red (KOH+ purple), single or in groups of 2–4) either arising from a cyanobacterial mat, or on the surface or within the thallus; smaller areoles ±circular with hyaline to red-brown ±unpigmented area, larger areoles becoming divided into subareoles and developing a semi-effigurate margin; *upper cortex* absent, but upper 10 μm with a pale brown pigment; *basal layer* of thallus dark brown (KOH+ magenta); *hypothallus* lacking but space between areoles often filled with red-brown covering of *Gloeocapsa*; *medulla* I−. *Photobiont* chlorococcoid, cells 5‒9 μm diam.

**Fig. 28.**

*Miriquidica gyrizans* (holotype). A & B, habitus; C, section through apothecium; D, ascospores in KOH; E, paraphyses in KOH. Scales: A = 1 mm; B = 0.5 mm; C = 50 μm; D & E = 10 μm.

*Apothecia* frequent, scattered, lecideine, black, 0.4‒0.6 mm diam.; *proper margin* thick, raised, 0.05 mm wide; *disc* black with a central umbo when young becoming ±completely gyrose when mature. *Excipulum* dark brown, composed of radiating hyphae 5 μm wide with cortical cells 7‒8 μm wide; KOH ±magenta (Atra-red). *Hymenium* 65‒75 μm tall; *epihymenium* dilute brown, 10‒15 μm tall; *paraphyses* simple with occasional branching, 1.5‒2.0 μm wide, widening slightly (3‒4 μm) at the apex, upper 5‒10 μm with a brown pigment. *Hypothecium* dark brown to red (KOH+ magenta; Atra-red), composed of irregularly orientated hyphae 5 μm wide. *Asci* indistinct *Lecanora*-type, slightly clavate, 50‒60 × 12‒15 μm; *ascospores* simple, hyaline, becoming pigmented when overmature, broadly ellipsoid with rounded apices, thick walled, (8.5–)10.6 ± 1.6(–14.5) × (4.5–)5.4 ± 0.4(–6.5) μm, l/w ratio (1.57–)2.1 ± 0.9(–2.4), *n* = 14.

*Conidiomata* not observed

#### Chemistry

Thallus KOH−, C−, PD−; miriquidic acid by TLC.

#### Etymology

Named for the gyrose apothecia.

#### Habitat

Most frequent on the upper (flat) surfaces of large granitic boulders in snowy subalpine or alpine areas near the timberline but also from the side of a boulder at a lower altitude.

#### Notes

*Miriquidica gyrizans* is unique within the genus for its gyroid (umbonate) ascomata. The subsquamulose thallus is also unusual, being previously known in the genus only from two species from New Zealand (Fryday 2008*b*). Coincidentally, these two species are also associated with cyanobacteria. *Miriquidica gyrizans* is currently known from three areas, the Dundas Bay area of GLBA, White Pass in Klondike Gold Rush National Historical Park and two collections from Kenai Fjords National Park. An ITS rDNA sequence of a specimen from the type locality (*Spribille* 38993; voucher T1196) places it in *Miriquidica* (Fig. 10), but not enough taxa of that group have been sampled to determine which species it is most closely related to. The *Miriquidica griseoatra* group was treated by Hafellner *et al.* (2014) but the genus as a whole has not been recently revised.

#### Additional specimens examined

**USA:** *Alaska*: Hoonah-Angoon Census Area, Glacier Bay National Park, Dundas Bay, 58.350567°N, 136.399067°W, 150 m, side of granitic rock by tarn in muskeg, 2012, *Fryday* 10153, *Spribille & Svensson* (MSC); *ibid.*, unnamed mountain W of bay, 58.3422°N, 136.4002°W, 435 m, rock outcrops on alpine ridge, 2012, *Fryday* 10164, *Spribille & Svensson* (MSC); *ibid.*, 58.3421°N, 136.4015°W, 460 m, upper surface of large, flat granitic boulder on alpine ridge, 2012, *Fryday* 10173, *Spribille & Svensson* (MSC, topotype); *ibid*., eastern approach to mountain top between Dundas Bay and Fern Bay, along ridgetop, 58.34212°N, 136.40008°W, 465 m, saxicolous on large boulder in snowbed, 2012, *Spribille* 38993 (MSC); *ibid.*, 58°20.527′N, 136°24.006′W, 2012, *Svensson* 2696 (MSC); Klondike Gold Rush National Historical Park, White Pass, 58.3421°N, 136.4015°W, 460 m, top of boulder next to snowbed, 2008, *Spribille* 26813, *Pérez-Ortega & Tønsberg* (KLGO, sub *Lecanora leptacina*); *ibid*., White Pass, *Pérez-Ortega* s. n. (KLGO 53923); Kenai Peninsula Borough, Kenai Fjords National Park, near Harding Icefield Trail, ridge above Exit Glacier, 60.1828°N, 149.6713°W, on stones, flat area with late snow, 770 m, 2015, *McCune* 36369 (hb. McCune); *ibid.*, McArthur Ridge near National Park Service weather station, granitic ridgetop with alpine tundra and *Tsuga mertensiana* krummholz, 59.4726°N, 150.337°W, 386 m, 2016, *McCune* 36989, 36990b (hb. McCune).

### Niesslia peltigerae Pérez-Ort. sp. nov.

MycoBank No.: MB 830112

Similar to *Niesslia cladoniicola* but ascospores slightly smaller (7–8 × 2.5–3) and different host (*Peltigera kristinssonii*) and lifestyle (parasitic vs saprotrophic).

Type: USA, Alaska, Hoonah-Angoon Census Area, Glacier Bay National Park, muskeg and forest below Excursion Ridge, 58.45527°N, 135.57344°W, 460 m, parasitic on *Peltigera kristinssonii* growing epiphytically on *Tsuga mertensiana*, snowbed habitat, 1 August 2012, *Spribille* 39341 (MSC—holotype).

(Fig. 29)

**Fig. 29.**
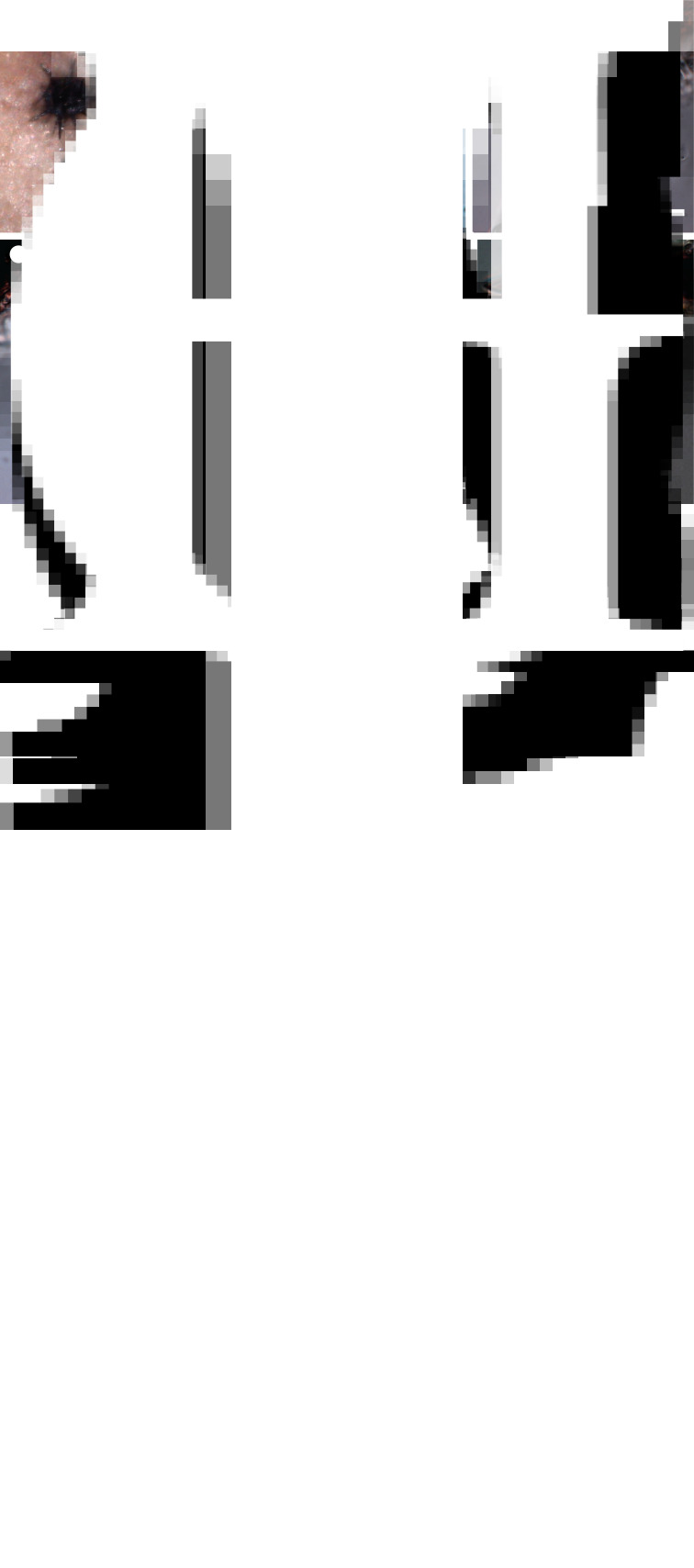
*Niesslia peltigerae* (holotype). A, ascomata, dry state collapsed; B, detail of ostiolum; C, periphyses; D, detail of perithecial wall; E, young asci; F, mature ascus; G, detail of a perithecial seta; H & I, ascospores (all except A in water, using DIC). Scales: A = 200 μm; B = 25 μm; C–F = 10 μm; G = 20 μm; H & I = 2 μm.

*Ascomata* perithecioid, sessile on the upper surface of the host thallus, 170–240 μm diam., richly setose, densely aggregated, black, subspherical, collapsed (concave) when dry, with a central ostiole. *Exciple* dark brown internal layer composed of elongated and flattened cells. *Setae* acute, with a wide base, simple, usually with one septum, dark brown, 45–95 μm long, basally to 12 μm wide (*c.* 5 μm at the median part). *Periphyses* formed around the ostiole, hyaline, to 12 μm in length. *Paraphyses* not observed in mature ascomata. *Asci* clavate, with a short ‘foot’ at the base and a truncate apex, unitunicate, 8-spored, I−, KI− (but epiplasm orange), 23–30 × 5–6 um; *ascospores* biseriate in asci, hyaline, elongated ellipsoid to fusiform, without perispore, with obtuse ends, 1-septate, 6–8 × 2.5–3(–4) μm (*n* = 20), usually with one or two guttules per cell.

#### Etymology

Named for its occurrence on a species of *Peltigera*.

#### Habitat

Parasitic, bleaching host thallus, on *Peltigera kristinssonii* Vitik. in *Tsuga mertensiana* (Bong.) Carrière parkland near the timberline.

#### Notes

The genus *Niesslia* Auersw. contains a number of lichenicolous species (Etayo *et al.* 2013), many of them growing on members of the *Peltigerales* (Etayo & Sancho 2008; Etayo 2017). *Niesslia peltigericola* (D. Hawksw.) Etayo, described originally growing on *Peltigera leucophlebia* (Nyl.) Gyeln. (Hawksworth 1980), is known to occur on several *Peltigera* species (Puolasmaa *et al.* 2012). This species differs from *N. peltigerae* by its smaller ascomata (up to 90 μm diam.), longer asci (40–58 μm), larger ascospores (10–15 × 4–4.5 μm) (Hawksworth 1980; Puolasmaa *et al.* 2012) and different lifestyle (saprotrophic or parasymbiont). Morphologically, the most similar species to *N. peltigerae* within the genus is *N. cladoniicola* D. Hawksw. & W. Gams. This species has slightly larger and narrower ascospores ((6.5‒)8.3‒10.3(‒13.1) × (1.6‒)2.2‒2.6(‒3.0) μm, but see Hawksworth (1975)) and smaller ascomata ((50‒)100‒130(‒150) μm; Zhurbenko & Pino-Bodas 2017) than *N. peltigerae* and does not cause visible damage to its host (*Cladonia* species). *Niesslia tatjanae* (S. Y. Kondr.) Etayo, growing on *Pseudocyphellaria* species and reported from Tasmania and Tierra del Fuego (Kondratyuk 1996; Etayo & Sancho 2008), also shows some similarities with *N. peltigerae* but its perithecia have shorter setae (to 40 μm long) and ascospores that are slightly larger (8‒10.5 × 2‒2.5 μm).

### Ochrolechia cooperi T. Sprib. sp. nov.

MycoBank No.: MB 830113

An *Ochrolechia* species with a smooth, creamy white thallus with numerous coralloid isidiate thallus outgrowths; apothecia apparently rare, seen in only one specimen, but differing from apothecia in *Ochrolechia yasudae* Vain. by lacking obvious pruina on the disc.

Type: USA, Alaska, Hoonah-Angoon Census Area, Glacier Bay National Park, NE of Gustavus, Falls Creek, ‘Yellowlegs Savanna’ muskeg, 58.44742°N, 135.60593°W, 245 m, on a conifer log still retaining its bark, in a muskeg ‘tree island’, 21 July 2012, *Spribille* 38730 (MSC—holotype; NY—isotype).

(Fig. 30)

**Fig. 30.**

*Ochrolechia cooperi* (holotype). A, habitus (composite image); B, detail of coralloid isidia; C, section of apothecium. Scales: A = 1 mm; B = 200 μm; C = 100 μm.

*Thallus* crustose, rimose, to 5–7 cm diam., to 1.2 mm thick, creamish white to cream, bearing scattered coralloid isidia, sometimes large parts of the thallus lacking isidia; *isidia* (Fig. 30B) 0.1–0.3(–0.5) mm diam., pinkish creamish brown to latte brown, darker than surrounding thallus starting out as globose outgrowths, branching upwards; *prothallus* not observed. *Photobiont* chlorococcoid, cells 10–15 μm diam., with a hyaline wall *c.* 1 μm wide.

*Ascomata* apothecia (Fig. 30C), round, 0.8–2.4 mm diam., observed in only one specimen, becoming hollowed out (abortive?); *thalline margin*, robust, whitish; *proper margin c.* 50 μm thick; *amphithecium* in section 190 μm wide, basally 310 μm, with algal cells occurring in small, discontinuous patches. *Hymenium* in best-developed specimens to 200 μm tall, hyaline, IKI+ hazy aqua blue, covered in crystals above, these KOH+ light yellow, dissipating; *paraphyses* thin, to 1.8 μm wide medianly, loosening in KOH. *Hypothecium* to 90 μm thick, hazy creamish. *Asci c.* 155 × 58 μm, with wall swelling to 20 μm thick in KOH; *ascospores* not seen.

*Conidiomata* not seen.

#### Chemistry

Thallus C+ red, KOH−, PD−; amphithecium cortex and epihymenium strongly C+ red; amphithecium medulla and hymenium slowly C+ red; gyrophoric acid, ±lecanoric acid by TLC.

#### Etymology

Named to mark a century since the pioneering field trips to Glacier Bay of William Skinner Cooper (1884–1978), a prominent American ecologist whose studies on plant succession in Glacier Bay and subsequent political lobbying efforts were influential in the establishment of Glacier Bay as a National Monument in 1925.

#### Habitat

On wood or bark of conifers, at low elevations from seashores to *c*. 250 m. Currently known only from southern Alaska.

#### Notes

The North American epiphytic species of *Ochrolechia* were monographed by Brodo (1991). *Ochrolechia cooperi* is readily distinguished from other species of *Ochrolechia* by its elaborate, coarse coralloid isidia that arise irregularly over the thallus. Brodo (1991) cites only one other North American species of *Ochrolechia* that develops coarse isidia, *O. yasudae*. *Ochrolechia cooperi* differs from *O. yasudae* (and the similar European species *O. subviridis* (Høeg) Erichs.) in the overall gross morphology, in which isidia are localized in coarse, shrub-like patches on an otherwise smooth thallus (compared with continuous cover of fine isidia in *O. yasudae* and *O. subviridis*), in the lack of an arachnoid hypothallus and in the epruinose apothecial discs (pruinose in the other species). Although the colour and habitus vaguely recall *O. frigida* (Sw.) Lynge, that species produces, at most, spines (in the var. *pterulina* Nyl.) but is not known to develop complex dendroid/coralloid isidia.

In recent years taxonomists have been conservative in describing new species of *Ochrolechia*, and in the absence of detailed molecular studies a large range of forms have been attributed to variability in two species, *O. androgyna* (Hoffm.) Arnold (Tønsberg 1992; Kukwa 2011) and *O. frigida* (Kukwa 2011). A DNA sequence set for *O. cooperi* was published by Resl *et al.* (2015, as ‘*Ochrolechia* sp. Spribille 38907’) in the context of a wider, eight-locus sampling of the subclass Ostropomycetidae. We combined all available, reliable, published, multilocus sequence data for *Ochrolechia* with a subset of the Resl *et al.* taxon sample to determine the broader affinities of *O. cooperi* (Fig. 6). *Ochrolechia*, in this sample, forms four main clades: 1) with *O. africana* Vain., 2) with alectoronic acid-containing species (see note under the section ‘*Ochrolechia xanthostoma* (Sommerf.) K. Schmitz & Lumbsch and similar taxa’ (below)), 3) with *O. trochophora* (Vain.) Oshio, and 4) with *O. frigida, O. androgyna* and other species. We recover *O. cooperi* in the third clade, suggesting that a close relationship to *O. frigida* can be ruled out.

*Ochrolechia cooperi* is so far known from GLBA, from the Chugach National Forest in south-central Alaska (two collections), from one site in Kenai Fjords National Park and from Mitkof Island (see specimen list below). Within GLBA it has been found only in areas not glaciated during the Little Ice Age, in the Falls Creek lowlands and Taylor Bay areas.

#### Additional specimens examined

**USA:** *Alaska*: Hoonah-Angoon Census Area, just outside Glacier Bay National Park, near upper Falls Creek hydro plant, just E of Falls Creek, *Tsuga* bark, 2012, *Spribille* 38907 (GZU; DNA voucher P150); Glacier Bay National Park, Cross Sound, Taylor Bay at ‘campsite cove’, 58.25467°N, 136.56860°W, 9 m, lignicolous on side of suspended log on high beach, 2012, *Spribille* 39501, *Fryday* 10381 (MSC); *ibid.*, 58.24684°N, 136.56791°W, 10 m, corticolous on *Tsuga heterophylla* on coastal headland, 2012, *Spribille* 39658 *& Fryday* (MSC); *ibid.*, 58.25665°N, 136.57160°W, 20 m, muskeg, corticolous (*Pinus contorta*), 2012, *Fryday* 10424 (MSC); Valdez-Cordova Census Area, Chugach National Forest, Plot CH06-Plut 71, packet no. 16, Turnagain, *Tsuga mertensiana* branch, 20 ix 2011, *K. Dillman* s. n. (ALA); *ibid.*, Plot CHUGACH 74, packet no. 16, *Tsuga mertensiana* bole, 20 viii 2012, *K. Dillman* s. n. (ALA); Kenai Peninsula Borough, Kenai Fjords National Park, peninsula into Three Hole Bay off Aialik Bay, 59.76306°N, 149.60042°W, 70–80 m, corticolous on trunk of *Tsuga mertensiana* at edge of muskeg, 2015, *Tønsberg* 45512 (ALA, BG); *ibid*., near pond at N end of Three Hole Bay off Aialik Bay, 59.78701°N, 149.60402°W, 0–10 m, corticolous on trunk of *Tsuga mertensiana*, 2015, *Tønsberg* 45528a (ALA, BG); *ibid*., corticolous at base of trunk of *Picea sitchensis*, 2015, *Tønsberg* 45534 (ALA, BG); N shore of Mitkof Island, Frederick Sound, 4 km SE of Petersburg, NW of Frederick Point, 56°47.939′N, 132°53.056′W, lignicolous on seashore driftwood, 2012, *Spribille* 37696, *Dillman, Pérez-Ortega & Wagner* (hb. Spribille).

### Placynthium glaciale Fryday & T. Sprib. sp. nov.

MycoBank No.: MB 830114

Superficially similar to *Placynthium nigrum* but with a lighter-coloured, olivaceous thallus and cuboid, submuriform ascospores.

Type: USA, Alaska, Hoonah-Angoon Census Area, Glacier Bay National Park, upper end of Muir Inlet, 59.08015°N, 136.33685°W, 50 m, saxicolous on partially buried cobbles in young post-glacial soils (exposed *c.* 30 yr BP), dense argillite-like boulder, 28 August 2014, *Spribille* 40765 *& Fryday* (MSC—holotype and one isotype; NY—two isotypes).

(Fig. 31)

**Fig. 31.**
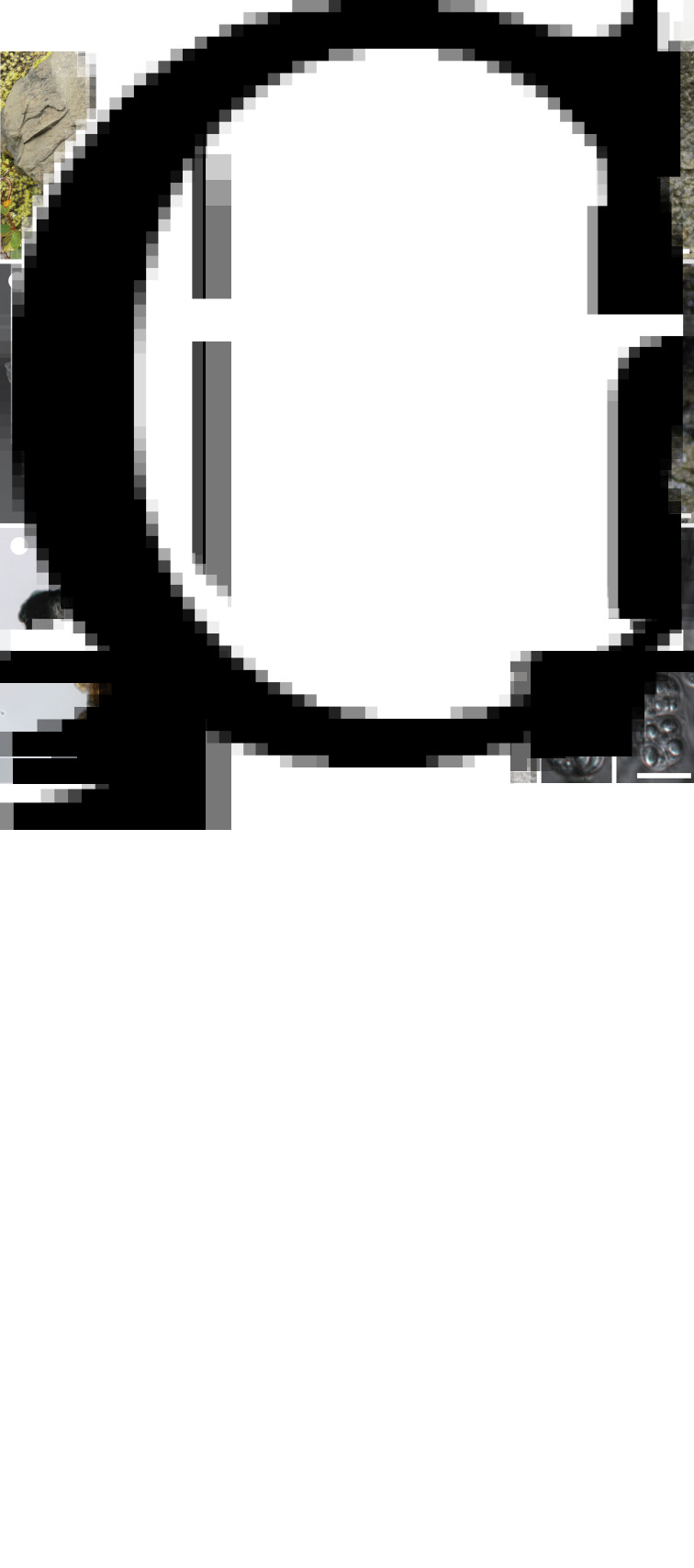
*Placynthium glaciale* (holotype). A, thalli colonizing recently deglaciated rocks (*c.* 30 cm diam.) at type locality; B & D, habitus; C, detail of apothecium and branches with SEM; E, section of apothecium; F, asci and paraphyses, in Lugol's solution; G–J, ascospores, in K. Scales: B = 1 mm; C & D = 200 μm; E = 100 μm; F–J = 10 μm (scale bar the same for G–J).

*Thallus* crustose, rimose, olivaceous brown, to 3.5 cm diam. and 0.2 mm thick, covered in crowded, opuntioid, dactyloid lobes; *individual lobes* (30–)70–150 μm diam., in section weakly stratified, with continuous cellular (paraplectenchymatous) cortex, individual cells roundish, 4–6 μm diam.; *hypothallus* apparently absent or present, bluish, KOH+ mauve, not extending beyond edge of main thallus. *Photobiont* an unknown cyanobacterium, individual cells roundish, 6–7 μm diam., sometimes forming chains, easily dislodged from thallus and floating free in microscopic section.

*Ascomata* apothecia, round, scattered, (0.3–)0.48–0.58(–0.8) mm diam., the disc flat to concave or weakly convex, dark brown to jet black, matt; *proper margin* prominent and remaining so with age, black, lustrous, sometimes slightly incurved and covering the disc. *Excipulum c.* 50 μm wide laterally, 50–90 μm wide basally, purplish internally, blue-black towards the outer edge, the pigments KOH−, non-amyloid; composed of radiating hyphae *c.* 5 μm thick in the middle part of the excipulum, with terminal cells to 10 μm wide, with wide lumina and a wall 1–1.5 μm thick. *Hymenium* 65–90 μm tall, streaked blue-black, I+ blue before treatment with KOH, pigments more intense towards the top; *paraphyses* septate, mostly straight, weakly branched and anastomosing or with short ‘thumb’ branches, 1.8–2.0 μm wide medianly, swelling to 3 μm in KOH. *Hypothecium* to 230 μm thick, of which the top 50–60 μm differentiated into a hyaline to pale brown subhymenium and the lower part is pale brown, composed of tangled hyphae to 2.5 μm wide, I+ blue after KOH. *Asci* (Fig. 31F) 53–60 × 11–16 μm, with 5–7 ascospores developing to maturity, asci dehiscing when ascospores become submuriform, no asci with a full complement of 8 ascospores observed; *ascospores* (Fig. 31G–J) round to square or cuboid, frequently isodiametrical, submuriform, beginning simple and divided first medianly, then transversely, (6–)8.4–10.8(–13) × (5.5–)6.4–7.1(–9) μm, l/w ratio 1.4 ± 0.3 (*n* = 45, five samples used).

*Conidiomata* not observed.

#### Chemistry

All spot tests negative.

#### Etymology

Named for its close association with glacial forelands.

#### Habitat

On siliceous rock (mostly soil-embedded small boulders, 20–40 cm diam.) in recently deglaciated forelands.

#### Notes

The genus *Placynthium* is still poorly known in North America, having last been revised over 50 years ago (Henssen 1963). However, no species of *Placynthium* has been reported to have muriform ascospores. We obtained DNA from two specimens (Table 1) and a phylogenetic analysis (Fig. 9) shows *P. glaciale* to be included in a broader clade that contains the *P. asperellum* (Ach.) Trevis. group, *P. subradiatum* (Nyl.) Arnold and two accessions that are treated here as *Placynthium* sp. S38458 (isolates T1310 and T1304, see ‘Known Unknowns’ section).

So far *P. glaciale* is known only from the forelands of Muir Glacier in the upper East Arm of Glacier Bay, which would have been first exposed by the retreating Muir Glacier *c.* 30 yr BP, and a marble rock face on the shore of the terminal lake of Patterson Glacier (Alaska, Petersburg Borough; see below) exposed at a similar time.

#### Additional specimens examined

**USA:** *Alaska*: Hoonah-Angoon Census Area, Glacier Bay National Park, East Arm of Glacier Bay, upper end of Muir Inlet, north shore, lower mid slope, 59°05.034′N, 136°20.179′W, saxicolous on 30 yr-old surfaces, 115 m, 2011, *Spribille* 36576 (sterile), 36580, 36581 (MSC); *ibid.*, 59°04.953′N, 136°20.229′W, 30 m, siliceous rock and pebbles, 2011, *Fryday* 9786, 9791 (MSC); Petersburg Borough, Patterson Glacier, 56.939363°N, 132.654869°W, 105 m, marble rock face on the shore of the terminal lake, 2015, *Fryday* 11175 (MSC).

### Porpidia seakensis Fryday sp. nov.

MycoBank No.: MB 830115

Separated from other species of the genus by the combination of a thin to endolithic thallus, strongly constricted apothecia with a brown pruinose disc, large ascospores and a macrocarpa-type exciple.

Type: USA, Alaska, Hoonah-Angoon Census Area, Glacier Bay National Park, Bartlett Cove, road between Park HQ and dock, 58.4569°N, 135.8628°W, 40‒50 m, granitic rock in woodland, 14 August 2012, *Fryday* 10485 (MSC—holotype).

(Fig. 32)

**Fig. 32.**

*Porpidia seakensis*. A–C, habitus (A & B, *Fryday* 9626; C, holotype); D & F, section through apothecium under brightfield (D) and polarized (F) light; E, asci, ascospores and paraphyses, in KOH (D–F from holotype). Scales: A = 0.5 mm; B = 0.2 mm; C = *c.* 0.2 mm; D & F = 50 μm; E = 10 μm.

*Thallus* completely endolithic on granite but present as a thin effuse white stain when on schist and becoming slightly areolate in depressions; *medulla* I−. *Photobiont* chlorococcoid, cells globose, 8‒15 μm diam.

*Apothecia* scattered, lecideine, 0.4‒0.8(‒1.0) mm across, distinctly constricted below; *disc* red-brown, lightly pruinose; *proper margin* slightly raised, 0.05‒0.08 mm wide, inrolled and paler at the inner edge. *Excipulum* internally pale- to red-brown with darker brown cortex, 20 μm wide, composed of radiating hyphae 6‒10 μm wide; extending below the hypothecium where it can be 60‒100 μm thick. *Hymenium* 125 μm tall; *subhymenium* 25‒40 μm thick; *epihymenium* dilute brown, 20‒25 μm thick with small granules that do not dissolve in KOH; *paraphyses* narrow, 1 μm wide, branched and anastomosing, only slightly wider at the apex (up to 2 μm) which is not pigmented. *Hypothecium* dark brown, 100 μm thick. *Ascus Porpidia*-type, cylindrical to slightly clavate, 80‒95 × (12‒)15‒20 μm; *ascospores* hyaline, simple, ellipsoid with attenuated apices, (17.0–)21.5 ± 2.3(–24.0) × (7.5–) 9.2 ± 0.4(–10.5) μm, l/w ratio (2.0–)2.4 ± 0.4(–3.1) *n* = 17, with a thin perispore 2 μm thick.

*Conidiomata* not observed.

#### Chemistry

Thallus KOH+ yellow, C−, PD+ orange; stictic acid by TLC.

#### Etymology

A play on the common abbreviation for south-east Alaska (‘SE AK’).

#### Habitat

On siliceous rocks and boulders in open, well-lit wooded areas.

#### Notes

*Porpidia seakensis* (Fig. 32) is a distinctive species that has so far not been found outside the Bartlett Cove area of GLBA, although it is common there. The only other species of the *Porpidia macrocarpa* group (*macrocarpa*-type exciple, stictic acid complex; Gowan 1989) with large ascospores (>20 μm) is *P. superba* (Körb.) Hertel & Knoph, which has thick, bullate areoles and an orange-brown exciple (Superba-brown).

Two DNA sequences from *Porpidia seakensis* (28S rDNA and *Mcm7*) were obtained for this study but were not incorporated into any tree because of insufficient overlap with previously sampled loci in the genus; ITS, which has been used for example by Orange (2014) in studying relationships in *Porpidia*, was not successfully amplified from our isolate. The sequences are published here (Table 1) for future reference.

#### Additional specimens examined

**USA:** *Alaska*: Hoonah-Angoon Census Area, Glacier Bay National Park, Bartlett Cove, ‘river trail’, 58.4569°N, 135.8628°W, 40‒50 m, schistose rock in woodland, 2011, *Fryday* 9626 & *Spribille* (MSC); *ibid*., 58.4569°N, 135.8628°W, 2012, *Fryday* 10486 (MSC); *ibid.,* Bartlett Cove, entrance to Park HQ, 58°27.265′N, 135°52.380′W, 25 m, small siliceous boulder, 2011, *Fryday* 9648 (MSC); *ibid*., Bartlett Cove, 25 m E of Service Rd, N side, 58.4541°N, 135.8793°W, 20–25 m, granitic boulder, 2012, *Fryday* 10490, 10491, 10493 (MSC); *ibid.*, Bartlett Cove, housing complex, 58.4556°N, 135.8756°W, 15 m, flat stones, 2012, *Fryday* 10499 (MSC).

### Rhizocarpon haidense Brodo & Fryday sp. nov.

MycoBank No.: MB 830116

Similar to *Rhizocarpon infernulum* (Nyl.) Lynge and *R. cinereovirens* (Müll. Arg.) Vain. but distinguished from *R. infernulum* by its unpigmented or pale brown exciple, and from *R. cinereovirens* by its smaller ascospores, predominantly brown epihymenium (Cinereorufa-green absent or present in low quantities), by its thallus containing stictic acid (norstictic acid absent or in trace amounts only) and lack of a well-developed prothallus.

Type: Canada, British Columbia, Haida Gwaii (‘Queen Charlotte Islands’), Moresby Island, Jedway, along Skincuttle Inlet, E of settlement, 52°19′N, 131°12′W, rocky shore and *Picea-Tsuga* forest, on rock at base of cliff at edge of beach, 25 July 1967, *I. M. Brodo* 12480, *Shchepanek & Schofield* (CANL—holotype).

(Fig. 33)

**Fig. 33.**

*Rhizocarpon haidense* (holotype). A & B, habitus; C & D, section through apothecium under polarized (C) and brightfield (D) light; E, ascospores in ascus, with paraphyses, using DIC microscopy. Scales: A = 5 mm; B = 1 mm; C & D = 50 μm; E = 10 μm.

*Thallus* effuse, creamy to pale brown, thin, cracked areolate, areoles angular, slightly convex, 0.3–0.4 mm across; cortex absent; *medulla* with numerous minute crystals that dissolve in KOH to give a yellow solution, I−. *Photobiont* chlorococcoid, cells globose, 7–12 μm diam.

*Apothecia* lecideine 0.4–0.7 mm diam., flat to slightly convex; *disc* dark brown to black; *proper margin* persistent, 0.05 mm wide, slightly raised, paler than the disc. *Exciple* composed of hyaline, radiating cells *c.* 8–10 × 5–8 μm that are largely obscured by numerous minute crystals that dissolve in KOH to give a yellow solution; *cortical cells* pale brown, 5–6 μm diam. *Hymenium* hyaline, 90–100 μm tall; *paraphyses* sparingly branched and anastomosing, 2–2.5 μm thick, not or only slightly wider at the apex; *epihymenium* upper 10 μm diffuse brown but usually HNO_3_+ red indicating presence of small quantities of Cinereorufa-green. *Hypothecium* dark brown, upper part of vertically orientated hyphae intergrading into the hymenium, lower part of more randomly orientated hyphae. *Asci Rhizocarpon*-type, 50–60 × 15–18 μm, slightly clavate; *ascospores* hyaline, 1-septate, (14–)16.7 ± 2.4(–22) × (6.5–)7.8 ± 1.0(–9.5) μm, l/w ratio (1.7–)2.2 ± 0.4(–2.9), *n* = 20, slightly constricted at the septum, apices equally rounded.

*Conidiomata* not observed.

#### Chemistry

Thallus KOH+ yellow, PD+ orange, C−; stictic and constictic acids by TLC, occasionally with a trace of norstictic acid and other unknown substances.

#### Etymology

For Haida Gwaii, the type locality and where the species was first detected.

#### Habitat

On siliceous rock near the coast, often in the aerohaline zone.

#### Notes

*Rhizocarpon haidense* is similar to *R. cinereovirens,* with which it shares a pale brown to hyaline proper exciple (giving the apothecia a pale margin), a character that readily separates these two species from *R. infernulum.* However, it differs from *R. cinereovirens* in that the thallus produces stictic acid as the major substance rather than norstictic acid, although this is sometimes present in trace amounts. The epihymenium of *R. haidense* also lacks appreciable amounts of Cinereorufa-green, the pigment present in *R. cinereovirens* (although again this is usually present in trace amounts causing the epihymenium to react HNO_3_+ red). As noted previously (Fryday 2002), the production of Cinereorufa-green in the epihymenium of *R. infernulum* appears to be related to the degree of exposure to ultraviolet light, with the substance being present in larger amounts in specimens from exposed situations. However, the difference between *R. haidense* (brown epihymenium) and *R. cinereovirens* (blue-green hymenium) appears to be consistent. The last two species also differ in *R. haidense* having somewhat smaller ascospores ((17–)19.5(–22) × (7–)8.8(–10) μm in *R. cinereovirens*) and being known only from maritime rocks in NW North America, where *R. cinereovirens* has yet to be reported. *Rhizocarpon haidense* also usually has a much less well-developed thallus and smaller apothecia, although this is probably an environmental modification to its habitat on marine rocks and specimens from less extreme environments are difficult to separate from *R. cinereovirens* macroscopically.

DNA sequences were obtained from three gene loci and are used in Figs 5 and 10. However, an analysis of the placement of these sequences relative to other species in the genus *Rhizocarpon* was not attempted for this study to avoid pre-empting the work under preparation by collaborators.

#### Additional specimens examined (all Canadian collections in CANL)

**Canada:** *British Columbia*: Haida Gwaii (Queen Charlotte Islands), Graham Island, 11.2 miles N of Skidegate Mission, 53°26′N, 132°54′W, 1967, *Brodo* 10046 *& Shchepanek*; *ibid.*, Tana Bay, 53°11′N, 132°33′W, on rock just above shore, 1967, *Brodo* 10157, *Shchepanek & Schofield*; *ibid.*, Seal Inlet in Rennell's Sound, 53°31′N, 132°44′W, on shaded rock above littoral zone, 1967, *Brodo* 10274, *Shchepanek & Schofield*; *ibid.*, 53°29′N, 132°47′W, in lower aerohaline, 1967, *Brodo* 10334, *Shchepanek & Schofield*; *ibid.*, north side of Russell Sound, 53°23′N, 132°31′W, 1967, *Brodo* 10340, *Shchepanek & Schofield*; *ibid.*, head of Tian Bay, 53°47′N, 133°04′W, in aerohaline, 1967, *Brodo* 10522, *Shchepanek & Schofield*; *ibid.*, Langara Island, off NW corner of Graham Island, Henslung Harbour (Dadens), 54°12′N, 133°00′W, in aerohaline, 1967, *Brodo* 10586, *Shchepanek & Schofield*; *ibid.*, Haida Point, 53°15′N, 132°01′W, 1988, *Brodo* 11730A *& Shchepanek*; *ibid.*, near head of Dinan Bay, 53°39′N, 132°41′W, on rock at edge of beach, 1971, *Brodo* 18362, *Wong & Turner*; *ibid.*, Cone Head, SW corner of Rennell Sound, 53°23′N, 132°39′W, *Coccotrema* zone, 1988, *Brodo* 26907; *ibid.*, Gregory Beach, 53°24′N, 132°31′W, on headland rocks in *Coccotrema* zone, 2000, *Brodo* 302650, *F. G. Brodo & Bettner*; Burnaby Island, Section Cove, 52°24′N, 131°21′W, 1971, *Brodo* 17557A, *Wong & Schofield*; *ibid.*, Pelican Point, 52°24′N, 131°16′W, 2000, *Brodo* 17557A *& Sloan*; Skidegate Inlet, Maude Island, 53°12′N, 132°05′W, 1967, *Brodo* 11284 *& Shchepanek*; *ibid.*, outside Long Inlet, 53°21′N, 132°21′W, 1967, *Brodo* 11392 *& Shchepanek*; Moresby Island, between De la Beche and Haswell Bays, 52°32′N, 131°36′W, 1967, *Brodo* 11955, 11961 *& Shchepanek*; *ibid.*, Kootenay Inlet, 52°51′N, 132°13′W, 1967, *Brodo* 12138, 12168 *& Shchepanek*; *ibid*., Louscoone Inlet, 52°13′N, 131°15′W, on rocks at edge of beach, 1967, *Brodo* 12309, 12319 *& Shchepanek*; *ibid.*, Tasu, Growing Island, 52°46′N, 132°02′W, in aerohaline, 1967, *Brodo* 12851, *Shchepanek & Schofield*; *ibid.*, south entrance to Louise Narrows, 53°57′N, 132°54′W, 1967, *Brodo* 17922*,Wong & Schofield*; Limestone Island, Boat Cove, 52°54′N, 131°36′W, on shoreline rocks above *Coccotrema* zone, 2000, *Brodo* 30117 *& F. B. Brodo*; Kunghit Island, Luxana Bay area, 52°04′N, 131°00′W, on shoreline rocks 4.5 m above barnacle zone, 2000, *Brodo* 30015 *& Sloan*; Vancouver Island, south end of Long Beach, 49°01′N, 125°40′W, 1969, *Shchepanek* 250, *Soper & Brayshaw*.—**USA:** *Alaska*: Juneau, Aaron Island, 7 miles W of Auke Bay, 58°27′N, 134°49′W, on shaded shoreline rock, 1988, *Brodo* 10586, *Hart & F. Brodo* (CANL); Hoonah-Angoon Census Area, Glacier Bay National Park, Taylor Bay, 58.25467°N, 136.56860°W, 0–5 m, supralittoral zone, above splash zone, metamorphic rock (Hornblende augen gneiss), 2012, *Fryday* 10452 *& Spribille* (MSC); *ibid.*, 9 m, vertical shale face at sea level, 2012, *Spribille* 39505 *& Fryday* (MSC); Petersburg Borough, Kupreanof Island, Little Duncan Bay, 56.6193°N, 133.1645°W, sea level, siliceous rocks by shore, 2014, *Fryday* 10680, *Spribille & Dillman* (MSC).

#### Comparative specimens examined (*R. cinereovirens*)

**USA:** *South Dakota*: Custer Co., above Game Lodge in Custer State Park (11 miles E of Custer), on N-facing hill, 4300 ft, Sec 27, T3S, R6E, 1960, *Wetmore* 7575 *(*MSC). *New York*: Suffolk Co., Amagansett, Bunker Hill on Bunker Hill Road, oak-hickory woods, 1961, *Brodo* 3265 (MSC).

### Sagiolechia phaeospora Fryday & T. Sprib. sp. nov.

MycoBank No.: MB 830117

A *Sagiolechia* species differing from all other species in the genus in its brown, submuriform ascospores and association with a non-trentepohlioid alga as a photobiont.

Type: USA, Alaska, Hoonah-Angoon Census Area, Glacier Bay National Park, Excursion Ridge, 58.46469°N, 135.55736°W, 918 m, saxicolous on argillite, 1 August 2012, *Spribille* 39391 (MSC—holotype).

(Fig. 34)

**Fig. 34.**

*Sagiolechia phaeospora* (holotype). A, habitus; B, section through apothecium (composite image); C, ascus and immature and mature ascospores; D, overmature ascospores (C & D in Lugol's solution after KOH). Scales: A = *c.* 1 mm; B = 50 μm; C & D = 10 μm.

*Thallus* crustose, thin, whitish, cracked areolate to rimose; areoles/cracked partitions if present 0.4–0.9 mm diam., internally hardly stratified, POL+ birefringent. *Photobiont* a single-celled green alga; *cells* roundish or angular, 10–14 μm diam.

*Ascomata* apothecia, jet black, matt, 0.6–1.0 mm diam.; *disc* effigurate, with a central umbo up to 220 μm wide, composed of thick-walled hyphae 4.5–6 μm diam.; *proper margin* prominent, black. *Excipulum* 140–150 μm wide laterally, the outer 30 μm deeply pigmented (‘carbonized’), internally medium to dark brown, 60 μm wide basally, I+ blue before pretreatment with KOH. *Hymenium* 180–200 μm tall, hyaline, I+ blue; *paraphyses* predominantly simple, thin, 1–1.5 μm diam., not widened apically, upper 1/4 of hymenium brownish pigmented. *Hypothecium* 50–150 μm tall, hyaline. *Asci* 8-spored, oblong-cylindrical, *c.* 70–72 × 16–17 μm; *ascospores* submuriform, brownish, (14–)17.8–18.5(–22) × (8–)9.5–10.6(–14) μm (*n* = 21, two specimens used).

*Conidiomata* sunken, inconspicuous; *conidia c.* 3 × 1 μm.

#### Chemistry

All spot tests negative.

#### Etymology

In reference to the brownish ascospores.

#### Habitat

Saxicolous on small slabs of argillite rock in snowbed habitats in the low alpine zone (*c.* 920 m).

#### Notes

*Sagiolechia* A. Massal. is a loosely defined genus that includes species with hyaline, transversely septate ascospores, deeply melanized (‘carbonized’) apothecial pigments and an association with *Trentepohlia* Mart. photobionts, together with at least one lichenicolous species not considered to possess its own thallus. Four species are recognized: *S. fusiformis* (Müll. Arg.) Zahlbr., from rocks in Japan (Mueller 1892); *S. protuberans* (Ach.) A. Massal., which is widespread on calcareous rocks (Vězda 1967); *S. atlantica* Henssen on volcanic conglomerate on Madeira (Henssen 1995); *S. parasitica* Alstrup & E. S. Hansen, which is lichenicolous on *Hymenelia cyanocarpa* (Anzi) Lutzoni in Greenland (Alstrup & Hansen 2001). Many authors have segregated the arctic-alpine species *Sagiolechia rhexoblephara* (Nyl.) Zahlbr, which would count as a fifth species, into a distinct monotypic genus, *Rhexophiale* Th. Fr., on account of the different observed ontogeny of its ascomata (Henssen 1995).

*Sagiolechia phaeospora* (Fig. 34) is the first described member of the genus with brown ascospores (Fig. 34C & D), the first species with muriform ascospores, and the first associated with a non-trentepohlioid photobiont. In our phylogenetic analysis (Fig. 7), we analyzed only one specimen each of *S. protuberans* and *S. phaeospora*, but even this limited sampling makes two things clear: 1) *S. phaeospora* forms a clade with the type species of *Sagiolechia*, *S. protuberans*, and 2) both species form a small monophyletic group with *Rhexophiale rhexoblephara* (Nyl.) Almq., challenging Henssen's (1995) hypothesis that observed ontogenetic differences reflect deep evolutionary splits. Our result supports the close relationship postulated between *S. protuberans* and *S. rhexoblephara* in the anatomical study of Vězda (1967).

An additional, possibly related species has since been seen by one of us (AMF, 27 May 2014) from Ungava Bay (Québec, Nord-du Québec, Kativik, near Tasiujaq, 58.82833°N, 69.88778°W, 36 m, maritime outcrops (Labrador Trough), Site 86.12b, 29 July 2013, *J. Gagnon* s. n. (QFA)) and appears to be undescribed. It has brown 3-septate ascospores (*c*. 18 × 6 μm), the apothecia are often short-lirelliform, and as with *S. phaeospora* the associated photobiont is not *Trentepohlia*. Also probably referable to *Sagiolechia*, perhaps representing yet another species, is a collection from the terminal moraine of Baird Glacier (Alaska, Petersburg Borough, *Fryday* 11235, MSC) that has small apothecia with a carbonized exciple and hyaline 3-septate ascospores.

*Sagiolechia phaeospora* was collected three times during the present survey, in the same area of alpine heath on Excursion Ridge in the south-eastern corner of GLBA.

#### Additional specimens examined

**USA:** *Alaska*: Hoonah-Angoon Census Area, Excursion Ridge, 58.46274°N, 135.55288°W, 919 m, saxicolous on argillite in alpine heath tundra, 2012, *Spribille* 38406 (MSC; DNA isolate T1184); ibid., 58.4635°N, 135.55809°W, 915 m, saxicolous, 2012, *Svensson* 2633 (MSC).

### Sclerococcum fissurinae Pérez-Ort. sp. nov.

MycoBank No.: MB 830129

Lichenicolous on *Fissurina insidiosa*. Similar to *Sclerococcum parasiticum* but ascospores are slightly smaller (8–12 × 3–4 μm vs 9–15 × 3.5–5 μm) and it has a different host species.

Type: USA, Alaska, Hoonah-Angoon Census Area, just outside Glacier Bay National Park boundaries, 6.5 km NE of Gustavus, along Falls Creek Road, 58.43899°N, 135.64068°W, on *Fissurina insidiosa* corticolous on *Alnus* along side drainage, 37 m, 28 July 2012, *Spribille* 39036 (NY—holotype; GZU, H, UPS—isotypes).

(Fig. 35)

**Fig. 35.**

*Sclerococcum fissurinae* (holotype). A, ascomata on *Fissurina* thallus; B, transverse section of ascoma; C, detail of excipulum; D, mature ascus with ascospores and paraphyses; E, young ascospore; F & G, mature ascospores (B–H in water and using DIC microscopy). Scales: A = 0.5 mm; B = 50 μm; C = 10 μm; D–G = 5 μm.

*Apothecia* rounded, to 0.6 mm diam.; *disc* flat, black; *proper margin* distinct, concolorous with the disc. *Excipulum* upper part 25–40 μm wide in section, red-brown to maroon, KOH+ dark olivaceous green, HNO_3_+ becoming orange, composed of elongated, more or less radiating hyphae, becoming prismatic at the base, 6–15 × 5–7 μm diam. *Hymenium* hyaline, 35–50 μm tall; *epihymenium* reddish brown to maroon, KOH+ dark olivaceous green, HNO_3_+ turning vivid orange; *paraphyses* simple, 1–2 μm wide, enlarged at the apex (up to 6 μm wide), with swollen and reddish brown apical caps. *Hypothecium* reddish brown to maroon, up to 30 μm high. *Asci* with a K/I+ blue outer layer, 8-spored, 25–33 × 8–12 μm; *ascospores* brown, ellipsoid, ornamentation granulate when young, usually disappearing when mature, 3-septate, more rarely 1–2-septate, straight or rarely slightly curved, not or slightly constricted at the septa, (8–)10–12(–14) × 3–4 μm (*n* = 35).

#### Etymology

Named for its occurrence on *Fissurina*.

#### Habitat

On *Fissurina insidiosa* C. Knight & Mitt. in *Alnus*-dominated riparian areas, known so far only from GLBA but likely to be widespread in outer coastal SE Alaska.

#### Notes

Diederich *et al.* (2018) synonymized the apotheciate genus *Dactylospora* Körb. with the sporodochia-producing genus *Sclerococcum* Fr., based on molecular data. Several species of the genus *Sclerococcum* possess 3-septate ascospores (Triebel 1989; Ihlen *et al.* 2004). The most similar to *S. fissurinae* (Fig. 35) are *S. attendendum* (Nyl.) Ertz & Diederich and *S. parasiticum* (Flörke) Ertz & Diederich. The former has larger ascospores (12–15 × 4.5–5.5 μm) and the apothecia usually have a rough or radially striate margin. Furthermore, this species is known growing only on crustose saxicolous species such as *Amygdalaria* Norman, *Pertusaria* s. str., *Pilophorus* Th. Fr. and *Porpidia* Körb. species (Triebel 1989). *Sclerococcum parasiticum*, by contrast, has slightly larger ascospores (9–15 × 3.5–5 μm) and usually grows on *Lepra* and *Ochrolechia* species (also on *Mycoblastus sanguinarius* (L.) Norman; see ‘Catalogue of All Named Taxa Found’ (below)).

#### Additional specimens examined

**USA:** *Alaska*: Hoonah-Angoon Census Area, Icy Straits, ridge above Fern Harbor, 58.31133°N, 136.45689°W, corticolous on *Tsuga heterophylla* branches, 86 m, 2012, *Spribille* 38091 (MSC); just outside Glacier Bay National Park boundaries, 6.5 km NE of Gustavus, along Falls Creek Road, 58.43899°N, 135.64068°W, corticolous on *Alnus* along side drainage, 37 m, 2012, *Spribille* 39050 (GZU—topotype).

### Spilonema maritimum T. Sprib. & Fryday sp. nov.

MycoBank No.: MB 830119

Similar to *Spilonema paradoxum* but differing in developing concentrically radiating, flattened thalli and in its close association with supralittoral habitats.

Type: USA, Alaska, Juneau Borough, west side of Douglas Island at Peterson Creek beach access, 58.285534°N, 134.674141°W, on exposed seashore rocks at back of beach, about two metres above high tide line, 21 September 2010, *Spribille* s. n. (UPS—holotype; BG, GZU, MSC, NY—isotypes).

(Figs 36 & 37)

*Thallus* composed of radiating filaments, forming rosettes 0.5–1.5 cm diam., olivaceous brown; *individual filaments* sausage-like, 19–40 μm diam. (visible below apothecial section in Fig. 37A), with isidioid spinules to 18 μm diam. (Fig. 36D–F); entire thalline area richly occupied by minute bacterial biofilms and becoming conglutinated in a ‘biofilm cake’ with age (Fig. 36C); thallus in section composed of a *Stigonema*-like cyanobacterium 7–13 μm diam., enmeshed with fungal hyphae, the fungal-cyanobacterial contact areas I+ blue after KOH pretreatment, the whole filament ensheathed in a gelatinous layer; *cellular cortex* absent; *hypothallus* bluish, KOH+ mauve.

**Fig. 36.**

*Spilonema maritimum*. A–C, habitus of healthy (A & B) and eroded (C) thalli, indicating in (B) an apothecium (arrow) and pycnidia (asterisks) (A, isotype; B, *Fryday* 10389; C, *Spribille* 39589); D–F, details of thallus surface with SEM, including upper (D & E) and lower (F) surfaces (*Fryday* 10389). Scales: A & C = 1 mm; B = 0.5 mm; D = 200 μm; E = 50 μm; F = 100 μm.

**Fig. 37.**

Anatomical details of the apothecium in *Spilonema maritimum* (holotype). A, section; B, detail of excipular hyphae; C–E, ascus apical structures in Lugol's solution, at full concentration (C) and while fading (D); E, the same as D but using DIC microscopy; F–H, ascus apical structure in dissipating Lugol's solution, arrows indicate amyloid apical tube. Scales: A = 100 μm; B–H = 10 μm.

*Ascomata* apothecia, rare, round, single, 0.15–0.55 mm diam., difficult to distinguish from pycnidia when wet; *disc* convex blue-black, shiny; *proper margin* excluded from surface view. *Excipulum* (Fig. 37B) of radiating hyphae to 6 μm diam., streaked with bluish black pigments; *hyphae* internally with large, angular lumina, non-amyloid. *Hymenium* 42–70 μm tall, bluish streaked with pigments concentrated at the base of the hymenium, I+ blue before KOH; top of hymenium black-bluish, HNO_3_+ mauve, KOH−; *paraphyses* septate, sparsely branched, with bulges, 1.5–2.5 μm wide medianly, swelling to 3 μm in KOH and 5 μm terminally. *Hypothecium* 100–140 μm tall, pale blue to hyaline, composed of thin-walled hyphae with lumina 3–8 μm diam; upper part I+ blue; *subhymenium* weakly differentiated. *Asci* 8-spored, 30–42 × 10–13 μm, covered in a massive I+ deep blue gelatinous sheath and thus very difficult to visualize because the ascus turns almost completely black in Lugol's solution (Fig. 37F–H); *ascospores* sparse and hard to find, simple, ellipsoid, (6.5–)7.8–8.9(–12) × (2.5–)3.6–3.9(–5) μm (*n* = 16, two samples used).

*Pycnidia* present in four of five specimens studied, shiny black, pigmented bluish to greenish black internally, pyriform, opening up to gaping, 130–190 μm diam.; *conidia* bacilliform, straight, 3–4.2 × 1.3–2 μm.

#### Chemistry

All spot tests negative; no substances detected by TLC.

#### Etymology

Named for its close association with maritime rocks.

#### Habitat

On rocks in the supralittoral zone, known in GLBA from Taylor Bay and Fern Harbor, and outside of GLBA from Kruzof and Douglas Islands, Alaska, as well as from Haida Gwaii and Vancouver Island, British Columbia.

#### Notes

*Spilonema maritimum* was initially thought to be a member of the Lichinomycetes, perhaps related to *Ephebe* Fr., but discovery of fertile material showed that it belongs to the *Peltigerales* in the Lecanoromycetes. Spribille *et al.* (2014*a*) published a molecular phylogeny of *Spilonema* Bornet, a small genus with three named species to date, *S. americanum* (Henssen & Tønsberg) T. Sprib. *et al*., *S. revertens* Nyl. and *S. paradoxum* Bornet. *Spilonema maritimum* clearly does not fit into any of these species and is distinguished by its flattened, radiating outer lobes and filamentose centre, and the tendency to become conglutinated with age. Similar to *Spilonema* sp. 1 (Spribille *et al.* 2014*a*), it occupies supralittoral habitats, but it differs from that undescribed species in its radiating habit and lack of thalline mounds. The metagenomic DNA obtained from samples of *S. maritimum* yielded PCR products either a) containing so much superimposed signal that the traces were unusable, or b) with readable sequences that clearly come from multiple fungal genomes. One of these was an unidentified putative *Orbiliaceae* (LSU sequence from isolate T1338; Table 1); another came from the lichenized class Lichinomycetes (mtSSU from isolate T1808; Table 1), which caused us to again reassess our assumptions regarding the main fungal component of this lichen. One mitochondrial SSU sequence (from isolate T1338, used in Fig. 9) aligned closely with previously obtained sequences from *Spilonema* and because this agrees with the fungal morphology, we have deposited this as putatively belonging to *S. maritimum*. The multiple fungal species we detected in *S. maritimum* highlight the difficulties in sequencing fungal DNA with general fungal primers without knowing the cellular source of the genomic DNA. However, we note that all aspects of ascomatal anatomy are consistent with other species of *Spilonema*.

*Spilonema maritimum* is found in the upper supralittoral zone in the Icy Straits near the open ocean in GLBA. It appears to be common along the outer Pacific coast of NW North America as far as southern Vancouver Island. According to I. M. Brodo (personal communication, 2019), material of *S. maritimum* was the basis for the first report of *S. revertens* Nyl. from British Columbia (Benton *et al.* 1977). That species is now known from other localities in British Columbia but occurs in rock crevices or over mosses in dry, inland habitats, unlike *S. maritimum*. The distinctive position of *S. maritimum* in the zonation of maritime rocks on Haida Gwaii is discussed by Brodo & Sloan (2005, as *S. revertens*).

#### Additional specimens examined (all Canadian specimens from CANL)

**Canada:** *British Columbia*: Queen Charlotte Islands [ = Haida Gwaii], Maude Island in Skidegate Inlet, 53°12′N, 132°05′W, on rocks at edge of beach in aerohaline, 1967, *Brodo* 11253 *& Shchepanek*; *ibid.*, Torrens Island, Skidegate Inlet, 53°15′N, 131°59′W, on rocks, upper aerohaline, 1971, *Brodo* 17309 & *Wong*; *ibid.*, Murchison Island, 52°36′N, 131°28′W, in lower aerohaline, 1967, *Brodo* 11862 *& Shchepanek*; *ibid.*, Hibben Island, off the W coast of Moresby Island, 53°00′N, 132°22′W, rocks high above water, 1968, *Brodo* 14029; *ibid.*, Lina Island, Skidegate Inlet, 53°13′N, 132°08′W, exposed shoreline rocks, aerohaline, 1967, *Brodo* 11337 *& Shchepanek*; *ibid.*, Skidegate Inlet, Robbers Island, 53°13′N, 132°02′W, shore rocks on beach, 1967, *Brodo* 11148 *& Shchepanek*; *ibid.*, Skidegate Inlet, Balch Island (North), 53°14′N, 132°05′W, stone at edge of beach, 1967, *Brodo* 11527 *& Shchepanek*; *ibid.*, Graham Island, Seal Inlet in Rennell Sound, 53°29′N, 132°47′W, rock at shore, 1967, *Brodo* 10313 *& Shchepanek*; *ibid.*, Graham Island, Skidegate Landing at Haida Point, 53°15′N, 132°01′W, shoreline rock in lower aerohaline, *Brodo* 11716 *& Shchepanek*; *ibid.*, Graham Island, Cone Head, SW corner of Rennell Sound, 53°23′N, 132°39′W, upper hygrohaline, 1988, *Brodo* 26941; *ibid.*, E coast of Moresby Island, N side of Gray Bay, 53°08′N, 131°47′W, shoreline rocks, aerohaline, 1967, *Brodo* 12603; *ibid.*, Moresby Island, N shore of Copper Bay, 53°11′N, 131°46′W, on rock of upper hygrohaline, 1971, *Brodo* 17260; *ibid.*, Moresby Island, 1/2 mile W of Ikeda Point, 52°19′N, 131°09′W, shoreline rocks, lower aerohaline, 1971, *Brodo* 17577; *ibid.*, Moresby Island, Cumshewa Head, 53°02′N, 131°36′W, upper surface of headland rocks, 1971, *Brodo* 17427 *& Wong*; *ibid.*, Moresby Island, Ingraham Point, entrance to Carpenter Bay, S shore, 52°14′N, 131°02′W, exposed headland rocks, 2000, *Brodo* 29962B; *ibid.*, Kunghit Island, foot of Balcom Inlet, 52°06′N, 131°01′W, rock outcrop at shoreline in aerohaline, 1967, *Brodo* 12372 *& Shchepanek*; *ibid.*, Cape St James, 51°56′N, 131°01′W, on rock at edge of ridge, 1971, *Brodo* 17618, *Wong & Schofield*; *ibid.*, Limestone Island, Boat Cove, 52°54′N, 131°36′W, on shoreline rocks, *c.* 2 m, above *Fucus* limit, 2000, *Brodo* 30120A *& Brodo*; *ibid.*, Langara Island, 54°12′N, 133°00′W, rocks in lower aerohaline, 1967, *Brodo* 10598; *ibid.*, Huxley Island, N shore, 52°28′N, 131°21′W, rock in hygrohaline, 1971, *Brodo* 17538, *Wong & Schofield*; *ibid.*, Wathus Island, in Masset Inlet, 53°40′N, 132°29′W, southern point of land, shoreline rock, aerohaline, 1971, *Brodo* 18341A, *Wong & Turner*; *ibid.*, Chaatl Island, cove on W coast, 53°08′N, 132°35′W, rock wall on headland, lower aerohaline, 1988, *Brodo* 26973, *Brodo*, *Sharnoff & Sharnoff*; Vancouver Island, Bamfield Marine Station, rocky point immediately N of station, 48°70′N, 125°10′W, 1976, *Benton & Richardson*.—**USA:** *Alaska*: Juneau, Shrine of Ste.-Therese, 10 miles W of Auke Bay, 58°28′N, 134°47′W, lower aerohaline, 1988, *Brodo* 26145 (CANL); Sitka, Starrigavan Bay, near campground, 57°08′N, 135°22′W, protected headland rocks, aerohaline, 1988, *Brodo* 26075 (CANL); Sitka harbour, saxicolous, 2010, *Spribille* s. n. (GZU); Hoonah-Angoon Census Area, Glacier Bay National Park, Taylor Bay, 58.25428°N, 136.56633°W, 0–5 m, on hornblende augen gneiss, in splash zone, 2012, *Fryday* 10389 (MSC, fertile); *ibid.*, *Fryday* 10408 (MSC); *ibid*., *Spribille* 39586, 39588, 39589 (MSC); Icy Straits, Fern Harbor, 58.30938°N, 136.45254°W, seashore crags, 0–4 m, 2012, *Spribille* 38205 (MSC; sub *Physcia caesia*).

### Thelocarpon immersum Fryday sp. nov.

MycoBank No.: MB 830120

Separated from all other species of the genus by the combination of immersed perithecia, the presence of paraphyses, and the globose ascospores.

Type: USA, Alaska, Hoonah-Angoon Census Area, Glacier Bay National Park, park entrance, 58.45281°N, 135.77898°W, 16 m, on soil in calcareous wet meadow in glacial outwash plain, 1 July 2012, *Spribille* 37899, *Pérez-Ortega & Tønsberg* (MSC—holotype).

(Fig. 38)

**Fig. 38.**

*Thelocarpon immersum* (holotype). A & B, habitus (arrows indicate ascomata); C & D, section through ascoma under brightfield (C) and polarized (D) light; E, ascospores, in KOH. Scales: A = 1 mm; B = 100 μm; C & D = 50 μm; E = 10 μm.

*Thallus* absent, although the perithecia are associated with various cyanobacteria and chlorococcoid algae.

*Ascomata* perithecioid, yellow-green, 0.08‒0.12 mm diam., embedded in a mat of cyanobacteria and chlorococcoid algae, with only the tips protruding. *Excipulum* hyaline except upper 100 μm which is yellow-brown; *periphyses* present, simple, 30–40 μm long. *Hymenium* I+ orange-brown; *paraphyses* long (80–100 μm), narrow (1 μm wide), sparingly branched and anastomosing. *Ascus* initially cylindrical 75‒90 × 15‒17 μm with the upper 12‒15 μm narrowed to 5‒7 μm wide, later becoming clavate and up to 20 μm wide; *wall* I+ orange-brown, contents I+ yellow, tholus absent; *ascospores* globose, 5‒7 μm diam., I+ yellow.

*Conidiomata* not observed.

#### Chemistry

All spot tests negative.

#### Etymology

The name refers to the immersed ascomata.

#### Habitat

Known only from the holotype collection, which occurred on a biofilm over calcareous soil in a glacial outwash plain.

#### Notes

*Thelocarpon* is a genus of small, inconspicuous species that occur on various substrata, usually in damp habitats. Eight species have been reported from North America, of which only one, *T. sphaerosporum* H. Magn., has globose ascospores. The species are morphologically and anatomically varied and attempts have been made in the past to subdivide the genus. To date, DNA sequence data is available for only three species.

The family *Thelocarpaceae* was first monographed by Magnusson (1935), who recognized 11 species in *Thelocarpon* Nyl. and two in *Thelococcum* Nyl., the latter genus having immersed rather than sessile ascomata. As our new species has immersed ascomata, Magnusson would have included it in *Thelococcum* but the two species he included in that genus both have larger, ellipsoid ascospores. Among the species Magnusson included in *Thelocarpon*, only *T. sphaerosporum* has globose ascospores but that species, in addition to having sessile ascomata, also has an exposed hymenium.

Salisbury (1966) also monographed the genus and recognized 13 species. He included the species with immersed ascomata in *Thelocarpon* but removed the species with an exposed hymenium (including *T. sphaerosporum*) to *Ahlesia* Fuckel. Of the species he retained in *Thelocarpon*, only *T. coccosporum* Lett. has globose ascospores. However, that species, unlike our new species, has sessile ascomata and lacks paraphyses.

Poelt & Hafellner (1975) reviewed the varying characters of the genus, including relative immersion of ascomata, exposure of hymenium, development of ascus apical apparatus and presence/structure of paraphyses. They concluded that subdividing the genus using any one character would bring different results and therefore accepted a wide circumscription of the genus that included the species previously included in *Ahlesia* and *Thelococcum*. Subsequently, Poelt & Vězda (1977), in their key to the species of *Thelocarpon* in Europe, included 18 species but the only species with globose ascospores were *T. sphaerosporum* and *T. coccosporum.* In more recent studies, Orange *et al.* (2009*a*) included 15 species from the British Isles but only *T. sphaerosporum* with globose ascospores, whereas only two species, neither with globose ascospores, were included from the Greater Sonoran Desert (Knudsen & Lumbsch 2007).

### Toensbergia blastidiata T. Sprib. & Tønsberg sp. nov.

MycoBank No.: MB 830122

Resembling *Toensbergia leucococca* in the corticolous habit, the cream-coloured areoles and the production of alectorialic acid, but distinct in being blastidiate (sorediate in *T. leucococca*) and in sometimes forming thick and more or less continuous thalli to a decimetre or more wide (unlike the persistently discrete squamules of *T. leucococca*).

Type: USA, Alaska, Glacier Bay National Park and Reserve, base of Marble Mtn at Whidbey Passage, 58°37.90′N, 136°14.68′W, 3–10 m, corticolous on *Alnus viridis* subsp. *crispa* just uphill from beach, 3 July 2012, *Tønsberg* 41670 *& Spribille* (MSC—holotype; NY—isotype).

(Fig. 39)

*Thallus* corticolous, crustose, episubstratal, areolate, blastidiate, cream-coloured and in herbarium specimens becoming rose (due to the presence of alectorialic acid); *areoles* initially solitary, rounded, convex, usually constricted below, to about 0.3 mm wide, soon starting to produce blastidia and then becoming larger, irregular, and often confluent with adjacent areoles that in some well-developed specimens, such as in parts of the type collection, result in patches to 1 dm or more wide that may crack and form angular, irregular, flat ‘secondary areoles’ to 0.8 mm high and 2 mm or more wide (Fig. 39C); surfaces of areoles blastidiate almost from the beginning, minutely coralloid when viewed under the dissecting microscope. *Blastidia* globose to opuntioid, to 60 μm wide, lined with a layer of rounded fungal cells, 5–7 × 5–6 μm, sometimes dotted blue, the pigment probably due to the presence of hypothallus hyphae that have been lifted upwards with the growth of the areoles. *Medullary layer* not observed. *Hypothallus* usually distinct, thin, not felty, usually bluish grey, sometimes bluish black or blackish, visible between areoles and along thallus margin. *Photobiont* chlorococcoid, cells ± globose, to 15 μm diam.

**Fig. 39.**

*Toensbergia blastidiata* (holotype). A–C, habitus, thallus with dispersed (A & B) and confluent (C) blastidia; D–F, details of blastidia with SEM. Scales: A = 1 mm; B, C & E = 0.5 mm; D = 200 μm; F = 50 μm.

*Ascomata* and *pycnidia* not seen.

#### Chemistry

Thallus PD+ yellow, C+ red; alectorialic acid with satellite(s) detected by TLC.

#### Etymology

For the blastidiate thallus surface.

#### Habitat

On bark of *Alnus viridis* subsp. *crispa*, *A. incana* subsp. *tenuifolia*, *A. rubra* Bong., *Frangula purshiana* (DC.) A. Gray, *Malus fusca* (Raf.) C. K. Schneid. and *Pinus contorta*. Apparently widespread in coastal NW North America from Kodiak Island in south-central Alaska to the Olympic Mountains of Washington State.

#### Notes

This is a sterile sorediate crust (Fig. 39) containing dominant alectorialic acid forming creamish patches on alder trunks. *Toensbergia blastidiata* may be superficially similar to *Fuscidea muskeg*, especially when old and pink. This is a sterile crust and is easily distinguished when the latter is fertile. Multilocus rDNA sequencing reveals that *T. blastidiata* is closest to *Pertusaria geminipara* (Th. Fr.) C. Knight ex Brodo, a species that we had previously sequenced for a study on evolution within Ostropomycetidae (Resl *et al*. 2015) and ultimately excluded because it was too different from other *Pertusaria* species. A phylogenetic analysis shows that DNA sequences from the new species and *P. geminipara*, both of which contain alectorialic acid, form a strongly supported clade (Fig. 5) with the genera *Toensbergia* Bendiksby & Timdal (which likewise contains alectorialic acid) and *Sporastatia* A. Massal. (which contains gyrophoric acid). According to the results of Bendiksby & Timdal (2013), *Toensbergia* and *Sporastatia* form a group sister to the rest of the subclass Lecanoromycetidae, a result we also obtained. The DNA sampling of *Sporastatiaceae* has not been extensive and we await more sequenced loci before further speculating on the phylogenetic relationships within this group. In the meantime, we propose moving *Pertusaria geminipara* into an expanded *Toensbergia* (see below). It is the first fertile species to be included in *Toensbergia*; its ascomatal anatomy is described and illustrated by Brodo (1984*a*).

#### Additional specimens examined (*T. blastidiata*)

**Canada:** *British Columbia*: Vancouver Island, Pacific Rim National Park Reserve, Radar Hill, near summit, 49°05.060′N, 125°50.501′W, 100 m, corticolous on *Alnus rubra*, 2010, *Tønsberg* 40799 (BG).—**USA:** *Alaska*: Hoonah-Angoon Census Area, near Gustavus, vicinity of Bear Track Lodge N of Gustavus airport, 6.5 km NE of Gustavus, along Falls Creek Road, 58.43792°N, 135.63803°W, 30 m, corticolous on *Malus fusca*, 2012, *Spribille* 39061 (MSC); *ibid*., Glacier Bay National Park, Glacier Bay, S Sandy Cove, 58.71026°N, 135.97581°W, sea level, corticolous on *Alnus*, 2014, *Spribille* 40737 (MSC); *ibid*., base of Marble Mtn at Whidbey Passage, 58°37.90′N, 136°14.68′W, 3–10 m, corticolous on *Alnus viridis* subsp. *crispa* uphill from beach, 2012, *Tønsberg* 41671, 41673 (MSC–topotypes); Borough of Sitka, Baranof Island, *c*. 10 km E (direct) of Sitka, off the main road from Sitka to Herring Cove (Sawmill Creek Rd) at Alaska Pulp Corporation pulp mill, 2 miles along road to Blue Lake, along road to Beaver Lake, 60 m, corticolous on *Alnus rubra*, 1991, *Tønsberg* 16347 (BG); Old Sitka, E of Starrigavan Bay, along the beach N of the mouth of Starrigavan River, 57°07.942′N, 135°22.221′W, 0–10 m, corticolous on twig of *Alnus* (shrub), 1991, *Tønsberg* 16455 (BG); Kodiak Island Borough, Kodiak Island E, along road to Anton Larsen Bay, bank of Red Cloud River, 57°49′N, 152°37′W, 20 m, corticolous on trunk of *Alnus*, 1991, *Tønsberg* 15245 (BG); Petersburg Borough, Mitkof Island, SE of Petersburg, inland from Sandy Beach [along road to Frederic Point], 56°48.2′N, 132°54.7′W, 10–20 m, corticolous on branches/twigs of *Pinus contorta* in muskeg, 2001, *Tønsberg* 30322b (BG); Borough of Juneau, Douglas Island E, West Juneau, 0.5 miles along Douglas Hwy NW of Juneau-Douglas Bridge, 58°18′N, 134°27′W, 10 m, on trunk of *Alnus rubra*, 1991, *Tønsberg* 16132 (BG). *Washington*: Clallam Co., Olympic Peninsula, Olympic National Park, just N of Ozette Lake, W bank of Ozette River, 48°09′20″N, 124°40′10″W, 10 m, corticolous on *Rhamnus purshiana* [ = *Frangula p.*], 1993, *Tønsberg* 19448 (BG); *ibid*., Ozette Lake, north end, Deer Point, 48°08.2′N, 124°38.2′W, 10 m, on *Alnus rubra* on lakeshore, 1999, *Tønsberg* 27113 (BG).

### Toensbergia geminipara (Th. Fr.) T. Sprib. & Resl comb. nov.

MycoBank No.: MB 830124

Basionym: *Lecanora geminipara* Th. Fr., *Lich. Scand.* (*Upsaliae*) **1**(1), 236 (1871). —*Pertusaria geminipara* (Th. Fr.) C. Knight ex Brodo, *Bryologist* **87**, 105 (1984); type: Norway, Telemark, Tinn, Gusta-fjell, 1856, *Th. M. Fries* (UPS-L-150351, syntype!).

### Xenonectriella nephromatis Pérez-Ort. sp. nov.

MycoBank No.: MB 830125

A *Xenonectriella* species characterized by the size of the asci (120–130 × 8–12 μm), ascospores (12–16 × 5–8 μm) and the host (*Nephroma* cf. *bellum*).

Type: USA, Alaska, Hoonah-Angoon Census Area, Glacier Bay National Park, near end of Geikie Inlet, NE shore, 58°36.421′N, 136°30.970′W, just above sea level, on *Nephroma* cf. *bellum* over *Alnus*, 7 September 2011, *Spribille* 36426 (US—holotype).

(Fig. 40)

**Fig. 40.**

*Xenonectriella nephromatis* (holotype). A, ascomata; B, detail of ascomata; C, transverse section of a perithecium; D, detail of periphyses; E, detail of perithecial wall; F, ascus; G & H, ascospores (C–H in water, using DIC microscopy). Scales: A = 1 mm; B = 250 μm; C = 100 μm; D = 25 μm; E & F = 10 μm; G & H = 2.5 μm.

*Ascomata* perithecioid, dispersed, immersed in host thallus, erumpent, dark red, subspherical to obpyriform, to 600 μm diam., without hairs. *Ascomata wall* dark reddish orange, KOH+ quickly violet-purple, without orange oil guttules, composed of several layers of cells; *outer layer* dark reddish orange, to 25 μm wide, composed of several layers of enlarged prismatic cells, 10–18 × 2–4 μm; *inner part* hyaline, to 20 μm wide, composed of several layers of flattened cells. *Periphyses* abundant around the ostiole, forming a protruding cone, thin, 15–25 × 1.5–2.5 μm. *Interascal elements* not observed in mature specimens. *Asci* unitunicate, ±cylindrical with a thin wall, widened at the apex, with obtuse apex, 8-spored, 120–130 × 8–12 μm; *ascospores* uniseriately arranged inside the ascus, broadly ellipsoid but quite variable in shape, 1-septate, hyaline, not or slightly constricted at the septum, apices rounded or obtuse, ornamented, 12–16 × 5–8 μm (*n* = 24).

#### Etymology

Named for its occurrence on *Nephroma*.

#### Habitat

On *Nephroma* cf. *bellum* (thallus in poor condition).

#### Notes

The genus *Xenonectriella* was introduced by Weese (1919) for the species *X. lutescens* (Arnold) Weese, which is lichenicolous on *Solorina* spp. It was resurrected by Rossman *et al.* (1999) for species of the order *Hypocreales* similar to *Pronectria* Clem. with dark-coloured perithecial walls reacting KOH+ and lactic acid+ dark purple-red, with long cylindrical asci and uniseriate, ornamented, usually light brown, ascospores. The genus *Cosmospora* Rabenh. is similar to *Xenonectriella* and it is likely that species assigned to these genera are congeneric. However, we follow Etayo & Sancho (2008) who maintain the difference between the genera based on the lifestyle of *Xenonectriella*, which is only lichenicolous with the development of the ascomata always inside the host thallus, whereas *Cosmospora* is fungicolous, saprotrophic or lichenicolous (one species) and usually possesses sessile ascomata.

*Xenonectriella nephromatis* (Fig. 40) is similar to *X. rosea* Etayo and *X. ornamentata* (D. Hawksw.) Rossman in the colour of the ascomata, although *X. rosea* may possess pinkish perithecia (Rossman *et al.* 1999; Etayo & Sancho 2008). *Xenonectriella rosea* has slightly smaller ascospores (9.5–12.5 × 6–7.5 μm) with verrucose ornamentation and occurs on *Pannaria leproloma* (Nyl.) P. M. Jørg. and *Psoroma* spp. whereas *X. ornamentata* has 4-spored asci with much larger ascospores (25–31 × 7–9 μm). *Xenonectriella aurantiaca* Etayo from Tierra del Fuego (Etayo & Sancho 2008) has similarly sized ascospores but its ascomata are clearly orange and it grows on *Pseudocyphellaria vaccina* (Mont.) Malme. So far, *X. nephromatis* is known only from the type collection growing together with *Tremella nephromatis* Diederich.

## Other Species Treated in Detail

### Absconditella rosea Kalb & Aptroot

MycoBank No.: MB 824111

*Bryologist* **5**, 57 (2018); type: Venezuela, Merida, Rangel, Aug. 1989, *K. Kalb, A. Kalb & M. López-Figueiras* 25745 (VEN—holotype, n.v.; isotype from hb. Kalb, studied by Z. Palice).

**Fig. 41.**

*Absconditella rosea* (*Spribill*e 39168). A & B, habitus; C, section of apothecium and thallus with fragments of bryophyte material, in Lugol's solution; D, ascospores, asci and paraphyses, in Lugol's solution after pretreatment with K. Scales: A = 1 mm; B = 100 μm; C = 50 μm; D = 10 μm.

(Fig. 41)

*Thallus* filmy, greenish, covering mosses and to a lesser extent plant detritus, in section essentially a biofilm, with spreading fungal hyphae, bacterial colonies and roundish algal cells 6–12 μm diam.

*Ascomata* apothecia, round, (0.13–)0.22(–0.35) mm diam. (*n* = 12), single to clustered in small groups, strongly globose when young, opening with a small pore, eventually disc exposed but remaining highly concave when dry; *disc* deep pink to orange-pink, matt; *proper margin* pale whitish pink. *Excipulum c*. 50 μm wide laterally, 40 μm wide basally, composed of structured, interwoven, small fungal filaments *c.* 1.5 μm wide (±prosoplectenchymatous). *Hymenium* c. 75–85 μm tall, hyaline, weakly I+ golden; *paraphyses* separating easily in water, *c*. 0.5 μm wide, with little structural strength and easily breaking or becoming ‘wavy’ under pressure of microscope cover slip, widened to *c.* 1.2 μm apically. *Hypothecium c*. 30 μm high, hyaline. *Asci* 8-spored, narrowly cylindrical, non-amyloid, *c*. 55–85 × 6–7 μm [sic]; *ascospores* narrowly ellipsoid to ellipsoid, the majority 3-septate, a few 2-septate or even 1-septate, often with slightly pointed ends, occasionally ± papillate-warted (residual ascoplasma?), (12–)16.3(–21) × (4–)4.9(–6) μm (*n* = 30).

#### Chemistry

All spot tests negative.

#### Habitat

On bryophytes in meadows in the uppermost beach zone.

#### Notes

In earlier versions of this manuscript, we treated this as a new species provisionally called *Absconditella rosea*, until Kalb & Aptroot (2018) published a taxon from páramo habitats in Venezuela with exactly that name. The GLBA specimens shared with the description of the Venezuelan material the pink to orange-pink colour of the apothecia, deeply concave apothecial discs and 3-septate ascospores, and initially we thought our Alaskan material differed in ascospore length. We measured 12–21 × 4–6 μm in the GLBA specimens, with an average length of 16.25 μm, while Kalb & Aptroot (2018) reported ascospores measuring 11–13 × 5.5–6.5 μm in *A. rosea*. However, Zdeněk Palice (personal communication) measured a range of ascospore sizes from an isotype specimen of *A. rosea* (15–21.5 × (4.5)5–5.5(–6) μm (*n* = 10)) that strongly overlap with the range measured in GLBA specimens. Similarly, apothecial diameters were larger than reported in the protologue, with some apothecia measuring up to 0.45 mm. As a result of these discrepancies, we provide a full description of the Alaskan specimens. Despite the long distance between the two localities, we can find no justification for distinguishing the Alaskan material as a separate species from *A. rosea* at this time.

Baloch *et al.* (2010), in their phylogenetic revision of *Ostropales*, showed that the type of *Absconditella* Vězda, *A. sphagnorum* Vězda & Poelt, as well as several other species, are closely related to the non-lichenized genera *Bryodiscus* B. Hein *et al*. and *Sphaeropezia* Sacc. One of the most commonly collected species, *A. lignicola* Vězda & Pišút, is however more closely related to the genus *Cryptodiscus* Corda. In the current literature, *Absconditella rosea* may key to *A. lignicola* on account of its 3-septate ascospores, but in habit it more closely resembles the type of the genus, *A. sphagnorum*. DNA sequences were already obtained from *A. rosea* and published by Resl *et al.* (2015) under the name ‘*Absconditella* sp. *Spribille* 39168’. Analysis of *A. rosea* in a set of all available published *Absconditella* sequences shows it belongs to a clade including *A. sphagnorum* with strong support (Fig. 7). Our phylogeny includes all currently available, named *Absconditella* species from GenBank that seemed logical to include (*A. rubra* van den Boom *et al.* is represented in GenBank only by ITS sequences, which are absent from several other species so would not be useful for inferring relationships). Our phylogeny shows more clearly than a previous study (Aptroot *et al*. 2014) that *Absconditella* and *Geisleria* Nitschke are likely congeneric, assuming that a specimen from Sweden labelled *Absconditella* sp. 2 by Baloch *et al*. (2010) is correctly placed in *Absconditella*. We refrain from making any changes to the nomenclature, however, as we have not sampled *Absconditella* with the intention of testing the *Geisleria-Absconditella* hypothesis, and many species remain unsampled. We note, that *Geisleria* is the older name.

#### Specimens examined

**USA:** *Alaska*: Hoonah-Angoon Census Area, Glacier Bay National Park, along shoreline N of Point Gustavus, 58.40633°N, 135.90598°W, 2–4 m, on bryophytes on upper beach, 2012, *Spribill*e 39168 *& Svensson* (MSC), *Spribille* 39165 (MSC, sub *Gyalideopsis muscicola*), *Svensson* 2769 (MSC).

### Lecanora alaskensis H. Magn.

MycoBank No.: MB 410846

*Annals Cryptog. Exot.* **5**, 19 (1932); type: USA, Alaska, Wrangell, on slate beach, 1900, *A. S. Foster* s. n. (UPS L-74656—holotype!).

(Fig. 42)

**Fig. 42.**

*Lecanora alaskensis*. A & B, habitus; C & D, section through apothecium under brightfield (C) and polarized (D) light; E, asci containing immature ascospores, in Lugol's solution; F, thallus granule, with SEM; G, surface of thallus granule with SEM, showing extrusion of wax-like fibrils. Scales: A = 1 mm; B = 200 μm; C, D & F = 100 μm; E = 10 μm; G = 1 μm. A, E, F & G from *Dillman* 714a (TNFS); B–D from *Tønsberg* 41794 (BG).

*Thallus* crustose, to 4.5 cm across, rugulose to minutely bullate or scurfy, usnic yellow to ochre-usnic yellow or pale latte brown (dark greenish in type); *areoles* distinct to indistinct, 0.15–0.5 mm diam., strongly convex; internally undifferentiated; *medulla* hydrophobic, perhaps on account of wax-like extrusions (Fig. 42G); *hypothallus* not seen. *Photobiont* chlorococcoid, cells globose, 8.5–11 μm diam.

*Ascomata* apothecia, round, globose to strongly tuberculate, (0.5–)0.9–1.6(–3.9) mm diam.; *disc* strongly convex, reddish brown to medium brown (to black), matt; *margin* lecanorine, pale ochre-grey, quickly becoming excluded. *Excipulum* to 70 μm wide laterally, 45–100 μm basally, of radiating, thick-walled hyphae, I−, filled or streaked with POL+ crystals. *Hymenium* 60–80 μm tall, hyaline to hazy golden brown, I+ blue both before and after treatment with KOH; uppermost part of hymenium inspersed with POL+ granules, red-brown to golden brown in transmitted light; *paraphyses* mostly simple, thin, *c*. 1–1.5 μm wide medianly and 2 μm wide terminally; *subhymenium* slightly darker, KOH+ golden, I− except top of hypothecium including ascogenous hyphae sometimes I+ bluish. *Hypothecium* variable, 160–500 μm tall, hazy yellow-brown to golden brown, pale brown or ochre, composed of thick-walled hyphae with narrow lumina, including many grana. *Asci* 8-spored, *Lecanora*-type, 40–60 × 9–14(–18); *ascospores* simple, narrowly ellipsoid, (10–)12.1–13.4(–17) × (3.7–)4.0–4.7(–6) μm (*n* = 45, four specimens used).

*Pycnidia* seen once, 110–170 μm diam., dark brown; *conidia* filiform, falcate, *c*. 15 × 0.5 μm.

#### Chemistry

Usnic acid, zeorin, thiophanic acid (major constituents), unidentified xanthone (aff. arthothelin), expallens unknown.

#### Habitat

On siliceous rocks just above the high tide line; currently known only from SE Alaska.

#### Notes

*Lecanora alaskensis* was described by Magnusson (1932) and until now has been reported only from the type specimen, collected by A. S. Foster at Wrangell, Alaska. The same specimen was originally reported as *Lecidea flexuosa* Fr. ( = *Trapeliopsis flexuosa* (Fr.) Coppins & P. James) by Herre (1919). We did not immediately recognize the conspecificity of the new material reported here and the type of *L. alaskensis* because the type is strikingly dark green, the apothecia have a persistent, somewhat beaded amphithecium, and the discs are dark brown to black. This may be an artifact of shade or other modifying factors (bird excrement) that occasionally darken thalli in supralittoral habitats. Analysis by TLC confirmed the highly distinctive chemical profile of both the type and the recently collected material, including one specimen from GLBA. Remarkably, this secondary metabolite profile (usnic acid, zeorin and a series of xanthones) matches that of *L. expallens* Ach., typically an epiphytic species. Unlike *L. expallens*, which is fully leprose, *L. alaskensis* does not produce asexual thalline propagules and is richly fertile. It also overlaps with the chemistry of *L. atrosulphurea*, (discussed under the description of *L. viridipruinosa* earlier in this paper), though that species always has black apothecial discs with a greenish pigment (probably Cinereorufa-green), and the thallus sometimes contains norstictic acid (Edwards *et al.* 2009). We obtained a single mitochondrial SSU DNA sequence (Table 1) but this is not informative enough to place it in a specific clade within the broader genus *Lecanora*. Although a supralittoral species, *L. alaskensis* does not correspond to any taxon discussed by Brodo (2010), most of which probably belong in the genus *Myriolecis* (Zhao *et al.* 2015; more recently treated as *Polyozosia*, see Kondratyuk *et al*. (2019)).

#### Specimens examined

**USA:** *Alaska*: Tongass National Forest, Kupreanof Island, north shore, 57°5′N, 133°50′W, on rocks at high tide line, 1994, *K. Dillman* 714 (TNFS); Frederick Sound, NE of Petersburg, 56°N, 133°W, growing on rocks at high tide line, 15 v 1994, *K. Dillman* s. n. (TNFS L-3370); Kupreanof Island, Lindenberg Peninsula, N of Portage Bay, mouth of Todahl Creek, 59°N, 133.17°W [sic; incorrect coordinates], 0 m, growing on rock outcrop above high tide line, *K. Dillman* 1182 (TNFS-3345); Hessa Island, S Prince of Wales Wilderness, N of Douglass and Seagull Islands, 54.77214°N, 131.3244°W [sic; incorrect coordinates], 100 m, on rock outcrops in muskeg, 1999, *K. Dillman* 1999-11 (MSC); Etolin Island, west side, S of King George Bay along beach fringe rocks near large waterfall, 56.2908°N, 132.5758°W, 2 m, schist in salt spray zone, 25 vii 2005, *L. Geiser* s. n. (MSC); Hoonah-Angoon Census Area, Glacier Bay National Park, Fern Harbor area, 58.3100°N, 136.4533°W, 5–15 m, saxicolous on top of boulder under overhanging rock wall (but probably subjected to direct rain), 2012, *Tønsberg* 41794 (MSC).

### Lecanora leptacina Sommerf.

MycoBank No.: MB 388574

*Supplementum Florae Lapponiae* (Oslo), 96 (1826).—*Lecanora varia* var. *leptacina* (Sommerf.) Leight., *Lich.-Fl. Great Brit.*, Edn 3, 177 (1879).—*Lecanora intricata* var. *leptacina* (Sommerf.) Stizenb., *Ber. Tät. St. Gall. Naturw. Ges.*, 371 (1882) [1880–81]; type: Norway, Nordland, Saltdal, in Andraeis alpinum summor[um], Aug. 1824, *S. C. Sommerfelt* s. n. (O-L-000411—holotype!); TLC: isousnic acid, usnic acid, zeorin by TLC).

This is a characteristic species found on *Andreaea* spp. on acidic rock and boulders in open, more or less oceanic heaths. It was originally described by Sommerfelt (1826) from Saltdal, Norway. The chemistry of the species appears to be somewhat shrouded in confusion. According to Foucard (2001) and Edwards *et al.* (2009), the chemical constituents in *Lecanora leptacina* include psoromic acid, conpsoromic acid and atranorin. However, the type specimen contains usnic acid, isousnic acid and zeorin. Specimens from coastal Norway are concordant with the type specimen in lacking psoromic acid, but may contain small amounts of atranorin. Whether or not material treated as *L. leptacina* in Scandinavia and the British Isles represents more than one species or only chemotypes is uncertain.

*Lecanora leptacina* accounts for the ‘known unknown’ species *Lecanora* sp. S26813 from KLGO (Spribille *et al.* 2010). The chemistry reported there includes miriquidic acid, but this was almost certainly an artifact of the inclusion of a small fragment of *Miriquidica gyrizans* (with which it is admixed on the specimen) in the TLC assay. In North America, *L. leptacina* is otherwise known from previous reports from Alaska (the first being Murray & Murray 1978), as well as from Mt Katahdin, Maine (Fryday 2006).

*Lecanora leptacina* was regarded as a variety of *Lecanora varia* and *Lecanora intricata* by 19th century authors (see synonymy). Our phylogenetic analysis has a limited taxon sample but recovers *L. leptacina* on an isolated branch between the *Lecanora polytropa* group and the clade that includes *Myriolecis*, *Protoparmeliopsis* and *Rhizoplaca* (Fig. 10).

#### Specimens examined

**Norway:** *Nord-Trøndelag*: Meråker, N-facing slope of Steinfjellet, 63.3263°N, 12.0095°E, muscicolous on *Andreaea* mosses on boulder in low alpine heath, 640 m, 2013, *Holien* 14257 (TRH L-16109); Namdalseid, between Kjerringklumpen and Tverrelva, 64.1331°N, 10.9186°E, muscicolous on *Andreaea* growing on boulder in low alpine heath, 505 m, 2009, *Holien* 12403 (TRH L-12988); Steinkjer, SE of Mokk, Litlklumpen, 63.955°N, 12.1331°E, muscicolous on mossy boulder in low alpine heath, 590 m, 2009, *Holien* 12382 (TRH L-13004).—**USA:** *Alaska*: Hoonah-Angoon Census Area, Glacier Bay National Park, Dundas Bay, rock outcrops on alpine ridge, 58.3422°N, 136.4002°W, 435 m, 2012, *Fryday* 10166 (MSC).

## Lepra subvelata (G. K. Merr.) T. Sprib. and similar taxa

**Fig. 43.**
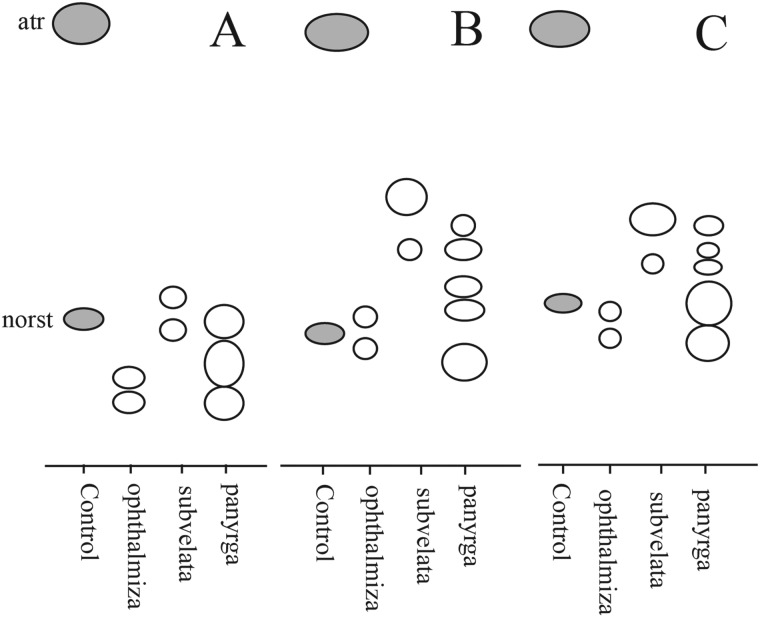
Thin-layer chromatography spot patterns in solvent systems A, B and C for fatty acids in *Lepra ophthalmiza* (from holotype, H), *L. subvelata* (from isotype, CANL) and *L. panyrga* (from Greenland: *Poelt & Ullrich* s. n., August 1983, GZU).

The basis for North American *Lepra* taxonomy continues to be the *Pertusaria* monograph of Dibben (1980). Dibben contributed observations of ascomatal characteristics but in assessing the chemistry of species now treated as *Lepra* he did not take fatty acids into account; thus, species with rich fatty acid profiles are listed by Dibben as ‘chemical constituents unknown’ or ‘unverified’. Two such species are *Lepra ophthalmiza* and *L. panyrga*. In the study of lichens of KLGO, Spribille *et al.* (2010) reported two distinct chemical strains of *L. ophthalmiza* (as *Pertusaria*), which also appeared to be morphologically distinct. One of these, which they reported as *Pertusaria* sp. TT32951, possessed a characteristic pair of fatty acids identified as nephrosterinic and isonephrosterinic acids, as well as traces of atranorin and chloratranorin (based on analysis of *Spribille* 15403, GZU). The other, reported as *P. ophthalmiza* (Nyl.) Nyl., yielded major myelochroic and isomyelochroic acids and accessory atranorin, an unusual substance in *Pertusariales* (based on analysis of *Spribille* 24747, KLGO and a series of types, see below). We have now had the chance to study, with the help of J. Elix (Canberra), the chemistry of all relevant types and numerous specimens from western North America. The lichen previously called *Pertusaria* sp. TT32951 corresponds to the type of *Pertusaria subvelata*, described by Merrill (1908) from near Skagway, Alaska. Dibben (1980) synonymized *P. subvelata* with *Pertusaria panyrga*. However, the two, differ in their fatty acid profiles. Hanko (1983) characterized the chemistry of a number of specimens of *L. panyrga* from northern Europe and described them as containing the fatty acids 1H (major) and bH in trace amounts. The first substance has not been clarified to our knowledge in any lichen but the second substance has been resolved as (–)-pertusarinic acid (Huneck *et al*. 1986; Shimada *et al.* 1993). Notwithstanding further work needed on the chemistry of *L. panyrga*, all three species, *L. ophthalmiza*, *L. panyrga* and *Pertusaria subvelata* (combined into *Lepra* below), can be easily separated by TLC (Fig. 43).

Wei *et al*. (2017) provided a revised phylogeny of *Lepra* species, including DNA sequences from a GLBA specimen of *Pertusaria subvelata* that we had previously published (Resl *et al*. 2015) as *Lepra subvelata* in their Fig. 1. Contrary to what might be expected given the synonymization by Dibben (1980), the topology they recovered does not support the monophyly of *L. subvelata* and *L. panyrga*. For its part, *P. subvelata* was strongly supported as sister to much of the rest of the genus *Lepra* that excludes the *L. ophthalmiza* group. At the same time, if the vouchers sequenced by Wei *et al*. (2017) were correctly identified, *L. panyrga* may be part of the *L. ophthalmiza* complex, which might include additional species not analyzed here. In any case, Wei *et al*. (2017) did not make the formal combination of *Pertusaria subvelata* into *Lepra*, which is provided below. *Lepra subvelata* and *L. ophthalmiza* occur in the same regions and habitats in western North America but can be distinguished by the prominent, flat-topped fertile warts of *L. subvelata*, typically with sharply demarcated ‘tower’ walls and often an exposed, bluish grey disc, compared to the whitish grey, flattened, pruinose granule-dominated mounds of *L. ophthalmiza*, as outlined in the key below.

### Lepra ophthalmiza (Nyl.) Hafellner

MycoBank No.: MB 818770

*Stapfia* **104**(1), 173 (2016). —*Pertusaria multipuncta* var. *ophthalmiza* Nyl., *Lich. Scand.* (Helsinki), 180 (1861).—*Pertusaria ophthalmiza* (Nyl.) Nyl., *Flora* **48**, 354 (1865); type: Finland, Lapponia kemensis, 1856, *Edwin Nylander* (H-NYL 33726—holotype!; myelochroic acid (major), isomyelochroic acid (major), atranorin (trace) determined by HPLC).

*Pertusaria multipuncta* f. *sphaerulifera* Erichs., *Feddes Repert.* **35**, 386 (1934); type: Canada, British Columbia, Aleza Lake, ad corticem abietis, 24 vii 1931, *V. Kujala* (H—holotype! and isotype!; myelochroic acid [major], isomyelochroic acid [major], atranorin [trace] detected in both specimens by HPLC).

*Pertusaria lecanina* subsp. *nigra* Fink in Hedr., *Mycologia* **26**, 160 (1934).— *P. lecanina* var. *nigra* (Fink) Zahlbr., *Cat. Lich. Univers.* **10**, 454 (1940); type: USA, Montana, [Lake Co.,] Rost Lake, dead yew tree at 3000 ft, 15 July 1901, *W. P. Harris* (MICH—lectotype!, designated by Dibben (1980); myelochroic acid [major], isomyelochroic acid [major], atranorin [trace] determined by HPLC).

### Lepra panyrga (Ach.) Hafellner

MycoBank No.: MB 818771

*Stapfia* **104**(1), 173 (2016). —*Urceolaria panyrga* Ach., *Methodus Lichenum*, Sectio prior (Stockholmiæ): 146, tab. IV, fig. 2 (1803). —*Pertusaria panyrga* (Ach.) A. Massal., *Framm. Lichenogr.*, 53 (1855); type: Lapland, habitat in radicibus et caulibus herbarum prope terram, quos incrustat in Alp., *Wahlenberg* (H-ACH, n.v.).

### Lepra subvelata (G. K. Merr.) T. Sprib. comb. nov.

MycoBank No.: MB 830126

Basionym: *Pertusaria subvelata* G. K. Merr., *Bryologist* **11**, 111 (1908); type: USA, Alaska, on birch bark at Skagway, 4 September 1902, [*G. K. Merrill* s. n.] (FH—holotype, n.v.; CANL—isotype!; nephrosterinic and isonephrosterinic acid by TLC).

## Key to distinguish *Lepra ophthalmiza*, *L. panyrga* and *L. subvelata*

1 Apothecia embedded in soralium-like warts, typically not with corticate, ringed edges; apothecial disc seldom clearly visible, pinkish; myelochroic and isomyelochroic acids as major substances.**L. ophthalmiza**
  Apothecia usually highly raised above thallus, often with ring-like, corticate folds surrounding the wart; apothecial disc often visible, greenish pruinose; myelochroic and isomyelochroic acids absent 2
2(1) With nephrosterinic and isonephrosterinic acids; epiphytic.**L. subvelata**
  With fatty acids 1H (major) and (–)-pertusarinic acid (trace; bH *sensu* Hanko (1983)); terricolous over bryophytes in tundra **L. panyrga**

## Ochrolechia xanthostoma (Sommerf.) K. Schmitz & Lumbsch and similar taxa

Brodo (1988) studied a group of *Pertusaria* species characterized by apothecia with one to several perithecioid ostioles in a wart, thin-walled ascospores and the presence of alectoronic acid, sometimes among other substances, as a secondary metabolite. He transferred them to *Ochrolechia* and recognized two species, *O. rhodoleuca* (Th. Fr.) Brodo from northern Norway and *O. subplicans* from the northern Pacific; the latter with two subspecies, subsp. *subplicans* (Nyl.) Brodo and subsp. *hultenii* (Erichsen) Brodo. He mentioned the existence of an additional taxon, *Pertusaria xanthostoma* (Sommerf.) Fr., which he acknowledged was similar to *O. subplicans* subsp. *hultenii* but possessed different substratum preferences (corticolous and terricolous) and four, rather than eight, ascospores per ascus. Schmitz *et al.* (1994) subsequently transferred *P. xanthostoma* into *Ochrolechia*.

Two lichens from this group are present in GLBA. The first occurs on rock both along seashores and in the alpine zone, and is a good match for *O. subplicans* subsp. *hultenii* (see ‘Catalogue of All Named Taxa Found’). The second occurs on bark and wood and appears to correspond to the species reported by Brodo & McCune (2017) as *O. xanthostoma*. However, we came to suspect it may not belong to *O. xanthostoma* s. str., as it typically produces only one ostiole per wart, as opposed to 2–4 (and up to 8). Resolving whether or not the GLBA corticolous material is conspecific with *O. xanthostoma* requires an assessment of the variability within that species.

*Ochrolechia xanthostoma* was originally described from juniper bark in the area around Bodø, Nordland, Norway by Sommerfelt (1823, as *Porina xanthostoma*; lectotypes in O, see Kukwa 2011). It has been reported from Norway, Iceland and Scotland, as well as North America, Russia, Greenland and Australia (Kukwa 2011). In Europe, the application of the name has been extended to cover material from rock at Ben Lawers, Scotland described by Nylander (1865) as *Lecanora poriniformis* Nyl. (UPS-L-717928, isolectotype!). Like the northern Pacific *O. subplicans* subsp. *hultenii*, *L. poriniformis* was described as having 6–8 ascospores per ascus (though no 8-spored asci were found by Kukwa 2011). *Lecanora poriniformis* was only moved to *Pertusaria* as late as Clauzade & Roux (1985) and later treated as a synonym of *O. xanthostoma* by Kukwa (2011). *Ochrolechia xanthostoma* was first reported from North America by Räsänen (1933) from New Brunswick, but the specimen upon which that was based was shortly thereafter named as the new species *P. rubefacta* Erichsen (Erichsen 1934; Dibben 1980), which does not appear to be closely related. *Pertusaria xanthostoma* was subsequently reported by Dibben (1980) from areas surrounding the hypermaritime Gulf of Alaska and the Aleutian Islands, from British Columbia by Noble *et al*. (1987; no voucher cited), and from British Columbia and Oregon by Brodo & McCune (2017).

We sequenced DNA from multiple specimens from Alaska and the one specimen labelled *O. xanthostoma* from Europe that was fresh enough to obtain DNA from, which however came from rock. We found the GLBA material to be genetically distinct from the European specimen labelled as *O. xanthostoma*, and both together formed a sister group to *O. subplicans* subsp. *hultenii* within a strongly supported, monophyletic *Ochrolechia* (Fig. 6). It is tempting to conclude that these data support the interpretation that GLBA corticolous material is distinct from *O. xanthostoma*. However, while it is true that material from the type locality of *O. xanthostoma* has multiple ostioles per verruca, some corticolous European specimens studied at UPS have 1–2 ostioles per verruca and look similar to those from GLBA. This means that, strictly speaking, we still cannot rule out 1) that the saxicolous European specimen sampled may be a second European species distinct from *O. xanthostoma*, and 2) that the range of variability of the European corticolous material encompasses that of the GLBA corticolous material, and that they are conspecific. We consider the latter scenario unlikely, but no fresh European corticolous material is available to us to directly test this hypothesis; the most recent specimen collected from bark that we could find in Norwegian herbaria was from 1981. For this reason, we refrain from describing the GLBA corticolous material as a new species, and treat it as *Ochrolechia* sp. S38011 (see ‘Known Unknowns’).

Yet another taxon exists that has not previously been discussed in the context of this group, namely *Perforaria minuta* Degel., and while it is likely not relevant to delimiting *O. xanthostoma*, this may be as good of a place as any to take it into account and correct some misinformation about its characters. Degelius (1937) described *P. minuta* from a small specimen collected on Kodiak Island by Eric Hultén. The species was characterized by poriform ascomata and punctiform soralia on a creamish thallus over *Picea* bark. Brodo (1973) placed *P. minuta* as a synonym under *Coccotrema pocillarium* (Cumm.) Brodo, but John Elix studied the chemistry of the soralia and several ascomata from the specimen by HPLC (Spribille *et al.* 2010) and found it to contain alectoronic acid. In keeping with an annotation by Rolf Santesson on the type specimen, Spribille *et al.* validated the combination *Coccotrema minutum* (Degel.) R. Sant. ex T. Sprib. *et al*. and published a photograph of the type. However, the first author (TS) missed some important details. Brodo (1973) stated, based on personal communication from Santesson, that the type possessed cephalodia and the chemistry of *Coccotrema pocillarium* (Cumm.) Brodo (that is, stictic and constictic acids and an unknown). Spribille *et al.* (2010) neglected to notice that the type specimen showed no indication that its chemistry had been tested, other than perhaps via spot test (discoloration in the corner of one thallus fragment). More critically, they failed to notice that the type specimen *lacks* cephalodia (and they were also not noted in Degelius’ detailed protologue, which mentioned only a green alga (‘protococcaceae’)). The lack of cephalodia, in addition to the secondary metabolite profile of the type specimen (alectoronic acid), makes it clear that *P. minuta* does not belong in *Coccotrema* but instead is related to the alectoronic acid-containing species of poriform *Ochrolechia* discussed here. To rectify the taxonomic position of *P. minuta*, we propose to accommodate it in *Ochrolechia*.

### Ochrolechia minuta (Degel.) T. Sprib. comb. nov.

MycoBank No.: MB 830127

*Perforaria minuta* Degel., *Acta Horti Gothoburg.* **12**, 122 (1937).—*Coccotrema minutum* (Degel.) R. Sant. ex T. Sprib. *et al.*, *Bryologist* **113**(3), 449 (2010); type: USA, Alaska, Kodiak Island Borough, ‘ad Kodiak in insula Kodiak in cortice Piceae’, 1932, *E. Hultén* 5030b (UPS—holotype, studied again July 2019).

### Steineropsis alaskana T. Sprib. & Muggia

MycoBank No.: MB 516028

*Bryologist* **113**(3), 454 (2010); type: USA, Skagway Borough, White Pass, 59°37.147′N, 135°09.657′W, 1051 m, on rock in snowbed with late-lying snow, 2008, *Spribille* 26809 *& Pérez-Ortega* (US—holotype; NY—isotype).

(Fig. 44)

**Fig. 44.**
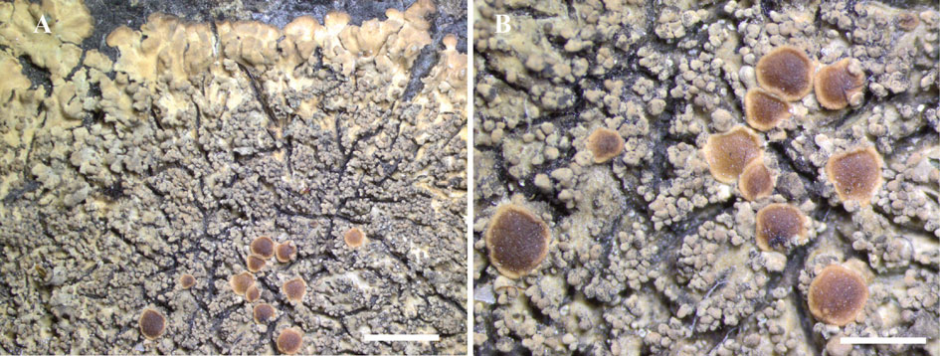
*Steineropsis alaskana* (Mendenhall Glacier, 21 September 2010, *Spribille* s. n., ALTA). A & B, habit of thallus with apothecia. Scales: A = 2 mm; B = 1 mm.

*Apothecia* (Fig. 44) biatorine, 0.8–1.5 mm diam., sessile, slightly convex; *disc* red-brown with a paler proper margin; thalline isidia forming an irregular pseudothalline margin. *Exciple* of radiating hyphae 3–4 μm thick; *cells c.* 10 μm long; cortical cells isodiametric *c*. 4–5 μm across, dilute brown. *Hymenium c*. 150 μm tall, KI−; *paraphyses c.* 1.5–2.0 μm thick, simple or sparingly branched and anastomosing, not or only slightly widening at the apex (to 3 μm); *epihymenium* dilute brown *c*. 10 μm tall. *Hypothecium* hyaline, composed of randomly organized hyphae, dilute brown near base with a dark brown lower edge. *Asci* cylindrical, 60–80 × 12–15 μm, KI+ pale yellow, *Pannaria*-type *sensu* Spribille & Muggia (2013); *ascospores* broadly ellipsoid, 18–20 × 9–11 μm, wall <1 μm thick, not ornamented.

The genus *Steineropsis* T. Sprib. & Muggia was described to accommodate a sterile placodioid cyanolichen from south-eastern Alaska, named *S. alaskana* T. Sprib. & Muggia (Spribille *et al.* 2010). Spribille & Muggia (2013) subsequently presented molecular data to support the position of *Steineropsis* as sister to *Protopannaria* (Gyeln.) P. M. Jørg. & S. Ekman in the *Pannariaceae* (Collematineae) and also mentioned recently discovered fertile material. The ascomata of the species have, however, not been formally described until now.

#### Fertile specimens examined (additional sterile collections listed in ‘Catalogue of All Named Taxa Found’)

**USA:** *Alaska*: near Juneau, Mendenhall Glacier Visitor's Centre, 58°24.985′N, 134°32.692′W, 77 m, abundant, saxicolous on seepy rock, 2010, *Spribille* s. n. (ALTA, GZU); Glacier Bay National Park, Excursion Ridge, ridgetop, 58.46503°N, 135.55757°W, 903 m, 2012, *Spribille* 39437 (MSC); Petersburg Borough, Patterson Glacier, 56.9356°N, 132.6530°W, 130 m, rock outcrop in mature *Alnus* forest, 2015, *Fryday* 11146, 11153, 11155 *& K. Dillman* (MSC).

### Steineropsis laceratula (Hue) T. Sprib. & S. Ekman comb. nov.

MycoBank No.: MB 830128

*Pannaria laceratula* Hue, *Bull. Soc. Bot. Fr.* **48**, LIX (1902) [1901]. —*Fuscopannaria laceratula* (Hue) P. M. Jørg., *J. Hattori Bot. Lab.* **76**, 205 1994); type: Japan, Hakkoda, sur *Betula*, 1200 m, 10 August 1904, *Faurie* 5946 (W—isotype!).

Sequences generated from an epiphytic specimen of *Fuscopannaria laceratula* (USA, Alaska, Tongass National Forest, *c*. 5 km N of Petersburg, Sukoi Islets, on *Picea sitchensis*, *Nash* 43995, BG) alerted Ekman *et al*. (2014) to the evolutionary proximity of this species to *Steineropsis* (see also Lendemer *et al*. 2017). Our three-locus dataset from a rock-dwelling specimen from the outer coast of GLBA confirms this placement (Fig. 9).

The similarity of *S. laceratula* to *S. alaskana* is striking enough that some members of the field expedition assumed, on first impression, that *S. laceratula* was an epiphytic occurrence of the locally more common *S. alaskana*. The two share a characteristic range of cervine brown cortical pigments (illustrated for *S. alaskana* in Spribille *et al.* (2010) and in Fig. 44), and also lack an amyloid apical ring structure in the ascus. *Steineropsis laceratula* was reported to possess such a structure by Jørgensen (2000), but this was lacking in material we studied (e.g. *Brodo* 14150, UPS L-563845). Instead, the ascus compares favourably with that in *S. alaskana* and *Protopannaria* (see Spribille & Muggia 2013: Fig. 3P).

The inclusion of *Fuscopannaria laceratula* in *Steineropsis* expands that genus to now include both an apparently obligately saxicolous species (*S. alaskana*) and a species usually found on tree bark but secondarily also on rock (*S. laceratula*), and extends the distribution of the genus to East Asia.

## Known Unknowns

We treat as ‘known unknowns’ lichens for which we have no names, but which we are convinced are not otherwise accommodated in species included in the study. Some of these may constitute species new to science, and indeed several are well known to us but material or data have not been sufficient for proceeding with a description. In other cases, identification is not currently possible as a worldwide taxonomic treatment for the group in question is lacking, and thus a validly published name possibly already exists.

*Amygdalaria* sp. F10121 — Saxicolous on pebbles on beach ridges and in sparse alpine tundra, 0–922 m. **EX:** 319, S37941; 407, F10121; **WA:** 319, S37941; 105, F9999, F10000; 391, S38637 (sub *Lecanora polytropa*). This species appears to be close to *Amygdalaria consentiens*, differing mainly in the pseudocyphellate pseudothalline margins of the ascomata. We have obtained a single ITS rDNA sequence (Table 1) but are prevented from comparing it in the absence of a more comprehensive sampling of *A. consentiens*, for which we currently have no ITS sequences. Also seen from the Olympic Peninsula of Washington (Clallam Co., 47.8564°N, 123.0359°W, *R. Droker* 16 June 2015-1, MSC).
#*Arthonia* sp. S38095 — Lichenicolous on *Lecanactis*, over bark of *Picea sitchensis*, 124 m. **DUN:** 334, S38095. Ascospores are 1-septate, *c.* 10 × 3 μm with the upper cell slightly larger than the lower.
*Arthonia* sp. S38167 — Corticolous on *Picea sitchensis* twig, 27 m. **DUN:** 339, S38167. This species is similar to *A. arthonioides* in possessing 2–3-septate ascospores, 12–15 × 5–6 μm, but differs in having an I+ blue hymenium, and in the different habitat.
*Arthonia* sp. S38303 — Lignicolous on sheltered, soft lignum of giant stump, 568 m. **EX:** 357, S38303. Botryose apothecia with dark hypothecium and KOH− hymenium, I+ blue after KOH; ascospores not seen. Despite not observing ascospores, no other species in the current survey matches this specimen.
*Arthopyrenia* sp. S38039 — Corticolous on *Alnus*, 33 m. **GUS:** 879, S36809; [V329, S38039, S38055]. This species does not match any species of *Arthopyrenia* known to us. The ascospores are 3-septate and brown, *c.* 16 × 6 μm.
*Arthopyrenia* sp. S39514 — Corticolous on *Alnus* and *Salix*, 10 m. **DUN:** 463, S39514; **WA:** 384, S38506. Following Harris (1975), this species would key to *Arthopyrenia analepta*, but ascospores are narrower and longer (17–18 × 4–5 μm), breaking at the septum. The perithecia tend to be smaller than in GLBA collections of *A. analepta* (not much more than 130 μm diam.) and the perithecium wall is brown, KOH+ paler or brownish, lacking green-black pigments. It is similar to the description of *Sporoschizon petrakianum* Riedl, from Austria (Riedl 1960), which has been treated as a synonym of *Naetrocymbe punctiformis* (Hafellner & Türk 2016). In GLBA this appears to be a distinct species.
*Bacidia* sp. S36806 — Corticolous on *Alnus* and *Salix*, 20 m. **GUS:** 879, S36806 (UPS); [574, T41821]; 547, T41847. This is a member of *Bacidia* s. str. with weak brown pigmentation and minute crystals in the excipulum. Internally it resembles *B. absistens* but the exterior looks different, with small, piebald apothecia that are comparatively more convex.
*Bacidina* sp. A — Lichenicolous over senescent *Peltigera collina*, 60 m. **GUS:** 857, S36164. This corresponds to *Bacidina* sp. A of Spribille *et al.* (2009).
*Bellemerea* sp. F9943 — Saxicolous on granitic rock, 0–30 m. **EA:** just S of terminus of Riggs Glacier, F10651; **WA:** 319, SS37933; 326, S37993 (sub *Rhizocarpon lecanorinum*); 386, S38536; 102, F9928, F9943 (sub *Rhizocarpon lecanorinum*); 103, F9950, S37933, S37940, S37994; 205, M2492. TLC: norstictic and gyrophoric acids. Similar to *B. cinereorufescens* but separated from that species and all other species of the genus by the raised apothecia with a thick thalline margin and an umbonate disc, and the tall hymenium (100–125 μm high). We initially intended to describe this as a new species from GLBA but molecular work in *Bellemerea*, including on GLBA specimens, has shown it to be nested within a more widespread species occurring throughout the western cordillera as far south as Montana (T. Wheeler, personal communication). Specimens with umbonate apothecia have also been seen by us elsewhere (e.g. in Montana; TS).
*Bilimbia* sp. S38926 — Corticolous on *Cupressus nootkatensis* and *Populus balsamifera*, 0–68 m. **DUN:** 413, S38926; **GB:** 874, S36699. Similar to *B. microcarpa* but differing from typical specimens in the thin and immersed thallus, the darker hypothecium, and ascospores with a tendency to have constricted septa.
*Chaenotheca* sp. S38742 — Corticolous in sheltered underhangs of large, leaning *Cupressus nootkatensis*, 237–250 m. **EX:** 401, S38739, S38742, S38743; 446, S39339, S39340. This species possesses a powdery greenish thallus that fades to white in herbarium specimens; the ascomata are borne on stalks which are pale basally, with distinct white pruina on the upper stalk and capitulum. No substances were detected by TLC. An mtSSU sequence was obtained (T1137, Table 1) but is insufficient to place the species at the present time due to the low level of sampling of this locus in the genus.
*Chaenothecopsis* sp. 7 — Lignicolous on snag, 569 m. **EX:** 358, S38326, S38331, S38332. This appears to correspond to *Chaenothecopsis* sp. 7 (‘*viridis*’) of Goward (1999). See also the discussion of this species by Titov (2006; English and German translation of keys by Stordeur *et al.* 2010).
*Chaenothecopsis* sp. S37846 — Lignicolous on snag, 222 m. **EX:** 313, S37846. Capitulum KOH−, appearing lightly pruinose, stalk dark, *c.* 1 mm, with a paraplectenchymatous-type cellular structure; asci *c.* 40–45 × 4 μm, with apical canal at maturity; ascospores 6.5–8.5 × 3–3.5 μm, pigmented lightly brown, smooth, with ± pointed ends. This species is similar in the dimensions of the asci and ascospores to *Chaenothecopsis* sp. 7 (above) but differs in the lack of aeruginose pigments and the presence of distinctive pruina around the excipulum. It also differs from the Chinese *Chaenothecopsis tibellii* Titov in the lack of ascospore ornamentation (see Titov 2006; English and German translation of keys by Stordeur *et al.* 2010).
*Cliostomum* sp. S36738 — Corticolous on *Picea sitchensis* in or immediately behind beach fringe, 2 m. **GUS:** 876, S36738 (sub *C. leprosum*); 341, S38234; 436, S39161. TLC: sphaerophorin. A sterile species consisting of an inconspicuous white thallus with irregular sorediate patches. The species is characterized by the presence of sphaerophorin, an uncommon substance. The genus is speculative.
*Cliostomum* sp. T41758 — Lignicolous on snag, 37–569 m. **DUN:** 563, T41758; **EX:** 108, F10045; [858, S36191]; 358, S38305; [V431, S39054]. An unknown sorediate-leprose species consistently bearing large, black, pyriform pycnidia *c.* 0.25 mm diam.; wall pale brown (KOH yellowish brown); conidia short oblong, 3.5–4.0 × 1.5–2.0 μm. TLC revealed only an unidentified pigment. The genus is speculative and this could alternatively be a species of *Anisomeridium*.
[*Fuscopannaria* sp. T41714b — Corticolous on trunk of *Populus balsamifera*, 16 m. **GUS:** 557, T41714b. This species has elongated lobes and marginal/terminal, bead-like soredia. It could not be identified to genus by P. M. Jørgensen (personal communication, 2013). We place it provisionally in *Fuscopannaria*.]
*Gyalideopsis* sp. S39617 — Muscicolous on log in upper beach meadow, 0–2 m. **GUS:** 437, S39167. This species differs from the locally common *G. epicorticis* in the long elongated, not hooked, conidial mass, strongly resembling *G. cyanophila* (Sérusiaux 1998). The material is sparse and not fertile.
*Gypsoplaca* sp. S38752 — Saxicolous on argillite, 830 m. **EX:** 404, S38752. This specimen was included by Garima Singh in an evolutionary study of *Protoparmelia*, but published results of DNA sequences (Singh *et al.* 2015) as well as a subsequent phylogenetic analysis (Shi *et al.* 2018) indicate it is an undescribed member of the genus *Gypsoplaca*.
*Halecania* sp. S38343 — Saxicolous on argillitic rock, 895 m. **EX:** 370, S38343. Similar to *H. athallina* (newly described in this paper) but with apothecia on small discrete areoles.
*Hypogymnia* sp. S36014 — Corticolous and lignicolous on *Tsuga* bark, 48–713 m. **GUS:** 855, 36014; **EX:** ‘Mooselator’ [west slope of Excursion Ridge], S38905. This species has the chemistry of *H. wilfiana* Goward *et al*. (atr, physodic and 2′-*O*-methylphysodic acids) but lacks apinnatic acid. In addition, it differs in its trailing habit, with slightly upturned lobe tips that tend to end in flared ‘thumbs’ (as opposed to the ‘paws’ formed by *H. wilfiana*).
*Hypogymnia* sp. S36481 — Corticolous on *Alnus* bark, 30 m. **GB:** 868, S36481. This species superficially resembles *H. vittata* in possessing trailing, narrow lobes with lobe tips that end in flared hood-soralia. However, it differs in that the lower surface is completely eroded, recalling the East Asian species *H. fragillima*, and in chemistry (atr, physodic and 2′-*O*-methylphysodic acids), lacking vittatolic acid, which is otherwise present in all specimens of *H. vittata* tested in GLBA (*n* = 10).
*Hypogymnia* sp. S38816 — Corticolous on krummholz *Picea*, 922 m. **EX:** 407, S38802, S38816. This is a ‘*Cavernularia*’-type *Hypogymnia* in which the entire upper surface is covered with erumpent isidia; soredia, pycnidia and apothecia are absent. The form was not recognized in the field, where it was mistaken for *Imshaugia aleurites*. This species contains atranorin, physodic acid and unidentified substances. An ITS sequence from S38816 (isolate DNA T1315, Table 1) has base call ambiguities at 14 positions relative to a reference sequence of *Hypogymnia lophyrea* (HQ725073), which could indicate the presence of multiple haploid strains in the PCR template.
*Lecanora* sp. F10122 — Saxicolous on alpine argillite, 922–942 m. **EX:** 375, S38425; 407, F10122, S38827; 408, S38863. TLC: atr. This is a member of the *L. formosa* group, probably related to our new species *L. viridipruinosa* (described above). It differs from the latter in possessing a scurfy, rimose thallus and lacking zeorin. The apothecia apparently vary from pruinose (S38425) to somewhat shiny (F10122). DNA sequences (isolate T1181, Fig. 10) support its distinctness.
*Lecanora* sp. F10126 — Saxicolous, 618–922 m. **DUN:** 428, S39021 (aeruginose form; sub *Euopsis granatina*); **EX:** 407, F10126; 408, S38865 (sub *Sagedia simoënsis*). This appears to be a member of the *L. polytropa* group with strongly aeruginose apothecia. TLC: usnic acid, zeorin.
*Lecanora* sp. S36321 — Corticolous on dead *Alnus* twigs below eagle perches, *c.* 0–5 m. **GB:** 864, S36353, S36355 (sub *Physcia adscendens*); Willoughby Island, S36321 (leg. A. Fryday). TLC: usnic and cf. variolaric acids. Ascospores 10 × 5.5 μm; apothecial section with POL+ crystals, KOH−, C−, PD−. We know of no species with this chemistry.
*Lecanora* sp. S38412 — Saxicolous on argillite on alpine ridgetop, 919 m. **EX:** 374, S38412. TLC: atr, roccellic/angardianic acids. This species is morphologically similar to *L. gangaleoides* but lacks the characteristic substance gangaleoidin. We have sequenced the ITS and mitochondrial SSU rDNA of this specimen (isolate T1333, Fig. 10, Table 1) but cannot place it with certainty without a more comprehensive molecular taxon sampling of *Lecanora* s. lat.
*Lecanora* sp. S38599 — Corticolous on *Salix*, 2 m. **WA:** 391, S38599, S38605. TLC: atr, zeorin. Known from two collections, this is a member of the *L. subfusca* group, similar to *L. glabrata*, with atranorin and a faint trace of zeorin and no detectable fatty acids. The asci are strikingly thick-walled but no ascospores could be found in our material. The epihymenium lacks a continuous crystal layer; instead it is inspersed with scattered, fine crystals.
*Lecanora* sp. T41777 — Lignicolous on driftwood, 2 m. **DUN:** 571, T41777. Similar to *L. symmicta* but with strongly clustered apothecia and possessing only an unknown pigment in TLC.
*Lempholemma* sp. S39326 — Terricolous on silt over bedrock in creek, 155 m. **EX:** 444, S39326. Similar to *L. radiatum* but with wider, canaliculate lobes.
*Lepraria* sp. S39564 — Terricolous on roots under tip-up, 9 m. **DUN:** 463, S39564. TLC: atr, angardianic/roccellic acid, stictic acid + satellite, two pigments. This does not fit any species known to us, but the material is insufficient to describe it here as new.
*Megalaria* sp. F10005 — Saxicolous on an upper beach pebble, 0–5 m. **WA:** 105, F10005. This species differs from known species of *Megalaria* in being saxicolous, as well as in having smaller ascospores (15‒17 × 5‒6 μm) and a hyaline hypothecium. The placement in *Megalaria* is provisional, and is based on the 1-septate ascospores and *Lecidella*-type ascus structure. The type specimen of *Megalaria*, *M. grossa*, possesses a *Lecidella*-type ascus and not *Lecanora-*type as usually reported (e.g. Hafellner 1984).
*Micarea* sp. F10313 — Corticolous, 687 m. **EX:** 448, F10313. Unpigmented apothecia; ascospores 3-septate, 25–28 × 4–5 μm; internally unpigmented, C−, KOH−. This species may be a gyrophoric acid-deficient form of *M. peliocarpa*; the ascospore size is comparable to *M. alabastrites*, but the epihymenium in that species is C+ red.
*Micarea* sp. F10320 — Saxicolous on rocks near ground level in snowbed (upper bay) or subalpine ridges, 43–700 m. **EX:** 449, F10320 (sub *Rhizocarpon anaperum*); **WA:** 319, S37940. This species has a pale grey, almost white thallus, a red-brown, KOH− hypothecium and an unidentified lichen substance detected by TLC. It is similar to *Micarea subconfusa* (which has a smaller and lead grey thallus) and *M. assimilata* (which is muscicolous and has a KOH+ red hypothecium). No other species with secondary chemistry is known in the *M. assimilata* group.
*Micarea* sp. S38509 — Corticolous on *Shepherdia canadensis*, 10 m. **WA:** 384, S38508 (sub ‘Unknown genus S38989’), S38509 (sub *Biatora meiocarpa*). TLC: methoxymicareic acid (G. Thor, 7/2019). Similar to *Micarea nowakii* Czarnota & Coppins in the size of the apothecia and ascospores and the presence of KOH+ mauve pigments, but differing in thallus chemistry (methoxymicareic instead of micareic acid).
*Mycocalicium* sp. S39331 — On *Tsuga* resin, 155 m. **EX:** 444, S39331. Stalk KOH+ bleeding reddish brown; ascospores 8–9 × 4.5–5 μm, with distinctly blunt ends.
*Myochroidea* sp. S39317 — Corticolous on *Salix*, 2 m. **EA:** near 438, S39317. Similar in morphology to *Myochroidea minutula*, with ascospores 10 × 5 μm, but disc distinctly concave.
*Ochrolechia* sp. S38011 — Corticolous on *Alnus*, *Cupressus nootkatensis*, *Picea sitchensis*, *Populus balsamifera* and *Salix,* on *Tsuga* twigs and also lignicolous on beach logs, 0–58 m. **DUN:** 462, S39728; 463, S39528; 467, S39671; **EA:** 122, F10228; near 438, S39303, S39304; **EX:** 858, S36180; **GB:** 864, S36340, S36368; 868, S36475, S36534; 327, S38011; S Sandy Cove, S40751; **GUS:** 857, S36123; 876, S36744; 878, S36783; 879, S36802; 434, S39103; 230, M2753; 556, T41657; 576, T41835; **WA:** 391, S38593, S38603; 549, T41640. This species is discussed at length in the ‘Other Species Treated in Detail’ section and appears to be related to *Ochrolechia xanthostoma*, a species reported from Norway, Scotland and New Zealand. It is not possible to determine whether the GLBA material is a new species, however, until the status of *O. xanthostoma* s. str. is resolved and more is known about the range of variability of the sorediate taxon *O. minuta*, which was described from Kodiak Island, Alaska.
*Ochrolechia* sp. S38864 — Saxicolous on the highest ridgetop rocks, possibly snow-free most of winter, 942 m. **EX:** 408, S38864. TLC: gyrophoric acid. A species with a thick thallus formed of peculiar bullate areoles that, over time, turn tan in herbarium specimens. The cortex is C+ red, the medulla C−. The cortex is also heavily inspersed with small crystals that dissolve in KOH to give a yellow solution. DNA was obtained (isolate T1341, Fig. 6) and suggests that this species is distinct from *O. tartarea* (L.) A. Massal., a species that can share a similar thallus morphology.
*Ochrolechia* sp. S38970 — Muscicolous on exposed rock, 406 m. **DUN:** 423, S38968, S38970. TLC: gyrophoric and lecanoric acids, 1–2 fatty acids. A sterile species characterized by areoles which break open and reveal soredia within, in some cases forming small irregular ‘hoods’. A collection from Excursion Ridge (**EX:** 455, 39421) is not an exact morphological match but may also belong here, and the chemistry is similar (gyrophoric and lecanoric acids, one fatty acid).
*Palicella* sp. T41595 — Corticolous on *Salix*, 2 m. **WA:** 391, S38598; 542, T41595, T41597. TLC: usnic acid, zeorin, 1–3 unidentified pigments. The GLBA material resembles *Palicella filamentosa* in habit but that species contains atranorin, usnic acid and paraensic acid D (Palice *et al.* 2011). The material differs from *L. symmicta* in its small, highly convex, brown apothecia.
*Parmeliella* sp. S24412 — Terricolous on fine organic accumulations, 903–918 m. **EX:** 454, S39414; 455, S39453. This matches *Parmeliella* sp. S24412 from KLGO (Spribille *et al*. 2010), DNA sequences of which were already published by Muggia *et al.* (2011; 28S and mitochondrial SSU from two specimens). It is ecologically and morphologically distinct from *Parmeliella triptophylla*, but unfortunately our material is not sufficient to describe it as a new species. This would also require the review of numerous types, an effort that is beyond the scope of this study.
*Pertusaria* sp. S26420 — Saxicolous on underhang, 10 m. **WA:** 384, S38523. This appears to be an exact match to *Pertusaria* sp. S26420 from KLGO (Spribille *et al.* 2010). The thallus reacts KOH+ red needles in section (norstictic acid) and contains pinkish pigments in the soralia. The genus is speculative.
*Pertusaria* sp. S38786 — Saxicolous (closely adhering to rock) on argillite in the alpine zone, 903–922 m. **EX:** 406, S38786; 407, S38835. TLC: norstictic acid. This species is similar to *P. alaskensis*, described from Unalaska (Erichsen 1938), but differs in having ascospores 6 per ascus (observed on intact asci), 80–98 × 26–28 μm, and an epithecium KOH+ magenta.
*Pertusaria* sp. S39274 — Corticolous on *Alnus*, 2 m. **EA:** 441, S39274. Similar to *P. sommerfeltii* but possesses warts with a highly constricted base and a whitish rim around the ostiole; the hymenium is creamish, KOH+ intensifying, and the ostiole is brown and KOH−.
*Pertusaria* sp. T41520 — Corticolous on *Tsuga* trunks, 40 m. **GUS:** 341, S38257; 531, T41520. TLC: fpc. This species is unusual in being dominated by large, pink pycnidia (conidia rod shaped, *c.* 5 × 1 μm), giving it the habit of an *Ochrolechia*, but containing fumarprotocetraric acid.
*Placopsis* sp. F9765 — Saxicolous on rocks in recently deglaciated forefields, 50 m. **EA:** 869, F9765 (sub *P. fusciduloides*). This is an undescribed member of the *P. lambii* group (dubbed *Placopsis* ‘*scripta*’ by Schneider *et al*. (2016): Fig. 2, a *nomen nudum*) characterized by darkly pigmented soredia in soralia arranged perpendicular to the lobe axis. It will be described in detail elsewhere.
*Placopsis* sp. S39599 — Saxicolous on rocky headlands and boulders, 0–11 m. **DUN:** 463, S39599; **EA:** 872, S36598; **EX:** 217, M2609; 218, M2663; **GUS:** 876, S36752. This species has often been referred to *P. lambii* in the western North American literature but differs from that species in, amongst other things, possessing cephalodia. It also differs from *P. fusciduloides* in its mostly epruinose lobe tips. It will be described elsewhere.
*Placynthium* sp. S38458 — Saxicolous on limestone, 15–22 m. **GB:** 864, F9720, F9725, F9730; **WA:** 318, S37914; 381, S38458. Specimen S38458 was sequenced and clusters with a specimen from Coronation Island on the Tongass NF (isolates T1310 and T1304, respectively; Fig. 9), and broadly with other specimens identified in the past as *P. nigrum* from western North America. However, none of the material so far sequenced from western North America, except one specimen from Montana, genetically matches European *P. nigrum*. Specimens have 3-septate ascospores. It is possible that material with 1-septate and 3-septate ascospores is genetically distinct and the assumption that they are not by Henssen (1963) has led to a species circumscription that is now heterogeneous. We cannot rule out at this point that this species occurs in Europe and already has a name.
*Placynthium* sp. S38419 — Saxicolous in the alpine zone, 936 m. **EX:** 375, S38419 (sub *Lecanora viridipruinosa*). This *Placynthium* species differs from all others collected in its combination of 1-septate ascospores and thick paraphyses. A DNA sequence (isolate T1305, Fig. 9) was recovered in the *P. asperellum* group.
*Platismatia* sp. S38191 — Corticolous on *Picea sitchensis* and *Tsuga heterophylla* branches, 0–27 m. **DUN:** 339, S38191; **GB:** 868, S36519. TLC: atr, caperatic acid. This morph, collected at Fern Harbor and Seebree Island, has much smaller pycnidia than *P. glauca* and does not develop a dark lower surface; it could easily be confused with a pale *Tuckermannopsis chlorophylla*. Extractions have yielded DNA but no PCR products have been obtained.
*Polycauliona* sp. S39572 — Saxicolous on rocky headlands, 10 m. **DUN:** 463, S39572, S39573. Analysis of ITS rDNA sequences (including isolate T1301 (Table 1) and a sequence from a specimen from Mitkof Island, below) based on the taxon sample of Arup *et al.* (2013) suggest that this species is sister to *Polycauliona candelaria*. In GLBA and elsewhere in SE Alaska, this species appears to be restricted to the upper supralittoral zone, which combined with its thick, strap-shaped lobes distinguish it from the other frequent species of *Polycauliona* in the area, *P. pollinarioides* (see ‘Catalogue of All Named Taxa Found’). The latter species can occur on both coastal rocks and trees. We hesitate to proceed with describing a new species after sequencing a specimen from Vancouver Island, British Columbia (data not shown) that morphologically fits the GLBA material but yielded an ITS rDNA sequence corresponding to *P. pollinarioides*. Other specimen examined: Mitkof Island, *Fryday* 10661 (MSC; DNA voucher KS140, Table 1).
#*Polycoccum* sp. P2287 — Lichenicolous on *Acarospora* sp. near glacier terminus, 15 m. **WA:** A571, P2287. Two other *Polycoccum* species are known growing on *Acarospora* species, namely *P. microsticticum* and *P. acarosporicola* (Atienza *et al.* 2003; Halıcı *et al.* 2013). Both species also grow on members of the subgenus *Phaeothallia* but they have larger ascospores than the specimen studied from Glacier Bay. *Polycoccum microstictum* ascospores are 14–18 × 7–8 μm and those of *P. acarosporicola* are even larger (28.5–31.5 × 8–9.5 μm). Ascospores observed in specimen P2287 are 13–14 × 5–6 μm. This size is similar to ascospores found in *P. rubellianae* (11–14 × 6–7 μm), a species known only from *Caloplaca rubelliana*. Material found so far is too scanty for a formal description.
*Porpidia* sp. F10440 — Saxicolous on side of large glacial erratic in muskeg, 22 m. **DUN:** 468, F10440. This collection was initially identified as *P. carlottiana* because of the pruinose apothecia and exciple with a dark cortex and ± hyaline medulla. However, the apothecia are sessile with a well-developed proper margin, which is anomalous for that species, and furthermore an ITS sequence (U. Ruprecht, unpublished data) placed it in the *P. cinereoatra* group. The exciple pigmentation separates it from all known species of this group, in which the exciple is ± uniformly dark.
+*Ptychographa* sp. T41644 — Lignicolous on loose piece of deciduous shrub near the ground, 5–10 m. **WA:** 552, T41644. Distinguished from the only described species in the genus, *P. xylographoides*, by the lack of a lichenized thallus and the tendency for wood to be eroded everywhere except under the ascomata.
*Puttea* sp. S38314 — Lignicolous in deep recess of living, large *Tsuga* tree, 569 m. **EX:** 358, S38314. Ascospores short-fusiform, asymmetrical, 6–10 × 1.8–2 μm; epihymenium POL+, with blue-green, KOH+ green pigments; exciple robust; pycnidia abundant. This does not match any species known to us and its placement in *Puttea* is tentative.
*Pycnora* sp. S40795 — Corticolous on *Salix* branch, 2 m. **EA:** terminus of Riggs Glacier, S40795 (sub *Lecanora symmicta*). Similar in habit to *Lecidea roseotincta* but with a distinctly brown thallus. The ascus in this specimen is similar to that seen in *Pycnora* s. str. (‘tholus with lateral amyloid zone’ (Bendiksby & Timdal 2013); similar to that in *Candelaria*, illustrated by Hafellner (1984), not surprising given that *Pycnora* is now placed in *Candelariales*).
*Ramboldia* sp. S38597 — In apothecia of *Lecanora symmicta*, corticolous on *Salix*, 2 m. **WA:** 391, S38597. Ascospores are 15–16 × 5 μm, often with a slight curve, and the hymenial pigment is green (KOH+ green intensifying). *Ramboldia insidiosa* (Fr.) Hafellner was described from the Alps as a hymenial parasite of *Lecanora varia* that eventually becomes autonomous (Poelt 1974). The present collection appears to have taken over a thallus of *Lecanora symmicta* and differs from *R. insidiosa* in its different host, its relatively convex apothecia and longer ascospores ((8–)6–12 μm in *R. insidiosa*).
*Rhizocarpon* sp. S39392 — Saxicolous on alpine ridge, 918 m. **EX:** 454, S39392. This collection is close to *Rhizocarpon badioatrum* but differs in the thallus containing diffractaic acid (no substances in *R. badioatrum*) and having usually paler brown, thinner, and more convex areolae with a less angular margin. See *Rhizocarpon badioatrum* in the ‘Catalogue of All Named Taxa Found’ for further details.
*Rhizocarpon* sp. F10115 — Saxicolous on alkaline argillite rock outcrop in alpine heath, 922 m. **EX:** 407, F10115. A member of the *R. geographicum* group, this species is unique within the group in having a thallus containing rhizocarpic and norstictic acids and pigmented, muriform ascospores. However, what is truly remarkable about this single collection is the presence of two different types of ascospores, either subglobose (*c.* 15 μm diam.) or broadly ellipsoid (*c.* 45 × 15 μm), in different apothecia and, in one case, the same apothecium, where they occur in discrete hymenia separated by an excipulum.
*Rinodina* sp. S38838 — Saxicolous on soft argillite in the alpine zone, 922 m. **EX:** 407, S38838. Similar to *Rinodina badiexcipula* Sheard but the ascospores are more than 25 μm long, the thalline rim is greyish (not reddish brown) and the thallus is largely lacking or immersed (well developed in *R. badiexcipula*).
*Stereocaulon* sp. S24431 — Saxicolous on rocky shores, 0–10 m. **EA:** just S of terminus of Riggs Glacier, S40806; **GB:** 868, F9757. TLC: atr, lobaric acid. These specimens should be compared to *Stereocaulon* sp. S24431 from KLGO which Spribille *et al.* (2010) discuss in relation to described *Stereocaulon* species. The thalli are characterized by slender pseudopodetia with a scabrose stereome surface and phyllocladia almost completely converted into sorediate masses. In the GLBA specimens two types of cephalodia are represented: pale brown with a reddish brown cyanobacterium (S40806) and grey-brown and cerebriform, with a blue-green cyanobacterium (F9757).
*Stereocaulon* sp. S39567 — Saxicolous on rocky headland, 5–8 m above the high tide line. **DUN:** 463, 39567. TLC: atr, lobaric acid. This species comes closest to *Stereocaulon depreaultii* in the key of Lamb (1978) but differs from all species described by Lamb (1977) in having a completely corticate stereome and bud-like phyllocladia constricted at the bases. Only limited material from a single locality is available. More surveys are needed in Cross Sound and nearby areas to try to locate more populations of this enigmatic species. A DNA sequence is published here (Table 1) for use in future studies.
*Tingiopsidium* sp. F9804 — Saxicolous on sedimentary rock just back from shore, 2 m. **EA:** 872, F9804, F9805; **WA:** 101, F9917. An ITS rDNA sequence from F9805 (isolate T1189, Table 1) suggests it is close to the more southern species *T. sonomense* (unpublished data), but it differs from that species in its peg-like as opposed to strap-shaped isidia and much narrower lobes.
*Trapeliopsis* sp. S40723 — Lignicolous and muscicolous on rotting log suspended in old-growth *Tsuga heterophylla* rainforest, 20 m. **GUS:** Bartlett Lake trail, S40723. This peculiar species is esorediate and fertile, and is characterized by the development of abundant proliferating ascomatal buds: the apothecia are essentially tuberculate in one plane. DNA was obtained (isolate KS87) and places the species on an isolated branch in *Trapeliopsis* relative to well-known species (Fig. 8).
Unknown genus F10343 — Saxicolous in alpine heath, on alkaline argillite, 918 m. **EX:** 454, F10343 (sub *Rhizocarpon chioneum*). The apothecia resemble those of the genus *Catillaria* microscopically (lax paraphyses with a brown, sharply delimited cap) but have simple ascospores. The material is sparse. An mtSSU sequence (T1192, Table 1) was not sufficiently informative to place the species.
Unknown genus S37916 — Terricolous in freshly deglaciated soil, 22 m. **WA:** 318, S37916. This peritheciate species is characterized by large, dark brown, muriform ascospores, to 140 × 45 μm, which can even be seen under a dissecting microscope. Paraphyses remain intact and are anastomosing.
#Unknown genus S38748 — Lichenicolous on *Xylographa* on snag, 717 m. **EX:** 403, S38748. Does not correspond to any known species or genus (M. Zhurbenko, personal communication 2018) but the ascus and ascomatal characters suggest a relationship to *Arthoniaceae*; it differs from most *Arthonia* species in its simple ascospores. Also known from Scotland on *X. trunciseda* (B. Coppins, personal communication 2020).
Unknown genus S38989 — Corticolous on *Shepherdia canadensis* and *Tsuga mertensiana*, 10–465 m. **DUN:** 426, S38989; **WA:** 319, S37921; 384, S38508, S38509 (sub *Biatora meiocarpa*). TLC: unknown phenolic substance. A crust perhaps part of an expanded *Biatora sensu* Kistenich *et al.* (2018) with small apothecia with a thin exciple, and ascospores 11–13 × 5–7 μm; ascus ± Bacidia-type.
Unknown genus S39453 — **EX:** 455, S39453 (sub *Parmeliella* sp. S24412). A minute species with a well-developed thallus and black perithecia. The ascospores are 1 per ascus and 79–82 × 25–34 μm; paraphyses disintegrate but periphyses persist. Asci I+ blue.
Unknown genus T41551 — Corticolous on *Sambucus* bark, 2 m. **GUS:** 532, T41551a (in part), T41551b. An inconspicuous but highly distinctive species that forms a continuous crust of cyanobacterium-filled goniocysts when wet, with apothecia embedded between the goniocysts. Ascospores long-fusiform, non-septate. **GUS:** 857, S36109 may also belong here.
*Varicellaria* sp. S38337 — Loosely saxicolous over ridgetop rocks, 895 m. **EX:** 370, S38337. TLC: gyrophoric acid, unidentified pigment. This sterile species resembles *Ochrolechia* sp. S38864 but its thallus areoles do not become tan in the herbarium packet over time and it is more loosely attached to the rock substratum. An ITS rDNA sequence (isolate T1342, Fig. 6) suggests an affinity to *Varicellaria*, but further research is required.
*Varicellaria* sp. S39454 — Terricolous/muscicolous on organic accumulations, 880 m. **EX:** 455, S39454; 459, F10357. TLC: lecanoric acid. A distinctive sterile species characterized by a chunky coralloid thallus with lobe tips ending in what appear to be incipient but abortive apothecia that are strongly KOH+ yellow in the medulla. The genus placement is speculative in the absence of ascomata or molecular data.

## Catalogue of All Named Taxa Found

#*Abrothallus parmeliarum* (Sommerf.) Arnold — Lichenicolous on corticolous and saxicolous *Parmelia* and *Platismatia* spp. and once on *Parmeliopsis hyperopta*, 0–922 m. **DUN:** 339, S38179 (sub *Lichenopuccinia poeltii*), S38197 (sub *L. poeltii*); 463, S39555, S39590; S583, P2256; S586, P2093; **EX:** 407, S38823 (sub *Stigmidium conspurcans*); S612, P2227; **GB:** 868, S36543 (anamorph), S36538; **GUS:** A569, P2222, P2369, P2387.
**#***Abrothallus prodiens* (Harm.) Diederich & Hafellner — Lichenicolous on thalli of *Hypogymnia apinnata* and *H. enteromorpha* growing on *Picea sitchensis*, 20 m. **GUS:** 881, S36837; 397, S38711; A569, P2255.
#*Abrothallus welwitschii* Tul. — Lichenicolous on *Sticta limbata*, 0–10 m. **GB:** 868, S36512, S36482. This species has been found previously in south-east Alaska from Prince of Wales Island (Diederich 2003), Mitkof Island (unpublished record) and the Chilkoot Trail (Spribille *et al.* 2010).
**Absconditella rosea Kalb & Aptroot — See ‘Other Species Treated in Detail’.
*Absconditella sphagnorum Vězda & Poelt — Muscicolous on *Sphagnum* on hummock in cold muskeg. **DUN:** 415, S38937. First published record for western North America.
*Acarospora badiofusca* (Nyl.) Th. Fr. — Saxicolous; alpine heath with rock outcrops; on alkaline argillite, 830 m. **EX:** 407, F10131 (sub *Protoparmeliopsis muralis*); 409, S38882 (det. M. Westberg, as ‘cf.’). Previously known in Alaska from the Pitmegea River (Thomson 1979).
*Acarospora cervina* A. Massal. — Saxicolous in alpine heath, 830 m. **EX:** 404, F10073 (det. K. Knudsen).
*Acarospora fuscata* (Schrad.) Th. Fr. — Saxicolous on alpine ridge, 919 m. **EX:** 374, S38409 (group; det. M. Westberg). A widespread group containing taxonomically difficult cryptic species.
*Acarospora glaucocarpa* (Ach.) Körb. var. *glaucocarpa* — Saxicolous on pebbles in limestone-influenced areas, rubbly slopes and high beaches, 0–14 m. **WA:** 390, S38581; north beach on isthmus separating north end of Scidmore Bay from main West Arm, S37998.
*Acarospora glaucocarpa* var. *sarcogynoides* H. Magn. — Saxicolous on soft argillite, alpine slope, 922 m. **EX:** 407, S38836 (det. M. Westberg).
*Acarospora sinopica* (Wahlenb.) Körb. — Saxicolous, mainly on metal-rich (highly oxidized) rocks, found in crags and on small boulders behind beaches, 10–15 m. **EA:** 872, S36619 (det. M. Westberg, as ‘cf.’); **GUS:** 435, S39136 (sub *Tremolecia atrata*); **WA:** 105, F10007.
*Adelolecia kolaënsis (Nyl.) Hertel & Rambold — Saxicolous on metamorphic (hornblende augen gneiss) to sedimentary rocks from the supralittoral zone to the alpine zone, 0–918 m. **DUN:** 463, F10390, F10391, F10409, F10410; **EX:** 454, F10334.
*Adelolecia pilati* (Hepp) Hertel & Hafellner — Saxicolous in alpine talus, 907 m. **EX:** 373, S38395.
*Agonimia gelatinosa* (Ach.) Brand & Diederich — Growing over bryophytes on limestone and granite outcrops, 10–100 m. **WA:** 101, F9903 (sub *Leptogium imbricatum*).
*Agonimia tristicula* (Nyl.) Zahlbr. — Muscicolous over rock, 0–10 m. **GB:** 868, F9755, S36541; N Sandy Cove, F9814 (sub *Verrucaria* sp.).
[ **+** *Agyrium rufum* (Pers.) Fr. — Lignicolous on stump in mixed conifer forest, 40 m. **EX:** 125, F10271.]
*Alectoria ochroleuca* (Hoffm.) A. Massal. — Terricolous in alpine heath with rock outcrops, 883–922 m. **EX:** 405, F10079; 407, S38850.
*Alectoria sarmentosa* (Ach.) Ach. — Corticolous on conifer branches and tree trunks, on *Picea*, *Pinus* and *Tsuga*, mostly in montane habitats away from marine influence, and into the krummholz zone, 12–922 m. **DUN:** 337, S38120; 338, S38131; 339, S38185; 463, S39523; **EX:** [858, S36181, S36186]; 353, S38289; 407, S38810; ‘Mooselator’, S38906; 448, S39355; **GB:** 868, S36493; **GUS:** 316, S37878; 397, S38690; 862, S36298. TLC (S36493): usnic, alectoronic, barbatic acids.
*Allocalicium adaequatum* (Nyl.) M. Prieto & Wedin — Corticolous on *Alnus* in beach fringe, 2 m. **GUS:** 341, S38224.
*Alyxoria culmigena* (Libert) Ertz (syn. *Opegrapha herbarum* Mont.) — Lignicolous on hard driftwood just above high tide line, 2 m. **DUN:** 462, F10380, S39500, S39506; 572, T41789, T41790, T41792.
*Amygdalaria consentiens* (Nyl.) Hertel *et al*. — a) acid-deficient chemotype: saxicolous, found twice, on pebble on elevated beach and on top of boulder in muskeg, 0–68 m. **EX:** 413, S38929; **WA:** 384, S38525; 391, S38632. TLC: nil; b) stictic chemotype: saxicolous on rocks in the alpine zone, 903–936 m. **EX:** 375, S38423, S38420; 455, S39424; TLC: stictic, constictic acids; c) chemotype not identified: saxicolous on granitic rocks on beach ridges, 0–30 m. **EA:** 869, F9761; **WA:** 103, F9949; 105, F9997; 545, T41620.
*Amygdalaria continua* Brodo & Hertel — Saxicolous on granitic rock outcrops in forest and on alpine ridges, including in areas of water seepage, 80–435 m. **DUN:** 120, F10172; 414, F10141.
*Amygdalaria pelobotryon* (Wahlenb.) Norman — Saxicolous on pebbles and small rocks from uplifted beach ridges (*c.* 20 yr old) to alpine tundra, 43–937 m. **EA:** 869, S36557 (as ‘aff.’); **EX:** 405, F10102; 409, S38899 (as ‘aff.’); 373, S38384; 454, S39407, F10331. TLC: gyrophoric acid.
*Amygdalaria subdissentiens* (Nyl.) M. Inoue & Brodo — Saxicolous on vertical rock face, 406 m. **DUN:** 423, S38977 (as ‘aff.’); **EX:** 373, S38392. TLC: stictic, gyrophoric acids. The specimen from Dundas (S38977) has a more dispersed thallus than the typical Excursion Ridge specimen, with an indeterminate margin and much less frequent apothecia and cephalodia. Furthermore, the cephalodia appear to contain the cyanobacterium *Gloeocapsa* (normally *Stigonema* in this lichen).
*Anisomeridium polypori (Ellis & Everh.) M. E. Barr — Corticolous on *Populus balsamifera*, 3 m. **GB:** 874, S36697.
*Arctomia delicatula* Th. Fr. — Unspecific in substratum association: most often corticolous on *Alnus* and *Populus balsamifera*, but also once on *Picea sitchensis* twigs, once lichenicolous over *Physcia*, once muscicolous in glacial outwash plain, and once terricolous in the alpine zone, 0–880 m. Often mixed in amongst other lichens in small quantities and not detected in the field. **EA:** 440, S39192, S39194, S39245, S39236; 233, M2776; **EX:** 459, F10360 (sub *Candelariella* sp.); **GB:** 865, S36401 (sub *Cheiromycina petri*); **GUS:** 857, S36139 (sub *Leptogium saturninum*); **WA:** 105, F10001 (sub *Porpidia* cf. *thomsonii*); 322, S37968; 323, S37969; 384, S38512; 395, S38667 (sub *Caloplaca atrosanguinea*), S38682 (sub *Fuscopannaria convexa*); 542, T41592; 545, T41621b; 549, T41634. We extracted DNA from one sample (isolate P166, from S37968, published by Resl *et al.* (2015)) to explore whether the GLBA species might be divergent from the putatively tundra-restricted arctic-alpine form known in Europe, but ITS rDNA data did not suggest any divergence.
*Arthonia arthonioides* (Ach.) A. L. Sm. — Corticolous on *Cupressus nootkatensis*, *Picea sitchensis* and *Tsuga heterophylla* in coastal *Tsuga-Picea* forest, 0–50 m. **DUN:** 131, F10446; 332, S38065; 334, S38097, S38099; **EX:** 213, M2568; [858, S36189]; 446, S39336; 448, S39347; **GUS:** 100, F9893; 136, F10466; 855, F9606; Bartlett Cove housing complex, F9849, F9861; 876, S36734.
**#***Arthonia biatoricola* Ihlen & Owe-Larss. — Lichenicolous on *Micarea*, overgrowing *Picea sitchensis* twigs, also over *Populus balsamifera*, 0–10 m. **GB:** 868, S36531; **GUS:** 341, S38246.
*Arthonia aff. *didyma* Körb. — Lignicolous on snag and corticolous on *Picea sitchensis*, 0–33 m. **GUS:** [V329, S38053]; 436, S39156; 204, S38438. Our material is similar to *A. didyma*, differing in subtle traits of size and habit (M. Grube, personal communication). However, in the absence of detailed study it is not clear this warrants recognition as another taxon.
*#*Arthonia digitatae* Hafellner — Lichenicolous on *Cladonia* cf. *umbricola*, 0–60 m. **DUN:** 219, M2674b; **WA:** A578, P2364.
*Arthonia ilicina* Taylor — Corticolous on *Alnus* and *Malus*, 9–86 m. **DUN:** 562, T41746; 333, S38075, S38077, S38086; 462, S39720 (as ‘aff.’); **EX:** [125, F10277]; 433, S39074.
**#*Arthonia lepidophila* (Anzi) Clauzade *et al*. — Lichenicolous on *Cladonia* cf. *squamosa*, 203 m. **EX:** 565, P2338. Characterized by the combination of 2-septate ascospores and the KOH+ olive hymenial reaction.
**Arthonia ligniaria Hellb. — Lignicolous on snag, 51 m. **GUS:** 882, S36839. Ascospores 1-septate, *c*. 18 × 7.5 μm and hymenium I−. *Arthonia ligniaria* was described from Sweden and has been reported from the UK (Coppins 1989*b*) and elsewhere in Europe.
*Arthonia ligniariella* Coppins — Lignicolous in deep recess (hollow) of large, live *Tsuga*, 569 m. **EX:** 358, S38315.
*Arthonia muscigena* auct., s. lat. — Over metamorphic rock in the supralittoral zone, in splash zone; also corticolous on *Populus balsamifera* and on *Tsuga* twig, and foliicolous on *Picea sitchensis*, 0–48 m. **DUN:** 134, F10464 (sub *Hydropunctaria maura*); 573, T41812; **GUS:** 855, S36024; 341, S38241.
*#*Arthonia peltigerea* Th. Fr. — Lichenicolous on *Solorina crocea*, terricolous, 903 m. **EX:** 455, S39440.
*Arthonia phaeobaea* (Norman) Norman — Saxicolous on gneiss in the supralittoral zone, 0–5 m. **DUN:** 462, F10379 (sub *Myriolecis* aff. *contractula*); 220, M2672; **GB:** 865, F9741, F9744; 873, F9821; 874, F9835; 875, F9841, F9842; **GUS:** 340, S38223.
*Arthonia* aff. *radiata* (Pers.) Ach. — Corticolous on *Alnus* bark, 10 m. **DUN:** 463, S39509; 467, S39647.
#*Arthonia stereocaulina* (Ohlert) R. Sant. — Lichenicolous on *Stereocaulon* spp., on sea stacks and in post-glacial *Dryas* mats, 0–115 m. **DUN:** 586, P2161; **EA:** 870, F9781.
**#*Arthonia thelotrematis* Coppins — Lichenicolous on *Thelotrema lepadinum*, corticolous on *Alnus*, 86 m. **DUN:** 333, S38093. This species was described by Coppins (1989*a*) from the UK and the Azores, and subsequently reported from mainland Europe (Etayo & Diederich 1998) and New Zealand.
*Arthonia vinosa Leight. — Corticolous in mixed woodland, 3 m. **GB:** 874, F9829; **GUS:** road to dock, F9852.
#*Arthophacopsis parmeliarum* Hafellner — Lichenicolous on *Parmelia sulcata*, both in rock- and bark-dwelling specimens, 0–10 m. **DUN:** 463, S39556; **WA:** 395, S38676; 542, T41588.
+*Arthopyrenia analepta* (Ach.) A. Massal. — Corticolous on *Alnus* and *Salix*, 0–152 m. **DUN:** 430, S39034; **EA:** 440, S39217; **GB:** 864, S36342, S36375; **GUS:** [V329, S38039 (sub *Arthopyrenia* sp. S38039)]; **WA:** 319, S37919; 327, S38013; 391, S38595.
*+*Arthopyrenia plumbaria* (Stizenb.) R. C. Harris — Corticolous on *Alnus*, 9–27 m. **DUN:** 339, S38147; 462, S39727, S39711.
*Arthothelium macounii (G. Merr.) W. J. Noble — Corticolous on *Cupressus nootkatensis* branch, 68 m. **DUN:** 413, S38928.
*Arthothelium norvegicum Coppins & Tønsberg — Corticolous on *Picea sitchensis* branch, 2 m. **GB:** 868, S36505 (ver. M. Grube). Previously reported from California to British Columbia by Tønsberg & Williams (2006).
*Arthrorhaphis alpina* (Schaer.) R. Sant. — On soil accumulations in crack on side of granitic boulder in muskeg, 90 m. **DUN:** 115, F10144.
**#*Arthrorhaphis muddii* Obermayer — Lichenicolous on *Dibaeis baeomyces*, seepy soil, 597 m. **DUN:** 427, S38997. This species was described from the UK and Austria by Obermayer (1994), who described it in detail, and outside of those countries it has been found only in Norway (Ihlen 1998).
*Aspicilia cf. *fumosa* Owe-Larsson & A. Nordin — Saxicolous, 125 m. **WA:** 388, S38559. Thallus light grey, KOH−; ascospores 20–24 × 13–15 μm; epihymenium olive-brown to olive; hymenium 160–180 μm; excipulum 20–50 μm; paraphyses moniliform; conidia not found. TLC: nil.
*Aspicilia aff. *indissimilis* (H. Magn.) Räsänen — Saxicolous on argillite in the alpine zone, 125–922 m. **EX:** 407, F10117; 458, S39487; **WA:** 388, S38560 (det. T. Wheeler and B. Owe-Larsson). Ascospores (6–)8 per ascus, 19–25 × 10–15 μm (8–10 μm wide in S39487); epihymenium olive to brown; hymenium 100–180 μm; excipulum 30–50 μm, paraphyses (sub-)moniliform; thallus KOH−; conidia 12–16(–18) μm. TLC: nil. The taxonomy of this group requires more research.
*Aspicilia aff. *olivaceobrunnea* Owe-Larss. & A. Nordin — Saxicolous in alpine heath with rock outcrops; on alkaline argillite, 883 m. **EX:** 405, F10095 (sub *Lecidea* aff. *griseomarginata*; det. B. Owe-Larsson).
*Aspicilia subradians* (Nyl.) Hue — Saxicolous on argillite in the alpine zone, 883–919 m. **EX:** 374, S38413 (as ‘aff.’, paraphyses non- to submoniliform, perhaps *A. cinerea* group); 405, F10095 (sub *Lecidea* aff. *griseomarginata*); 407, F10117 (det. B. Owe-Larsson). TLC: norstictic acid.
**Atla recondita Savić & Tibell — Saxicolous on semi-inundated slightly basic rock, with *Staurothele* cf. *verruculosa*, 225 m. **EX:** 128, F10302. The affinity of F10302 to *A. recondita* was confirmed by ITS rDNA (Table 1). This appears to be the first report of the species since it was described from Sweden by Tibell & Tibell (2015).
*Atrophysma cyanomelanos* T. Sprib. — See ‘Descriptions of New Genera and Species’.
#*Bachmanniomyces punctum* (A. Massal.) Diederich & Pino-Bodas (syn. *Phaeopyxis punctum* (A. Massal.) Rambold *et al.*) — Lichenicolous on *Cladonia pyxidata*, 0–3 m. **WA:** A573, P2368.
#*Bachmanniomyces uncialicola* (Zopf) D. Hawksw. — Lichenicolous on *Cladonia amaurocraea*, terricolous, 922 m. **EX:** 407, S38822.
*Bacidia bagliettoana* (A. Massal. & De Not.) Jatta — Muscicolous/terricolous on plant detritus, organic accumulations in rock crevices and on soil in post-glacial *Dryas* mats, 0–115 m. **EA:** 869, S36554; 870, F9774, F9779, F9789; **GB:** 867, S36436; **WA:** 101, F9901 (sub *Thalloidima sedifolium*); 381, S38444; 390, S38587.
*Bacidia rosellizans* S. Ekman — Corticolous on *Alnus*, 46 m. **GUS:** 879, S36808.
**Bacidina brandii (Coppins & van den Boom) M. Hauck & V. Wirth — On organic accumulations on gravelly high beaches and in *Plantago maritima* zone, 0–4 m. **EA:** 438, S39186, F10222; **GB:** 873, S36658. Described from Europe by Coppins & van den Boom (2002) and has since been widely reported there but, apparently, until now, not from elsewhere. Colour photographs have been published by Czarnota (2016).
*Bacidina circumpulla* S. Ekman — See ‘Descriptions of New Genera and Species’.
*Bacidina egenula (Nyl.) Vězda — Saxicolous on gneiss rocks beside creek, 0–5 m. **DUN:** 133, F10457 (sub *Verrucaria* sp.).
**Bacidina saxenii (Erichsen) M. Hauck & V. Wirth — Saxicolous on vertical shale rock face at sea level and side of large roadside boulder, 5–20 m. **DUN:** 462, S39507; **GUS:** 140, F10488. *Bacidina saxenii* has until now been reported only from Europe. Its known distribution and ecology are discussed by Ekman *et al.* (2012), who also provide photographs of the morphology.
**Bacidina sulphurella (Samp.) M. Hauck & V. Wirth — Corticolous on *Ribes lacustre*, 2 m. **GUS:** 341, S38254. Until now reported only from Europe, this species is similar to *B. arnoldiana* in ascomatal characters, but differs in the shape of its conidia (hooked at one end like a walking stick; *B. arnoldiana* has curved but never hooked conidia) and its occurrence as an epiphyte (*B. arnoldiana* is predominantly saxicolous; Coppins & Aptroot 2009).
*Bactrospora cascadensis* Ponzetti & McCune — Corticolous in rain-sheltered underhangs of *Tsuga heterophylla* trunks. In GLBA found only in montane forests, 222–687 m. **EX:** 112, F10064; 313, S37839 (aff.; discussed below); 366, S38335; 448, F10316, S39348; 358, S38308; no waypoint, M2587. S37839 is intermediate between *B. cascadensis* and *B. brodoi*. It resembles *B. brodoi* (and differs from the rest of *B. cascadensis* material cited here) in possessing an entirely endophloedal thallus, with no sign of the typical effuse, scurfy, pinkish thallus of *B. cascadensis*. However, it differs from *B. brodoi* (and agrees with *B. cascadensis*) in possessing Patellarioides-type ascospores (2.2–)2.8(–3.5) μm wide (narrower than in the otherwise similar *B. brodoi*; Ponzetti & McCune 2006), asci (11–)12.7(–15) μm wide, an I+ pale blue hymenium and subhymenium, and a strongly I+ blue excipulum.
*Baeomyces rufus* (Huds.) Rebent. — Saxicolous, muscicolous and terricolous on boulders and soil in shaded to open areas from forest to *Dryas*-covered glacial forelands, 0–115 m. **EA:** 869, S36561 (sub *Epilichen scabrosus*); 870, F9780; **GB:** 865, S36414; 868, F9753; **WA:** 383, S38485. One GLBA specimen has been used as a source of DNA in phylogenetic studies (Resl *et al.* 2015, isolate P82).
*Bellemerea cinereorufescens* (Ach.) Clauzade & Cl. Roux — Saxicolous in alpine heath with rock outcrops; on alkaline argillite, 830 m. **EX:** 404, F10068 (sub *Lecidella carpathica*), F10069.
*Bellemerea subsorediza* (Lynge) R. Sant. — Saxicolous on 20 yr-old gravels and granitic rocks, 0–43 m. **EA:** 869, S36559; **WA:** 383, S38493; 102, F9935; 388, S38561 (sub *Candelariella vitellina*). TLC: norstictic acid.
*Belonia incarnata Th. Fr. & Graewe — Terricolous in alpine sod, 918 m. **EX:** 454, S39411.
*Belonia russula* Körb. ex Nyl. — Saxicolous in alpine heath with rock outcrops; on alkaline argillite, 918 m. **EX:** 454, F10342.
*Biatora aegrefaciens* Printzen — Corticolous on *Picea sitchensis* twigs on exposed coastal headland, 27 m. **DUN:** 339, S38172.
*Biatora alaskana* Printzen & Tønsberg — Corticolous on *Alnus*, *Picea sitchensis* and *Tsuga heterophylla*, 0–213 m. **EX:** 312, S37816; [V431, S39057]; **GUS:** [V329, S38026]; 436, S39137; 876, S36728; Tower Rd, S38270; 210, M2555. We also include a form here we considered describing as a new species because it consistently has single-celled, fusiform ascospores up to 35 × 6 μm. This form was already treated in KLGO (Spribille *et al.* 2010) as *Biatora* sp. 24799. We, however, remain unconvinced and molecular data have not been obtained to test the hypothesis one way or another. Representative specimens are: **DUN:** 463, S39551; **GUS:** 856, F9616; 857, F9635. TLC: nil.
*Biatora albohyalina* (Nyl.) Bagl. & Carestia — Corticolous on *Ribes bracteosum*, 710 m. **EX:** 450, S39363 (det. C. Printzen). Ascospores 8–10.5 μm long, shorter than typical for the species.
*Biatora alnetorum* S. Ekman & Tønsberg — Corticolous on *Alnus*, 8 m. **EA:** 440, S39231. TLC: atr. Sterile, soredia as in Ekman & Tønsberg (2019).
*Biatora aureolepra T. Sprib. & Tønsberg — Corticolous on *Picea sitchensis* branch, 33 m. **GUS:** 204, S38436.
*Biatora beckhausii* (Körb.) Tuck. — Corticolous on *Salix*, 24 m. **WA:** 206, M2505.
*Biatora* aff. *chrysantha* (Zahlbr.) Printzen — Corticolous on *Alnus* and *Cupressus nootkatensis*, 8–42 m. **DUN:** 469, S39696 (det. C. Printzen); **EA:** 440, S39212; **WA:** 549, T41627b. Specimens are sterile and treated as ‘aff. *chrysantha*’ here because it is not possible to distinguish *B. chrysantha* and *B. chrysanthoides* in the sterile state (Printzen & Tønsberg 2003).
*Biatora cuprea* (Sommerf.) Fr. — On moss and detritus over rock, 922–936 m. **EX:** 375, S38418; 407, S38860.
*Biatora efflorescens* (Hedl.) Räsänen — Corticolous on *Alnus*, 9 m. **DUN:** 462, S39718; **EA:** 440, S39219, S39223; **GB:** 868, S36535; 556, T41683. TLC: argopsin, norargopsin (trace).
*Biatora flavopunctata* (Tønsberg) Hinteregger & Printzen — Corticolous on *Shepherdia canadensis* and *Vaccinium*, 10–713 m. **EX:** 376, S38430 (sub *Biatora meiocarpa* var. *tacomensis*); **WA:** 384, S38511; 544, T41603.
*Biatora hypophaea* Printzen & Tønsberg — Corticolous on *Alnus*, *Malus fusca*, *Oplopanax horridus*, *Picea sitchensis*, *Ribes lacustre*, *Salix*, *Sambucus racemosa* and *Viburnum edule*, 0–195 m. An extremely common low elevation crustose epiphyte in GLBA. **DUN:** 333, S38076, S38083; 336, S38115; 462, S39700, S39703 (sub *Arthopyrenia* sp.); **EA:** 123, F10245; 440, S39203 (conf. C. Printzen), S39226, S39237, S39255; 441, F10193; 442, 39291 (sub *Micarea cinerea*); 872, S36610; 234, M2804; no waypoint, M2808, M2816; **EX:** [125, F10255, F10260]; 432, S39067; **GB:** 864, S36339; 865, S36407; **GUS:** 100, F9895; 138, F10468, F10472, F10474; [V329, S38027]; 330, S38063 (sub *Fellhaneropsis vezdae*); 341, S38248 (sub *Biatora sphaeroidiza*), S38251, S38263 (conf. C. Printzen); 857, F9641, S36094, S36135; 857, S36138 (sub *Micarea cinerea*); 879, S36815, S36804; 436, S39162; housing complex, F9865; 224, M2706; 228, M2742; 230, M2764; 232, M2772.
*Biatora kodiakensis* Printzen & Tønsberg — Corticolous on *Alnus*, *Oplopanax horridus* and *Viburnum edule*, 0–10 m. **GB:** 864, S36362, S36343; 866, S36420 (sub *Bacidina* sp.); 556, T41649, T41681, T41682; **GUS:** 341, S38249 (det. C. Printzen), S38260; 531, T41533; 576, T41849; 228, M2743; **WA:** 549, T41627a, T41638, T41639. TLC: gyrophoric acid [no lecanoric].
*Biatora ligni-mollis* T. Sprib. & Printzen — Lignicolous on conifer, 25 m. **GUS:** Tower Road, M2485.
*Biatora marmorea* T. Sprib. — See ‘Descriptions of New Genera and Species’.
*Biatora meiocarpa* (Nyl.) Arnold — Corticolous on *Alnus*, *Oplopanax horridus*, *Salix* and *Shepherdia canadensis*, 8–92 m. **EA:** Muir Inlet, S36595; 440, S39216, S39232 (sub *Lichenochora lepidiotae*); **GUS:** 341, S38263 (sub *B. hypophaea*; det. C. Printzen); 436, S39145; **WA:** 384, S38509; 387, S38537, S38538 (det. C. Printzen).
*Biatora meiocarpa* var. *tacomensis* Printzen & Tønsberg — Corticolous on *Alnus* bark and *Vaccinium* stalks, 0–713 m. **EX:** 376, S38430; **GUS:** 138, F10476. TLC: xanthones.
*Biatora oligocarpa* Printzen & Tønsberg — Corticolous on *Populus balsamifera*, 0–33 m. **GUS:** [V329, S38050]; [557, T41714a]; **WA:** Blue Mouse Cove plot BM2a (det. TS).
*Biatora rufidula* (Graewe) S. Ekman & Printzen — Corticolous on trunk of fallen *Picea sitchensis*, 3 m. **GB:** 874, S36704.
*Biatora sphaeroidiza* (Vain.) Printzen & Holien — Corticolous on *Oplopanax horridus*, *Picea sitchensis*, *Salix*, *Sambucus racemosa* and *Viburnum edule*, 0–27 m. **DUN:** 339, S38153; **GB:** 864, S36374; **GUS:** 341, S38248 (det. C. Printzen, as ‘aff.’), S38249 (det. C. Printzen, sub *B. kodiakensis*), S38251 (sub *Biatora hypophaea*), S38261; 434, S39099 (sub *Biatora subduplex*), S39102 (det. C. Printzen, as ‘aff.’); 436, S39162 (sub *Biatora hypophaea*), S39145 (sub *Biatora meiocarpa*). TLC: a xanthone. In several specimens the apothecia are paler and the ascospores larger than is usually the case in *B. sphaeroidiza* s. str. (C. Printzen, personal communication 2019).
*Biatora subduplex* (Nyl.) Räsänen ex Printzen, s. lat. — Corticolous on *Alnus* and terricolous/muscicolous in alpine tundra, also once saxicolous in the alpine zone, 0–922 m. Material here includes an unusually wide range of ascospore sizes and might constitute more than one species. **DUN:** 336, S38113 (s. lat.); 466, S39638; **EA:** 440, S39252 (det. C. Printzen, as ‘cf.’), S39254; 441, F10202, F10220; **EX:** [858, S36173]; 404, F10070; 407, F10110; 218, M2667; 455, S39449, S39452; **GB:** 865, S36406; 866, S36427 (s. lat.); 868, S36525; 433, S39083; **GUS:** 138, F10469; 855, S36028, S36034; 857, F9638, S36152; 862, S36310; 878, S36762; 879, S36816; 317, S37896; [V329, S38020]; 341, S38248 (sub *Biatora sphaeroidiza*), S38251 (with large ascospores, sub *Biatora hypophaea*); 434, S39099; Tower Road, S37503; **WA:** 206, M2507; 319, S37920, S37922 (s. lat.), S37927 (det. C. Printzen); 320, S37957; 383, S38487; 387, S38538 (sub *Lecidea albohyalina*), S39541 (s. lat.); 542, T41594; 544, T41607 (sub *Lecidella elaeochroma*).
*Biatora toensbergii* Holien & Printzen — Corticolous on *Alnus*, 4–8 m. **DUN:** 462, S39721, S39703 (sub *Arthopyrenia* sp.; det. C. Printzen); **GB:** 556, T41680; **GUS:** 531, T41532. TLC: argopsin.
*Biatora vacciniicola* (Tønsberg) Printzen — Corticolous on *Alnus*, *Salix* and *Vaccinium* stalks, 0–713 m. **DUN:** 429, S39031; **EA:** Muir Inlet S shore, S36594, S36597; 440, S39219; **EX:** 376, S38430 (sub *Biatora meiocarpa* var. *tacomensis*), S38431; 441, F10206; **GB:** 866, S36422; **WA:** 384, S38505; 387, S38539; 544, T41604 (fertile). TLC: gyrophoric acid.
*Biatora vernalis* (L.) Fr. — On organic accumulation over vertical rock face, 2–830 m. **EX:** 404, S38750; 213, M2574; **WA:** 391, S38617 (det. C. Printzen).
#*Biatoropsis usnearum* Räsänen s. lat. — Lichenicolous on *Usnea longissima*, which was corticolous on windblown *Picea*, headland, 9 m. **DUN:** 463, S39557.
*Bilimbia microcarpa* (Th. Fr.) Th. Fr. — On organic accumulations on base of *Pinus contorta* in open flats, 16 m. **GUS:** 317, S37897.
*Blennothallia fecunda* (Degel.) Otálora *et al*. — Saxicolous on granitic rocks on seashore, 0–10 m. **WA:** 102, F9941.
*Brianaria bauschiana (Körb.) S. Ekman & M. Svenss. — Saxicolous on boulders in deep shade of old-growth forest and *Alnus* thickets, 0–58 m. **GB:** 865, S36413 (det. B. Coppins); **GUS:** 139, F10483, F10484, F10487; 856, F9627; 878, S36778 (det. B. Coppins).
*Brianaria sylvicola* (Flot. ex Körb.) S. Ekman & M. Svenss. — Saxicolous on boulder in open woodland, 15 m. **GUS:** housing complex, F9858.
*Brigantiaea fuscolutea* (Dicks.) R. Sant. — Terricolous between and at the edges of boulders in alpine sod and tundra, 830–922 m. **EX:** 374, S38415; 404, S38756; 407, F10112; 456, S39462; 457, S39480; 218, M2618a.
*Brigantiaea praetermissa Hafellner & St. Clair — Corticolous on *Cupressus nootkatensis*, 13 m. **DUN:** 466, S39634 (fertile). TLC: atr, zeorin. A predominantly inland species of British Columbia, south to Montana.
*Bryobilimbia hypnorum* (Lib.) Fryday *et al*. — Terricolous on organic accumulations over bases of trees and over rocks, as well as directly on bryophytes, from beaches to alpine ridges, 0–903 m. **DUN:** 120, F10168 (as ‘cf.’); **EX:** 455, S39439; 218, M2631a, M2635; **GUS:** 317, S37898; **WA:** 103, F9958; 104, F9968; 105, F9995 (sub *Caloplaca sinapisperma*), F9996; 318, S37909; 381, S38469; 205, M2492.
*Bryocaulon divergens* (Ach.) Kärnefelt — Terricolous in alpine heaths and tundra, 894–922 m. **EX:** 372, S38373; 407, S38848; 455, S39450; 406, S38794.
*Bryocaulon pseudosatoanum* (Asahina) Kärnefelt — Lignicolous on *Pinus contorta* in muskeg, 255 m. **EX:** Yellowlegs Savanna, S37852. Medulla C+ red.
**Bryodina rhypariza (Nyl.) Hafellner & Türk — Muscicolous over top of rock, alpine zone, 937 m. **EX:** 409, S38876 (det. W. Obermayer). New to continental North America, previous North American records have been from the Canadian Arctic Archipelago (Fryday 2000; Zhurbenko *et al.* 2010).
*Bryonora curvescens* (Mudd) Poelt — Muscicolous over rock, 903 m. **EX:** 406, S38778.
*Bryoria americana* (Motyka) Holien — Corticolous on *Picea sitchensis*, *Pinus contorta* and *Tsuga heterophylla*, 0–687 m. **EX:** 358, S38312 (det. T. Goward); 448, S39356, S39357; **GB:** 874, S36674, S36675; **GUS:** 107, F10024, F10026; 397, S38700 (det. T. Goward).
*Bryoria bicolor* (Ehrh.) Brodo & D. Hawksw. — Corticolous on *Picea sitchensis* branches, mainly in beach fringe or headland habitats, 10–60 m. **DUN:** 339, S38163; **GB:** 868, S36502; **GUS:** [V329, S38048]; 857, S36106. TLC: pc (3×) or fpc (1×).
*Bryoria carlottae* Brodo & D. Hawksw. — Lignicolous on *Pinus contorta* trees or snags in muskeg, 0–236 m. **DUN:** 338, S38129 (ver. T. Goward), S38135, S38139; 463, S39525 (det. T. Goward, as ‘cf.’); **EX:** 399, S38728; **GUS:** 397, S38699. TLC: pc.
*Bryoria friabilis* Brodo & D. Hawksw. — Corticolous on *Picea sitchensis*; our records are all from beach fringe habitats, 0–60 m. **GB:** 868, S36492; 874, S36676 (ver. T. Goward), S36678; **GUS:** 435, S39119, S39129; 857, S36116. TLC: gyrophoric acid.
[*Bryoria fuscescens* (Gyeln.) Brodo & D. Hawksw. — Corticolous on *Tsuga* snag, 59 m. **EX:** 860, S36247.]
*Bryoria glabra* (Motyka) Brodo & D. Hawksw. — Corticolous on *Picea sitchensis*, 3 m. **GB:** 874, S36673. TLC: pc.
*Bryoria inactiva* Goward *et al*. — Corticolous, probably on *Picea sitchensis* branch, found on branch fallen from canopy, 4 m. **GUS:** between lodge and beach, Park HQ GLBA, S38269 (det. T. Goward).
*Bryoria lanestris* (Ach.) Brodo & D. Hawksw. — Corticolous on *Pinus contorta* and *Tsuga mertensiana* branches in muskeg, 12–333 m. **EX:** 353, S38288; **GUS:** 316, S37873; 397, S38701. TLC: pc (2×) and fpc (1×).
*Bryoria nitidula* (Th. Fr.) Brodo & D. Hawksw. — Terricolous in alpine heath, 872–907 m. **EX:** between 405 and 406, S38773; 373, S38379; 456, S39468. TLC: pc.
*Bryoria pikei* Brodo & D. Hawksw. — Corticolous on *Picea sitchensis* branches; our records are all from beach or headland habitats, 0–12 m. **DUN:** 463, S39527; 467, S39650; **GB:** 874, S36679; **GUS:** 204, S38437; 397, S38696, S38706; 435, S39117; 463, S39531. TLC: alectorialic, barbatolic acids.
*Bryoria pseudofuscescens* (Gyeln.) Brodo & D. Hawksw. — Corticolous on *Picea sitchensis* branches, 3 m. **GB:** 874, S36672, S36677. TLC: norstictic acid.
*Buellia coniops* (Wahlenb.) Th. Fr. — Saxicolous, mainly on gneiss boulders and closely associated with seashores, 0–5 m. **DUN:** 463, F10379 (sub *Myriolecis* aff. *contractula*), F10403; S39580; **GB:** 863, F9704; 865, F9737, F9739, F9743; 873, F9824, S36655, S36656; **GUS:** 875, F9843, F9844, F9845; **WA:** 322, S37963; 549, T41629b.
*Buellia erubescens* Arnold — Corticolous on *Alnus* in beach fringe, sea level. **GB:** 864, S36326.
*Buellia griseovirens* (Turner & Borrer ex Sm.) Almb. — Corticolous on *Alnus*, *Picea sitchensis* and *Shepherdia canadensis*, also lignicolous on beach logs, 0–46 m. **DUN:** 113, F10133 (sub *Xylographa hians*); **GUS:** 876, S36741; 316, S37884; 434, S39107; 879, S36805; 531, T41537; 576, T41848, T41850. TLC: atr, norstictic acid.
*Buellia cf. *sequax* (Nyl.) Zahlbr. — Saxicolous on alpine ridgetop, 919 m. **EX:** 374, S38412 (sub *Lecanora* sp.). A southern species, identification tentative.
*Buellia triseptata A. Nordin — Corticolous on fine twigs of *Picea sitchensis* and *Tsuga heterophylla*, 0–27 m. **DUN:** 339, S38184; 463, S39541; **GUS:** 435, S39123.
*Calicium abietinum* Pers. — Lignicolous on conifer in muskeg, 250 m. **EX:** 109, F10053; **GUS:** 435, S39131.
*Calicium glaucellum* Ach. — Lignicolous on snags and exposed dead wood of otherwise still living *Pinus contorta* trees, 12–217 m. **EX:** 313, S37844; 398, S38726; 227, M2737; **GUS:** 397, S38707, S38713, S38714.
*Calicium lenticulare* Ach. — Lignicolous on *Tsuga* trunks and on snags, 124–717 m. **DUN:** nr 334, S38103; 563, T41759; **EX:** 403, S38744; [V431, S39060].
*Calicium parvum* Tibell — Corticolous on *Tsuga* bark, 687 m. **EX:** 448, S39359. First reported for Alaska from Lake Clark by McCune *et al.* (2018).
*Calicium viride* Pers. — Corticolous on *Picea sitchensis* and *Tsuga* underhangs, 12–124 m. **DUN:** 334, S38102; **EX:** 313, S37841; 433, S39094; 448, S39359; **GUS:** 397, S38715.
*Caloplaca allochroa* Y. Joshi *et al*. (syn. *Gyalolechia allochroa* (Y. Joshi *et al.*) Søchting *et al*.) — Saxicolous on vertical sheltered rock with overhanging vegetation, 809 m. **EX:** 452, S39368. A DNA sequence from this specimen was published by Vondrák *et al.* (2016*b*, as *Gyalolechia allochroa*) as new to Alaska and North America.
*Caloplaca ammiospila* (Wahlenb.) H. Olivier **(**syn. *Blastenia ammiospila* (Wahlenb.) Arup *et al.*) — Terricolous or muscicolous from raised beaches to the alpine zone, 0–922 m. **EX:** 407, F10105; **WA:** 391, S38640.
*Caloplaca atrosanguinea* (G. Merr.) I. M. Lamb — Corticolous on *Alnus*, *Populus balsamifera* and *Salix*, 0–215 m. **EA:** 127, F10280; 440, S39195; 441, F10192; 872, S36604; E Muir Pt, M2807, M2820, M2812; **GB:** 864, S36337; 862, S36313; 556, T41675; **GUS:** 862, F9675; [574, T41816]; 576, T41845; 210, M2552; **WA:** 391, S38590; 395, S38667, S38677 (sub *Scoliciosporum chlorococcum*); 542, T41587.
*Caloplaca borealis* (Vain.) Poelt — Corticolous on *Populus balsamifera* and *Salix*, beach fringe habitats, 0–10 m. **GB:** 864, S36334; **WA:** 391, S38608, S38609; 395, S38672.
*Caloplaca caesiorufella* (Nyl.) Zahlbr. — Corticolous on *Salix* in beach fringe, 0–10 m. **DUN:** 463, S39519; **EA:** 122, F10229; 123, F10244; nr 438, S39314, S39315; **GB:** 864, S36360; **GUS:** 316, S37864; 435, S39130. Hansen *et al.* (1987) questioned the distinctiveness of *C. caesiorufella*, and subsequent North American checklists have treated it as a synonym of *C. phaeocarpella*, but we prefer to recognize it until its status can be systematically resolved. An ITS sequence is published here (Table 1).
*Caloplaca exsecuta* (Nyl.) Dalla Torre & Sarnth. — Saxicolous in snowbed habitats, on erratic in muskeg, and on soft argillite in alpine tundra, 22–922 m. **DUN:** 468, S39677; **EX:** 407, F10116 (det. Vondrák); 453, F10325, F10326 (sub *Lecidea lapicida*), 458, S39489; 460, F10361 (det. Vondrák; sub *Rhizocarpon polycarpum*); 218, M2661. A sequence from S24441, from KLGO, has been deposited in GenBank, under Accession MG954227 (Vondrák *et al.* 2019*a*).
*Caloplaca flavocitrina* (Nyl.) H. Olivier (syn. *Flavoplaca flavocitrina* (Nyl.) Arup *et al.*) — Saxicolous, just above sea level. **DUN:** 460, F10372. Cliffs with something resembling this species were also seen from the boat in the north arm of Dundas Bay, but were not accessible.
*Caloplaca fuscorufa* H. Magn. — Saxicolous in the alpine zone, 907–918 m. **EX:** 373, S38386; 406, S38780; 454, S39387. First reported as new for North America from Lake Clark by McCune *et al.* (2018). Sequences from S39387 are available in GenBank: ITS sequences MF114598; Beta-tubulin MF115104; mitochondrial LSU MF114860 (Vondrák *et al.* 2019*b*).
*Caloplaca holocarpa* (Hoffm.) A. E. Wade (syn. *Athallia holocarpa* (Hoffm.) Arup *et al.*) — Saxicolous on soft argillite in the alpine zone, at least once associated with a bird perch, 890–922 m. **EX:** 407, F10131 (sub *Protoparmeliopsis muralis*), S38839; 453, F10322 (sub *Tephromela atra*), F10323; 455, S39432. Two DNA sequences (isolate T1228 from S39432 = KR902672 and T1233 from S38839 = KR902671) were published by Vondrák *et al.* (2016*a*, as *Athallia holocarpa*).
*Caloplaca kamtczatica Savicz — Corticolous on *Alnus*, *Picea sitchensis* and *Tsuga heterophylla*, beaches or headlands, 9–27m. **DUN:** 339, S38162, S38195; 463, S39513. We obtained DNA from one specimen (Table 1).
*Caloplaca litoricola* Brodo — Saxicolous on rocky headland, 9 m. **DUN:** 463, F10392 (sub *Herteliana alaskensis*), S39571, S39574.
*Caloplaca nivalis* (Körb.) Th. Fr. — Muscicolous over granitic rock in the alpine zone, 460 m. **DUN:** 121, F10179.
*Caloplaca persimilis* Wetmore (syn. *Gyalolechia persimilis* (Wetmore) Søchting *et al.*) — Corticolous on *Cupressus nootkatensis*, 68 m. **DUN:** 413, S38925; 567, T41766. A previously published ITS rDNA sequence from GLBA (KT804988 from S38925) places this specimen within the *Caloplaca persimilis* group (Vondrák *et al.* 2016*b*) where it clusters with a taxon described as *C. ussuriensis* from the Russian Far East. However, it is unclear if this will resolve as a species distinct from *C. persimilis*.
*Caloplaca sinapisperma* (Lam. & DC.) Maheu & A. Gillet (syn. *Bryoplaca sinapisperma* (Lam. & DC.) Søchting *et al.*) — Muscicolous or on organic accumulations over limestone, beaches and outcrops, 0–52 m. **GB:** 867, S36441, S36443 (without anthraquinones!); 382, S38480; **WA:** 105, F9995. We obtained DNA from two specimens (Table 1).
*Caloplaca sorocarpa* (Vain.) Zahlbr. — Corticolous on *Alnus* and *Salix*, 10 m. **GB:** 866, S36420, S36425 (sub *Nephroma resupinatum*); **WA:** 384, S38507; 544, T41605; 206, M2503.
*Caloplaca stillicidiorum* (Vahl) Lynge — Growing on *Dryas* detritus and bryophytes, 0–22 m. **WA:** 105, F9995 (sub *Caloplaca sinapisperma*); 318, S37902.
*Caloplaca tetraspora* (Nyl.) H. Olivier (syn. *Bryoplaca tetraspora* (Nyl.) Søchting *et al.*) — Terricolous on organic accumulations over rock outcrops, 10–100 m. **WA:** 101, F9903 (sub *Scytinium imbricatum*).
*Caloplaca tiroliensis* Zahlbr. (syn. *Parvoplaca tiroliensis* (Zahlbr.) Arup *et al.*) — Terricolous/muscicolous in alpine heath, 922 m. **EX:** 407, F10125. Verified by an ITS rDNA sequence, obtained too late to be included in the present GenBank submission.
*Caloplaca tornoënsis* H. Magn. — Saxicolous on small rock in snowbed, 830 m. **EX:** 404, S38753. DNA sequences from KLGO (S29473 = MG954221 and S26816 = MG954220) are published in Vondrák *et al.* (2019*a*).
*Calvitimela aglaea* (Sommerf.) Hafellner — Saxicolous on argillitic rocks in the alpine zone, 883–922 m. **EX:** 370, S38341 (sub *Melanelia hepatizon*); 373, S38382; 405, F10101; 407, S38829, S38830; 455, S39433; 218, M2655b. TLC: atranorin.
*Calvitimela perlata* (Haugan & Timdal) R. Sant. — Saxicolous on exposed alpine summit, 618 m. **DUN:** 428, S39024. This species was reported as new to North America based on material from GLBA and KLGO (Bendiksby *et al.* 2015); previously known from Norway.
*Candelariella arctica* (Körb.) R. Sant. — Saxicolous on rocky seashore, 27 m. **DUN:** 339, 38204 (det. M. Westberg). An uncommon species reported in AK from the Bering Sea and several locations in inland western AK (Thomson 1997). A 19th century report from Baranof Island under the name *Placodium crenulatum* (Cummings 1904) could refer to this species but should be checked.
*Candelariella efflorescens* R. C. Harris & W. R. Buck — Corticolous and muscicolous over *Alnus* and *Populus balsamifera*, 0–22 m. **EA:** 440, S39233, S39260; **GB:** 864, S36336; S36339 (sub *Micarea peliocarpa*); Marble Mtn, S38012; **GUS:** Tower Rd, S40731.
*Candelariella vitellina* (Hoffm.) Müll. Arg. — Saxicolous on bird perches, 125–942 m. **EX:** 408, S38865; 459, F10360 (as ‘aff.’); **WA:** 388, S38561. F10360 is parasitic on *Placynthium*. The occurrences on small cyanolichens deserve further study; *C. vitellina* was also recorded as parasitic on *Tingiopsidium* in KLGO (Spribille *et al.* 2010).
#*Capronia peltigerae* (Fuckel) D. Hawksw. — Lichenicolous on *Peltigera*, terricolous on beach ridge, 4 m. **EA:** 438, S39185.
*#*Carbonea aggregantula* (Müll. Arg.) Diederich & Triebel — Lichenicolous on *Lecanora polytropa* group near glacier terminus, 15 m. **WA:** A571, P2287, P2314.
*Carbonea vorticosa* (Flörke) Hertel — Saxicolous on rocks in the alpine zone, 465–937 m. **DUN:** 426, S38991; **EX:** 407, S38826; 409, S38893; 454, S39405.
*Carneothele sphagnicola* Fryday, M. Svenss. & Holien — See ‘Descriptions of New Genera and Species’.
*Catapyrenium cinereum* (Pers.) Körb. — Terricolous on organic accumulations and open ground from supralittoral sea stacks to the alpine zone, 0–922 m. **DUN:** 463, F10387, F10400; **EX:** 407, S38852.
*Catapyrenium daedaleum* (Kremp.) Stein — Terricolous and muscicolous, 11–43 m. **EA:** 872, S36637; **WA:** 319, S37925; 381, S38470.
*Catillaria chalybeia* (Borrer) A. Massal. — Saxicolous in the supralittoral zone, in splash zone, on gneiss, 0–5 m. **DUN:** 134, F10461, F10462.
#*Catillaria stereocaulorum* (Th. Fr.) H. Olivier — Lichenicolous on *Stereocaulon* on *c.* 20 yr-old gravels, 0–43 m. **EA:** 869, S36552; Muir Inlet, S40768.
*Catinaria atropurpurea* (Schaer.) Vězda & Poelt — Corticolous on *Picea sitchensis* and *Populus balsamifera*, 0–10 m. **GB:** 864, S36366; **GUS:** 576, T41839; 230, M2764.
*Catolechia wahlenbergii* (Ach.) Körb. — On organic accumulations in the side of a granitic boulder by tarn inflow in a muskeg, 150 m. **DUN:** 118, F10152.
#*Cecidonia xenophana* (Körb.) Triebel & Rambold — Lichenicolous on *Porpidia macrocarpa* aggr. on rock, or on unspecified saxicolous lichen over granite, 100–903 m. **DUN:** 117, F10149; **EX:** 406, S38782 (sub *Lecidea swartzioidea*). DNA was obtained from S38782 (Table 1).
#*Cercidospora cephalodiorum* Triebel & Grube — Lichenicolous on *Pilophorus dovrensis* in alpine heath with rock outcrops, on alkaline argillite, 918 m. **EX:** 454, F10340 (sub *Pilophorus dovrensis*).
#*Cercidospora epipolytropa* (Mudd) Arnold — Lichenicolous on *Lecanora* spp., 0–871 m. **EX:** 458, S39483 (sub *Lecanora frustulosa*); **WA:** 381, S38465 (sub *Lecanora polytropa*); 391, S38619, S38637 (sub *Lecanora polytropa*); A571, P2287; A576, P2186.
*Cetraria aculeata* (Schreb.) Fr. — Terricolous in the alpine zone and in water pools in muskeg near sea level, and once recorded lignicolous and fertile, 27–922 m. **DUN:** 428, 39000; 465, F10426, 39628; **EX:** 348, S38283 (fertile, lignicolous); 370, 38360; 405, F10080, F10093; 407, F10129; 408, S38870; 455, S39448; 459, F10358. The fertile specimen S38283, from wood in a muskeg, corresponds to the morphology of *Cetraria crespoae* (Barreno & Vázquez) Kärnefelt, the epiphytic form of *C. aculeata* originally described from Spain (Barreno & Vasquez 1981).
*Cetraria ericetorum* Opiz subsp. *reticulatum* (Räsänen) Kärnefelt — Terricolous in *Cassiope-Fauria* heath and alpine tundra, 484–922 m. **DUN:** 425, S38987; **EX:** 405, F10081; 407, S38843.
*Cetraria islandica* (L.) Ach. subsp. *crispiformis* (Räsänen) Kärnefelt — Terricolous in the alpine zone, 903 m. **EX:** 406, S38790.
*Cetraria islandica* subsp. *orientalis* (Asahina) Kärnefelt — Terricolous in the alpine zone, 894–918 m. **EX:** 372, S38374; 454, F10339 (sub *Toensbergia geminipara*).
*Cetraria laevigata* Rass. — Terricolous in the alpine zone, 872–895 m. **EX:** 370, S38352; 456, S39465.
*Cetraria nigricans* Nyl. — On small accumulations of soil over rock, 907 m. **EX:** 373, S38404.
*Cetrelia cetrarioides* (Duby) W. L. Culb. & C. F. Culb. — Corticolous on *Alnus*, 2 m. **EA:** 441, S39266; **GB:** S Sandy Cove, S40752. TLC: atr, perlatolic acid.
*Chaenotheca brunneola* (Ach.) Müll. Arg. — Lignicolous on snags or corticolous on *Picea* or *Tsuga*, 21–717 m. **EX:** 109, F10047; 357, S38304; 403, S38745; 433, S39093; **GUS:** [V329, S38059]; 204, S38442, S38443.
*Chaenotheca chrysocephala* (Turner ex Ach.) Th. Fr. — Corticolous on *Pinus contorta* in a muskeg, also lignicolous, 0–5 m. **DUN:** 421, S38961 (sub *Chaenotheca ferruginea*); **GUS:** 107, F10027; 230, M2762; 397, S38716 (sub *Chaenotheca ferruginea*).
*Chaenotheca ferruginea (Turner ex Sm.) Mig. — Corticolous on rain-shaded parts of large *Picea* or *Tsuga* trunks, or lignicolous, 248–396 m. **DUN:** 421, S38961; **EX:** 213, M2569; 354, S38294, S38296 (sub *Chaenothecopsis aeruginosa*); **GUS:** 397, S38716.
*Chaenotheca furfuracea* (L.) Tibell — Lignicolous on a *Picea* snag, also terricolous on rock and detritus of tip-up, 58–569 m. **EX:** 358, S38328; **GUS:** 878, S36773.
*Chaenotheca gracillima* (Vain.) Tibell — Lignicolous on snags, or fungicolous on *Trichaptum abietinum*, in mixed woodland by creek, 44–569 m. **EX:** [125, F10267 (on *Trichaptum*)]; [859, F9658 (on lignum)]; 358, S38325; 398, S38722, S38723, S38725. The specimen from *Trichaptum* was compared to *Chaenotheca balsamconensis*, J. L. Allen & McMullin a species described from North Carolina that occurs exclusively on *Trichaptum* (Allen & McMullin 2015), but differed in possessing a brown, KOH− stalk.
*Chaenotheca trichialis* (Ach.) Hellb. — On polypore on *Tsuga* snag and corticolous on *Tsuga*, 59–569 m. **EX:** [860, S36257]; 358, S38327 (sub *Chaenotheca chrysocephala*).
*Chaenotheca xyloxena* Nádv. — Lignicolous on snag, 58 m. **GUS:** 878, S36788.
*+*Chaenothecopsis aeruginosa* Goward & E. B. Peterson — Corticolous on *Tsuga*, 222–396 m. **DUN:** 469, S39697; **EX:** 313, S37840, S37845; 354, S38296.
#*Chaenothecopsis arthoniae* Tibell — Lichenicolous on *Arthonia arthonioides*, corticolous on *Malus fusca* and *Tsuga heterophylla*, 9–222 m. **DUN:** between 462 and 469, S39684; **EX:** 313, S37842; [226, M2731]; 358, S38330; 448, S39347 (sub *Arthonia arthonioides*); **GUS:** 855, S36051. First reported for Alaska by Dillman *et al.* (2012) from KLGO.
#*Chaenothecopsis consociata* (Nádv.) A. F. W. Schmidt — On *Chaenotheca trichialis* over *Tsuga* bark, 569 m. **EX:** 358, S38327 (sub *Chaenotheca chrysocephala*).
*#*Chaenothecopsis lecanactidis* Tibell — On *Lecanactis* on sheltered trunk of *Tsuga*, 569 m. **EX:** 358, S38329. Recently reported as new to North America from Washington (Hardman *et al.* 2017).
+*Chaenothecopsis nana* Tibell — Corticolous on *Picea* and *Tsuga* bark, 21–396 m. **EX:** 354, S38295; 433, S39092.
+*Chaenothecopsis pusiola* (Ach.) Vain. — Lignicolous on snag, 58 m. **GUS:** 878, S36788 (sub *Chaenotheca xyloxena*).
+*Chaenothecopsis savonica* (Räsänen) Tibell — Lignicolous on small snag, 222 m. **EX:** 313, S37843 (as ‘aff.’).
+*Chaenothecopsis tasmanica* Tibell — Corticolous on *Picea* trunk, 124 m. **DUN:** 334, S38102 (sub *Calicium viride*); **EX:** 313, S37842.
*+*Chaenothecopsis viridireagens* (Nádv.) A. F. W. Schmidt — Lignicolous on small snag, 222 m. **EX:** 313, S37843 (sub *Chaenothecopsis* aff. *savonica*); 354, S38295 (sub *Chaenothecopsis nana* s. lat.).
*Cheiromycina petri* D. Hawksw. & Poelt — Corticolous on *Alnus*, 2 m. **GB:** 865, S36401. Recently reported as new for Alaska by McCune *et al.* (2018).
#*Cirrenalia lichenicola* Pérez-Ort. — See ‘Descriptions of New Genera and Species’.
*Cladonia amaurocraea* (Flörke) Schaer. — Terricolous in the alpine zone, 872–922 m. **EX:** 370, S38355; 407, S38851; 456, S39467. TLC: usnic and barbatic acids.
*Cladonia arbuscula* (Wallr.) Flot. subsp. *beringiana* Ahti — Terricolous on damp sandy soil, wet muskeg and in alpine tundra, 20–922 m. **DUN:** 338, S38145; 464, S39621, S39622; **EX:** between 405 and 406, S38775 (det. T. Ahti); 407, S38840; 453, S39371, S39374 (det. T. Ahti, as ‘cf. *arbuscula*’); 456, S39470; **GUS:** Tower Rd, S37518 (as ‘cf.’), S37531. TLC: usnic acid, fpc.
*Cladonia asahinae* J. W. Thomson — Terricolous on organic accumulations over rock in low elevation snowbed, 92 m. **WA:** 387, S38551. TLC: fatty acid, fpc.
*Cladonia bellidiflora* (Ach.) Schaer. — Terricolous in heath and muskeg, also seen corticolous (on *Pinus contorta*) and on stumps, 20–937 m. **DUN:** 338, S38141; 464, F10423; **EX:** [858, S36214]; [859, S36235]; 375, S38427; nr 409, S38902; 453, S39376. TLC: squamatic acid.
*Cladonia borealis* S. Stenroos — Terricolous in grassy sandy soil in the Gustavus outwash plain, also in recently deglaciated terrain, 0–33 m. **EA:** Muir Inlet, S40780; **GUS:** State Dock, S36851; Tower Rd, S37515. TLC: usnic acid, barbatic + sat, fatty acid.
*Cladonia cariosa* (Ach.) Spreng. — Terricolous at base of *Pinus contorta* in open flats and over marble outcrop, 0–43 m. **EA:** Muir Inlet, S40775, S40781; **GB:** 867, S36453; **GUS:** 317, S37893 (s. lat.); **WA:** 319, S37944. TLC: fpc but little material used for assay (atr cannot be ruled out).
*Cladonia carneola* (Fr.) Fr. — a) chemotype 1: terricolous and once lignicolous on burl of old *Pinus contorta*, 0–43 m. **GUS:** 876, S36757; Tower Rd, S37514; **WA:** 319, S37950. TLC: usnic and isousnic acids, zeorin; b) chemotype 2: terricolous in upper beach meadow to muscicolous over boulders and corticolous on *Tsuga mertensiana* in subalpine snowbeds, 0–788 m. **DUN:** 425, S38984; **EX:** 460, S39496; **GUS:** 437, S39171. TLC: isousnic/usnic and barbatic acids, zeorin; c) chemotype not determined: terricolous and corticolous, 0–10 m. **DUN:** 464, F10413; **GUS:** 316, S37888; **WA:** 102, F9934.
[*Cladonia cenotea* (Ach.) Schaer. — Terricolous in grassy meadow, 3 m. **GUS:** State Dock, S36853 (det. T. Ahti, sub *C. verruculosa*).]
*Cladonia chlorophaea* (Flörke ex Sommerf.) Spreng. — Terricolous and on organic accumulations over rocks and trees in muskeg and forest, 10–245 m. **EA:** Muir Inlet, S40782; **DUN:** 338, S38143; **EX:** 400, S38737; 457, S39478; **WA:** 319, S37926; 383, S38497. TLC: fpc.
*Cladonia coccifera* (L.) Willd. — Terricolous on organic accumulations along shorelines and in snowbeds, 0–115 m. **EA:** between 870 and 871, S36591; **WA:** 102, F9933; 387, S38549. TLC: isousnic/usnic acid, zeorin.
*Cladonia concinna Ahti & Goward — Terricolous on sandy soil of Gustavus outwash plain, also over a boulder in snowbed, 33–92 m. **GUS:** Tower Rd, S37533 (conf. T. Ahti); **WA:** 387, S38549 (sub *Cladonia coccifera*). Described from the west coast of Vancouver Island (Ahti 2007).
*Cladonia coniocraea* (Flörke) Spreng. — Terricolous in upper beach meadows, corticolous on *Alnus*, lignicolous on *Pinus contorta*, also over moss and on fine *Tsuga heterophylla* twigs, 0–213 m. **DUN:** 462, S39708; **EX:** 312, S37830; [858, S36225]; **GB:** 864, S36396 (sub *Cladonia fimbriata*); 874, S36718 (sub *Cladonia umbricola*); **GUS:** 397, S38705; 437, S39170; 878, S36787. TLC: fpc.
[*Cladonia conista* (Nyl.) Robbins — Terricolous on sandy soil, 3 m. **GUS:** State Dock, S36848. TLC: fpc, bourgeanic acid.]
*Cladonia cornuta* (L.) Hoffm. — Terricolous on sandy soils, 10–33 m. **EA:** terminus of Riggs Glacier, S40808; **GUS:** Tower Rd, S37540; **WA:** 384, S38528.
*Cladonia crispata* (Ach.) Flot. var. *cetrariiformis* (Delise) Vain. — Terricolous in muskeg (even in standing water) and alpine tundra, 20–922 m. **DUN:** between 412 and 415, S38919; 338, S38142; 464, S39617, S39626; 465, S39630; **EX:** 110, F10059; 312, S37828; 371, S38366; 400, S38736; 407, S38845; 456, S39463; 457, S39479; **GUS:** Tower Rd, S37525-B. TLC: squamatic acid, ±barbatic acid.
*Cladonia crispata* var. *crispata* — Terricolous on tip-up in muskeg, 213 m. **EX:** 312, S37831 (ver. T. Ahti). TLC: squamatic acid.
*Cladonia ecmocyna* Leight. — Terricolous on ‘semi-open herbaceous ridge’, 610 m. **GB:** slopes and ridges of mainland immediately NW of Sebree Cove and W of Caroline Point, 1968, *Worley* 10533 (UBC, det. T. Goward). This species was not encountered during the present survey.
*Cladonia farinacea* (Vain.) Evans — Terricolous on mossy soil in forest on coastal headland, 17 m. **DUN:** 467, S39674 (det. T. Ahti). TLC: fpc. A distinctive and rare species in Alaska, until now known only from Kotzebue (Krog 1968) and Gates of the Arctic (Dillman *et al.* 2012).
*Cladonia fimbriata* (L.) Fr. — Terricolous on organic accumulations over rock, 10–903 m. **EA:** Muir Inlet, S40784; **EX:** 455, S39441; **GB:** 864, S36396, S36397 (sub *Cladonia pyxidata*); 865, S36411; **GUS:** 437, S39169 (sub *Cladonia pyxidata*); **WA:** 383, S38501. TLC: fpc.
*Cladonia furcata* (Huds.) Schrad. — Terricolous on sandy road banks, all collections from the Gustavus outwash plain, 30–40 m. **GUS:** 880, S36820, S36821; Tower Rd, S37522, S37539. TLC: fpc.
*Cladonia gracilis* (L.) Willd. subsp. *elongata* (Wulfen) Vain. — Terricolous in wet muskeg and alpine snowbeds, 20–903 m. **DUN:** 464, S39618; 465, S39633; **EX:** 406, S38798 (ver. T. Ahti), S38799; nr 455, S39458; 456, S39460 (dwarf morph); **GUS:** Tower Rd, S37524, S37525. TLC: fpc.
*Cladonia gracilis* subsp. *turbinata* (Ach.) Ahti — Terricolous on sandy soil, 33 m. **GUS:** Tower Rd, S37526.
*Cladonia gracilis* subsp. *vulnerata* Ahti — Terricolous in muskeg, 226 m. **DUN:** 338, S38146 (det. T. Ahti).
*Cladonia kanewskii* Oxner — Terricolous, 50 m. **DUN:** between 412 and 415, S38922. TLC: usnic acid, fatty acids.
*Cladonia maxima* (Asahina) Ahti — Terricolous in muskegs and wet headland forest, 10–222 m. **DUN:** 467, F10439, S39675; **EX:** 312, S37827.
*Cladonia mitis* Sandst. — Terricolous on sandy soil and alpine tundra, 0–903 m. **EX:** 312, S37822; 372, S38375; 406, S38792; 408, S38867; **GUS:** State Dock, F9870. TLC: usnic acid, unknown *R*_f_ A1/B5/C1-2.
[*Cladonia norvegica* Tønsberg & Holien — Corticolous on *Picea sitchensis* log in *Picea* forest, 19 m. **GUS:** 557, T41717. TLC: barbatic and 4-*O*-demethylbarbatic acids.]
*Cladonia ochrochlora* Flörke — Terricolous on sandy road cut and on organic or woody accumulations over rock, 0–39 m. **EA:** Muir Inlet, S40769; **GB:** S Sandy Cove, S40759; **GUS:** 100, F9891; 880, S36822 (det. T. Ahti); **WA:** 384, S38527 (det. T. Ahti). This species, which is ubiquitous further inland in NW North America, appears to be uncommon in GLBA.
*Cladonia phyllophora* Hoffm. — Terricolous on sandy lowland soil and in alpine tundra, 33–942 m. **EX:** 408, S38871; **GUS:** Tower Rd, S37521, S37534. TLC: fpc.
*Cladonia pocillum* (Ach.) Grognot — Terricolous over limestone, 33–52 m. **WA:** 382, S38479; E slopes of Marble Mtn opposite Drake Island, 9 August 1968, *I. A. Worley* 11234 (UBC, det. C. R. Björk). Not seen during the present survey.
*Cladonia portentosa* (Dufour) Coem. subsp. *pacifica* Ahti — Terricolous in muskeg, 27–149 m. **DUN:** 415, S38935; 416, S38945. TLC: usnic and perlatolic acids.
*Cladonia pseudalcicornis* Asahina — Terricolous over boulder in snowbed *Salix*/*Shepherdia* scrub, 92 m. **WA:** 387, S38548, S38552. TLC: fpc.
*Cladonia pseudoevansii* Asahina — Terricolous in wet muskeg, 25 m. **DUN:** 465, F10425, S39629, S39631 (both det. T. Ahti). Collection S39629 represents an usnic acid-free strain that co-occurred with specimens with the typical usnic acid-containing chemosyndrome.
*Cladonia pyxidata* (L.) Hoffm. — Terricolous on organic accumulations on logs, tip-ups, over boulders, on road cuts, in snowbeds and directly on soil, 0–907 m. **DUN:** 462, S39747; **EA:** 869, S36568; 312, S37829; **EX:** 358, S38316; 373, S38401; 404, S38757; 456, S39461; **GB:** 864, S36391; 873, S36662, S36664, S36668; **GUS:** 880, S36824; 437, S39169; **WA:** 319, S37946; 322, S37962; 383, S38486; 387, S38547; 864, S36397. TLC: fpc.
*Cladonia rangiferina* (L.) F. H. Wigg. — Terricolous in muskeg, heath and on sandy soil, 0–937 m. **DUN:** 337, S38127; 338, S38144; between 412 and 415, S38920 (PD+ orange); 416, S38946; 464, S39620; **EX:** 312, S37825, S37826; 453, S39377, S39378; 409, S38896; **GUS:** Tower Rd, S37536; State Dock, F9871. TLC: atr, fpc.
*Cladonia rei* Schaer. — Terricolous on beach ridges and old gravels, 0–43 m. **EA:** 438, S39183, S39183-B; 869, S36547; 869, S36565; Muir Inlet, S40779, S40786; terminus of Riggs Glacier, S40797; **GB:** 864, S36395; 873, S36670; **GUS:** 876, S36755; **WA:** 325, S37983; 383, S38498. TLC: homosekikaic acid, fpc.
*Cladonia scabriuscula* (Delise) Nyl. — Terricolous on sandy soil or on organic accumulations on logs and stumps, 0–713 m. **DUN:** 462, S39698, S39748 (conf. T. Ahti); **EX:** [859, S36233]; above ‘Mooselator’, S38903 (conf. T. Ahti); **GUS:** State Dock, S36857. TLC: fpc.
*Cladonia squamosa* Hoffm. — On organic accumulations over rock, stumps, soil, *Picea* and *Tsuga* trunks and logs, and in alpine heath, 0–872 m. **DUN:** 413, S38932; 416, S38944; 423, S38979; **EX:** [858, S36213]; [859, S36233 (sub *Cladonia scabriuscula*)]; 448, S39352; 456, S39464; 460, S39495 (det. T. Ahti: ‘arctic morph’ with fpc!); **GB:** 874, S36716; **GUS:** 855, S36012. TLC: squamatic or squamatic and barbatic acids (tr) (2×) or squamatic acid and fpc (1×).
*Cladonia stellaris* (Opiz) Pouzar & Vězda — Terricolous in heath 883–922 m. **EX:** 371, S38364 (s. lat.); 405, F10082; 406, S38793; 407, F10104, S38842; 453, S39373, S39379. TLC: usnic, perlatolic acids.
*Cladonia straminea* (Sommerf.) Flörke — Lignicolous on wooden stick in muskeg, 294 m. **EX:** 352, S38286 (as *C. metacorallifera*). TLC: usnic, didymic, squamatic acids.
*Cladonia stricta* (Nyl.) Nyl. — Terricolous on sandy soil, 33 m. **EX:** 406, S38798 (det. T. Ahti; sub *C. gracilis* subsp. *elongata*); **GUS:** Tower Rd, S37529, S37542 (det. T. Ahti). TLC: fpc.
*Cladonia stygia* (Fr.) Ruoss — Terricolous in the alpine zone, 922 m. **EX:** 407, S38846 (ver. T. Ahti). TLC: atr, fpc.
*Cladonia subfurcata* (Nyl.) Arnold — Terricolous in wet muskeg and on damp sand, 20–236 m. **DUN:** 464, S39627; **EX:** 399, S38219; **GUS:** Tower Rd, S37517. TLC: squamatic acid.
*Cladonia subulata* (L.) F. H. Wigg. — Terricolous on sandy soil, 20–33 m. **GUS:** Tower Rd, S37535 (ver. T. Ahti); **WA:** 383, S38482 (det. T. Ahti). TLC: fpc.
*Cladonia sulphurina* (Michx.) Fr. — Terricolous over rock, 10 m. **WA:** 383, S38503.
*Cladonia symphycarpa* (Flörke) Fr. — Terricolous on organic/mineral accumulations over rock and on sandy road cut, 22–903 m. **EX:** 406, S38797; **GUS:** 880, S36823; **WA:** 318, S37912; 319, S37943; 382, S38481. TLC: atr, norstictic acid.
*Cladonia trassii* Ahti — Terricolous on sandy soil, 33 m. **GUS:** Tower Rd, S37519. TLC: atr, fpc.
*Cladonia turgida* Hoffm. — Terricolous in compact muskeg-meadow, 100 m. **DUN:** 418, S38951 (ver. T. Ahti), S38952. TLC: atr. A rare species in Alaska and western North America. We found a single but conspicuous patch in the Dundas Basin.
*Cladonia umbricola* Tønsberg & Ahti — The common red-fruited *Cladonia* species on tree trunks and stumps at low elevations. Chemotype 1 (squamatic acid): at base of tree, 59 m. **EX:** [860, 36237 (together with chemotype 2)]. Chemotype 2 (squamatic acid plus usnic acid visible in all samples but often in too low concentrations to detect by TLC): lignicolous on rotten stumps and logs and corticolous or muscicolous over *Picea* and *Tsuga* tree trunks, 0–162 m. **EX:** [858, S36206]; [860, S36237, S36238 (det. T. Ahti)]; **GB:** 874, S36714, S36718 (as ‘cf.’); N Sandy Cove, S36650; 874, S36719; **GUS:** 856, S36076; 316, S37862; 855, S36020; 876, S36742; Tower Rd, S37504. Chemotype 4 (usnic, thamnolic acids): lignicolous on stump, 44 m. **EX:** [859, S36234]. Chemotype not determined: moss/bark over base of *Picea sitchensis*, and side of mossy log. **GUS:** 107, F10036. Chemotypes numbered after Goward (1999).
*Cladonia uncialis* (L.) F. H. Wigg. (incl. subsp. *biuncialis* (Hoffm.) M. Choisy) — Terricolous on sandy soils, in muskeg and in alpine heath, 27–889 m. **DUN:** 338, S38140; between 412 and 415, S38921; 465, S39632; 131, F10441; **EX:** 312, S37823; 453, S39375; **GUS:** Tower Rd, S37527, S37538. TLC: usnic, squamatic acids.
*Cladonia verruculosa* (Vain.) Ahti — Terricolous on young soils in glacial forelands and mineral accumulations over rock, 10–115 m. **EA:** between 870 and 871, S36592 (ver. T. Ahti); [**GUS:** State Dock, S36853 (ver. T. Ahti)]; **WA:** 383, S38499 (ver. T. Ahti); 387, S38550 (ver. T. Ahti). TLC: fpc.
*Cladonia wainioi* Savicz — Terricolous in wet muskeg, 20–213 m. **DUN:** 464, F10416, S39624, S39625; **EX:** 312, 37824. TLC: atr, merochlorophaeic acid.
*Clauzadea monticola* (Schaer.) Hafellner & Bellem. — Saxicolous on limestone, including pebbles, underhangs and larger rocks, 0–886 m. **EX:** 217, M2594; **GB:** 863, F9693, F9696, F9703; 864, F9728, S36388; **WA:** 101, F9912, F9925; 105, F9984; 383, S38489; 391, S38622, S38624; 392, S38645; 209, M2527; waypoint not recorded, M2534.
*Cliostomum griffithii* (Sm.) Coppins — Corticolous and lignicolous on *Cupressus nootkatensis* and *Picea sitchensis*, always near the seashore, 12–68 m. **DUN:** 413, S38927 (aff.); 572, T41793 (poor material); **EX:** 433, S39069; **GB:** 868, S36530; **GUS:** 397, S38694. TLC: atr, roccellic acid, except S38927 roccellic only, poor material but morphologically similar to *C. griffithii*.
*Cliostomum leprosum* (Räsänen) Holien & Tønsberg — Corticolous on *Tsuga heterophylla* and *T. mertensiana*, especially on veteran trees, 20–396 m. **DUN:** 332, S38068; 334, S38094; 467, S39657; 131, F10444; 563, T41757; 565, T41761; **EX:** [860, F9665, S36246]; 313, S37835, S37837; 354, S38291, S38292; 433, S39084; waypoint not recorded, M2588; **GUS:** 876, S36738. TLC: atr only.
**#*Clypeococcum placopsiiphilum* Øvstedal & D. Hawksw. — Lichenicolous on *Placopsis* sp. on sea stacks, 2 m. **DUN:** 586, P2164. Previously known from Antarctica (Øvstedal & Hawksworth 1986), Russia (Zhurbenko 2009) and Iceland (Berger 2000; Brackel 2010).
[*Coccotrema hahriae* T. Sprib. & Tønsberg — Corticolous on *Picea sitchensis* branches, 33 m. **GUS:** 329, S38058; 557, T41702. TLC: stictic, norstictic (tr) acids. This is the first record of the species outside of KLGO (Spribille *et al.* 2010).]
*Coccotrema pocillarium* (Cumm.) Brodo — Corticolous on *Alnus*, *Cupressus nootkatensis*, *Picea sitchensis* and *Tsuga heterophylla*, usually close to seashores but once (Excursion Ridge) in a high montane forest; 0–432 m. **DUN:** 114, F10137; 129, F10363; 333, S38088; 339, S38155, S38176; 412, S38916; 462, S39502; 466, S39636; 467, F10431, F10437, S39664, S39667; 560, T41737; 561, T41741; 573, T41802; **EX:** 108, F10039; 355, S38298; **GB:** 868, S36456, S36472, S36509, S36521; **GUS:** 341, S38230; 436, S39142, S39157. TLC: stictic acid, ±sats.
*Coenogonium pineti* (Ach.) Lücking & Lumbsch — Corticolous on *Picea sitchensis* and *Populus balsamifera*, 1–20 m. **GUS:** 434, S39097; 341, S38242. DNA sequences from S39097 were published by Resl *et al.* (2015).
*Collema furfuraceum* (Arnold) Du Rietz — Corticolous on *Populus balsamifera* trunk, 25 m. **GUS:** Tower Rd, S40727.
*Collema glebulentum* (Nyl. ex Cromb.) Degel. — Terricolous on soil over rock at edge of creek and saxicolous over basic rocks, 219–918 m. **EX:** 128, F10288; 310, S37807; 406, S38781; 407, S38833; 454, S39389; **GB:** 864, S36383.
*Collemopsidium sublittorale* (Leight.) Grube & B. D. Ryan — Saxicolous on limestone rocks on shore, 0–5 m. **GB:** 863, F9706.
*Cornicularia normoerica* (Gunn.) Du Rietz was originally reported by Macoun (1902) as having been collected by Trevor Kincaid on 13 July 1897 at Muir Glacier. There is no indication that the material was seen in later studies (e.g. Krog 1968; Kärnefelt 1986). Attempts to locate the material in North American herbaria have been unsuccessful, and it is possible that the identification was revised and the material is now filed under another name. Collections by Trevor Kincaid from other parts of Alaska (St George Island, St Paul Island) from late July and August 1897 are filed at the University of Michigan herbarium, but there is no record of this specimen in the Consortium of North American Lichen Herbaria (https://lichenportal.org/cnalh/). This is the only reported location from Alaska, although its occurrence in GLBA would not be implausible: the species is known from adjacent regions of northern British Columbia. We suspect that the *Cornicularia normoerica* specimen reported by Macoun (1902) could be the same reported as *Pseudephebe pubescens* by Degelius (1937), though there is nothing on the packet at UPS to indicate an earlier identification as *C. normoerica*. There is also a possibility that the original report by Macoun (1902) was itself a clerical error; Macoun's reports on his own collections are known to be full of errors and impossible records (Godfrey 1977).
*#*Cornutispora lichenicola* D. Hawksw. & B. Sutton — Lichenicolous in apothecia of *Micarea* and on *Lepra subvelata* and *Pertusaria subambigens*, 15–20 m. **GUS:** 434, S39101; A569, P2300, P2384.
#*Corticifraga fuckelii* (Rehm) D. Hawksw. & R. Sant. — Lichenicolous on *Peltigera*, terricolous or muscicolous over marble, 0–125 m. **GB:** 867, S36438; **WA:** 388, S38554; 389, S38579.
#*Corticifraga nephromatis* Pérez-Ort. — See ‘Descriptions of New Genera and Species’.
#*Corticifraga scrobiculatae* Pérez-Ort. — Lichenicolous on *Lobarina scrobiculata*, on *Alnus*, 8 m. **EA:** 440, S39258.
*Cryptothele neglecta Henssen — Saxicolous on small siliceous cobble deposited over limestone, 15 m. **WA:** 381, S38467. A distinctive species with >50 ascospores per ascus, 3.5–4.2 × 2.5 μm, previously known only from Sweden (Jørgensen 2007). The specimen matches material from the type locality (Sweden, [Närke], Askersund, Lind, 1870, *Blomberg* s. n., UPS!).
*Cystocoleus ebeneus* (Dillwyn) Thwaites — Saxicolous on the side of granitic boulder, 20–25 m. **GUS:** 142, F10497.
*Dendriscosticta wrightii* (Tuck.) Moncada & Lücking — A symbiodeme-forming fungus that occurs in the form of two lichen symbioses in GLBA: a) chloromorph: corticolous on *Alnus*, 0–46 m. **EA:** nr 438, S39297; **GB:** N Sandy Cove, F9816, S36654; **GUS:** 876, S36723; 879, S36811; 881, S36830, S36833; [V329, S38016]; [557, T41706, T41710]; 559, T41727; b) cyanomorph (‘*Dendriscocaulon*’): corticolous on *Alnus*, 5–10 m. **EA:** 123, F10239; **GB:** S Sandy Cove, S40748; **GUS:** 111, F10061.
*Dibaeis baeomyces* (L. f.) Rambold & Hertel — Terricolous, forming sterile white crusts, found only in the Dundas Basin, 149–465 m. **DUN:** nr 416, S38948; 426, S38996. Rarely fruiting in SE Alaska (recently found with apothecia near Mendenhall Glacier). DNA from a GLBA specimen was published by Spribille *et al.* (2014*b*, as isolate P80).
*#*Didymellopsis pulposi* (Zopf) Grube & Hafellner — Lichenicolous on *Leptogium*, on organic accumulations over rock, 22 m. **WA:** 318, S37917. One previous record from North America, from the Northwest Territories (Zhurbenko 2013).
*Enchylium tenax* (Sw.) Gray — Muscicolous over siliceous rock, 0–10 m. **GB:** 868, S36542.
#*Endococcus propinquus* (Körb.) D. Hawksw. — Lichenicolous on saxicolous crustose lichen, alpine zone, 832 m. **EX:** 216, M2583.
#*Endococcus rugulosus* Nyl. — Lichenicolous on *Aspicilia*, saxicolous in snowbed, 871 m. **EX:** 458, S39491.
*Ephebe lanata* (L.) Vain. — Saxicolous on rocks at edge of lake, and on mountain top, 127–618 m. **DUN:** 419, S38955; 428, S39020.
#*Epicladonia sandstedei* (Zopf) D. Hawksw. — Lichenicolous on *Cladonia pyxidata*, 2 m. **WA:** A573, P2156, P2368.
*#*Epicladonia stenospora* (Harm.) D. Hawksw. — Lichenicolous on *Cladonia*, terricolous, 894 m. **EX:** 372, S38372.
*Epigloea medioincrassata* (Grummann) Döbbeler — On algal biofilm on dead mosses. **EX:** 565, P2236.
**#*Epigloea urosperma* Döbbeler — Lichenicolous on *Placynthiella uliginosa*, muscicolous over log in upper beach meadow, 2 m. **GUS:** 437, S39166. This species appears to be restricted to *Placynthiella* species. In addition to being widely reported in Europe, it was also recently reported from Bolivia and the Seychelles (Diederich *et al.* 2017).
#*Epilichen scabrosus* (Ach.) Clem. — Lichenicolous on *Baeomyces rufus*, terricolous, 0–115 m. **EA:** 869, S36561; 870, F9780 (sub *Baeomyces rufus*).
*Erinacellus dendroides* (Henssen) T. Sprib. *et al*. — Corticolous on *Pinus contorta* (most records) and on *Picea sitchensis* (1×), 18–32 m. **GUS:** 857, S36105; 862, S36292, S36300, S36301; 316, S37872. GLBA material was the basis of a molecular study establishing this as a new genus; it was previously placed in *Spilonema* (Spribille *et al.* 2014*a*, isolates L1728, L1729).
*Euopsis granatina* (Sommerf.) Nyl. — Saxicolous on pebbles and outcrops, 0–871 m. **DUN:** 428, S39021; **EX:** 455, S39435 (sub *Atrophysma cyanomelanos*); 458, S39484 (sub *Lecidea phaeops*); 218, M2655a; **WA:** 105, F9991.
*Euopsis pulvinata* (Schaer.) Vain. — Saxicolous, alpine zone, 871–907 m. **EX:** 373, S38381; 455, S39430; 458, S39494.
*Farnoldia jurana* (Schaer.) Hertel — Saxicolous on pebbles and underhangs, 10–43 m. **WA:** 319, S37938; 383, S38504 (sub *Lecidella* aff. *carpathica*).
*Felipes leucopellaeus* (Ach.) Frisch & G. Thor (*Arthonia leucopellaea* (Ach.) Almq.) — Corticolous on *Tsuga heterophylla* bark, 222 m. **EX:** 313, S37838; 314, S37848.
*Fellhanera bouteillei (Desm.) Vězda — Corticolous on *Picea sitchensis* and *Salix* on upper beach, 2 m. **GUS:** 857, S36155; 435, S39120 (sub *Polycauliona pollinarioides*), S39124 (sub *Scoliciosporum chlorococcum*).
*Fellhaneropsis vezdae (Coppins & P. James) Sérus. & Coppins — Corticolous on *Alnus*, *Picea sitchensis*, *Ribes lacustre* and *Tsuga heterophylla* twigs, and fungicolous on *Fomitopsis* cf. *pinicola*, 1–164 m. **GUS:** 856, S36073; 878, S36768, S36771; 330, S38063; 341, S38254 (sub *Bacidina sulphurella*); 229, M2746.
*Fissurina insidiosa* C. Knight & Mitten — Corticolous on *Alnus* (mainly), also twice on *Tsuga heterophylla*, 9–86 m. **DUN:** 333, S38074, S38091 (sub *Sclerococcum fissurinae*); 431, S39035; 462, S39742; 562, T41743, T41752; **EX:** [125, F10257, F10258]; [859, F9657, S36228]; [860, F9664, S36239]; [V431, S39035, S39050 (sub *Sclerococcum fissurinae*)]. Described from New Zealand but apparently frequent in the south-eastern United States and also reported from Haida Gwaii, British Columbia (Brodo 1995, as *Graphis*). It is not known if the SE Alaskan material corresponds to the type. A set of DNA sequences has been published from GLBA (Resl *et al.* 2015).
*Flavocetraria cucullata* (Bellardi) Kärnefelt & A. Thell — Terricolous, 922–936 m. **EX:** 375, S38426; 407, S38849.
*Flavocetraria nivalis* (L.) Kärnefelt & A. Thell — Terricolous in alpine heath, 907–922 m. **EX:** 373, S38399; 407, S38858.
*Frutidella caesioatra* (Schaer.) Kalb — Muscicolous or loosely saxicolous on tops and sides of boulders, 150–484 m. **DUN:** 118, F10151; 119, F10157; 423, S38975 (sub *Rhizocarpon badioatrum*); 425, S38983.
*Frutidella furfuracea* (Anzi) M. Westb. & M. Svenss.(syn. *Lecidea pullata* (Norman) Th. Fr.) — Corticolous on *Picea sitchensis* krummholz and *Pinus contorta* in muskeg, 32–883 m. **EX:** 405, S38766; **GUS:** 862, S36294. TLC: sphaerophorin.
*Fuscidea intercincta* (Nyl.) Poelt — Saxicolous on granite and argillite in subalpine and alpine habitats, 460–918 m. **DUN:** 121, F10176; 428, S39022 (sub *Pyrenopsis* sp.); **EX:** 454, F10333, F10346 (sub *Pyrenopsis phaeococca*), F10344, S39405 (sub *Carbonea vorticosa*); 218, M2643.
*Fuscidea muskeg* Tønsberg & M. Zahradn. — See ‘Descriptions of New Genera and Species’.
*Fuscidea thomsonii* Brodo & V. Wirth — Saxicolous on gneiss in the supralittoral zone, 0–5 m. **DUN:** 462, F10369; 463, F10395, F10404.
*Fuscopannaria ahlneri* (P. M. Jørg.) P. M. Jørg. — Corticolous on *Alnus* and branches of *Picea sitchensis*, 20 m. **GUS:** [557, T41700]; 559, T41735 (conf. P. M. Jørgensen 2013).
[*Fuscopannaria confusa* (P. M. Jørg.) P. M. Jørg. — Corticolous on *Picea sitchensis* branches, 20 m. **GUS:** 557, T41711b (det. P. M. Jørgensen 2013).]
*Fuscopannaria convexa* P. M. Jørg. — Corticolous on *Alnus*, *Populus balsamifera* and *Salix*, mostly close to seashore, 0–32 m. **EA:** 440, S39249; 441, F10194, F10196; **GB:** 867, S36428; **GUS:** 862, S36309; [V329, S38033 (sub *Szczawinskia tsugae*)]; no waypoint, M2482; **WA:** 395, S38682, S38684 (sub *Fuscopannaria leucostictoides*).
*Fuscopannaria dillmaniae* T. Sprib. — See ‘Descriptions of New Genera and Species’.
*Fuscopannaria incisa* (Müll. Arg.) P. M. Jørg. — Corticolous on *Alnus*, 30 m. **EA:** 441, F10207, **GUS:** [V329, S38028].
*Fuscopannaria leucostictoides* (Ohlsson) P. M. Jørg. — Corticolous on *Alnus*, *Populus balsamifera* and *Salix*, in beach fringe habitats, 0–10 m. **EA:** 441, S39280; **GB:** 868, S36470; 874, S36695 (as ‘cf.’); **WA:** 391, S38589; 395, S38684 (as ‘cf.’).
*Fuscopannaria pacifica* P. M. Jørg. — Corticolous on *Alnus*, *Picea sitchensis*, *Populus balsamifera* and *Tsuga heterophylla*, 0–162 m. **EA:** 441, F10218; **EX:** [V431, S39044 (brown morph)]; **GUS:** 100, F9900; 138, F10479, F10481; 857, F9633; [V329, S38028 (s. lat.)], S38047, S38054; 855, S36011, S36030; 878, S36769; Tower Rd, S37505, S38277 (brown morph).
*Fuscopannaria praetermissa* (Nyl.) P. M. Jørg. — Terricolous on organic accumulations or muscicolous, also on sandy soil, along beaches and in alpine zone, 0–942 m. **EX:** 407, F10128 (pruinose morph); 408, S38868; 455, S39434, S39435 (sub *Atrophysma cyanomelanos*), S39443; **GB:** 868, S36539; [**GUS:** State Dock, F9875, S36858].
*Fuscopannaria ramulina* P. M. Jørg. & Tønsberg — Corticolous on *Alnus*, *Populus balsamifera* and *Salix*, 8–60 m. **EA:** 123, F10243; 872, S36605, S36611; 440, S39191 (sub *Hypotrachyna sinuosa*), S39246, S39253; 441, F10195, F10196 (sub *Fuscopannaria convexa*); **GB:** 868, S36459; **GUS:** 138, F10482; 857, S36093 (sub *Parmeliella triptophylla*), S36143; [557, T41701, T41715a]; [574, T41817]; 576, T41843; **WA:** 391, S38594, S38618; 542, T41589.
*Fuscopannaria sorediata P. M. Jørg. — Corticolous on *Alnus*, once on fine *Tsuga* twigs, 5–58 m. **GB:** N Sandy Cove, S36641; **GUS:** 878, S36760; 879, S36810 (aff.); 881, S36832. GLBA specimens appear to match *Fuscopannaria* sp. S24650, a species recorded in KLGO (Spribille *et al.* 2010). P. M. Jørgensen (personal communication to TS, 28 February 2012) concluded that *Fuscopannaria* sp. S24650 is identical to *F. sorediata*, a species he originally described from Tennessee (Jørgensen 2000). The relationship of these lichens to *Fuscopannaria alaskana*, from which no DNA sequences have been published, as well as to south-east Alaskan specimens of *F. ahlneri*, warrants systematic study.
#*Geltingia associata* (Th. Fr.) Alstrup & D. Hawksw. — Lichenicolous on *Ochrolechia subplicans*, 0–2 m. **DUN:** 586, P2193.
*Gowardia nigricans* (Ach.) Halonen *et al*. — Terricolous in alpine heath with rock outcrops, 883 m. **EX:** 405, F10094.
*Graphis scripta* (L.) Ach. — Corticolous on *Alnus*, 0–86 m. **DUN:** 333, S38080, 38084; 462, S39739; 562, T41748; **EA:** 440, S39221; **EX:** [860, S36241]; 433, S39075; **GB:** 868, S36467; N Sandy Cove, S36638; **GUS:** 857, F9640, S36083, S36145; [V329, S38023, S38035]; 330, S38060; 462, S39739; 531, T41526; 576, T41826; 224, M2832b. One specimen was used for DNA extraction (isolate T1176, Table 1).
*Gyalecta jenensis* (Batsch) Zahlbr. — Saxicolous on limestone and argillite rocks on seashore and creek banks, 0–155 m. **EX:** 444, S39330; **GB:** 863, F9698; 864, S36390. This species was found at two localities in GLBA. The material from limestone possesses deeply urceolate apothecia with widely opened discs; that from argillite, which occurred along a stream draining a large peatland, possesses apothecia with a narrow ostiole.
*Gyalectaria diluta* (Björk *et al.*) I. Schmitt *et al*. — Corticolous on *Alnus*, *Picea sitchensis*, *Tsuga heterophylla* and *Vaccinium ovalifolium*, lignicolous, and fungicolous on a polypore, 0–220 m. **DUN:** 462, S39725; **EX:** [860, S36255]; [225, M2717]; 236, M2831; **GUS:** 855, S36026, S36038; 874, F9832; 878, S36775; 882, S36843; 857, S36101, S36121; 341, S38237; no waypoint, M2477. A set of DNA sequences from a GLBA specimen has been published by Resl *et al.* (2015; isolate P145).
*Gyalidea fritzei (Stein) Vězda — Saxicolous in sheltered finger-sized depression in argillitic rock in alpine zone, 918 m. **EX:** 454, S39394.
*Gyalidea* aff. *lecideopsis* (A. Massal.) Lettau var. *eucarpa* (Servít) Vězda — Saxicolous on rock in stream, 225 m. **EX:** 236, S39048; 128, F10303-F10312; [225, M2715]; 236, M2830. Naming this material has been difficult owing to the lack of a global taxonomic study of *Gyalidea*. Clarifying the status of these collections would require checking about seven type specimens, a task beyond the scope of the present study. Sequences were obtained from S39048 (Table 1). *Gyalidea lecideopsis* var. *lecideopsis* has been reported from Haida Gwaii (Brodo 1995) and *G. lecideopsis* var. *convarians* (Nyl.) Vězda from the Bering Straits (Vězda 1966).
**Gyalidea subscutellaris (Vězda) Vězda — Terricolous on *c.* 20 yr-old glacial foreland soils, *Dryas*-dominated habitats, 0–115 m. **EA:** 869, S36566; 870, F9773. Described from *Bartramia* moss cushions in the High Tatra (Slovakia; Vězda 1960), and since reported from Poland, the UK and Sweden (summarized by Svensson *et al.* 2017).
*Gyalideopsis epicorticis* (A. Funk) Tønsberg & Vězda — Corticolous on *Alnus* bark and fine *Tsuga heterophylla* twigs, also once each on *Malus fusca* and *Salix*, mainly at low elevations (3–162 m) but also found once on *Picea sitchensis* in krummholz (922 m). **EX:** [125, F10278]; 407, S38817; **GB:** 874, S36709 (sub *Gyalideopsis piceicola*); **GUS:** [V329, S38024]; 855, S36004; 857, S36118 (sub *Gyalideopsis piceicola*), S36155 (sub *Fellhanera bouteillei*); [858, S36201]; 878, S36764, S36782; Tower Rd, S38275, S38282; [557, T41693]; 576, T41836; 230, M2749; 237, M2833.
*Gyalideopsis helvetica* van den Boom & Vězda — Corticolous on *Alnus*, 0–2 m. **GB:** 864, S36323. The collection is sterile but supports goniocystangia typical of *G. helvetica*.
*Gyalideopsis muscicola P. James & Vězda — Muscicolous on *Rhytidiadelphus triquetrus* and on mosses over bark on tree trunks, on high beaches and over logs, 0–9 m. **DUN:** 462, F10383, S39504 (fertile); **GB:** 874, S36717 (fertile); 556, T41647; **GUS:** 437, S39165; [State Dock, S36859].
*Gyalideopsis piceicola* (Nyl.) Vězda & Poelt — Corticolous on smooth *Alnus* bark, *Picea* twigs, fine *Tsuga* twigs and once fungicolous on polypore, 0–162 m. **EX:** [858, S36170 (fertile)]; [859, S36232]; **GB:** 857, S36155 (sub *Fellhanera bouteillei*); 874, S36709; **GUS:** 138, F10475; 855, S36054; 857, S36118, F9644; 878, S36770 (sub *Parmeliella parvula*); 531, T41524; 532, T41550; [557, T41692]; 224, M2704a.
*Halecania athallina* Fryday — See ‘Descriptions of New Genera and Species’.
*Halecania viridescens Coppins & P. James — Corticolous on *Alnus* twig below eagle perch, on *Shepherdia canadensis* stalk and on *Sambucus racemosa*, 0–2 m. **DUN:** 339, S38154, S38159; **GB:** 864, S36356; 867, S36434; **GUS:** 231, M2766. TLC: argopsin, unknowns. First reported from North America by Tønsberg (1994), from Washington.
*Helocarpon crassipes* Th. Fr. — Saxicolous in tundra with bryophytes and *Dryas*, 115 m. **EA:** 870, F9782.
*Herteliana alaskensis* (Nyl.) S. Ekman — Saxicolous in supralittoral splash zone, on gneiss, 0–5 m. **DUN:** 463, F10392, F10410 (sub *Adelolecia kolaënsis*).
#*Heterocephalacria bachmannii* (Diederich & M. S. Christ.) Millanes & Wedin (syn. *Syzygospora bachmannii* Diederich & M. S. Christ) — Lichenicolous on *Cladonia* sp., terricolous, 895 m. **EX:** 370, S38354; 371, S38367.
#*Heterocephalacria physciacearum* (Diederich) Millanes & Wedin (syn. *Syzygospora physciacearum* Diederich) — Lichenicolous on *Physcia alnophila*, corticolous on *Populus balsamifera*, upper beach, 7 m. **WA:** 395, S38671 (sub *Phoma physciicola*).
*Heterodermia galactophylla* (Tuck.) W. L. Culb. — Corticolous on *Alnus* and *Populus balsamifera*, 0–33 m. **GB:** N Sandy Cove, S36642; 867, S36431; **GUS:** 316, S37882; 330, S38062; Tower Rd, S40728; [557, T41708].
*Hydropunctaria alaskana* Thüs & Pérez-Ort. — See ‘Descriptions of New Genera and Species’.
*Hydropunctaria maura* (Wahlenb.) Keller *et al*. — Saxicolous in the supralittoral splash zone on gneiss, sea level. **DUN:** 134, F10464; 463, F10410 (sub *Adelolecia kolaënsis*), F10411; **GUS:** 873, F9818; 875, F9836 (sub *Verrucaria epimaura*); shore E of Park HQ, F9647. This ubiquitous species is certainly found on seashores in all sectors.
*Hypogymnia apinnata* Goward & McCune — Corticolous on *Picea sitchensis* branches (9×) and *Alnus* (2×), 0–162 m. **DUN:** 339, S38160; 463, S39549; **EA:** nr 438, S39294; 872, S36612; **GB:** 868, S36483, S36496; Beardsley Island, Stephens 240 (UC, det. B. McCune 1992); **GUS:** 397, S38695; 435, S39128; 855, F9604, S36007; 857, S36100, S36126; [861, F9673]; **WA:** Blue Mouse Cove Plot BM1a, BM2a (GLBA herbarium). TLC: atr, apinnatic acid.
*Hypogymnia duplicata* (Ach.) Rass. — Corticolous on *Pinus contorta* (4×) and *Tsuga heterophylla* (2×) but common and thus often disregarded, 0–569 m. **DUN:** 337, S38119; 464, S39612; **EX:** [859, F9660]; 358, S38313; **GUS:** 107, F10019; 855, S36006; 878, S36786; 210, M2544.
*Hypogymnia enteromorpha* (Ach.) Nyl. — Corticolous on *Pinus contorta* (4×), *Picea sitchensis* (1×) and *Tsuga heterophylla* (1×), but common and thus often disregarded, 0–222 m. **DUN:** nr 334, S38110; 338, S38130; 464, S39610; **EX:** [859, F9659]; [V431, S39056]; **GB:** N Sandy Cove, S36643; **GUS:** 862, S36280; 210, M2551, M2557.
*Hypogymnia hultenii* (Degel.) Krog — Corticolous on *Picea sitchensis*, *Pinus contorta*, *Tsuga heterophylla* and less commonly on *Alnus*, from beach fringe to upper montane area, 0–687 m. **DUN:** 339, S38174; 463, S39547; 573, T41806; **EA:** 872, S36613 (sub *Pertusaria glaucomela*); **EX:** 433, S39087; 448, F10315; **GB:** 864, S36327; S Sandy Cove, S40742; **GUS:** 100, F9894; 107, F10023; 434, S39110; 855, S36018; 857, S36137; 576, T41829; **WA:** 391, S38602; Blue Mouse Cove plot BM1A, BM2A (GLBA herbarium). A typical rainforest epiphyte that has advanced with glacial retreat as far north as Wolf Point in the East Arm.
*Hypogymnia inactiva* (Krog) Ohlsson — Corticolous on *Pinus contorta* in muskeg and low elevation sand flats, 32–240 m. **EX:** Yellowlegs Muskeg, S37853, S37855; **GUS:** 862, S36303.
*Hypogymnia lophyrea* (Ach.) Krog — Corticolous on *Picea sitchensis* and *Pinus contorta* from beach fringe to krummholz, 0–922 m. **DUN:** 129, F10365; 338, S38136; 463, S39530; **GB:** 868, S36526; **GUS:** 857, S36129; 230, M2748.
*Hypogymnia occidentalis* L. Pike — Corticolous on *Picea sitchensis*, 21 m. **EX:** 433, S39086. TLC: atr, physodic acid, unidentified substances.
*Hypogymnia oceanica* Goward — Corticolous on *Picea sitchensis* and *Pinus contorta* twigs, 0–33 m. **GUS:** 316, S37880; [V329, S38049]; 857, S36112.
*Hypogymnia physodes* (L.) Nyl. — Corticolous on *Alnus* (2×), *Picea sitchensis* (2×) and *Pinus contorta* (2×) but often not collected, 0–32 m. **EA:** 440, S39211, S39235; **GB:** N Sandy Cove, S36644; **GUS:** Mans 204, S38432; 397, S38704; 862, S36286; **WA:** Blue Mouse Cove plot BM1a (GLBA herbarium).
*Hypogymnia tubulosa* (Schaer.) Hav. — Corticolous on *Picea sitchensis*, 0–922 m. **EX:** 407, S38806; **GB:** 868, S36514; **GUS:** 436, S39159; **WA:** Blue Mouse Cove plot BM2a (GLBA herbarium). Two forms occur in GLBA. One has long, narrow lobes and pointed lobe tips, and accounts for most specimens; the other (represented only by S38806) has shorter lobes that end in blunt, rounded tips, similar to *H. tubulosa* in Europe.
*Hypogymnia vittata* (Ach.) Parrique — Corticolous on *Alnus* (3×), *Malus fusca* (1×), *Picea sitchensis* (2×) and *Pinus contorta* (2×), 8–250 m; also terricolous in alpine heath, 872–937 m. **DUN:** 464, S39608; **EA:** 370, S38358; 405, S38765; 407, S38804; 409, S38897; 440, S39210; 441, F10208; 456, S39472; 459, F10359 (sub *Parmelia omphalodes*); **EX:** 109, F10051; [125, F10279]; 407, F10108; **GB:** N Sandy Cove, S36639; S Sandy Cove, S40746; **GUS:** 857, S36077, S36127; 434, S39108. TLC: atr, physodic, 2′-*O*-methylphysodic, vittatolic acids.
*Hypotrachyna sinuosa* (Sm.) Hale — Corticolous on *Alnus* (8×), *Picea sitchensis* (5×) and *Tsuga heterophylla* (1×), strongly associated with beach fringes, 0–162 m. **EA:** nr 438, S39300; 440, S39191; 441, F10191; 872, S36600; **GB:** 864, S36332; 868, S36513; 874, S36711; Marble Mtn beach, S38002; S Sandy Cove, S40745; **GUS:** 855, F9603; 857, F9639, S36107; [V329, S38032]; 434, S39098; 436, S39148; 855, S36016; **WA:** Blue Mouse Cove plot BM1a (GLBA herbarium).
*Icmadophila ericetorum* (L.) Zahlbr. — Lignicolous on mossy log, 162 m. **GUS:** 855, S36042. A common species, probably overlooked in other sectors.
*Imshaugia aleurites* (Ach.) S. L. F. Meyer — Corticolous on *Pinus contorta*, 233–250 m. **EX:** 109, F10054; 314, S37850.
*Ionaspis lacustris* (With.) Lutzoni — Saxicolous on semi-inundated, acidic to slightly basic rock along creeks, 0–225 m. **DUN:** 463, S39600; **EX:** 128, F10285.
*Ionaspis odora* (Ach.) Stein. — Saxicolous on granitic rock and alkaline argillite, 10–918 m. **EA:** Muir Inlet, S40766, S40777, S40787; **EX:** 454, F10332 (sub *Porpidia* sp.); **GB:** 865, S36415; **WA:** 101, F9914, F9920, F9921; 319, S37932 (sub unidentified pyrenocarp); 381, S38446, S38447 (sub *Rhizocarpon geminatum*).
*Ionaspis ventosa* P. M. Jørg. & R. Sant. — Saxicolous in *Dryas* mats in glacial forelands and on soft argillite on alpine ridge, 13–918 m. **EA:** 870, F9792; **EX:** 409, S38901; 454, S39420; **WA:** 321, S37959. Apothecia innate, 0.2–0.25 mm diam.; ascospores 10–12 × 5–6 μm; epihymenium KOH−; exciple and hypothecium I+ blue; hymenium I+ yellow. DNA sequences of six loci from S39420 were used by Resl *et al.* (2015: isolate P126) for a wider study of the subclass Ostropomycetidae.
*Jamesiella anastomosans* (P. James & Vězda) Lücking *et al*. — Muscicolous on *Lobaria* on *Picea* branch, 12 m. **GUS:** 397, S38709; 531, T41525. First reported for Alaska from Lake Clark by McCune *et al.* (2018).
*Japewia subaurifera* Muhr & Tønsberg — Corticolous on *Picea* from 0–922 m, on *Pinus contorta*, also once on giant *Tsuga* snag, 569 m. **DUN:** 570, T41772; **EX:** 358, S38306; 407, S38820; 448, S39354; **GB:** 868, S36524; 874, S36704 (sub *Biatora rufidula*); 868, S36524; **GUS:** 210, M2558a. TLC: secalonic A.
*Japewia tornoënsis* (Nyl.) Tønsberg — Corticolous on *Picea* branches in krummholz (883–922 m), once on *Alnus* bark and seashore driftwood (0–5 m). **EX:** 405, S38763; 407, S38811 (sub *Lecidea roseotincta*), S38818; 217, M2596; **GB:** S Sandy Cove, S40744; **GUS:** 435, S39133.
*Kaernefeltia californica* (Tuck.) A. Thell & Goward — Corticolous on *Pinus contorta*, 0–32 m. **GUS:** 107, F10021, F10028; [861, F9668, F9669]; 862, F9681, F9682, S36299; Moose Flats, *J. Dickson* s. n. (UBC, n.v.); 540, T41584; 210, M2558b.
*Kaernefeltia merrillii* (Du Rietz) A. Thell & Goward — Corticolous on *Pinus contorta*, 16 m. **GUS:** 862, F9682 (sub *Kaernefeltia californica*); 317, S37894.
*Lambiella aliphatica* T. Sprib. & Resl — See ‘Descriptions of New Genera and Species’.
*Lambiella caeca* (J. Lowe) Resl & T. Sprib. — Corticolous on *Pinus contorta* branches and on old cones, 16–32 m. **DUN:** 464, F10420 (sub *Platismatia glauca*); **GUS:** 107, F10034; [861, F9669 (sub *Kaernefeltia californica*)]; 862, S36295; 317, S37892; 210, M2556. This is the same species as *Rimularia* sp. S29406 of the KLGO study (Spribille *et al.* 2010). *Lambiella caeca* was published from several western North American sites by Resl *et al.* (2015) using a multilocus DNA sequence set, including one voucher from GLBA (isolate T1072, S36295). It was previously thought to occur mainly in eastern North America (Rambold & Printzen 1992).
**#*Lasiosphaeriopsis lecanorae* Pérez-Ort. & Halıcı — On *Lecanora polytropa* s. lat. on rock, 10 m. **WA:** 384, S38517. Described from Spain (Pérez-Ortega & Halıcı 2008) and otherwise reported from central Europe (Schiefelbein *et al.* 2017) and, tentatively, from Svalbard (Zhurbenko & Brackel 2013).
*Lathagrium fuscovirens* (With.) Otálora *et al*. — Saxicolous on limestone rocks on shore, 2–5 m. **GB:** 863, F9685; 864, F9723, S36387; 867, S36444; N tip Willoughby Island, F9708.
*Lecanactis megaspora* (G. Merr.) Brodo — Corticolous on veteran, large *Picea sitchensis* (2×) and *Tsuga heterophylla* (3×), or *T. mertensiana* (1×) on dry trunk areas sheltered from direct rain, 28–687 m. **DUN:** 332, S38066; 563, T41756; 565, T41762; **EX:** [858, F9655]; 354, S38292; 443, S39318; 448, F10318, S39361; [860, S36250]; **GUS:** 857, S36096 (as ‘cf.’); 881, S36834 (as ‘cf.’).
*Lecania cuprea (A. Massal.) van den Boom & Coppins — Saxicolous on limestone rocks on shore, 0–5 m. **GB:** 863, F9705.
*Lecania hydrophobica* T. Sprib. & Fryday — See ‘Descriptions of New Genera and Species’.
*Lecania subfuscula* (Nyl.) S. Ekman — Terricolous, 0–8 m. **EA:** 232, M2773.
*Lecanora alaskensis* H. Magn. — See ‘Other Species Treated in Detail’.
*Lecanora boligera* (Norman ex Th. Fr.) Hedl. — Corticolous on *Picea sitchensis* branch in krummholz, 883 m. **EX:** 405, S38762.
*Lecanora caesiosora* Poelt — Saxicolous on vertical sheltered rock with overhanging herbaceous vegetation, high subalpine zone, 809 m. **EX:** 452, S39365, S39367. TLC: atr.
*Lecanora cinereofusca* H. Magn. — Corticolous on *Alnus* bark, 5 m. **DUN:** 573, T41799. TLC: pannarin, placodialic acid.
*Lecanora confusa* Almb. — Corticolous on *Alnus* twigs overhanging cliff on near-outer coastal headland, 10 m. **DUN:** 467, S39646. A Californian/Pacific Northwest species at the northern limits of its range.
*Lecanora ecorticata J. R. Laundon — Saxicolous at base of overhanging rock wall, 8 m. **DUN:** 572, T41780. TLC: usnic and isousnic acids, atr. The specimen seems to fit Laundon's (2003) description, except for the presence of isousnic acid. Laundon reported it for British Columbia but the species has been overlooked in recent editions of the North American lichen checklist.
*Lecanora expallens* Ach. — Corticolous on *Picea sitchensis*, 21 m. **EX:** 312, S37811; 433, S39085.
*Lecanora farinaria Borrer — Corticolous on *Alnus* and *Picea sitchensis*, also lignicolous on suspended log on high beach, 4–9 m. **DUN:** 462, S39503; **GB:** 874, S36707; **GUS:** 341, S38225; 531, T41527; 559, T41734; [574, T41818, T41819, T41820]; 576, T41851; 230, M2750. TLC: atr, fatty acid (probably roccellic).
*Lecanora frustulosa* (Dicks.) Ach. — Saxicolous on alpine argillitic rocks, 871–936 m. **EX:** 375, S38421; 407, S38833 (sub *Collema glebulentum*); 455, S39430 (sub *Euopsis pulvinata*); 458, S39483. TLC: usnic acid only or nil, but we suspect too little material was used for assay.
*Lecanora intricata* (Ach.) Ach. — Saxicolous on soft argillite, 922 m. **EX:** 372, 38369 (as ‘aff.’); 407, S38837; 454, S39397; **WA:** 388, S38556 (as ‘aff.’). TLC: usnic acid, zeorin, several fatty acids (major).
*Lecanora leptacina* Sommerf. — See ‘Other Species Treated in Detail’.
*Lecanora orae-frigidae* R. Sant. — Lignicolous on beached driftwood, with *Xylographa opegraphella*, 0–5 m. **WA:** 106, F10018; 396, S38689 (sub *X. opegraphella*).
*Lecanora paddensis* (Tuck.) T. Sprib. — Corticolous on *Salix*, 2 m. **WA:** 391, S38592.
*Lecanora phaeostigma* (Körb.) Almb. — Corticolous on large *Tsuga* trunk, 687 m. **EX:** 448, S39344.
*Lecanora poliophaea* (Wahlenb.) Ach. — Saxicolous in intertidal zone on sedimentary boulders, sea level. **GB:** 874, F9834 (as ‘cf.’); **GUS:** 875, F9840. The inclusion of a recently published sequence of this species (Kistenich *et al.* 2018) into a broad sample of *Lecanorales* (Fig. 10) confirms that the fungal component of this lichen belongs to the same clade as members of the genus *Myriolecis* Clem. (Fig. 10), a genus described in 1909. However, Kondratyuk *et al.* (2019) pointed out an older name for this group, *Polyozosia* A. Massal., published in 1855 and based on *Lichen peltatus* * *poliophaea* Wahlenb. However, Kondratyuk *et al.* transferred into *Polyozosia* only a small number of the many species that would need to be accommodated there. We refrain from adopting the new nomenclature and making further combinations, or combining *L. poliophaea* into *Myriolecis*, as we have not studied this problem ourselves and await further research.
*Lecanora polytropa* (Ehrh. ex Hoffm.) Rabenh. s. lat. — Saxicolous on small rocks from pebbles to boulders, often early successional rock surfaces, 0–5 m. **EA:** 872, S36630; **EX:** 374, S38405; **WA:** 105, F10003 (small), F10004 (‘cottony’), F10005, S38647 (‘cottony’); 381, S38465; 384, S38517; 389, S38575; 391, S38637; 392, S38647. Several species are probably hidden in this group but a taxonomic revision is beyond the scope of this study. TLC: usnic acid, zeorin, unidentified fatty acid(s).
*Lecanora pulicaris* (Pers.) Ach. — Corticolous on *Alnus*, 8–27 m. **DUN:** 339, S38148 (s. lat.); **EA:** 440, S39208; **GB:** S Sandy Cove, S40743. TLC: atr, roccellic acid. This matches the fumarprotocetraric acid-deficient chemotype described by Brodo (1984*b*) from the Pacific coast.
*Lecanora stanislai Guzow-Krzemińska *et al*. — Corticolous on *Salix*, 32 m. **GUS:** 862, S36318. TLC: usnic acid, zeorin. A recently described, sterile, sorediate species from the *Lecanora symmicta* group, widespread in Eurasia and North America (Guzow-Krzemińska *et al.* 2017).
*Lecanora swartzii* (Ach.) Ach. — Saxicolous in the alpine zone, 937 m. **EX:** 409, S38880. TLC: atr, unidentified fatty acid, pigment.
*Lecanora symmicta* (Ach.) Ach. — Corticolous on *Alnus* (4×), *Picea* (2×), *Populus balsamifera* (1×) and *Salix* (4×), 0–11 m. **DUN:** 113, F10132; 463, S39510; **EA:** 123, F10241; 872, S36609; nr 438, S39317 (sub *Lecidea* sp.); **GUS:** 435, S39115; 230, M2764; **WA:** 391, S38604; 395, S38663. TLC (S39115): usnic acid, zeorin.
*Lecanora viridipruinosa* M. Svenss. & T. Sprib. — See ‘Descriptions of New Genera and Species’.
*Lecidea albofuscescens* Nyl. — Corticolous on *Picea sitchensis* twigs on coastal headlands, also once on *Pinus contorta* twigs, 0–37 m. **DUN:** 429, S39029 (sub *Micarea peliocarpa*); 463, S39544; **GB:** 868, S36527; **GUS:** 435, S39124 (sub *Scoliciosporum chlorococcum*); 230, M2751, M2760. See discussion under the new species *Lecidea streveleri* and Fig. 10 (MS008 is a sequence from M2751).
*Lecidea berengeriana* (A. Massal.) Th. Fr. — Terricolous/muscicolous in alpine tundra, 900 m. **EX:** 216, M2582; 218, M2632b.
*Lecidea ecrustacea* (Anzi ex Arnold) Arnold — Saxicolous in the alpine zone, 895 m. **EX:** 370, S38347 (sub *Rhizocarpon eupetraeoides*).
*Lecidea erythrophaea* Flörke ex Sommerf. — Corticolous on *Alnus*, *Cupressus nootkatensis* and *Salix*, 0–46 m. **DUN:** between 412 and 413, S38918; **GUS:** 879, S36806; 316, S37865.
*Lecidea globulispora Nyl. — Lignicolous on a thin twig of *Pinus contorta*, 17 m. **GUS:** 210, M2558a. Thallus thin to invisible; ascospores globose, 3–4 μm diam.; epihymenium brown, KOH−; paraphyses capitate, terminal cell to 4 μm, with brown pigment. Part of a complex of round-spored species probably related to the *Lecanora fuscescens* group, which are in need of taxonomic revision.
*Lecidea griseomarginata* Fryday — See ‘Descriptions of New Genera and Species’.
*Lecidea haerjedalica* H. Magn. — Saxicolous, 43–937 m. **EX:** 409, S38888; **WA:** 319, S37940 (sub *Micarea* sp. F10320). Only previous Alaskan record is from Barrow (Fryday 2004).
*Lecidea lactea* Flörke ex Schaer. — Saxicolous, 10–919 m. **EX:** 374, S38410; **WA:** 101, F9908, F9909, F9918; 218, M2649. We recognize two distinct taxa in the *L. lapicida* group with an epilithic thallus containing norstictic acid and a ± hyaline hypothecium: one has a thin thallus with sessile apothecia (*L. lapicida* var. *pantherina*), whereas the other has a thick white thallus and innate apothecia (*L. lactea*). See *Lecidea griseomarginata* (Descriptions of New Genera and Species) for a fuller discussion of these taxa.
*Lecidea lapicida* (Ach.) Ach. — Saxicolous, granite to alkaline argillite, 0–922 m. **EX:** 370, S38342, S38345; 374, S38412 (sub *Lecanora* sp.); 407, S38828, S38837 (as ‘aff.’; sub *Lecanora intricata*); 453, F10321 (sub *Opegrapha geographicola*), F10326; **WA:** 102, F9945; 105, F9987. TLC: stictic acid.
*Lecidea malmeana* Zahlbr. — Corticolous on *Pinus contorta*, 22 m. **GUS:** 210, M2563. DNA from this specimen is used for the analysis presented in Fig. 10.
*Lecidea paupercula* Th. Fr. — Saxicolous on granite and alkaline argillite, 15–918 m. **DUN:** 423, S38976 (sub *Rhizocarpon geographicum* aggr.); **EX:** 454, F10328; **WA:** 101, F9909 (sub *Lecidea lactea*); 381, S38450 (sub *Rhizocarpon macrosporum*).
*Lecidea phaeops Nyl. — Saxicolous on low alpine outcrop, 871 m. **EX:** 458, S39484. First reported for North America from Haida Gwaii (British Columbia), from a similar habitat (Brodo *et al.* 1987).
*Lecidea praenubila* Nyl. — Saxicolous, 883–937 m. **EX:** 370, S38349; 405, F10097 (sub *Melanelia hepatizon*); 409, S38879.
*Lecidea roseotincta* Coppins & Tønsberg — Corticolous on *Alnus*, *Oplopanax* and *Salix*, 2–86 m, also on seaside and subalpine krummholz *Picea sitchensis* at the treeline, 922 m. **DUN:** 333, S38076; 339, S38150; 462, S39712; 463, S39512, S39515; 467, S39643; 573, T41800; **EA:** 122, F10228; **EX:** 407, S38803, S38811; **GB:** 864, S36339 (sub *Micarea peliocarpa*); Marble Mtn beach, S38007; 556, T41658; **WA:** 327, S38007; 391, S38591.
*Lecidea silacea* Ach. — Saxicolous on small metal-rich rocks, 11 m. **EA:** 872, S36631.
*Lecidea sphaerella* Hedl. — Corticolous on *Oplopanax horridus* and *Populus balsamifera*, 3–32 m. **GB:** 874, S36693 (det. C. Printzen), S36696; **GUS:** 862, S36315, S36316; 237, M2842.
*Lecidea streveleri* T. Sprib. — See ‘Descriptions of New Genera and Species’.
*Lecidea swartzioidea* Nyl. — Saxicolous on alpine ridgetop, on alkaline argillite, 903–918 m. **EX:** 374, S38412 (sub *Lecanora* sp.); 406, S38782; 454, F10329. TLC: norstictic acid; exciple C−, dark hypothecium and medulla I + .
*Lecidella carpathica* Körb. — Saxicolous on granitic and argillitic rocks on shores and outcrops as well as on underhangs, 0–922 m. **DUN:** 339, S38203 (as ‘aff.’), S38216 (as ‘aff.’); **EX:** 404, F10068; 405, F10097 (sub *Melanelia hepatizon*), F10099; 407, F10131 (sub *Protoparmeliopsis muralis*); 454, F10333 (sub *Fuscidea intercincta*), S39391 (as ‘aff.’, sub *Sagiolechia phaeospora*); **GB:** 868, F9753 (sub *Baeomyces rufus*); **WA:** 102, F9939; 383, S38504 (as ‘aff.’); 384, S38522 (as ‘aff.’).
*Lecidella elaeochroma* (Ach.) M. Choisy, s. lat. — Corticolous on *Shepherdia canadensis* 0–43 m. **GB:** 867, S36435; **WA:** 319, S37922 (sub *Biatora subduplex*); 544, T41606, T41607. TLC: a xanthone.
*Lecidella patavina* (A. Massal.) Knoph & Leuckert — Saxicolous on alpine rocks, 890–937 m. **EX:** 370, S38342 (sub *Lecidea lapicida*), S38349 (sub *Lecidea praenubila*); 373, S38387; 407, F10130; 409, S38883; 453, F10324; 454, S39391 (sub *Sagiolechia phaeospora*, with *Lecidella carpathica*), S39409.
*Lecidella stigmatea* (Ach.) Hertel & Leuckert — Saxicolous on gneiss and alkaline argillite, from the supralittoral to the alpine zone, 0–922 m. **DUN:** 462, F10373 (sub *Rhizocarpon* cf. *grande*), F10374, F10378; **EX:** 407, F10126 (sub *Lecanora* sp. F10126); 459, F10360 (sub *Candelariella* sp.).
*Lecidella wulfenii* (Hepp) Körb. — Muscicolous or (loosely?) saxicolous on alkaline argillite in alpine heath, 883–922 m. **EX:** 405, F10089; 407, F10113; 453, F10327.
*Lecidoma demissum* (Rutstr.) Gotth. Schneid. & Hertel — Terricolous on organic accumulations, 618 m. **DUN:** 428, S39025.
*Lempholemma intricatum (Arnold) Zahlbr. — Saxicolous on limestone rock face, 11 m. **EA:** 872, S36623 (det. M. Schultz, April 2012), S36626; **EX:** 454, S39415; **GB:** 867, S36446, S36449. A specimen of this species was erroneously reported as *Leciophysma finmarkicum* from KLGO (Spribille *et al.* 2010).
*Lempholemma isidioides (Nyl. ex Arnold) H. Magn. — Saxicolous on limestone rock face near shore, 0–5 m. **GB:** 867, F9749 (det. M. Schultz).
**Lempholemma minutulum (Bornet) Zahlbr. — Saxicolous on pebbles on beach ridges; 0–5 m. **WA:** 105, F9979 (det. M. Schultz). Ascospores 15 × 10 μm, simple; conidia *c.* 5–6 μm; photobiont *Nostoc*, cells *c.* 7 μm. This species has been reported only for France and Switzerland. It was recently treated by Nimis *et al.* (2018) with a brief description *ad interim* in the genus *Collemopsidium*, but without a formal combination because the validity of the original description is in question.
*Lempholemma polyanthes* (Bernh.) Malme — Saxicolous on hard limestone rocks on shore, 0–5 m. **GB:** 864, F9724 (sub *Placynthium nigrum*); 867, S36448 (det. M. Schultz, April 2012).
*Lempholemma radiatum* (Sommerf.) Henssen — Terricolous or on organic accumulations on bare glacial till, on mossy bank beside creek, and over granitic rocks, 0–10 m. **EA:** vicinity of Nunatak Knob, east slope of Muir Inlet, (S of McBride Glacier), 8 August 1968, *I. A. Worley* 10422 (UBC-L4893, det. A. Henssen); 440, F10190; **WA:** 102, F9946; 104, F9976. *Lempholemma radiatum* was mentioned as a dominant lichen species of the ‘black crust phenomenon’ on calcareous post-glacial soils by Worley (1973). However, it was not dominant in any of the areas we surveyed.
*Lepra* aff. *borealis* (Erichsen) I. Schmitt *et al*. — Corticolous on *Alnus*, *Malus fusca*, *Menziesia ferruginea*, *Pinus contorta, Salix* and *Vaccinium ovalifolium*, 0–250 m. **DUN:** 462, S39710; 561, S41740; **EA:** 123, F10248; **EX:** [858, S36175]; 110, F10058; [125, F10276]; **GB:** 864, S36328; 556, T41679; **GUS:** 855, S36029, S36037, S36039; [V431, S38228]; 531, T41521; 556, T41678; 576, T41841; Bartlett Cove, S40724; **WA:** 391, S38615. TLC: fpc, pc. A sterile sorediate crust similar to *Lepra borealis* but with a bluish tinge, ±flat to concave soralia, and containing fumarprotocetraric and protocetraric acids but (unlike *L. borealis*) lacking succinprotocetraric acid. The *L. borealis*/*pupillaris* complex contains more species than currently described and is in need of further studies. These should be based on material from Europe, North America and Asia (where it has been reported by Ren (2015) and Himelbrant *et al.* (2019)).
*Lepra dactylina* (Ach.) Hafellner — Terricolous, on sod or on organic accumulations over alkaline argillite, alpine zone, 883–922 m. **EX:** 370, S38357; 372, S38376; 405, F10087; 407, F10123; 454, F10348.
*Lepra ophthalmiza* (Nyl.) Hafellner — Corticolous on *Alnus*, 2 m. **WA:** 327, S38001. TLC: isomyelochroic acid. See also ‘Other Species Treated in Detail’.
*Lepra subvelata* (G. Merr.) T. Sprib. — Corticolous on *Alnus*, *Pinus contorta*, *Picea sitchensis* and *Tsuga heterophylla*, 0–50 m. **DUN:** 333, S38090; 339, S38151; 462, S39724; 463, S39517; 464, S39602, F10417; 467, S39645; **EA:** 441, S39272; **EX:** [858, S36200]; **GUS:** 855, S36003, S36025; 857, S36088; 876, S36747; 879, S36801; 316, S37871; Tower Rd, S38278; 210, M2542. TLC: nephrosterinic, isonephrosterinic acids. One of the most common *Lepra* species on oligotrophic bark in NW North America. This is the same as *Pertusaria* sp. TT32951 of Spribille *et al.* (2010). See ‘Other Species Treated in Detail’.
*Lepraria diffusa* (J. R. Laundon) Kukwa — Corticolous on *Alnus*, *Cupressus nootkatensis*, *Populus balsamifera* and *Tsuga heterophylla*, 10–59 m. **DUN:** 114, F10139; 467, S39666; **EX:** [860, S36242]; [V431, S39043]; **GB:** 874, S36692; **GUS:** 856, S36064. TLC: cf. 4-oxypannaric acid-6-methyl ester.
*Lepraria finkii* (B. de Lesd.) R. C. Harris — Corticolous on *Picea sitchensis*, 9 m. **DUN:** 463, 39538. TLC: atr, zeorin, stictic + sats, nephrosteranic acid. Poor material.
*Lepraria eburnea* J. R. Laundon — Over shoreline and supralittoral rocks, granite and hornblende gneiss, underhangs, also once on *Alnus* bark, 0–37 m. **DUN:** 462, F10375 (sub *Rhizocarpon reductum*); [**EX:** V431, S39038]; **WA:** 102, F9944; 383, S38490. TLC: alectorialic acid, ±cf. psoromic acid.
*Lepraria jackii* Tønsberg — Muscicolous at base of conifer stump, 18 m. **GUS:** 316, S37890. TLC: atr, angardianic/roccellic acid, unidentified fatty acids (tr.).
*Lepraria neglecta* (Nyl.) Lettau s. lat. (*sensu* Lendemer 2013 (as *L. neglecta* (Nyl.) Erichsen)) — Terricolous in snowbeds and over rock, seashore outcrops to alpine zone, 5–903 m. The species is morphologically and chemically heterogeneous (see Lendemer 2013). **DUN:** 414, S38934 (TLC: atr, angardianic/roccellic acid, stictic acid + sat); 423, S38980 (atr, jackinic/rangiformic acid+ sat, fpc); 572, T41788 (angardianic/roccellic acid, fpc); 463, F10388 (alectorialic acid + sat, porphyrilic acid); **EX:** 406, S38796; 455, S39445 (alectorialic, angardianic/roccellic acids); **WA:** 383, S38496 (atr, porphyrilic, angardianic/roccellic acids); 546, T41624 (atr, jackinic/rangiformic acid, fpc).
*Lepraria nivalis* J. R. Laundon — Corticolous on *Cupressus nootkatensis*, 0–42 m. **DUN:** 412, S38912; 469, S39695. TLC: atr, pc.
*Lepraria pacifica Lendemer — Corticolous on *Picea* branch, sheltered underhangs, 12 m. **GUS:** 397, S38693. TLC: divaricatic acid, zeorin.
*Lepraria rigidula* (B. de Lesd.) Tønsberg — Corticolous on *Alnus* and *Picea sitchensis*, 0–8 m. **DUN:** 463, S39538; **EA:** 440, S39263; **GB:** 864, S36330; **WA:** 542, T41593. TLC: atr, nephrosteranic acid.
*Lepraria torii* Pérez-Ort. & T. Sprib. — Corticolous, often on *Tsuga heterophylla* snags, also on *Picea sitchensis*, once directly lignicolous and on detritus, 0–569 m. **DUN:** 564, T41760b, T41760c; between 462 and 469, S39683; **EX:** [858, S36190]; [859, S36227, S36230]; 310, S37800; 358, S38307, S38319; 433, S39089; **GB:** 874, S36686; **GUS:** 855, S36005, S36032; 876, S36739; 100, F9886; [558, T41719]; 559, T41723. TLC: fpc, fatty acid (angardianic/roccellic acid).
*Lepraria vouauxii* (Hue) R. C. Harris — Saxicolous on calcareous rock faces and underhangs, often where seepy, 20–903 m. **EX:** nr 406, S38801; **GB:** 867, S36439; Puffin Island, S36651; **WA:** 392, S38653; 103, F9953. TLC: dibenzofurans (including pannaric acid-6-methylester), plus another major dibenzofuran (= 4-oxypannaric acid-6-methylester?), vouauxii unknown.
*Leptogidium dendriscum* (Nyl.) Nyl. — Corticolous on *Alnus* (mainly trunks; 3×), *Malus fusca* (1×), *Picea sitchensis* twigs (4×) and *Tsuga heterophylla* twigs (4×) in low elevation rainforest, 0–60 m. **EA:** 442, S39287; **EX:** [859, F9661, F9663]; 432, S39066; [858, S36193, S36194 (sub *Szczawinskia tsugae*)]; **GB:** 868, S36468; S Sandy Cove, S40740; **GUS:** 855, S36004 (sub *Gyalideopsis epicorticis*); 857, S36103 (sub *Dendriscosticta wrightii* dendriscocauloid morph), S36125; 862, F9680; 878, S36784; 879, S36807; 316, S37869; Tower Rd, S37506, S40733; [V329, S38033 (sub *Szczawinskia tsugae*)]; [557, T41697]; **WA:** Blue Mouse Cove plot BM1a (GLBA herbarium).
*Leptogium saturninum* (Dicks.) Nyl. s. lat. — Corticolous on *Alnus* (5×), *Populus balsamifera* (1×) and *Salix* (2×), typically at the back of beach meadows, 0–15 m, also saxicolous once at 937 m. **EA:** 872, S36601, S36615; nr 438, S39308 (fertile); 440, S39229; 441, F10205; **EX:** 409, S38895; **GUS:** 857, F9637, S36139, S36142 (fertile); **WA:** 391, S38612. We sequenced several loci from a rare, richly fertile specimen (isolate T1731 from S39308) but it does not appear close to any of the species sequenced by Stone *et al.* (2016). The ITS sequence we obtained differs in at least 38 positions from the next nearest published sequence from this group (GenBank Accession MK778616, from Siberia).
*#*Lichenochora lepidiotae* (Anzi) Etayo & Nav.-Ros. — Lichenicolous on *Fuscopannaria* sp., corticolous on *Alnus*, 8 m. **EA:** 440, S39232. This species was reported by Zhurbenko (2013) as new to North America from the Canadian Arctic.
*Lichenomphalia umbellifera* (L.) Redhead *et al.* — Lignicolous on log, 234 m. **EX:** 349, S38285; **GUS:** 435, S39132.
#*Lichenopuccinia poeltii* D. Hawksw. & Hafellner — Lichenicolous on *Parmelia* spp., usually where corticolous on fine *Picea sitchensis* or *Tsuga heterophylla* twigs close to the seashore, 9–27 m. **DUN:** 339, S38152, S38179, S38197; 463, S39543; **GUS:** A569, on *Parmelia* sp., P2322.
#*Lichenostigma alpinum* (R. Sant. *et al.*) Ertz & Diederich (syn. *Phaeosporobolus alpinus*) — Lichenicolous on *Ochrolechia frigida, Ochrolechia sp.* and *Pertusaria subambigens*. **EX:** 565, P2338; 566, P2391, P2393; **GUS:** A569, P2289, P2372, P2375, P2408.
*Lichinodium sirosiphoideum* Nyl. — Over *Lopadium* on dry, mossy branch near base of trunk of *Pinus contorta* in glacial outwash plain, 17 m. **GUS:** 538, T41580. We also refer another collection here, **GUS:** 878, S36767 (*Tsuga heterophylla* twig, 58 m), which is poorly developed and could be a young *L. sirosphoideum* or *L. canadense*. The taxonomy of *Lichinodium* is still poorly understood.
*Lithographa tesserata* (DC.) Nyl. — Saxicolous on granitic boulders, collected at 100 m and seen at *c.* 300 m in the same basin. **DUN:** 418, F10150, S38950. A rare species, first reported from North America from the Gaspé Peninsula in Québec (Sirois *et al.* 1988) and subsequently from a few sites on the North American Pacific coast (e.g. Haida Gwaii; Brodo 1995). DNA sequences from this specimen formed the basis of the first definitive phylogenetic placement of *Lithographa* (Spribille *et al.* 2014*b*; Resl *et al.* 2015, isolate P95).
#*Llimoniella pertusariae* Diederich & Etayo — Lichenicolous on *Lepra* sp., corticolous on *Alnus*, 0–10 m. **GB:** 868, S36462.
*Lobaria anomala* (Brodo & Ahti) T. Sprib. & McCune (syn. *Pseudocyphellaria anomala* Brodo & Ahti) — Corticolous on *Alnus*, *Picea sitchensis* and *Populus balsamifera*, 0–33 m. **DUN:** 463, S39529; **EA:** 441, S39276; **GB:** 864, S36373; 868, S36532; 874, S36710; **GUS:** [V329, S38043]; 436, S39144, S39149 (sub *Nephroma bellum*); 576, T41828; **WA:** Blue Mouse Cove plot BM1a, BM2a (GLBA herbarium).
*Lobaria hallii* (Tuck.) Zahlbr. — Corticolous on *Alnus*, *Picea sitchensis*, *Populus balsamifera* and *Salix*, in beach fringe forest, 0–24 m. **EA:** 441, S39275 (fertile); 442, S39290; 872, S36606; 233, M2786; **GB:** 864, S36333; S Sandy Cove, S40749; **GUS:** Tower Road, F10633; **WA:** 395, S38668, S38685.
*Lobaria kurokawae* Yoshim. — Terricolous in alpine heath, 872–903 m. **EX:** 405, F10096; 406, S38789; 456, S39473; 217, M2612. TLC: nil.
*Lobaria linita* (Ach.) Rabenh. — Terricolous over talus, in snowbeds and on shoreline rocks, 0–907 m. **EX:** 373, S38402; 404, F10071; 453, S39369; nr 455, S39457; **GB:** N tip of Willoughby Island, F9714; slopes and ridges of mainland immediately NW of Sebree Cove and W of Caroline Point, 1968, *Worley* 10934 (UBC, n.v.); Mt Wright, 1974, *Noble & Sandgren* 313, 334 (MIN, n.v.); **GUS:** semi-open sand rise along road from Bartlett Cove to Gustavus, 1968, *Worley* 10980 (UBC, n.v.); [State Dock, S36847]. Like the arctic-alpine species *Solorina crocea* and *Thamnolia vermicularis*, this species was also collected at low elevations on sand by Ian Worley in 1968. Unlike those species, *L. linita* was found again in 2012 on sand near the State Dock (ferry terminal) in Gustavus. The low and high elevation occurrences of *L. linita* were discussed by Brodo (1984*a*) but note that the slender-lobed, usually fertile, epiphytic form is treated here as *L. tenuior* (see below).
*Lobaria oregana* (Tuck.) Müll. Arg. — Corticolous on branches of *Picea sitchensis* and *Tsuga heterophylla*, less often on *Populus balsamifera*, common in low elevation rainforest and mostly not collected, 0–50 m. **DUN:** 463, S39522; **GUS:** 855, F9605; 856, S36063; 210, M2552.
*Lobaria pulmonaria* (L.) Hoffm. — Corticolous on *Alnus*, *Picea sitchensis*, *Populus balsamifera*, *Ribes lacustre* and *Tsuga heterophylla*, mostly close to the seashore, 0–44 m. **EA:** near 438, S39309; 440, S39224; [**EX:** 859, S36226]; **GB:** 864, S36372; 866, S36418; 868, S36533; **GUS:** 341, S38252; 397, S38692; 857, S36119; **WA:** Blue Mouse Cove plot BM1a, BM2a (GLBA herbarium).
*Lobaria retigera* (Bory) Trevis. — Corticolous, apparently on conifer, 0–5 m. **GUS:** Bartlett Lake trail, near NPS office, *H. T. Root* 1828 (GLBA herbarium!); near main lodge, 1976, *Nash* 13384 (ASU, photo!). Not seen during the present survey.
[*Lobaria silvae-veteris* (Goward & Goffinet) Goward & Goffinet — Corticolous on *Picea sitchensis* branches, 33 m. **GUS:** Tower Rd, S37541; 557, T41707. This species was found locally only in one small slough in the Tower Road area just outside the park boundary. It is considered by some to be a photomorph of *Lobaria oregana* (Goffinet & Goward 1998).]
*Lobaria tenuior* (Hue) M. Sâto — Corticolous on *Cupressus nootkatensis*, *Picea sitchensis* and *Tsuga mertensiana*, 0–740 m. Common and usually not collected. **DUN:** 467, S39669; **EX:** 410, S38908; **GB:** 865, S36408; 874, F9830, S36681.
*Lobarina scrobiculata* (Scop.) Nyl. — Corticolous on *Alnus* and *Picea sitchensis*, 0–32 m. **GB:** 868, S36477, S36490; S Sandy Cove, S40741; **GUS:** 862, F9683; 436, S39151; **WA:** Blue Mouse Cove plot BM2a (GLBA herbarium).
*Lopadium disciforme* (Flot.) Kullh. — Corticolous on *Alnus* (3×), *Picea sitchensis* (7×), *Pinus contorta* (1×) and *Tsuga heterophylla* (4×), 3–250 m. **DUN:** 332, S38067; 467, F10430; **EX:** 109, F10055 (sterile); [125, F10256]; [V431, S39047]; [858, S36184]; [225, M2726]; **GB:** 868, S36500; 874, F9827, S36687, S36701, S36702 (sub *Mycoblastus affinis*); **GUS:** 434, S39104; 855, F9614, S36057; 874, S36701; 876, S36730; 882, S36842; 531, T41531; 538, T41579; [557, T41713]; 237, M2835. Mitochondrial DNA sequences from *L. disciforme* (S36687, isolate T1327) differ greatly from those in *L. pezizoideum* (S38861, isolate T1326, both published here).
*Lopadium pezizoideum* (Ach.) Körb. — On organic accumulations or muscicolous over vertical sheltered rock and on low outcrops, alpine zone, 435–922 m. **DUN:** 120, F10169; 462, S39681; **EX:** 406, S38788; 407, F10119, S38861; 454, F10337, S39416; 458, S39485; 217, M2614; 218, M2618a.
*Loxospora elatina* (Ach.) A. Massal. — Corticolous on *Alnus*, *Pinus contorta*, on *Tsuga heterophylla* bark and *T. heterophylla* snag (bark), once on *Malus fusca*, 0–222 m. **DUN:** 464, S39607; **EX:** [860, S36248]; 313, S37847; 433, S39078; **GB:** 868, S36465; **GUS:** 535, T41573; 536, T41575. TLC: thamnolic, elatinic acids.
*Loxosporopsis corallifera* Brodo *et al*. — Corticolous on *Alnus*, *Menziesia*, *Picea sitchensis*, *Tsuga heterophylla* and *Vaccinium ovalifolium*, very common, 0–687 m. **DUN:** 334, S38100; 467, S39659 (fertile); **EX:** [858, F9653 (fertile), S36177 (fertile), S36187, S36196 (sub *Szczawinskia tsugae*)]; 313, S37834; 358, S38318; 400, S38731; 444, S39319; 448, F10314, S39346; 236, M2827b; **GB:** N Sandy Cove, S36645; 865, S36398; 868, S36523; 874, S36683; **GUS:** 107, F10020; 855, S36041; housing complex, F9602. TLC: divaricatic acid. DNA from a Glacier Bay specimen was used by Resl *et al.* (2015: isolate T1087) in a broader study of Ostropomycetidae.
*Massalongia carnosa* (Dicks.) Körb. — Muscicolous or over organic accumulations, often over rock, 0–115 m. **EA:** 870, S36570; between 870 and 871, S36585 (fertile); **WA:** 102, F9932; 319, S37924; 384, S38514; 549, T41628, T41633.
*Mastodia tesselata* (Hook. f. & Harv.) Hook. f. & Harv. — Saxicolous on the tops of intertidal boulders, sea level. **GUS:** 875, F9840 (sub *Lecanora poliophaea*), F9846, F9847; 876, S36751; 575, T41824; **WA:** 393, S38654; 550, T41641. Additional material from Glacier Bay was used by Garrido-Benavent *et al.* (2017*a*, *b*) in studies of the phylogeography of *M. tesselata*.
*Megalaria brodoana* S. Ekman & Tønsberg — Corticolous on *Alnus*, *Cupressus nootkatensis*, *Malus fusca* and *Tsuga heterophylla*, 0–86 m. **DUN:** 333, S38082; 412, S38914; 462, S39729; 562, T41744; **EX:** 433, S39081; [125, F10262, F10263]; [225, M2724]; **GUS:** 855, S36056; 330, S38061-B. TLC: nil. Only one previous Alaskan record, from Kodiak Island (Ekman & Tønsberg 1996).
*Megalaria pulverea (Borrer) Hafellner & E. Schreiner — Corticolous on *Alnus* and *Tsuga heterophylla*, 2 m. **EA:** 441, F10197; **GUS:** 855, S36015; 857, S36079; 559, T41733. TLC: atr, zeorin [no fpc].
*Melanelia commixta* (Nyl.) A. Thell — Saxicolous. Not seen during the present survey. **GB:** Muir Glacier, *Trelease* 1158 (NY, det. R. Harris, 1999, as *Cetrariella commixta*).
*Melanelia hepatizon* (Ach.) A. Thell — a) no substances ‘chemotype’: saxicolous in the alpine zone, 406–942 m. **DUN:** 423, S38978; **EX:** 370, S38341; 405, F10097 (cf.; not TLC'd but Pd−); 406, S38779; 408, S38862; 455, S39427. TLC: nil; b) stictic chemotype: saxicolous on rock in alpine tundra, 618–883 m. **DUN:** 428, S39005; **EX:** 405, S38772. TLC: stictic acid.
*Melanohalea exasperatula* (Nyl.) O. Blanco *et al.* — Corticolous on *Picea sitchensis* twigs on coastal headlands, 9–27 m. **DUN:** 339, S38171; 463, S39545; 467, S39654; **GB:** Willoughby Island, S36319 (sub *Physcia tenella*).
*Melanohalea multispora* (A. Schneid.) O. Blanco *et al*. — Corticolous on *Populus balsamifera* and *Salix*, usually at the back of uplifted beaches, 2–7 m. **EA:** 872, F9811; nr 438, S39312; terminus of Riggs Glacier, S40792; **WA:** 391, S38588, S38614; 395, S38662, S38680.
*Melanohalea olivacea* (L.) O. Blanco *et al.* — Corticolous on angiosperms; exact GLBA substratum not known. **Sector:** not known, reported for Glacier Bay by Esslinger (1977, as *Parmelia olivacea* ‘acid-deficient chemotype’, based on a specimen at US).
#*Merismatium decolorans* (Rehm ex Arnold) Triebel — Lichenicolous on *Biatora* sp. over *Alnus*, and on *Lobaria* sp., 34–60 m. **GUS:** 857, S36084; A578, P2191.
*Micarea botryoides (Nyl.) Coppins — Corticolous on *Picea sitchensis*, saxicolous on vertical shale outcrop, also on decaying bark flaps on tip-up and fungicolous on small polypore, 0–155 m. **DUN:** 131, F10443; 462, S39679; 463, S39508; **EX:** 444, S39327; **GUS:** 855, F9613; 856, F9629 (fertile); 876, S36725; 210, M2548.
*Micarea cinerea* (Schaer.) Hedl. — Corticolous on *Alnus* (7×), *Oplopanax horridus* (1×), *Picea sitchensis* (3×), *Tsuga heterophylla* (3×) and *Vaccinium ovalifolium* (1×), at low elevations, 0–195 m. **DUN:** 336, S38114; 462, S39701; **EA:** nr 438, S39297 (sub *Dendriscosticta wrightii*); 440, S39202; 441, F10198; 442, S39291; **GB:** 868, S36468 (sub *Leptogidium dendriscum*); 556, T41666; **GUS:** [V329, S38034]; 435, S39124 (sub *Scoliciosporum chlorococcum*); 855, S36033; 856, S36066, S36074; 857, S36138; 878, S36774; 224, M2705; 228, M2741; 237, M2837.
**Micarea czarnotae Launis *et al*. — Lignicolous on soft conifer wood, 230 m. **EX:** 108, F10044. TLC: methoxymicareic acid. Apothecia to 0.5 mm, with C+ violet pigments; ascospores 7–11 × 2.8–4 μm; thallus lacking crystals in the goniocysts. This new species was recently recognized within the *Micarea micrococca* complex by Launis *et al.* (2019) based on specimens from Finland, Poland and the Netherlands.
**Micarea farinosa Coppins & Aptroot — Saxicolous on rocks under tip-ups, 49–58 m. **DUN:** 332, S38069; **GUS:** 878, S36777, S36779. TLC: nil.
*Micarea inopinula* (Nyl.) Coppins & T. Sprib., ined. (syn. *M. prasinella* (Jatta) I. M. Lamb) — Corticolous, grading into muscicolous, mostly on *Tsuga heterophylla* trunks, 3–687 m. **DUN:** 334, S38098; 131, F10448; **EX:** 312, S37819; 448, S39358; [858, S36211, S36212]; 213, M2571; **GB:** 874, S36717 (sub *Gyalideopsis muscicola*); **GUS:** 143, F10496; 856, F9617, F9628, S36075; [860, F9667]; 882, S36844; 204, S38433 (sub *Xylographa trunciseda*); housing complex, F9859, F9860; [557, T41718a, T41718c]; 559, T41725. TLC: gyrophoric acid.
*Micarea melaena* (Nyl.) Hedl. — Lignicolous, 220 m. **EX:** 227, M2739.
**Micarea melaeniza Hedl. — Lignicolous on conifer wood, 50 m. **GUS:** 856, F9622. Ascospores unicellular; epihymenium dark brown, KOH−; hypothecium dark brown, KOH−; conidia 2–3 × 0.8–1 μm; pycnidial wall dark brown. Previously reported only from Sweden (Coppins 1983), Austria (Berger & Türk 1991) and Mongolia (Palka & Śliwa 2006).
*Micarea micrococca* (Körb.) Gams ex Coppins — Lignicolous on soft snag, also corticolous on *Alnus*, 22–45 m. **EX:** [225, M2712]; **GUS:** 204, S38440. TLC: methoxymicareic acid.
*Micarea misella* (Nyl.) Hedl. — Lignicolous in *Pinus contorta* muskeg and *Tsuga heterophylla* forest, 0–18 m. **EA:** 233, M2793; **GUS:** 316, S37885; road to Bartlett Cove dock, F9850, F9851, F9853, F9854, F9856, F9857.
*Micarea myriocarpa V. Wirth & Vězda ex Coppins — Saxicolous on vertical shale outcrops and growing on bark flaps under tip-up, 9–50 m. **DUN:** 462, S39678; 463, 39553; **EX:** [858, F9656].
*Micarea nigella Coppins — Lignicolous on dead tree, 20 m. **GUS:** 143, F10498. A collection from a dead tree behind the superintendent's house at Park HQ housing complex.
*Micarea paratropa* (Nyl.) Alstrup — Saxicolous on granitic boulders, 150–460 m. **DUN:** 116, F10146; 120, F10161; 121, F10177; 222, M2695.
*Micarea peliocarpa* (Anzi) Coppins & R. Sant. — Common, corticolous on *Alnus* (7×), *Malus fusca* (1×), *Picea sitchensis* (3×), *Pinus contorta* (1×), *Shepherdia canadensis* (1×), *Tsuga heterophylla* (2×); also once fungicolous on a bracket fungus and once saxicolous, common at low elevations, 0–180 m. **DUN:** 429, S39029; 462, S39719; 571, T41778; **EA:** 234, M2805; **EX:** [125, F10264]; [858, S36197]; [860, S36240]; 407, S38811 (sub *Lecidea roseotincta*); 433, S39076; **GB:** 864, S36339; 865, S36405; **GUS:** 139, F10483 (sub *Brianaria bauschiana*); 316, S37870, S37883 (as ‘aff.’); 317, S37895; 330, S38063 (sub *Fellhaneropsis vezdae*); 855, S36060; 856, F9627 (sub *Brianaria bauschiana*); 534, T41571; 556, T41676; 576, T41846; 857, S36136; Tower Rd, S37501; no waypoint, M2481; 230, M2764; **WA:** Marble Mtn beach, S38000 (sub *Pachyphiale fagicola*). TLC: gyrophoric, lecanoric (tr.), 5-*O*-methylhiascic acids. Some ascospores above the size range given by Coppins (2009).
*Micarea prasina* Fr. s. lat. — A common species, especially on old wood, in the outwash plain between Bartlett Cove and Gustavus and up into the muskegs below Excursion Ridge. Remarkably, it was not collected elsewhere in GLBA. Lignicolous (17×), corticolous on *Picea* or *Tsuga* (13×), and muscicolous (2×), 0–569 m. **EX:** 108, F10044; 112, F10063; [858, S36207]; 357, S38302; 358, S38317; 398, S38718; 403, S38749; [V431, S39055]; 444, S39321; no waypoint, M2584, M2586; 211, M2564; [225, M2723]; 227, M2733; **GUS:** 100, F9889; 855, S36043, S36044; 857, S36141 (as ‘*prasina* group’); 879, S36817, S36818; 882, S36840, S36841; 316, S37887; [V329, S38052]; 341, S38227, S38265, S38266; 204, S38439; 559, T41724, T41726; no waypoint, M2476; 215, M2580; 229, M2744; 230, M2754. TLC: micareic acid.
*Micarea synotheoides* (Nyl.) Coppins — Corticolous in coastal *Tsuga-Picea* forest, 20 m. **DUN:** 131, F10449 (sub *Opegrapha fumosa*).
*Micarea ternaria* (Nyl.) Vězda — Terricolous on *c*. 20 yr-old glacial foreland soils, *Dryas*-dominated habitats, 0–115 m. **EA:** 870, F9775.
*Micarea turfosa* (A. Massal.) Du Rietz — Muscicolous or terricolous on organic accumulations, 59–436 m. **DUN:** 219, M2677; 222, M2687a.
*Micarea xanthonica* Coppins & Tønsberg — Corticolous on *Picea sitchensis* and *Tsuga heterophylla*, and lignicolous on soft wood of snags, in beach fringe forests, 0–9 m. A highly localized species in GLBA, found only on the Taylor Peninsula and the beach fringe forest between Bartlett Cove and Point Gustavus. **DUN:** between 462 and 469, S39685; 560, T41738; 564, T41760d; **GUS:** 341, S38229, S38231, S38258; 876, S36726, S36753; [558, T41721]. TLC: xanthones.
*Microcalicium disseminatum* (Ach.) Vain. — Lignicolous on *Pinus contorta* snag, 236 m. **EX:** 399, S38727.
**#*Milospium graphideorum* (Nyl.) D. Hawksw. — Lichenicolous over unidentified white crust on *Picea sitchensis* bark, 0–10 m. **GB:** 868, S36545. This hyphomycete typically occurs over members of the genus *Lecanactis* s. lat., and is widespread in Europe (Hawksworth 1984).
*Miriquidica atrofulva* (Sommerf.) A. J. Schwab & Rambold — Saxicolous on metal-rich rock, 11 m. **EA:** 872, S36618.
*Miriquidica complanata (Körb.) Hertel & Rambold — Saxicolous on pebbles and larger cobbles near beaches, 0–100 m. **WA:** 101, F9926; 384, S38521; F9993 (as ‘cf.’; sub *Verrucaria* sp.); 105, F9997 (as ‘cf.’; sub *Amygdalaria consentiens*), F10005 (sub *Lecanora* aff. *polytropa*). TLC: miriquidic acid.
*Miriquidica gyrizans* Fryday — See ‘Descriptions of New Genera and Species’.
*#*Mixtoconidium nashii* **(**Hafellner) Etayo & van den Boom — Lichenicolous on *Ramalina*, corticolous on *Picea sitchensis* on coastal headland, 10 m. **DUN:** 467, S39651. A distinctive species of the California fog belt, usually occurring on *Niebla,* with ascomatal anthraquinones in the form of pruina. This is its northernmost record to date.
*#*Muellerella atricola* (Linds.) Sacc. & D. Sacc. — Lichenicolous, on *Tephromela atra* over rock, 937 m. **EX:** 409, S38880 (sub *Lecanora swartzii*).
#*Muellerella erratica* (A. Massal.) Hafellner & V. John — Lichenicolous on *Porpidia*, saxicolous in talus, 907 m. **EX:** 373, S38393; 374, S38405 (sub *Lecanora polytropa* group).
#*Muellerella hospitans* Stizenb. — Lichenicolous on *Mycoblastus*, corticolous on *Tsuga heterophylla* on coastal headland, 10 m. **DUN:** 467, S39662.
#*Muellerella lichenicola* (Sommerf. : Fr.) D. Hawksw. — Lichenicolous on *Mycoblastus affinis* and *Pertusaria subambigens*, 22 m. **GUS:** A569, P2247, P2322, P2325, P2406.
#*Muellerella pygmaea* (Körb.) D. Hawksw. — Lichenicolous on *Porpidia*, saxicolous, 937 m. **EX:** 407, S38837 (sub *Lecanora intricata*); 409, S38894.
#*Muellerella ventosicola* (Mudd) D. Hawksw. — Lichenicolous on ridgetop, on *Ophioparma* (?), 919 m. **EX:** 374, S38412 (sub *Lecanora* sp.).
*Multiclavula corynoides (Peck) R. H. Petersen — Tundra with bryophytes and *Dryas*, 115 m; limestone rocks and mossy ground on shore, 0–5 m. **EA:** 870, F9778; **GB:** N tip Willoughby Island, F9717.
*Multiclavula mucida* (Pers.) R. H. Petersen — Lignicolous on log, 33 m. **GUS:** 330, S38064.
*Mycobilimbia epixanthoides* (Nyl.) Vitik. *et al.* — On organic accumulations over granitic rocks, also corticolous over *Alnus*, 8–92 m. **EA:** 440, S39220; **GUS:** [558, T41720; Tower Rd, S40726]; **WA:** 103, F9956, F9957 (sub *Protopannaria pezizoides*); 387: S38542. TLC: nil.
*Mycobilimbia tetramera* (De Not.) Vitik. *et al.* — Corticolous on *Populus balsamifera* (6×) and on organic accumulations/detritus in snowbed (1×), and muscicolous over seepy rock (1×), 2–92 m. **EA:** 123, F10250; 440, S39240, S39242; 441, F10219 (sub *Toniniopsis subincompta*); 233, M2794a; **GUS:** 877, S36758; **WA:** 385, S38531; 387, S38543.
*Mycoblastus affinis* (Schaer.) T. Schauer — Corticolous on *Alnus*, *Picea sitchensis*, *Pinus contorta* and *Tsuga heterophylla*, also lignicolous, 0–687 m. An abundant epiphytic lichen in GLBA on oligotrophic bark, far more so than the related *M. sanguinarius*. **DUN:** nr 334, S38107; 339, S38149, S38198; 462, S39716; 464, S39603, S39609; 467, F10435; 562, T41749; 570, T41774; **EX:** 448, S39345; [858, F9649, S36168 (sub *Platismatia norvegica*)]; [859, S36229]; 227, M2738; **GB:** N Sandy Cove, S36647; 868, S36473, S36506; 874, F9831, S36682, S36702; **GUS:** 316, S37868, S37877; 855, S36002; 862, F9679, S36289; 876, S36733; 879, S36800; 559, T41731. TLC: atr, planaic acid.
*Mycoblastus caesius* (Coppins & P. James) Tønsberg — Corticolous on *Alnus* (11×), especially on trunks, *Malus fusca* (1×), *Menziesia ferruginea* (1×), *Picea sitchensis* (2×) and *Tsuga heterophylla* twigs (2×), 0–46 m. **DUN:** 333, S38081; 462, S39714, S39740, S39730; 467, S39663; 562, T41754, T41755; **EX:** [858, S36172]; [859, S36183]; 433, S39073; **GB:** 868, S36466; 874, S36703; 556, T41672, T41687; **GUS:** 856, S36068; 857, S36086, S36132; 879, S36797, S36798; 341, S38235; 436, S39139; Tower Rd, S38271; 531, T41540; 533, T41564; 535, T41572; 536, T41574; 559, T41732; 224, M2704a; 230, M2758. TLC: perlatolic acid, ±fatty acid A1-2/C1-2. Not a *Mycoblastus*, but its correct placement requires more study.
*Mycoblastus sanguinarioides* Kantvilas — Corticolous on *Pinus contorta*, 32–213 m. **EX:** 312, S37812; **GUS:** 862, S36285; 210, M2554.
*Mycoblastus sanguinarius* (L.) Norman — Corticolous on *Alnus*, *Picea sitchensis* and *Tsuga heterophylla*, 0–687 m. **DUN:** 462, S39741; **EA:** 440, S39198, S39206 (sterile, sorediate morph); **EX:** 448, S39349; **GB:** 874, S36705; **GUS:** 857, S36087; 876, S36727.
*Mycocalicium subtile* (Pers.) Szatala — Lignicolous on *Pinus contorta* snag, 18 m. **GUS:** 316, S37860.
*Mycoporum antecellens (Nyl.) R. C. Harris — Corticolous on *Alnus, Menziesia*, *Salix* and *Tsuga*, 1–50 m. **DUN:** 462, S39711; **EX:** [858, S36176, S36179]; **GUS:** 857, F9643 (sub *Micarea* sp.), S36078, S36148; 341, S38226; 341, S38226.
*Myochroidea leprosula* (Arnold) Printzen *et al*. — Terricolous and muscicolous in alpine sod, 900 m. **EX:** 218, M2615.
*Myriolecis* aff. *contractula* Nyl. — Saxicolous on upper intertidal rocks, 0–5 m. **DUN:** 134, F10463; 462, F10379; F10407; **GB:** 864, F9735; 873, F9817; **WA:** 322, S37967. TLC: two xanthones (F10379). We follow Brodo (2010) in treating this as ‘aff.’, not an unambiguous match for the type. For comments on the nomenclature of *Myriolecis*, see *Lecanora poliophaea*.
*Myriolecis persimilis* (Th. Fr.) Śliwa *et al*. — Corticolous on *Alnus* and *Shepherdia canadensis*, 0–10 m. **GB:** Marble Mtn beach, S38014 s. lat.); **WA:** 384, S38508.
*Myriolecis schofieldii (Brodo) Śliwa *et al*. — Saxicolous on shorelines, sea level. **EA:** near 438, S39188. A DNA sequence from this specimen was used to confirm its phylogenetic placement (Fig. 10).
*Myriolecis straminea* (Ach.) Śliwa *et al*. — Saxicolous on rocky seashore and on sea stacks, 0–9 m. **DUN:** 339, S38210; 463, S39585. TLC: numerous xanthones. The xanthone profile of the GLBA specimens diverges from those of Norwegian specimens of *L. straminea* in GZU, but we did not test whether this difference is consistent between a larger sample of North American and European thalli.
*Myriolecis torrida* (Vain.) Śliwa *et al*. — Saxicolous on calcareous rock. **GB:** Muir Glacier, specimen cited by Śliwa (2007). The report is based on *Trelease* 933 (NY), collected in the 19th century, at which time the terminus of Muir Glacier would have been in GB, not EA.
**Myriospora dilatata (M. Westb. & Wedin) K. Knudsen & Arcadia — Saxicolous on iron-rich rock, 11–22 m. **EA:** 872, S36628; **WA:** 318, S37905 (det. M. Westberg).
**Myriospora myochroa (M. Westb.) K. Knudsen & Arcadia — Saxicolous on rocky headland, 9 m. **DUN:** 463, S39581 (det. M. Westberg, as ‘cf.’). Another Alaskan specimen, from the interior, was recently annotated to this: Eagle Summit, 1977, *T. Ahti* 25184 (H, det. K. Knudsen 2018; T. Ahti, personal communication).
**Myriospora tangerina (M. Westb. & Wedin) K. Knudsen & Arcadia — Saxicolous on gneiss rocks on shore, in tidal flats and on rock under tip-up on coastal headland, 2–9 m. **DUN:** 463, S39593 (det. M. Westberg); **GB:** 865, F9736; 873, F9822 (det. K. Knudsen). This and the previous two species were described (as *Silobia*) by Westberg *et al.* (2011), who provided a key and colour plates. None have been reported from North America until now.
#*Nectriopsis lecanodes* (Cesati) Diederich & Schroers — Lichenicolous on *Peltigera* on grassy beaches or on organic accumulation in crevices of bedrock outcrops, 0–3 m. **DUN:** 462, S39499; [**GUS:** State Dock, S36846].
*Nephroma arcticum* (L.) Torss. — Uncommon, terricolous in snowbeds and muscicolous on thick moss mats over logs in high montane old-growth *Tsuga* forest, 432–903 m. **EX:** 355, S38300; between 405 and 406, S38777; 456, S39475.
*Nephroma bellum* (Spreng.) Tuck. — Corticolous on *Alnus*, *Picea sitchensis*, *Populus balsamifera* and *Salix*, 0–124 m. **DUN:** 334-V338, S38104; 339, S38168; 462, S39706; 463, S39542; **GB:** 864, S36331; 867, S36432; 868, S36460 (sub *Nephroma resupinatum*); **GUS:** 436, S39149; **WA:** 391, S38611.
*Nephroma helveticum* Ach. subsp. *sipeanum* (Gyeln.) Goward & Ahti — Corticolous on *Alnus* and *Picea sitchensis*, beach fringes, 2–9 m. **DUN:** 463, S39542 (sub *Nephroma bellum*); **EA:** 123, F10247; 440, S39234; 441, F10201; **EX:** [859, F9662]; **GB:** N Sandy Cove, F9812; **GUS:** 857, F9631, F9632, F9633, S36128 (sub *Parmeliella triptophylla*), S36133; [557, T41704]; 233, M2791.
*Nephroma isidiosum* (Nyl.) Gyeln. — Corticolous on *Alnus* (11×), *Populus balsamifera* (3×) and *Salix* (1×), mostly in beach fringes or in the forest immediately behind them, 2–11 m. **EA:** 123, F10240; 872, S36607; near 438, S39305, S39295; 440, S39207, S39214, S39244; 441, S39273, F10212, F10213; 233, M2777, M2800; no waypoint, M2823b; **GB:** 864, S36367; 868, S36469; **GUS:** 138, F10473; 857, S36085, S36151; 576, T41830; **WA:** 395, S38673; Blue Mouse Cove plot BM1a (GLBA herbarium).
*Nephroma parile* (Ach.) Ach. — Corticolous on *Alnus*, *Salix* and *Viburnum edule*, and saxicolous on argillite and limestone, 0–18 m except for one saxicolous record at 880 m. **EA:** 872, S36602; **EX:** 459, F10353; **GB:** 863, F9690; 864, S36324; 865, S36399; 866, S36423; **GUS:** 138, F10478; 879, S36793; 341, S38262; **WA:** 102, F9942; 391, S38613; Blue Mouse Cove plot BM1a, BM2a (GLBA herbarium).
*Nephroma resupinatum* (L.) Ach. — Corticolous on *Alnus* and *Populus balsamifera*, in beach fringes and once in successional alder scrub, 0–10 m. **EA:** 441, F10214, F10215, S39285; 233, M2798; **GB:** 864, S36365; 866, S36425; 868, S36460; **GUS:** 436, S39138 (with lobuli); **WA:** SE corner of Gilbert Island, by Blue Mouse Cove, 1968, *Worley* 10693 (UBC, n.v.); Blue Mouse Cove plot BM1a (GLBA herbarium).
#*Nesolechia fusca* (Triebel & Rambold) Pérez-Ort. — Lichenicolous on *Hypogymnia lophyrea*, 10 m. **GB:** 868, S36537.
#*Nesolechia oxyspora* (Tul.) A. Massal. — Lichenicolous on *Hypogymnia*, *Parmelia* and *Platismatia*, these corticolous over *Picea* or lignicolous, 0–27 m. **DUN:** 339, S38197 (sub *Lichenopuccinia poeltii*); 583, P2204, P2256; 586, P2093, P2095; **GUS:** 434, S39095; 857, S36163; 876, S36748.
*#*Niesslia cladoniicola* D. Hawksw. & W. Gams — Lichenicolous on *Cladonia amaurocraea*, terricolous, 922 m. **EX:** 407, S38822 (sub *Bachmanniomyces uncialicola*).
#*Niesslia peltigerae* Pérez-Ort. — See ‘Descriptions of New Genera and Species’.
*Normandina acroglypta* (Norman) Aptroot — Muscicolous on epiphytic mosses on *Alnus* bark, 2–20 m. **DUN:** 129, F10364; **GB:** S Sandy Cove, S40755; **GUS:** Tower Rd, S40725. Previously reported for North America and Alaska by McCune *et al.* (2018). S40755 possesses a nearly corticate thallus.
*Normandina pulchella* (Borrer) Nyl. — Corticolous or muscicolous on loosely adhering mosses over *Alnus*, *Populus balsamifera* and *Picea sitchensis*, also once over rock, 0–86 m. **DUN:** 333, S38085; **EA:** nr 438, S39307; 440, S39190, S39243 (fertile); 442, S39290 (sub *Lobaria hallii*); 233, M2786; **EX:** [125, F10261]; **GB:** 864, S36369; 867, S36442 (sub *Leptogium saturninum*); 868, F9754, S36471; S Sandy Cove, S40757; **GUS:** 857, S36158; 881, S36831; 397, S38710; 436, S39163; Tower Rd, S37513; 533, T41561; [574, T41813].
*Ochrolechia androgyna* (Hoffm.) Arnold, s. lat. — Corticolous on *Pinus contorta* trunk in muskeg and outwash plain meadows, also on *Picea sitchensis* and *Tsuga heterophylla* on coastal headlands, 0–192 m. **DUN:** 467, F10429, S39649, S39661; [**EX:** 858, S36215]; 311, S37808; 409, S38874; **GB:** 868, S36479; **GUS:** 316, S37876. TLC: gyrophoric, lecanoric acids, fatty acid, androgyna B unknowns [identical or close to *O. androgyna* s. str.].
*Ochrolechia brodoi* Kukwa — Corticolous on *Picea sitchensis* twigs, krummholz, 883 m. **EX:** 405, S38767. Recently reported as new to North America, from Alaska by Brodo & McCune (2017).
*Ochrolechia cooperi* T. Sprib. — See ‘Descriptions of New Genera and Species’.
*Ochrolechia frigida* (Sw.) Lynge (incl. f. *lapuensis* (Vain.) Coppins) — Terricolous on alpine turf, 903–922 m. **EX:** 407, S38857; 455, S39444.
*Ochrolechia juvenalis* Brodo — Corticolous on *Picea sitchensis* twigs and *Pinus contorta*, also lignicolous once on beach log, 0–922 m. **DUN:** 464, S39604; **EX:** 405, S38767; 407, S38805; **GUS:** 862, S36297; 876, S36749. TLC: gyrophoric, lecanoric, ±variolaric acids, ±fatty acid or low-running xanthone.
*Ochrolechia laevigata* (Räs.) Vers. ex Brodo — Corticolous on *Alnus* bark, sea level. **GB:** 864, S36325. TLC: gyrophoric, lecanoric acids, high-running fatty acid.
*Ochrolechia montana Brodo — Corticolous on *Pinus contorta*, 20–250 m. **DUN:** 464, F10421; **EX:** 110, F10057; 312, S37810; **GUS:** 862, S36281. TLC: gyrophoric, lecanoric acids, unidentified fatty acid.
*Ochrolechia oregonensis* H. Magn. — Corticolous on *Alnus*, *Picea sitchensis*, *Populus balsamifera* and *Tsuga heterophylla*, 0–687 m. **EA:** 440, S39248; **EX:** 448, S39350; **GB:** 865, S36404; **GUS:** Park HQ, S36165; 100, F9885; 107, F10032; 876, S36736. TLC: gyrophoric, lecanoric acids, ±fatty acids. The specimens from *Populus* (S39248) and *Alnus* (S36325, S36404) have thinner thalli and smaller apothecia (mostly 1.5 mm) than specimens from conifer bark, but agree with *O. oregonensis* in the localization of C reactions (apothecial cortex C+ pink, medulla C−, thalline cortex C−, subcortex C+ pink, medulla C−). The ascospores in S36404 are also on the upper end of the dimensions provided by Brodo (1991), 65–82 × 24–33 μm (ascospores in S39248 were poorly developed or collapsed but reached the same size range).
*Ochrolechia subplicans* (Nyl.) Brodo subsp. *hultenii* (Erichs.) Brodo — Saxicolous on gneiss and argillite, found on rocky seashores and alpine outcrops, 0–27 m and 895–903 m. **DUN:** 339, S38211; 462, F10366, S39755; 463, S39584; **EX:** 370, S38350; 405, F10097 (sub *Melanelia hepatizon*); 407, F10122 (sub *Lecanora* sp. F10122); 409, S38887 (sub *Rhizocarpon inarense*); 455, S39431. A predominantly Aleutian species studied in detail by Brodo (1988) and until now known to extend only as far east as the Chugach National Forest in south-central Alaska and a disjunct locality in Oregon. TLC: alectoronic acid. DNA sequences of *O. subplicans* subsp. *hultenii* from GLBA (from S38211 = isolate P127, from shoreline rocks, and S38350 = isolate T1300, from alpine rocks) were published by Resl *et al.* (2015) as part of a larger survey of Ostropomycetidae and are included in Fig. 6. According to data provided by Brodo (1988) the alpine specimens should, from their ecology, conform to *O. subplicans* subsp. *subplicans*, but they did not sufficiently correspond in morphology; the ITS rDNA sequences from shoreline species versus the alpine specimen differed in four substitutions. See also discussion of alectoronic acid-containing *Ochrolechia* species under ‘Other Species Treated in Detail’.
*Ochrolechia szatalaënsis* Verseghy — Corticolous on *Alnus*, *Populus balsamifera* and *Salix*, 4–32 m. **EA:** near 438, S39313; 440, S39199, S39209; **GUS:** 862, S36312; **WA:** 395, S38686. TLC: variolaric acid.
*Opegrapha fumosa* Coppins & P. James — Corticolous on *Picea sitchensis* and *Tsuga heterophylla* associated with conifer woodland immediately behind beach fringe, 0–20 m. **DUN:** 131, F10449; **GUS:** 857, S36157; 341, S38239; 436, S39140, S39152.
#*Opegrapha geographicola* (Arnold) Hafellner — Lichenicolous on *Rhizocarpon geographicum* aggr. in alpine heath with rock outcrops, on alkaline argillite, 890 m. **EX:** 453, F10321. This species was reported as new for North America by Dillman *et al.* (2012).
#*Opegrapha sphaerophoricola* Isbrand & Alstrup — Lichenicolous on *Sphaerophorus venerabilis*, corticolous on *Pinus contorta* and *Tsuga heterophylla*, 0–192 m. **DUN:** 339, S38180; **EX:** 564, P2112; **GUS:** 587, P2109, P2189; 210, M2562; **WA:** A575, P2297.
#*Opegrapha thelotrematis* Coppins — Lichenicolous on *Thelotrema* sp. on *Alnus*, 84 m. **DUN:** 562, T41750 (sub *Ropalospora viridis*).
*Ophioparma lapponica* (Räsänen) Hafellner & R.W. Roger — Seen only once, saxicolous on rock in the alpine zone, 900 m. **EX:** 218, M2627. Ascospores single-celled, *c.* 20 × 3.5 μm.
*Oxneriaria mashiginensis (Zahlbr.) S. Y. Kondr. & Lőkös (syn. *Aspicilia mashiginensis* (Zahlbr.) Oxner; synonymy after Moniri *et al.* 2017) — Saxicolous on outcrop just above sea-level beach, ice-free since 1970s, *c.* 3–5 m. **EA:** 551, S40763 (det. T. Wheeler).
*Oxneriaria aff. *permutata* (Zahlbr.) S. Y. Kondr. & Lőkös (syn. *Aspicilia permutata* (Zahlbr.) Clauzade & Rondon) — Saxicolous on siliceous cobbles on uplifted beaches and over limestone bedrock, 2–15 m. **WA:** 321, S37961; 381, S38466. Ascospores 17–21 × 10–13 μm, few developed; epihymenium olive to brown-olive; hymenium 150–180 μm tall, excipulum 30–40 μm wide; paraphyses moniliform to submoniliform. Conidia not found. TLC: nil. Previously reported from North America under the name *Lecanora permutata* Zahlbr. by Ahti *et al.* (1973) from the Reindeer Preserve near Inuvik, Northwest Territories.
*Pachyphiale fagicola* (Arnold) Zwackh — Corticolous on *Alnus*, 0–4 m. **GB:** 864, S36350; Marble Mtn beach, S38000; **WA:** 327, S38011 (sub *Ochrolechia* sp. S38011). Ascospores in S38000 *c.* 31 × 4 μm, 7-septate, polysporous in ascus.
*Parmelia hygrophila* Goward & Ahti — Corticolous on *Alnus*, *Picea sitchensis*, *Pinus contorta*, *Populus balsamifera* and *Tsuga heterophylla*, 3–32 m. **DUN:** 339, S38188; **EA:** 872, S36599; **GB:** 874, S36680; **GUS:** 862, S36291; [329, S38051; Tower Rd, S40729, S40734]; **WA:** 395, S38675. TLC: atr, salazinic acid. This appears to be an extraordinarily variable species with respect to thallus morphology. Specimen S36599 is unusual in having a pruinose/crystalline upper surface and was initially considered to be similar to the European *Parmelia ernstiae* Feuerer & A. Thell. However, an ITS sequence (isolate T1335, Table 1) is identical to published sequences of *P. hygrophila* (e.g. GenBank Accession KT625508). Similarly, specimens S38051 and S40729 are distinct from all local *Parmelia* collections in the lack of laminal pseudocyphellae and the habit, with terminal and marginal isidioid soredia. However, here too an ITS sequence (isolate T1336, Table 1) suggests a close relationship with *P. hygrophila*, although it contains numerous ambiguous base calls, suggesting the presence of two closely related PCR templates.
*Parmelia omphalodes* (L.) Ach. — Saxicolous in alpine heath with rock outcrops; on alkaline argillite, 883–922 m. **EX:** 405, F10085; 407, F10108 (sub *Hypogymnia vittata*); 453, F10322 (sub *Tephromela atra*); 459, F10359.
*Parmelia pseudosulcata* Gyeln. — Corticolous on *Pinus contorta* in muskeg, 213 m. **EX:** 312, S37809. TLC: atr, lobaric acid, fpc.
*Parmelia saxatilis* (L.) Ach. Two morphs were identified: a) the ‘typical’ morph (isidia barrel-shaped and all over thallus): saxicolous on rocky headlands and upland rock outcrops, and corticolous over *Alnus* and *Pinus contorta*, 0–618 m. **DUN:** 338, S38132; 339, S38156; 428, S39013; 463, S39569, S39583; **EX:** 312, S37814; 455, S39426; **GB:** 864, S36338; slopes and ridge of mainland immediately NW of Sebree Cove and W of Caroline Point, *Worley* 10823 (UBC n.v.); **WA:** Blue Mouse Cove plot BM1a, BM2a (GLBA herbarium). TLC: atr, salazinic acid. *Parmelia saxatilis* is remarkable for occurring almost exclusively in areas unglaciated during the Little Ice Age, with the exception of the records from Marble Mtn and Caroline Pt; b) ‘*marginalis*’ morph (isidia fine and marginal): corticolous on *Alnus*, *Picea sitchensis* and *Tsuga heterophylla* and lignicolous on *Picea* branch, 9–27 m. **DUN:** 339, S38187, S38188 (sub *Parmelia hygrophila*); 462, S39734, S39736; 464, S39611; 467, S39665; **GUS:** 204, S38435. TLC: atr, salazinic acid, also 3 × lobaric acid. The divergent chemistry and morphology, and the sympatric occurrence of this morph, suggest it is a separate, as yet unnamed entity distinct from *P. saxatilis* (T. Goward, personal communication); it was distinguished by Spribille *et al.* (2010) from KLGO, as *Parmelia* S24712.
*Parmelia squarrosa* Hale — Corticolous on *Alnus*, *Picea sitchensis*, *Populus balsamifera* and *Tsuga heterophylla*, mostly on twigs, associated with beach fringe, 3–32 m. **DUN:** 339, S38158, S38189; **EA:** 441, S39267; **GB:** 868, S36503, S36528; **GUS:** 862, S36317 (as ‘aff.’); 857, S36099, S36130. TLC: atr, salazinic acid.
*Parmelia sulcata* Taylor — Two morphs were distinguished: a) the ‘typical’ morph: corticolous on *Alnus*, *Pinus contorta*, *Populus balsamifera* and *Salix*, 0–32 m. Perhaps only associated with beach fringe or under collected. **DUN:** 462, S39737; **GB:** 864, S36344; **GUS:** 862, S36287, S36305; 857, S36081, S36149; **WA:** 395, S38659; Blue Mouse Cove plot BM1a, BM2a (GLBA herbarium). TLC: atr, salazinic acid; b) alpine ‘punctiform’ morph: terricolous or loosely over rock, 895–903 m. **EX:** 370, S38340, S38344; 455, S39429. TLC: atr, salazinic acid. In this morph numerous punctiform soralia are present, in addition to elongate soralia, and the soredia are smaller and more darkly pigmented.
*Parmeliella parvula* P. M. Jørg. — Corticolous on *Tsuga heterophylla* twigs, 50–58 m. **EX:** [858, S36203, S36223]; **GUS:** 878, S36770; [557, T41712 (det. P. M. Jørgensen, 2014)].
*Parmeliella triptophylla* (Ach.) Müll. Arg. — Corticolous on *Alnus*, *Cupressus nootkatensis*, *Oplopanax horridus*, *Populus balsamifera*, *Ribes lacustre*, *Salix* and fine *Tsuga heterophylla* twigs, low elevation forest, 1–46 m. **DUN:** 467, S39670; **EA:** 441, F10199, F10211, S39270; **GUS:** 341, S38253; 436, S39146; 855, S36004 (sub *Gyalideopsis epicorticis*); 857, S36093, S36128, S36156; 862, S36307; 879, S36794; 881, S36829; Tower Rd, S37502, S37507, S38274; 531, T41529; 533, T41558; 210, M2552. DNA was extracted from two GLBA specimens to assess whether any divergent speciation patterns could be detected, but ITS sequences (isolates T1212 and T1213, Table 1) were identical at all positions to existing GenBank sequences (e.g. accession HM448804 from the UK).
*Parmeliopsis ambigua* (Hoffm.) Nyl. — Corticolous on *Picea sitchensis* twigs in krummholz, 883–922 m. **EX:** 405, S38768; 407, S38812. TLC: usnic, divaricatic acids. Rare in GLBA.
*Parmeliopsis hyperopta* (Ach.) Arnold — Corticolous on *Picea sitchensis*, *Pinus contorta* and *Tsuga heterophylla*, 59–883 m but probably also to sea level; under collected. **DUN:** 337, S38123; **EX:** [860, S36252]; 357, S38301; 405, S38759. The more common *Parmeliopsis* species in GLBA.
*Peltigera britannica* (Gyeln.) Holt.-Hartw. & Tønsberg — Two commonly recognized morphs were found in GLBA: a) the cyanomorph: corticolous on *Tsuga heterophylla*, 60 m. **GUS:** 856, S36065; b) the chloromorph: terricolous in forests and corticolous or lignicolous on tree trunks (*Alnus*, *Tsuga*) and snags, 0–687 m. **DUN:** 334-V338, S38105; **EX:** 108, F10041; [125, F10254]; [858, S36178]; 358, S38323; 448, S39353. Under-recorded.
*Peltigera castanea* Goward *et al*. — On organic accumulations in rock crevices on S-facing limestone wall. **GB:** 867, S36437.
*Peltigera chionophila* Goward & Goffinet — Terricolous, on moss hummocks and in snowbeds in montane to subalpine forest and parkland, also corticolous once on the lower trunk of *Tsuga mertensiana*, 333–903 m. **EX:** 112, F10065; 353, S38290; 406, S38791 (as ‘cf.’); 410, S38909; 422, S38964; nr 455, S39455; 460, F10362.
*Peltigera collina* (Ach.) Schrad. — Corticolous on *Alnus*, *Picea sitchensis* and *Populus balsamifera* and on organic accumulations on rock outcrops, mostly 0–37 m but two records at 872–880 m. **DUN:** 462, S39717; 463, S39558; **EA:** 872, S36614; nr 438, S39306; 442, S39288; **EX:** [V431, S39042]; 456, S39469; 459, F10356; **GB:** 868, S36455; **GUS:** 138, F10471; **WA:** 395, S38674; Blue Mouse Cove plot BM1a, BM2a (GLBA herbarium).
*Peltigera didactyla* (With.) J. R. Laundon — Terricolous, 10–883 m. **EA:** 870, S36584; 872, S36622; **EX:** 405, F10088 (sub *Scytinium gelatinosum*); **WA:** 384, S38526; 549, T41631 (s. lat.).
*Peltigera kristinssonii* Vitik. — Terricolous in *Luetkea-Cassiope mertensiana* snowbed, on log in *Lysichiton* muskeg, also as host of *Niesslia peltigerae*, 333–499 m. **DUN:** 422, S38966; **EX:** 447, S39343; 461, S39497.
*Peltigera lepidophora* (Nyl. ex Vain.) Bitter — In moss over rock, and in organic accumulations over marble, 11–30 m. **EA:** 872, S36621; **GB:** 867, S36451; **WA:** 103, F9953 (sub *Lepraria vouauxii*), F9959.
*Peltigera leucophlebia* (Nyl.) Gyeln. — a) s. str.: corticolous on base of *Tsuga* in old-growth forest, 40 m. **GUS:** 855, S36000; b) ‘*conspersa*’ morph ( = *Peltigera* sp. S24836 of Spribille *et al.* 2010): terricolous on sandy beaches and road banks, 3–39 m. **GUS:** [State Dock, S36852;] 880, S36825; c) not assignable to morph: terricolous between rocks, in heath and in forest, 49–937 m. **DUN:** just above 332, S38071; **EX:** 371, S38363; 409, S38875; 453, S39370; 454, S39412; **GUS:** 878, S36780, S36789.
*Peltigera malacea* (Ach.) Funck — Terricolous on dense short turf beneath limestone crags, also on looser soil in alpine heath, 10–889 m. **EX:** 453, S39380; **WA:** 104, F9960.
*Peltigera membranacea* (Ach.) Nyl. Three morphs were recognized that all appear to be referable, at this time, to *P. membranacea*. These were a) the typical, tomentose morph: terricolous on coastal headlands, on beach ridges and over marble outcrops, also on mossy base of tree, 0–230 m. **DUN:** 467, S39676; **EA:** 438, S39181; **EX:** 108, F10040; **GB:** 864, S36392, S36394; **GUS:** 855, S36047; 876, S36754; b) a glabrous morph: terricolous, 9–27 m. **DUN:** 339, S38217; 462, S39754. This morph was studied by S. Werth (now Munich, Germany) as part of a global microsatellite study of variation in the fungal partner in *P. membranacea* and was found not to differ significantly from the typical form of this species; c) dark-veined morph: terricolous, 0–903 m. **EA:** 438, S39178, S39179; **EX:** 371, S38365; nr 455, S39456. This morph is similar to the typical morph but with unusually dark-pigmented veins.
*Peltigera neckeri* Hepp ex Müll. Arg. — Terricolous on beach ridge, 4 m. **EA:** 438, S39180.
*Peltigera neopolydactyla* (Gyeln.) Gyeln. — Terricolous on moss and coarse organic debris over logs, 0–25 m. **GUS:** 855, S36049, S36050; 856, S36070; 878, S36772.
*Peltigera occidentalis* (E. Dahl) Kristinsson, s. lat. — Terricolous or on logs, 27–569 m. **DUN:** 339, S38218; **EX:** 358, S38320.
*Peltigera pacifica* Vitik. — Terricolous on coarse organic material on beach log, also corticolous on *Alnus*, 9–40 m. **DUN:** 462, S39749; **EX:** [125, F10265]; [V431, S39041].
*Peltigera polydactylon* (Neck.) Hoffm. — Terricolous on sandy beaches and soil up to the alpine zone, 0–903 m. **DUN:** 462, S39745 (s. lat.); **EX:** between 405 and 406, S38774, S38776; 410, S38910; 456, S39474 (s. lat.); **GB:** 864, S36393 (unusual in vein darkening pattern); 873, S36665; **GUS:** Tower Rd, S37523; 341, S38264; **WA:** 382, S38477.
*Peltigera ponojensis* Gyeln. — a) typical morph: terricolous on organic accumulations or over rock, on moss over rock, on uplifted beaches and sandy soil as well as lignicolous on a stump, 0–40 m. **EA:** 872, S36634, S36635; **GB:** 873, S36661; **GUS:** [State Dock, S36854, S36855;] Tower Rd, S37537; 876, S36756; **WA:** 388, S38553; b) polyphyllous morph: terricolous on organic accumulations between cracks in bedrock exposed to salt water during storm surges (0–2 m), also one record from soil in the alpine zone, 895 m. **DUN:** 462, S39745; 463, S39559; **EX:** [125, F10268]; 370, S38353; **GB:** 867, S36452.
*Peltigera praetextata* (Flörke ex Sommerf.) Zopf, s. lat. — a) grey morph, brown when wet: terricolous on soil and organic accumulations over bedrock, 0–52 m. **GB:** 873, S36666, S36667, S36671; **WA:** 319, S37951 (as ‘aff.’); 382, S38478. Probably not corresponding to *P. praetextata* in the sense of the type, but part of a wider western North American species complex; b) green morph, blue-green when wet: terricolous in early successional *Alnus* thickets, 11 m. **EA:** 872, S36633.
*Peltigera rufescens* (Weiss) Humb. — Terricolous on moss over rock, on young soils in glacial forelands, and on soil accumulations over low seaside crags, 0–43 m. **EA:** 869, S36550 (as ‘cf.’); 872, S36620; **WA:** 104, F9973; 318, S37911.
*Peltigera scabrosa* Th. Fr. — Terricolous on log and over granitic rock, 432–889 m. **DUN:** 120, F10162; **EX:** 355, S38299; 453, S39372.
*Peltigera venosa* (L.) Hoffm. — Terricolous on sandy road bank, 20 m. **GUS:** 880, S36819; NPS housing complex, F9862.
*Pertusaria alpina* Hepp ex Ahles — Corticolous on *Alnus* in shoreline fringe, 4–8 m. **GB:** 864, S36348; 865, S36402. TLC: stictic acid. This species was found on *Alnus* on both the west and east (Shag Cove) sides of Marble Mtn, and nowhere else.
*Pertusaria bryontha* (Ach.) Nyl. — Lichenicolous on *Peltigera*, and saxicolous, 922–937 m. **EX:** 407, S38821; 409, S38900.
*Pertusaria carneopallida* (Nyl.) Anzi — Corticolous on *Alnus*, 0–33 m. **EA:** Muir Inlet, S36596; terminus of Riggs Glacier, S40789; **GB:** 868, S36475 (sub *Ochrolechia* sp. S38011); **GUS:** [V329, S38056]; 531, T41523; **WA:** 551, T41643 (sub *Caloplaca* sp.).
*Pertusaria coriacea* (Th. Fr.) Th. Fr. — Muscicolous in alpine heath with alkaline argillite rock outcrops, 883 m. **EX:** 405, F10092.
*Pertusaria flavocorallina* Coppins & Muhr — Corticolous on *Alnus* (4×), *Picea sitchensis* (1×) and *Salix* (1×), in beach fringe, also once terricolous, 2–8 m. **DUN:** 417, S38949; **EA:** near 438, S39298; 440, S39227; 441, S39281; **GUS:** 857, S36114; [557, T41694; State Dock, S36850]; **WA:** 391, S38616. TLC: thiophaninic acid.
*Pertusaria glaucomela* (Tuck.) Nyl. — Corticolous on *Picea sitchensis*, especially twigs, apparently the sole phorophyte substratum, always in beach fringe, 0–11 m. **DUN:** 463, S39534; 566, T41763; **EA:** 872, S36613; **GB:** 865, S36410; 857, S36124; 868, S36494; Willoughby Island, S36319 (sub *Physcia tenella*), S36320 (sub *Polycauliona pollinarioides*); **GUS:** 107, F10031; 533, T41562. TLC: gyrophoric acid, cf. 5-*O*-methylhiascic acid. DNA was isolated from one specimen (P191, Table 1).
*Pertusaria mccroryae* Björk *et al*. — Corticolous on shaded *Picea sitchensis* and *Tsuga* trunks, 0–687 m. **DUN:** 560, T41738 (sub *Micarea xanthonica*); **EX:** 448, S39351. TLC: 5-*O*-methylhiascic acid.
*Pertusaria oculata* (Dicks.) Th. Fr. — Muscicolous over rock, exposed summits and alpine zone, 618–903 m. **DUN:** 428, S39001; **EX:** 455, S39451.
*Pertusaria sommerfeltii* (Flörke ex Sommerf.) Fr. — Corticolous on *Alnus* (5×), *Populus* (1×), *Salix* (2×), *Viburnum* (1×), 2–33 m. **EA:** 872, S36603; 440, S39205; **GB:** 866, S36417; 867, S36429; **GUS:** 857, S36154; 316, S37866; [V329, S38019]; 341, S38259; Tower Rd, S38280; **WA:** 391, S38606. TLC: lichexanthone. The common poriform *Pertusaria* on bark in GLBA.
*Pertusaria subambigens* Dibben — Corticolous on *Alnus* (1×), *Picea sitchensis* (6×), *Pinus contorta* (3×) and *Tsuga heterophylla* (1×), 0–20 m. **DUN:** 464, F10415; **EA:** 440, S39204; **EX:** 432, S39063; **GB:** 868, S36495; 874, S36684, S36705 (sub *Mycoblastus sanguinarius*); S Sandy Cove, S40738, S40739; **GUS:** 107, F10033; 857, S36110; 862, S36296; 316, S37871; 341, S38232; 435, S39121; 531, T41522; 534, T41569; Tower Rd, S40736. TLC: fpc. This species combines anatomical features of both *Pertusaria* and *Lepra* and its generic placement requires further study.
*Pertusaria subobducens* Nyl. — Terricolous on rock outcrops, 895–922 m. **EX:** 370, S38336, S38348; 407, S38831, S38860 (sub *Biatora cuprea*). TLC: 2,7-dichlorolichexanthone, stictic acid.
*Pertusaria suboculata* Brodo & Dibben — Corticolous on *Cassiope* sp., *Cupressus nootkatensis*, *Elliotia pyroliflora*, *Loiseleuria procumbens*, *Menziesia ferruginea*, *Tsuga mertensiana* and *Vaccinium ovalifolium*, also terricolous/muscicolous on coarse organic matter, 0–922 m, in frost pockets. **DUN:** 116, F10147; 121, F10181; 337, S38117; 412, S38913; 416, S38941; 425, S38986; **EX:** [858, S36174]; 376, S38429; 407, F10127; 454, F10341. TLC: fpc. A regional endemic and characteristic species of woody stems of *Ericaceae*, especially on upper mountain slopes.
+*Phaeocalicium compressulum* (Szatala) A. F. W. Schmidt — Corticolous on *Alnus* twigs, 0–11 m. **DUN:** 467, S39640; **GUS:** 857, S36095.
*+*Phaeocalicium interruptum* (Nyl.) Tibell — Corticolous on *Salix* branches at the back of beach meadows, 5 m. **EA:** terminus of Riggs Glacier, S40794; **GUS:** 857, S36146 (conf. L. Tibell, 2012); **WA:** 318, S37900 (as ‘cf.’). Recently reported as new to North America by Hardman *et al.* (2017).
+*Phaeocalicium populneum* (Brond. ex Duby) A. F. W. Schmidt — Corticolous on *Populus balsamifera* on raised beach ridge, 7 m. **WA:** 395, S38656, S38677 (sub *Scoliciosporum chlorococcum*).
*Phaeophyscia ciliata* (Hoffm.) Moberg — Corticolous on *Populus balsamifera*, upper beach, 7 m. Also reported by Geiser *et al.* (1994) from Glacier Bay but the voucher has not been seen by us. **WA:** 395, S38664.
*Phaeophyscia decolor* (Kashiw.) Essl. — Saxicolous on alkaline argillite, alpine heath with rock outcrops, 830 m. **EX:** 404, F10066.
*Phaeophyscia orbicularis* (Neck.) Moberg — Saxicolous or muscicolous over marble above high tide, 0–22 m. **GB:** 864, S36379; 867, S36450; S Sandy Cove, S40761; **WA:** 318, S37907. Apparently localized on sea-level limestone in the lower West Arm and west part of Glacier Bay proper (Marble Mtn, Oystercatcher Cove and Gloomy Knob).
#*Phaeospora parasitica* (Lönnr.) Arnold — Lichenicolous on *Biatora alaskana* over *Picea* trunk, 2 m. **GUS:** 857, S36098; 341, S38240. Previously reported in Alaska from Cape Krusenstern, on *Rhizocarpon* (Zhurbenko 2009).
*Phlyctis argena* (Spreng.) Flot. — Corticolous on *Alnus* and *Populus balsamifera*, 0–10 m. **EA:** 441, S39264, S39284; **GB:** N Sandy Cove, S36640; 868, S36464; **GUS:** 341, S38238 (fertile and ± esorediate = *P. speirea* G. Merr.). TLC: norstictic acid. Unusually uncommon in GLBA compared to other places in NW North America. Current data suggest it is restricted to the NE corner of Glacier Bay proper (Puffin Island, Seebree Island and Muir Point). DNA from S36464 was published by Resl *et al.* (2015). We interpret a phylogenetic analysis by Muscavitch *et al.* (2017) to suggest that *P. argena* and *P. speirea* are two thallus morphologies involving the same fungal species.
*#*Phoma physciicola* Keissl. — Lichenicolous on *Physcia alnophila*, corticolous on *Populus balsamifera*, upper beach, 7 m. **WA:** 395, S38671.
*Phylliscum demangeonii* (Moug. & Mont.) Nyl. — Saxicolous on soft argillite, 922 m. **EX:** 407, S38837 (sub *Lecanora intricata*).
*Physcia adscendens* H. Olivier — Corticolous on dead *Alnus* twigs below eagle perch, 2–4 m. **GB:** 864, S36355.
*Physcia alnophila* (Vain.) Loht. *et al.* — Corticolous on *Populus balsamifera* and *Salix*, upper beach or rocks above water line, 7–10 m. **EA:** 872, S36617; terminus of Riggs Glacier, S40788; **WA:** 395, S38657, S38670, S38671 (sub *Phoma physciicola*), S38683. *Physcia aipolia* f. *aipolia* was reported by Thomson (1963) from Muir Glacier but it is not clear where that specimen is and how it would be interpreted under the current taxonomy.
*Physcia caesia* (Hoffm.) Fürnr. — Saxicolous on large stable boulders in the upper intertidal, on rocky headlands, and on alpine argillite, also once corticolous on *Alnus* in beach fringe, 0–880 m. **DUN:** 463, S39578, F10410 (sub *Adelolecia kolaënsis* ); **EX:** 459, F10354; **GB:** 864, S36351; **GUS:** seen and photographed, for example, at docks at Park HQ; **WA:** Queen Inlet [photograph]. This species is far more common than the four collections would indicate and is found in all metamorphic intertidal areas of GLBA on large, stationary rocks.
*Physcia dubia* (Hoffm.) Lettau — Saxicolous on bird rocks, 115–125 m. **EA:** between 870 and 871, S36588; **GB:** Willoughby Island, S36322; **WA:** 388, S38555. Also reported from Muir Glacier by Thomson (1963).
*Physcia phaea* (Tuck.) J. W. Thomson — Saxicolous at the high tide line, 0–1 m. **WA:** 322, S37964.
*Physcia tenella* (Scop.) DC. — Corticolous on *Alnus* and *Picea sitchensis* twigs, often where there is local enrichment (e.g. below bald eagle perches), also once saxicolous, 0–27 m. **DUN:** 339, S38175; 463, S39565; **GB:** 864, S36349; Willoughby Island, S36319; Marble Mtn beach, S38005.
*Physconia* cf. *americana* Essl. — Corticolous on *Populus balsamifera*, 0–2 m. **GB:** 867, S36430. A poor specimen, from Oystercatcher Cove, was the only one of this otherwise common genus found during the current survey.
*Pilophorus acicularis* (Ach.) Nyl. — Saxicolous on forest floor boulder, 20–165 m. **DUN:** 335, S38111; 220, M2673; **GUS:** 142, F10495. In addition to occurring at a site in Fern Harbor, *P. acicularis* also occurs on erratics near the Glacier Bay Lodge in Bartlett Cove (not collected). The species appears to be uncommon in GLBA.
*Pilophorus clavatus* Th. Fr. — Saxicolous on boulder in forest, 149 m. **DUN:** 416, S38943.
*Pilophorus dovrensis* (Nyl.) Timdal *et al*. — Saxicolous on pebbles and sheltered rock, 10–918 m. **EA:** 870, S36575; **EX:** 454, F10340, S39383 (sub *Steineropsis alaskana*); S39401; 218, M2647; **WA:** 319, S37939; 384, S38521-B. TLC: isousnic acid. An arctic-alpine species, descending to sea level near glaciers.
*Pilophorus nigricaulis* Satô — Saxicolous on seepy rock faces or in areas with late-lying snow, also on erratics, 74–435 m. **DUN:** 120, F10160; 414, F10142, S38933 (as ‘aff.)’; 415, S38936 (as ‘aff.’); 422, S38965; 423, S38974; 222, M2689. TLC: atranorin, stictic, menegazziaic acids, ±zeorin. All the locations of this species are in the Dundas Basin, where it is not rare. S38974 is one of few known *Pilophorus* specimens to contain zeorin.
*Placopsis cribellans* (Nyl.) Räsänen — Saxicolous on granite and gneiss, 10–115 m. **EA:** 870, S36573 (sub *Tingiopsidium elaeinum*); upper Muir Inlet, S40764; **EX:** 454, S39420 (sub *Ionaspis ventosa*); **GUS:** Bartlett Cove at docks, *Fryday* s. n., 4 September 2012 (MSC); **WA:** 101, F9915; 381, S38448 (sub *Placynthium* sp.), S38452. TLC: gyrophoric, 5-*O*-methylhiascic acids. The Bartlett Cove specimen is the basis of DNA isolate T1074 published by Schneider *et al.* (2016).
*Placopsis fusciduloides* D. J. Galloway — Saxicolous on rocks and pebbles on high beaches, 2–918 m. **EA:** 869, F9765; 870, S36574; 872, S36625; **EX:** 454, S39399; **WA:** 391, S38635. TLC: gyrophoric, 5-*O*-methylhiascic acids. S38635 is the basis of DNA isolate KS101 published by Schneider *et al.* (2016).
*Placopsis gelida* (L.) Hoffm. s. str. — Saxicolous in recently deglaciated area (<30 yr), near sea level. **EA:** Muir Glacier, *Fryday* s. n., September 2014 (GZU). *Placopsis gelida* is widely reported for Alaska, but specimens corresponding to the Scandinavian species are uncommon in our area. The Muir Glacier specimen was the only one from GLBA that matched European material morphologically and genetically. It was the basis for the DNA isolate KS139 with six sequenced loci (ITS: KU844738) in the study of Schneider *et al.* (2016). Note that *Placopsis gelida* as used here is the same as ‘*Placopsis sulcata* T. Sprib. *ined*.’ of McCune *et al.* (2018). The systematics of *Placopsis*, including the typification of *P. gelida*, will be discussed in greater detail elsewhere.
*Placynthiella dasaea* (Stirt.) Tønsberg — Lignicolous on conifer stumps in advanced decay, 35–40 m. **EX:** [125, F10252, F10269]; **GUS:** 316, S37863.
*Placynthiella icmalea* (Ach.) Coppins & P. James — Lignicolous on stumps, muscicolous on dangling soft wood and moss, and corticolous once on *Pinus contorta*, 0–245 m. **EX:** [859, S36234 (sub *Cladonia umbricola*)]; 400, S38734; **GUS:** 107, F10037.
*Placynthiella uliginosa* (Schrad.) Coppins & P. James — Muscicolous on log in raised beach meadow, 2–4 m. **GUS:** 437, S39166 (sub *Epigloea urosperma*).
*Placynthium asperellum* (Ach.) Trevis. — Saxicolous on schistose and argillitic rocks, from beach ridges to alpine zone, 0–942 m. **EX:** 405, F10084 (narrow-lobed, trailing form); 408, S38863 (sub *Lecanora* sp. F10122); 409, S38885; 454, S39395; **GB:** 868, S36546, F9751; **WA:** 105, F9982 (as ‘juv.’); 392, S38651 (narrow-lobed, trailing form). Specimen S38885 is the source of DNA isolate T1306 (Fig. 9).
*Placynthium flabellosum* (Tuck.) Zahlbr. — Saxicolous at lake edge, 127 m. **DUN:** 419, S38956. We extracted and sequenced DNA from this specimen (isolate T1350, Fig. 9).
*Placynthium glaciale* Fryday & T. Sprib. — See ‘Descriptions of New Genera and Species’.
*Placynthium subradiatum (Nyl.) Arnold — Saxicolous on exposed limestone bedrock, 52 m. **WA:** 382, 38476. The specimen is the basis for DNA isolate T1309 (Fig. 9).
*Placynthium* aff. *tantaleum* (Hepp) Hue — On limestone, 5 m. **GB:** 864, S36386. This species is similar in morphology to *P. nigrum* but appressed and with a much more strongly developed blue-green hypothallus. Sequences from the GLBA specimen (isolate T1308) resolve as closely related to a sequence of *P. tantaleum* from Montana, USA (isolate T1183), with high support (Fig. 9). However, the species delimitations in this group are necessarily tentative pending a more comprehensive study.
*Platismatia glauca* (L.) W. L. Culb. & C. F. Culb. — Corticolous on *Alnus*, *Picea sitchensis*, *Pinus contorta* and *Tsuga heterophylla*, 0–922 m. **DUN:** 339, S38161; 462, S39731; 464, F10420, S39614; **EA:** 441, F10210, S39277; **EX:** 407, S38815; **GUS:** 100, F9892; 107, F10022; 855, S36008, S36021; 862, S36290; 397, S38703; **WA:** Blue Mouse Cove plot BM1a, BM2a (GLBA herbarium).
*Platismatia herrei* (Imsh.) W. L. Culb. & C. F. Culb. — Corticolous on *Picea sitchensis*, *Pinus contorta* and *Tsuga heterophylla*, 10–32 m. **DUN:** 131, F10447; 339, S38178; 464, S39613; **GB:** 868, S36518; **GUS:** [861, F9672]; 862, S36304; 881, S36836; 434, S39105; 573, T41809; no waypoint, M2482. TLC: atr, caperatic acid.
*Platismatia lacunosa* (Ach.) W. L. Culb. & C. F. Culb. — Corticolous on *Alnus* (1×) and *Pinus contorta* (4×) from 0–500 m, also saxicolous on alpine rocks, 895–903 m. **DUN:** 337, S38122; 462, S39733; 464, F10414 (fertile), S39616; **EX:** 109, F10048; 112, F10062; 370, S38361; 455, S39422; **GB:** 868, S36476. The alpine specimens are similar in morphology to the corticolous material but have a different ecology and biogeography, extending far westwards on the Aleutian Island chain (Krog 1968).
*Platismatia norvegica* (Lynge) W. L. Culb. & C. F. Culb. — Corticolous on *Alnus* (3×), *Picea sitchensis* (5×), *Pinus contorta* (1×) and *Tsuga heterophylla* (1×), 0–50 m. **DUN:** 339, S38183; 463, S39518; **EA:** 441, S39268; **EX:** [858, S36168]; **GB:** 864, S36345; 868, S36501, S36520; **GUS:** 107, F10025; 856, F9620; 857, S36111; 435, S39126; **WA:** Blue Mouse Cove plot BM1a, BM2a (GLBA herbarium). TLC: atr, caperatic acid.
#*Plectocarpon lichenum* (Sommerf.) D. Hawksw. — Lichenicolous on *Lobaria anomala* and *L. pulmonaria* on *Picea* branches, 12–33 m. **GB:** 868, S36532 (sub *Lobaria anomala*); **GUS:** [V329, S38043 (sub *Lobaria anomala*)]; 397, S38712; 436, S39164.
#*Plectocarpon nephromeum* (Norman) R. Sant. — Lichenicolous on *Nephroma* spp. (*N. bellum*, *N. resupinatum*), corticolous on *Alnus* and *Picea sitchensis*, 0–20 m. **EA:** no waypoint, M2823a; **GB:** 868, S36460 (sub *Nephroma resupinatum*), S36511; **GUS:** 434, S39111.
*Polyblastia albida Arnold — Saxicolous on limestone rocks on shore, 0–5 m. **GB:** 863, F9692, F9700; 864, F9722, F9726, F9727, F9731. This species has been found so far only on Willoughby Island and the adjacent mainland on Marble Mtn.
**Polyblastia efflorescens Coppins — Saxicolous on shaded limestone crags behind beach, 10–15 m. **WA:** 105, F10012, F10013. Distinguished from all other species of the genus (and many pyrenocarpous lichens) by the thallus dissolving into effuse green soralia. Until now known only from the British Isles (Gilbert & Coppins 1992).
*Polyblastia exalbida* (Nyl.) Zahlbr. — Saxicolous on limestone crags, 0–20 m. **WA:** 104, F9975.
*Polyblastia fulva* Zahlbr. — Saxicolous in rock crevices, 920 m. **EX:** 407, S38834; 454, F10345, S39385; 218, M2659a.
*Polycauliona pollinarioides (L. Lindblom & D. M. Wright) Frödén *et al*. — Corticolous on twigs of *Alnus* and *Picea sitchensis*, often near eagle perches, also lignicolous on stabilized driftwood; outside of GLBA also observed as saxicolous on supralittoral rocks, 0–27 m. **DUN:** 339, S38169; 467, S39652, S39653; 573, T41808; **GB:** 864, S36354; 873, S36657; Willoughby Island, S36319 (sub *Physcia tenella*), S36320; **GUS:** 435, S39120. We have sequenced two specimens for ITS rDNA that closely match *P. pollinarioides*, one from supralittoral rocks on Mitkof Island (isolate T1332; outside of GLBA) and the other from Albert Head, British Columbia (KS91), both published here (Table 1). We take a broad view of *P. pollinarioides*, including *P. kaernefeltii* in its circumscription. We note that the DNA sequences of *P. pollinarioides* from California (KC179388) and *P. kaernefeltii* from Chile (KC179385) published by Arup *et al.* (2013) are nearly identical to each other and highly similar to those we have obtained in coastal western North America (T1332 and KS91).
*Polycauliona polycarpa* (Hoffm.) Frödén *et al*. — Corticolous on dead *Alnus* twigs, below eagle perch, 4 m. This species was also reported from Glacier Bay by Rudolph (1955, as *Xanthoria polycarpa*). **GB:** 864, S36357 (conf. L. Lindblom, as *Xanthoria polycarpa* s. lat.); **WA:** 322, S37965. The affinity of S37965 to *P. polycarpa* was confirmed by ITS rDNA (Table 1).
*Polychidium muscicola* (Sw.) Gray — Terricolous on fine organic accumulations over rock or embedded in moss cushions, also terricolous on mineral soil in recent glacial forelands (*Dryas* mat stage), 10–942 m. **EA:** 870, S36570 (sub *Massalongia carnosa*), S36572; 872, F9806; terminus of Riggs Glacier, S40798; **EX:** 406, S38795; 408, S38866; 216, M2581; **WA:** 101, F9902; 103, F9954; 319, S37948; 383, S38497 (sub *Cladonia chlorophaea*); 384, S38524; 549, T41632.
#*Polycoccum deformans* R. Sant. & Brackel — Lichenicolous on *Placopsis* sp. in the supralittoral zone, sea level. **DUN:** 586, P2163. This species was previously reported as an associate of *Pyrenidium hyalosporum* Alstrup *et al*. from an Alaskan specimen by Grube & Hafellner (1990) but it was only recently formally described (Brackel & Berger 2010). It has been overlooked in subsequent North American checklists. In addition to Alaska, it is known from Iceland, Europe and Australia (Brackel & Berger 2010).
#*Polycoccum hymeniicola* (Berk. & Broome) Zhurb. — Lichenicolous on *Lobaria linita* on *Tsuga* trunk, 3–343 m. **EX:** 349, P2343, P2348; **GB:** 874, F9830 (sub *Lobaria tenuior*); **GUS:** 877, S36759; [558, T41722]. According to ongoing studies (V. Atienza, D.L. Hawksworth and S. Pérez-Ortega, unpublished data) *P. hymeniicola* belongs in neither *Polycoccum* nor *Endococcus*, where it had been previously placed by Hawksworth (2005).
*#*Polycoccum pulvinatum* (Eitner) R. Sant. — Lichenicolous on *Physcia* cf. *caesia*, saxicolous on rocky headlands and beaches, 0–9 m. **DUN:** 463, S39576, S39582; **GB:** 873, S36659.
*Polysporina lapponica (Ach. ex Schaer.) Degel. — Saxicolous on upper beach, 6 m. **WA:** 104, F9965 (sub *Porpidia* cf. *thomsonii*); 389, S38577 (det. M. Westberg, 2013).
*Polysporina urceolata (Anzi) Brodo — Saxicolous on soft argillite on alpine ridge, 918 m. **EX:** 454, S39409 (sub *Lecidella patavina*); 459, F10360 (sub *Candelariella* sp.).
*Porina chlorotica (Ach.) Müll. Arg. — Saxicolous on alkaline argillite in alpine heath, 918 m. **EX:** 454, F10349 (sub *Protoblastenia rupestris*).
*Porina leptalea* (Durieu & Mont.) A. L. Sm. — Corticolous on *Picea sitchensis* and *Tsuga heterophylla*, also lignicolous and once fungicolous on polypore, 0–569 m. **EX:** 358, S38310; 444, S39327 (sub *Micarea botryoides*); **GUS:** 204, S38434; 876, S36731; 341, S38268.
*Porina pacifica* Brodo — Saxicolous on deeply shaded vertical wall in forest, also once on rocks under tip-up, 9–27 m. **DUN:** 339, S38201; 463, S39594; 572, T41782, T41785; 573, T41797.
*Porpidia carlottiana* Gowan — Saxicolous on deeply shaded vertical rock-face in forest, 27 m. **DUN:** 339, S38214.
*Porpidia crustulata* (Ach.) Hertel & Knoph — Saxicolous on pebbles in glacial outwash plain and on raised beaches, 4–8 m. **EA:** 438, F10226 (as ‘cf.’); **WA:** 324, S37980.
*Porpidia flavicunda* (Ach.) Gowan — Saxicolous on exposed rocks jutting out from alpine heath and sod, 618–936 m. **DUN:** 428, S39007; **EX:** 375, S38424; 405, F10103; 407, F10111.
**Porpidia irrigua Orange — Saxicolous on sedimentary and metamorphic rock from raised beaches to erratics in muskegs and exposed alpine rocks, 0–922 m. **DUN:** 116, F10145; 426, S38994; **EA:** 870, F9787; 872, F9802; N shore Muir Inlet, F9797; **EX:** 407, F10120. It has been suspected for some time that there were two entities in *Porpidia* that contained methyl 2′-*O*-methylmicrophyllinate and were combined under the name *P. contraponenda* (Arnold) Knoph & Hertel (Fryday 2001). Orange (2014) showed that *P. contraponenda* s. str. was a species with innate apothecia and introduced the name *P. irrigua* for specimens with sessile apothecia. Several collections from NW North America have been shown to match *P. irrigua* morphologically and chemically, although molecular data is necessary to confirm their identity. The description of *P. contraponenda* given by Gowan (1989) clearly refers to *P. irrigua* and we have examined selected material from several North American herbaria (CANL, F, MSC, MIN, WIS) and no material has been found to match *P. contraponenda* in the strict sense. It is probable that *P. contraponenda* has not been correctly reported from North America.
*Porpidia macrocarpa* (DC.) Hertel & A. J. Schwab, aggr. — Saxicolous, 903 m. **EX:** 406, S38782 (sub *Lecidea swartzioidea*).
*Porpidia nigrocruenta (Anzi) Diederich & Sérus. — Saxicolous on granitic boulders in lowland woodland and shorelines and once on an alpine ridge, 0–918 m. **EX:** 454, F10347; **GUS:** 142, F10494; **WA:** 102, F9936; 319, S37934. This taxon, which is characterized by the presence of a KOH+ magenta pigment in the exciple, has been treated at various taxonomic ranks, from being included in the synonymy of *P. macrocarpa* (Gowan 1989) through forma (Fryday 2005) to species (Hertel 1973; Diederich *et al.* 1988). We consider that it warrants taxonomic recognition and, because species boundaries in the *P. macrocarpa* group are currently unclear, we prefer to recognize it at the species level.
*Porpidia seakensis* Fryday — See ‘Descriptions of New Genera and Species’.
*Porpidia soredizodes (Lamy ex Nyl.) J. R. Laundon — Saxicolous on boulder in deep shade, 2–4 m. **EA:** 872, F9802 (sub *Porpidia irrigua*); **GB:** 865, S36416. TLC: stictic acid + high-running unidentified substance.
*Porpidia speirea* (Ach.) Kremp. — Saxicolous on sheltered underhangs of rocks jutting out from alpine tundra, 878–903 m. **EX:** 406, S38786 (sub *Pertusaria* sp. S38786); 457, S39482.
**Porpidia striata Fryday — Saxicolous on rock in stream through muskeg, 25 m. **DUN:** 465, F10427 (sub *Rhizocarpon hochstetteri*). This species was described from Scotland and has since been reported from various European countries and Russia (Zhdanov 2012).
*Porpidia superba* (Körb.) Hertel & Knoph — Saxicolous on pebbles on raised beaches, dry to semi-inundated granitic and argillitic rocks from sea level to alpine zone, 0–918 m. **EX:** 128, F10299, F10300, F10301; 454, F10335, F10336, F10349 (sub *Protoblastenia rupestris*); **WA:** 101, F9910, F9919; 103, F9955; 105, F9978, F9998, F10014; 326, S37994; 381, S38445 (sub *Rhizocarpon geminatum*), S38447 (sub *Rhizocarpon geminatum*), S38453; 391, S38627.
*Porpidia* cf. *thomsonii* Gowan — Saxicolous on pebbles on raised beaches and granitic rock outcrops, 0–460 m. **DUN:** 120, F10167; 121, F10174; **WA:** 101, F9924; 104, F9963, F9964, F9965; 105, F9981, F9985, F9986, F10001, F10002. Gowan (1989) introduced the name *P. thomsonii* for collections from arctic North America that were intermediate between *P. crustulata* and *P. macrocarpa.* The collections from GLBA agree with that description but it is unclear if they are conspecific with the type specimen of *P. thomsonii*.
#*Pronectria fissuriprodiens* Etayo — Lichenicolous on *Lobaria pulmonaria*, epiphytic, 22 m. **GUS:** A569, P2370.
*Protoblastenia incrustans (DC.) J. Steiner — Saxicolous, including on argillite on alpine ridge, 21–918 m. **EX:** 454, S39390, S39393; **WA:** 320, S37955 (as ‘cf.’); no waypoint, M2533.
*Protoblastenia rupestris* (Scop.) J. Steiner — Saxicolous on sedimentary rocks, 0–918 m. **EA:** N shore of Muir Inlet, F9796; **EX:** 128, F10291; 454, F10349; 455, S39438.
**Protoblastenia siebenhaariana (Körb.) J. Steiner — Saxicolous on calcareous rock, 20–43 m. **WA:** 319, S37936; 392, S38644, S38652. This species has been reported from northern Europe as well as European Russia and Arctic Siberia (Urbanavichus & Andreev 2010).
*Protomicarea limosa* (Ach.) Hafellner — Terricolous on fine organic accumulations over granitic rock, including where seepy and on undersides and vertical faces, 68–618 m. **DUN:** 119, F10155; 120, F10163; 121, F10180; 413, S38931; 422, S38963; 427, S38998; 428, S39026; 219, M2680; 222, M2697. All the collections of this species are from the Dundas Basin.
*Protopannaria pezizoides* (Weber) P. M. Jørg. & S. Ekman — Terricolous on fine organic accumulations over rock, terricolous on recently deglaciated soils, on moss and once corticolous on *Populus balsamifera*, 0–913 m. **DUN:** 463, S39595; **EA:** 869, S36564, S36565 (sub *Cladonia rei*); **EX:** 454, S39413; **GB:** 864, S36371; **GUS:** 855, S36058; 878, S36776; **WA:** 103, F9957; 383, S38497 (sub *Cladonia chlorophaea*); 387, S38540; 543, T41600; 545, T41622.
*Protoparmelia badia* (Hoffm.) Hafellner — Saxicolous on argillite, 618–922 m. **DUN:** 428, S39016 (sub *Rhizocarpon intersitum*); **EX:** 372, S38368; 407, S38825.
*Protoparmelia ochrococca* (Nyl.) P. M. Jørg. — Corticolous on the upper side of a leaning *Pinus contorta* trunk in muskeg, 233 m. **EX:** 314, S37850 (sub *Imshaugia aleurites*).
*Protoparmeliopsis muralis* (Schreb.) M. Choisy — Saxicolous on argillite rock outcrops, especially bird perches, 880–922 m. **EX:** 407, F10131; 455, S39436; 459, F10351; 218, M2668.
*Protothelenella corrosa (Körb.) H. Mayrhofer & Poelt — Saxicolous on alkaline argillite in alpine heath, 918 m. **EX:** 454, F10331 (sub *Amygdalaria pelobotryon*).
*Protothelenella sphinctrinoidella* (Nyl.) H. Mayrhofer & Poelt — Terricolous on fine organic accumulations and over hepatics, also lignicolous, 918 m. **EX:** 454, S39418 (conf. H. Mayrhofer); 213, M2570.
*Pseudephebe pubescens* (L.) M. Choisy — Saxicolous on granite and other siliceous rocks, 10–618 m. Also reported from Muir Glacier by Degelius (1937). **DUN:** 428, S39003; **EA:** just S of Riggs Glacier terminus, F10653; **GB:** Muir Glacier, *Kincaid* (UPS, det. Degelius, conf. A. Nordin 2015); **WA:** 384, S38518.
*Pseudocyphellaria citrina* (Pers.) McCune *et al*. — Corticolous on *Alnus*, *Picea sitchensis*, *Pinus contorta* and *Salix*, associated with beach fringe, 3–33 m. **EA:** 440, S39215* (also GenBank no. MF537296); **GB:** 868, S36460 (sub *Nephroma resupinatum*); 874, S36689*; **GUS:** 862, S36288*; [V329, S38017, S38042]; [557, T41703]; 576, T41832; **WA:** Blue Mouse Cove plot BM1a, BM2a (GLBA herbarium). This species and *P. hawaiiensis* were until recently referred to *P. crocata*, but Lücking *et al.* (2017) have shown that species is not found in North or South America. Only the specimens marked with an asterisk* above were seen in that study, as well as the two specimens of *P. hawaiiensis* (below); other material is tentatively placed here but has not been reassessed.
*Pseudocyphellaria hawaiiensis* H. Magn. — Corticolous on *Salix* and *Picea*, sea level. **GUS:** 857, S36144, S36162. Both specimens are cited by Lücking *et al.* (2017). See note under *P. citrina*.
*Pseudocyphellaria mallota* (Tuck.) H. Magn. s. lat. — Corticolous on *Tsuga heterophylla* twig, edge of muskeg, 234 m. **EX:** 349, 38284. Only one small thallus found.
*Psilolechia clavulifera (Nyl.) Coppins — Saxicolous on sheltered rock in mixed woodland, 10 m. **GUS:** 875, F9848.
*Psilolechia lucida* (Ach.) M. Choisy — Saxicolous on rock in cavity at base of stump, in forest, 80 m. **DUN:** 561, T41739.
*Psora globifera* (Ach.) A. Massal. — Terricolous on open ground, 670 m. **GB:** E slopes of Marble Mtn opposite Drake Island, 9 August 1968, *I. A. Worley* 11216 (UBC, det. C. R. Björk).
*Psora nipponica* (Zahlbr.) G. Schneider — Terricolous on fine soil accumulations over argillite, 922 m. **EX:** 407, F10106, S38856. TLC: gyrophoric acid.
*Psora rubiformis* (Ach.) Hook. — Terricolous on fine soil accumulations over argillite, 883–922 m. **EX:** 370, S38362; 405, F10090; 407, F10124, F10125 (sub *Caloplaca tiroliensis*).
*Psoroglaena biatorella* (Arnold) Lücking — Saxicolous on limestone crags, 0–20 m. **WA:** 104, F9974.
*Psoroma hypnorum* (Vahl) Gray, s. lat. — Terricolous on fine soil accumulations and muscicolous over rock, also corticolous on *Alnus* and *Picea sitchensis*, 0-903 m. **DUN:** 463, S39550; **EA:** 870, S36571; between 870 and 871, S36586; 438, S39184; nr 438, S39296; vic. of Nunatak Knob, E shore of Muir Inlet (S of McBride Glacier), *Worley* 10335 (UBC L29687); **EX:** 406, S38788 (sub *Lopadium pezizoideum*); 217, M2608; 455, S39446; **GB:** 874, S36688; Tower Rd, S38273; State Dock, F9874; **GUS:** 232, M2771; semi-open sand rise along road from Bartlett Cove to Gustavus, *Worley* 10986, *Boas & Streveler* (UBC L29703; ascospores not measured); **WA:** 325, S37984; 383, S38502. An rDNA ITS sequence from an epiphytic specimen (S39296, isolate T1347, Table 1) closely matches material sequenced by Elvebakk *et al.* (2010) from Scandinavia and labelled *P. hypnorum*. Some variability in ascospore length and width was observed and may deserve more study.
*Psoroma tenue* Henssen var. *boreale* Henssen — Terricolous, 670 m. **GB:** E slopes of Marble Mtn opposite Drake Island, 9 Aug. 1968, *I. A. Worley* 11210 (UBC, det. C. R. Björk, not seen by us). Several other collections (**GB:** 864, F9715; **EA:** 970, F9762, F9771; **DUN:** 463, F10400, F10401) have the morphology of this taxon, but TLC of one specimen (F9762) failed to reveal any substances.
*Ptychographa* aff. *xylographoides* Nyl. — Lignicolous on *Pinus contorta*, 12 m. **GUS:** 397, S38708. Alaskan specimens we have seen (including from near Juneau) have smaller and more broadly elliptical ascospores than those of Scottish (typical) specimens. However, all the Alaskan specimens seen to date are too scant to use as the basis for a detailed analysis.
*Puttea caesia* (Th. Fr.) M. Svensson & T. Sprib. — Lignicolous on *Cupressus nootkatensis*. **DUN:** 219, M2679.
*Puttea exsequens (Nyl.) Printzen & Davydov — Lignicolous on exposed wood below high-water line, 155 m. **EX:** 444, S39320; 236, M2826. This may be the same species as *Biatora* sp. S24848 from KLGO (Spribille *et al.* 2010).
*Puttea margaritella* (Hulting) S. Stenroos & Huhtinen — Hepaticolous on *Ptilidium* at the base of snags and on logs, 21–250 m. **EX:** 109, F10049; 312, S37821; 433, S39090.
#*Pyrenidium actinellum* Nyl. — Lichenicolous on *Arctomia delicatula*, on *Lobaria oregana*, corticolous/muscicolous on fallen branch, and on *Protopannaria pezizoides*, 22–50 m. **EX:** [858, S36205]. **GUS:** A578, P2340; **WA:** A571, P2097-bis, P2417.
*Pyrenopsis furfurea* (Nyl.) Leight. — Saxicolous on argillite in alpine heath, 918 m. **EX:** 454, F10331 (det. M. Schultz; sub *Amygdalaria pelobotryon*).
*Pyrenopsis phaeococca (Tuck.) Tuck. — Saxicolous on pebbles on raised beach, also on argillite in alpine heath, 5–918 m. **EX:** 454, F10346; **WA:** 105, F9988. This North American name, based on a description of material from North Carolina and New Hampshire (Tuckerman 1872: 80), is adopted for this material since European names do not seem to match well (M. Schultz, personal communication).
*Pyrenopsis* cf. *reducta* Th. Fr. — Saxicolous on metamorphic rock on raised beach, 0–5 m. **EA:** N shore of Muir Inlet, F9799. Ascospores 8 per ascus, broadly ellipsoid, 7.5–10.5 × 4.5–5.5 μm and hymenium I+ blue. This species may be close or even identical to *P. sanguinea* Anzi, a name not used in Nordic literature; the determination remains somewhat uncertain (M. Schultz, personal communication).
#*Raciborskiomyces peltigericola* (D. Hawksw.) M. E. Barr — Lichenicolous on *Peltigera* sp., 233 m. **EX:** 567, P2140.
*Racodium rupestre Pers. — Corticolous on *Alnus* and trunk of *Populus balsamifera*, 4–16 m. **GB:** 556, T41688; **GUS:** 533, T41563.
*Ramalina farinacea* (L.) Ach. — Corticolous on *Alnus*, *Picea sitchensis* and *Tsuga heterophylla*, always in beach fringe, 0–27 m. **DUN:** 339, S38177; 463, S39511, S39535; 573, T41807; **EA:** near 438, S39310; **GB:** 868, S36488; N Sandy Cove, S36648 (sub *Ramalina roesleri*); **GUS:** 857, S36134; 435, S39127.
*Ramalina* cf. *obtusata* (Arnold) Bitter — Corticolous on *Picea sitchensis* twigs on headland, 10 m. **DUN:** 467, S39655 (sub *Ramalina roesleri*). A few small thalli mixed in among *R. roesleri*.
*Ramalina roesleri* (Hochst. ex Schaer.) Hue — Corticolous on *Alnus* (1×), *Picea sitchensis* (7×) and *Tsuga heterophylla* (2×), associated with beach fringe, 0–27 m. **DUN:** 131, F10445; 339, S38170, S38186, S38193; 463, S39536; 467, F10436, S39642, S39655; 572, T41783; 573, T41811; **GB:** 874, S36690; 868, S36491; N Sandy Cove, S36648; **GUS:** 435, S39122; 436, S39160 (fertile).
*Ramalina thrausta* (Ach.) Nyl. — Corticolous, specific substratum and elevation unknown. **GB:** N shore of Beartrack Cove, *Derr* 4254 (GLBA herbarium).
*Ramboldia cinnabarina* (Sommerf.) Kalb *et al*. — Corticolous on *Pinus contorta*, 68 m. **DUN:** 413, S38924.
*Ramboldia gowardiana* (T. Sprib. & M. Hauck) Kalb *et al*. — Corticolous on *Alnus* (1×), *Picea sitchensis* (1×) and *Pinus contorta* (3×), 0–32 m. **DUN:** 464, S39615; **EA:** 440, S39239; **GB:** 868, S36497; **GUS:** 862, S36293; 534, S41568; 210, M2559. Specimen S39239 is rather unusual in possessing abundant sterile nascent ascomata and occurring on alder bark.
*Ramboldia subcinnabarina* (Tønsberg) Kalb *et al*. — Corticolous on *Alnus*, 11 m. **EA:** 872, S36608. TLC: two fatty acids.
*Rhizocarpon anaperum (Vain.) Vain. — Saxicolous on pebbles and rocks in subalpine meadows, and recently deglaciated areas at sea level, 0–700 m. **EX:** 449, F10320; **WA:** 326, S37993 (sub *Rhizocarpon lecanorinum*). First correct report for Alaska; previous records were based on misidentifications (Ihlen & Fryday 2004).
*Rhizocarpon anseris* Lynge — Saxicolous on beach pebbles, 0–5 m. **WA:** 105, F9993 (sub *Verrucaria* sp.).
*Rhizocarpon badioatrum* (Flörke ex Spreng.) Th. Fr. — Saxicolous on cobbles and bedrock, 43–406 m. **DUN:** 423, S38975; **WA:** 319, S37940 (sub *Micarea* sp. F10320). Two entities have been recognized in *Rhizocarpon badioatrum* since Koerber (1855) but only at the rank of variety: var. *badioatrum* (syn. var. *rivulare* (Flotow) Th. Fr.) and var. *vulgare* (Körb.) Th. Fr. Timdal & Holtan-Hartwig (1988) considered them distinct species, differing in both thalline and apothecial morphology as well as chemically, but declined to formally recognize them pending further study of relevant type material. *Rhizocarpon badioatrum* var. *badioatrum* differs from var. *vulgare* in having usually darker brown, thicker, and more plane areolae with a more angular to crenulate margin; in the absence of diffractaic acid in the thallus; and in having a broader, more diffusely delimited, more reddish brown epithecium which is more strongly KOH+ red (Timdal & Holtan-Hartwig 1988). *Rhizocarpon badioatrum* var. *vulgare* is reported from GLBA as *Rhizocarpon* sp. S39392 (see ‘Known Unknowns’).
*Rhizocarpon caesium* Fryday — Saxicolous on semi-inundated rocks along creek, 219–225 m. **EX:** 310, S37802; 128, F10296, F10297.
*Rhizocarpon chioneum* (Norman) Th. Fr. — Saxicolous on alpine argillite, 918 m. **EX:** 454, F10343.
*Rhizocarpon eupetraeoides* (Nyl.) Blomb. & Forssell — Saxicolous on alpine rocks, 895–937 m. **EX:** 370, S38347; 409, S38880 (sub *Lecanora swartzii*).
*Rhizocarpon geminatum* Körb. — Saxicolous on pebbles and other shoreline rocks, 0–15 m. **GB:** 868, F9752; **WA:** 102, F9943 (sub *Rhizocarpon lecanorinum*); 381, S38445, S38447.
*Rhizocarpon geographicum* (L.) DC., aggr. — Saxicolous on boulders and bedrock, especially near summits, 406–936 m. **DUN:** 121, F10182; 423, S38975 (sub *Rhizocarpon badioatrum*), S38976; 428, S39008, S39009, S39017, S39021 (sub *Euopsis granatina*); **EX:** 373, S38383; 374, S38408; 375, S38422; 405, F10097 (sub *Melanelia hepatizon*); 407, S38832.
*Rhizocarpon* cf. *grande* (Flörke) Arnold — Saxicolous on supralittoral gneiss, 2–5 m. **DUN:** 462, F10373. Thallus of dispersed creamy areoles; ascospores initially 4–8 per ascus but only 1(–2) developing, muriform, pigmented blue-green (N+ red), 30–35 × 11–13 μm; epihymenium KOH+ purple-red; medulla C+ red, I−. The ascospores are too small and the thalline chemistry wrong for *R. disporum*. *Rhizocarpon grande* s. str. has an I+ medulla and 8 ascospores per ascus, plus the thallus is brown with grey pruina. Another specimen that matches this morphology is *Imshaug* 28792 (MSC) from Juneau Icefield on the SE Alaskan mainland.
*Rhizocarpon haidense* Brodo & Fryday — See ‘Descriptions of New Genera and Species’.
*Rhizocarpon hensseniae* Brodo — Saxicolous on boulders and bedrock in frost pocket areas and in cold muskeg up to alpine heaths, 75–883 m. **DUN:** 115, F10143; 117, F10148; 120, F10170, F10171; 415, S38938; 419, S38957; 422, S38962; 428, S39004; 222, M2693, M2698 (sub *Pilophorus* sp.); **EA:** 872, S36627; **EX:** 405, F10098.
*Rhizocarpon hochstetteri* (Körb.) Vain. — Saxicolous, on rocks in snowbeds and streams and outcrops, 20–465 m. **DUN:** 119, F10159; 120, F10165; 426, S38991 (sub *Carbonea vorticosa*); 465, F10427; **WA:** 103, F9951.
*Rhizocarpon inarense* (Vain.) Vain. — Saxicolous, 937 m. **EX:** 409, S38887.
*Rhizocarpon infernulum* (Nyl.) Lynge — Saxicolous, on pebbles on raised beaches and on vertical rock faces, 0–43 m. **DUN:** 462, S39505; **WA:** 104, F9962; 105, F9990 (sub *Staurothele septentrionalis*); 319, S37931, S37940 (as ‘cf.’; sub *Micarea* sp. F10320); 326, S37988.
*Rhizocarpon intersitum Arnold — Saxicolous on alpine ridges and mountain tops, 618–918 m. **DUN:** 428, S39016; **EX:** 454, S39398.
*Rhizocarpon jemtlandicum* (Malme) Malme — Saxicolous, 5 m. **EA:** just S of Riggs Glacier terminus, F10652.
*Rhizocarpon lavatum* (Ach.) Hazsl. — Saxicolous on alpine argillite, 830 m. **EX:** 404, F10070 (sub *Biatora subduplex*), F10075. Thallus I−; ascospores 35 × 15 μm, 8 per ascus; epithecium KOH+ purplish.
*Rhizocarpon lecanorinum* Anders — Saxicolous on pebbles, cobbles and exposed outcrops, especially on beach ridges, also on bird perches, 0–125 m. **GB:** N tip Willoughby Island, F9713; **WA:** 101, F9907, F9913; 102, F9937, F9943; 323, S37973; 326, S37993, S37989; 388, S38557, S38563.
*Rhizocarpon macrosporum* Räsänen — Saxicolous on exposed rocks, possibly associated with larger cobbles or bedrock, 10–125 m. **EA:** N shore of Muir Inlet, F9798 (sub *Staurothele septentrionalis*); **WA:** 101, F9927; 381, S38450; 383, S38493 (sub *Bellemerea subsorediza*); 387, S38545; 388, S38561 (sub *Candelariella vitellina*).
*Rhizocarpon oederi* (Ach.) Körb. — Saxicolous on metal-enriched boulders, 10–100 m. **EA:** 872, S36629; **WA:** 101, F9923. This is the first record of this species for Alaska since it was reported, without a locality, by Rothrock (1884, as *Buellia petraea* var. *oederi*). S36629 is the source of a DNA isolate (T1071, Figs 5 & 10).
*Rhizocarpon petraeum* (Wulfen) A. Massal. — Saxicolous on pebbles and larger granitic rocks, also semi-inundated rock along creek, 0–225 m. **EX:** 128, F10298; 406, S38787; **WA:** 101, F9911; 102, F9929; 105, F9980; 381, S38460.
*Rhizocarpon polycarpum* (Hepp) Th. Fr. — Saxicolous on granite and argillite in tarns, muskegs and tundra, 0–788 m. **DUN:** 118, F10154; **EX:** 460, F10361; **WA:** 101, F9916; 102, F9928, F9930, F9931, F9943 (sub *Rhizocarpon lecanorinum*); 381, S38445 (sub *Rhizocarpon geminatum*); 382, S38475; 388, S38558, S38561 (sub *Candelariella vitellina*).
*Rhizocarpon reductum* Th. Fr. — Saxicolous on pebbles and granitic and gneiss cobbles and boulders, associated with beaches and seashores, 0–25 m. **DUN:** 462, F10375; **GUS:** 141, F10492; **WA:** 326, S37993 (sub *Rhizocarpon lecanorinum*); 381, S38445 (sub *Rhizocarpon geminatum*).
*Rimularia limborina* Nyl. — Saxicolous on argillite in the alpine zone, 883–919 m. **DUN:** 223, M2701; **EX:** 374, S38406 (sub *Sagiolechia phaeospora*); 405, F10100; 409, S38878 (sub *Pyrenopsis* sp.). *Rimularia limborina* accounts for *Rimularia* sp. S24515 from KLGO (Spribille *et al.* 2010). A DNA isolate of *R. limborina* from F10100 was used by Spribille *et al.* (2014*b*) in a phylogenetic tree of *Baeomycetales*.
*Rinodina cinnamomea* (Th. Fr.) Räsänen — Terricolous on fine organic accumulations over argillite, 880 m. **EX:** 459, F10355 (sub *Rinodina turfacea*); 218, M2621. Specimen F10355 was first reported by Sheard (2018).
*Rinodina conradii* Körb. — Lignicolous on beach log above high tide line, 2–4 m. **WA:** 396, S38687.
*Rinodina disjuncta* Sheard & Tønsberg — Corticolous on *Picea sitchensis*, 5 m. **GB:** 868, S36489; **GUS:** 531, T41538; 576, T41831. TLC: sphaerophorin.
*Rinodina efflorescens* Malme — Corticolous on *Shepherdia canadensis* stalk, 4 m. **GB:** 867, S36435 (sub *Lecidella elaeochroma*). TLC: pannarin.
*Rinodina gennarii* Bagl. — Saxicolous on gneiss, supralittoral zone, sea level. **DUN:** 130, F10384, F10386 (det. J. Sheard). Previously reported by Geiser *et al.* (1998) for SE Alaska.
*Rinodina laevigata* (Ach.) Malme — Corticolous on *Populus balsamifera*, upper beach, 7 m. **GB:** 864, 36347; 867, S36433; **WA:** 395, S38658, S38665.
*Rinodina macrospora Sheard — Corticolous on *Alnus*, *Picea sitchensis* and *Tsuga heterophylla*, 0–27 m. **DUN:** 339, S38196; 573, T41804; **GB:** 868, S36515; Marble Mtn beach, S38004 (Spribille specimens det. H. Mayrhofer).
*Rinodina mniaroea* (Ach.) Körb. — a) typical chemotype (no substances): terricolous over fine organic accumulations or muscicolous over rock, 895–935 m. **EX:** 370, S38351; 373, S38400; 375, S38417; 455, S39442 (all specimens det. H. Mayrhofer). TLC: LW UV+ orange pigment; b) variolaric chemotype: terricolous, 942 m. **EX:** 408, S38872. TLC: variolaric acid (major); c) chemotype unknown: terricolous on beach ridges and over argillite, 0–922 m. **EX:** 407, F10107; **WA:** 105, F9995 (sub *Caloplaca sinapisperma*).
*Rinodina mniaroeiza* (Nyl.) Arnold — Terricolous on rock outcrop, 922 m. **EX:** 407, S38859 (det. H. Mayrhofer). TLC: atranorin. This has long been treated as a chemotype of *R. mniaroea* but molecular analyses (Resl *et al.* 2016) have shown it to be distinct from that species.
*Rinodina olivaceobrunnea* C. W. Dodge & G. E. Baker — Terricolous on fine organic accumulation on top of rock, alpine zone, 937 m. **EX:** 409, S38900 (det. H. Mayrhofer; sub *Pertusaria bryontha*).
*Rinodina orculata* Poelt & M. Steiner — Corticolous on *Picea sitchensis* krummholz, 922 m. **EX:** 407, S38819 (det. H. Mayrhofer). Previously reported in Alaska from KLGO (Spribille *et al.* 2010), and also known from Haida Gwaii (Sheard 2010).
*Rinodina pallidescens* Sheard & Tønsberg — Corticolous on *Alnus*, 5–10 m. **EA:** 123, F10242. This specimen was cited in the original description of the species (Sheard *et al.* 2014).
*Rinodina roscida* (Sommerf.) Arnold — Muscicolous on beach ridges, 0–5 m. **WA:** 105, F9995 (sub *Caloplaca sinapisperma*).
*Rinodina sheardii* Tønsberg — Corticolous on *Populus balsamifera* in outwash plain, 16 m. **GUS:** 533, T41565; [557, T41715b]. TLC: secalonic A, zeorin, gracilenta unknown, thiomelin.
*Rinodina stictica* Sheard & Tønsberg — Corticolous on *Alnus* (4×), *Oplopanax horridus* (1×), *Picea sitchensis* (2×) and *Sambucus racemosa* (1×), 0–8 m. **DUN:** 463, S39539 (but scant); **EA:** 440, S39213; **GB:** 864, S36335, S36346, S36363 (as ‘cf.’); Marble Mtn, S38006; 868, S36516; **GUS:** 857, S36153; 532, T41547, T41557; [574, T41815, T41822, T41823]; 231, M2767. TLC: atr, stictic acid, zeorin.
*Rinodina turfacea* (Wahlenb.) Körb. — Terricolous on fine organic accumulations over argillite, 872–942 m. **EX:** 407, F10123 (sub *Pertusaria dactylina*), S38853; 408, S38869; 456, S39471 (all det. H. Mayrhofer); 459, F10355. TLC: sphaerophorin.
*Ropalospora viridis* (Tønsberg) Tønsberg — Corticolous on *Alnus* (3×), *Malus fusca* (1×), *Picea sitchensis* (1×) and *Tsuga heterophylla* twigs (3×), 0–50 m. **DUN:** 333, S38079; 462, S39709 (unusual form); 562, T41750; **EX:** [858, S36171, S36185]; 433, S39080, S39082; **GB:** 868, S36480; N Sandy Cove, S36646; **GUS:** 855, S36031; 531, T41542. TLC: perlatolic acid. Specimen S39709 has an unusually dark overall colour as the areoles are distinctly brown and not greenish as in typical specimens. Based on morphology alone it could have been assigned to *Fuscidea arboricola*, but the chemistry matches *R. viridis*.
#*Roselliniella cladoniae* (Anzi) Matzer & Hafellner — Lichenicolous on *Cladonia* cf. *cariosa*, terricolous over shallow rock, 10 m. **WA:** 384, S38529.
**#*Roselliniopsis ventosa* (Rostr.) Alstrup — Lichenicolous on *Placopsis* sp. on coastal rock crags, 0–5 m. **DUN:** 586, P2157. Described from *Placopsis* in the Faroe Islands and otherwise reported from Greenland, the Azores, and Wrangel Island (Russia) in the Bering Sea (Zhurbenko 2009), as well as more recent reports from the United Kingdom.
*Rostania ceranisca* **(**Nyl.) Otálora *et al.* — Terricolous or over bryophytes, 22–922 m. **EX:** 407, F10128 (sub *Fuscopannaria* aff. *praetermissa*); **WA:** 318, S37910.
*Rostania occultata* (Bagl.) Otálora *et al*. — Corticolous on *Populus balsamifera*, 8 m. **EA:** 440, S39251 (sub *Fuscopannaria dillmaniae*).
*Rusavskia elegans* (Link) S. Y. Kondr. & Kärnefelt — Saxicolous, usually on calcareous rock. **GB:** Muir Glacier, reported by Lindblom (1997) based on an old specimen. Remarkably, this species was not seen during the present survey despite extensive sampling on calcareous rocks.
*Rusavskia sorediata* (Vain.) S. Y. Kondr. & Kärnefelt — Saxicolous on marble and gneiss just above tideline, sea level, 0–5 m. **GB:** 863, F9701; 864, F9732, S36389; **WA:** 548, T41625.
*Sagedia simoënsis* (Räsänen) A. Nordin *et al*. — Saxicolous on top of bird perch, 942 m. **EX:** 408, S38865 (det. T. Wheeler).
#*Sagediopsis campsteriana* (Linds.) D. Hawksw. & R. Sant. — Lichenicolous on *Ochrolechia subplicans*, saxicolous in talus, 907 m. **EX:** 373, S38390.
*Sagiolechia phaeospora* Fryday & T. Sprib. — See ‘Descriptions of New Genera and Species’.
*Sagiolechia protuberans (Ach.) A. Massal. — Saxicolous on slightly basic rocks near creek bank, 225 m. **EX:** 128, F10282. Though *S. protuberans* supposedly occurs only on limestone, we see no difference between our specimen and typical *S. protuberans*.
*Sagiolechia rhexoblephara* (Nyl.) Zahlbr. — Terricolous/muscicolous in the alpine zone, 900 m. **EX:** 217, M2613.
*Santessoniella arctophila* (Th. Fr.) Henssen — Terricolous on fine organic accumulations or on thin moss on raised beaches, once in *Plantago maritima* zone, 0–4 m. **EA:** 438, S39187, F10221; 232, M2770; **GB:** 873, S36663. *Santessoniella arctophila* is an orphaned taxon as the genus to which it belongs has been reduced to synonymy under *Psoroma* (Ekman *et al.* 2014). It appeared to be closely related to *Austroparmeliella lacerata* and *Psoroma tenue* in a multilocus study by Ekman *et al.* (2014) and to *Psoroma cinnamomeum* in another study based only on ITS rDNA (Elvebakk *et al.* 2010). It is not clear which genus name would be most appropriate for this group. One approach would be to extend the use of the name *Austroparmeliella* to this whole clade. However, notwithstanding suggestive results in Ekman *et al.* (2014), we are not convinced that the hypothesis that *Nebularia incrassata* is also part of this clade can be rejected. Determining the most appropriate of these alternatives is beyond the scope of the current study. A GLBA specimen (S36663, isolate T1221) is the source of DNA sequences published here (Fig. 9).
*Sarcogyne clavus (DC.) Kremp. — Saxicolous on pebbles in short turf below limestone crags, 0–20 m. **WA:** 104, F9961.
+*Sarea difformis* (Fr.) Fr. — Lignicolous on hanging wood of tip-up in muskeg hollow, 245 m. **EX:** 400, S38732. Ascospores *c*. 1.2 × 0.8 μm and hymenium *c.* 120 μm tall.
+*Sarea resinae* (Fr.) Kuntze — On resin, corticolous on sheltered *Tsuga mertensiana*, and on pitch of exposed wood at high water line, 155–271 m. **DUN:** 421, S38960; **EX:** 444, S39325.
*Schaereria cinereorufa* (Schaer.) Th. Fr. — Saxicolous in rock cracks on granitic mountain top, 618 m. **DUN:** 428, S39022 (sub *Pyrenopsis* sp.).
*Schaereria fuscocinerea* (Nyl.) Clauzade & Cl. Roux — Saxicolous, 900 m. **EX:** 218, M2670.
#*Sclerococcum boreale* (Holien & Ihlen) Ertz & Diederich — Lichenicolous on *Mycoblastus affinis, M. sanguinarius* and *Pertusaria subambigens* on *Alnus*, *Picea* and *Tsuga*, 0–60 m. **DUN:** 339, S38149 (sub *M. affinis*); 462, S39716 (sub *M. affinis*); 467, S39662 (sub *Muellerella hospitans*); A579, P2116; **EX:** [858, S36168 (sub *Platismatia norvegica*)]; [860, S36243]; **GB:** 868, S36506; **GUS:** 857, S36161; 868, S36461, S36507; 879, S36800 (sub *M. affinis*); A569, P2214, P2269, P2322, P2323, P2327, P2344, P2372, P2415, P2416, P2419; A578, P2190, P2196.
#*Sclerococcum deminutum* (Th. Fr.) Ertz & Diederich — Lichenicolous on *Biatora*, saxicolous over snowbed rock, and on *Ochrolechia androgyna* s. lat. over *Cupressus nootkatensis*, 42–92 m. **DUN:** 469, S39689; **WA:** 387, S38544.
#*Sclerococcum fissurinae* Pérez-Ort. — See ‘Descriptions of New Genera and Species’.
#*Sclerococcum frigidum* (Hafellner) Ertz & Diederich — Lichenicolous on *Brigantiaea fuscolutea*, 909 m. **EX:** 218, M2625.
**#*Sclerococcum gelidarium* Etayo & F. Berger — Lichenicolous on *Placopsis* sp., saxicolous, 903 m. **EX:** 406, S38785. Described from Iceland, this species has until now been otherwise known only from Chukotka in far eastern Russia (Zhurbenko 2009).
#*Sclerococcum lobariellum* (Nyl.) Ertz & Diederich — Lichenicolous on *Lobaria* sp., 15 m. **GUS:** A569, P2100, P2350.
#*Sclerococcum parasiticum* (Flörke) Ertz & Diederich — Lichenicolous on *Lepra subvelata*, *Mycoblastus sanguinarius* and *Ochrolechia* sp. over *Cupressus*, *Picea sitchensis* and *Pinus*, 0–20 m. **DUN:** 412, S38917; 464, S39606; 586, P2102; **GUS:** A569, P2289, P2300, P2321, P2371; A578, P2120; 229, M2747.
#*Sclerococcum purpurascens* (Triebel) Ertz & Diederich — Lichenicolous on cf. *Amygdalaria*, small rock on ridgetop, alpine zone, 918 m. **EX:** 454, S39382, S39404.
#*Sclerococcum urceolatum* (Th. Fr.) Ertz & Diederich — Lichenicolous on *Parmelia*, terricolous or muscicolous, 880 m. **EX:** 459, F10352 (sub *Scytinium intermedium,* n.v. SPO).
*Scoliciosporum chlorococcum* (Graewe ex Stenh.) Vězda — Corticolous on *Alnus*, *Oplopanax horridus* and *Picea sitchensis*, 0–27 m. **DUN:** 339, S38165; 463, S39520; **GB:** 864, S36329, S36359; **GUS:** 435, S39124; **WA:** 395, S38660, S38677; 224, M2704b, M2832d.
*#*Scoliciosporum intrusum* (Th. Fr.) Hafellner — Lichenicolous on *Lecanora* sp., on vertical sheltered rock with overhanging vegetation, 809 m. **EX:** 452, S39364.
*Scutula effusa* (Auersw. ex Rabenh.) Kistenich *et al.* (syn. *Bacidia auerswaldii* (Hepp ex Stizenb.) Mig.) — Corticolous on *Cupressus nootkatensis* and *Populus balsamifera*, 0–237 m. **EX:** 446, S39334; **GUS:** 341, S38245.
#*Scutula epiblastemica* (Wallr.) Rehm — Lichenicolous on *Peltigera collina*, corticolous on dead *Salix*, 18 m. **GUS:** 316, S37867.
*Scytinium callopismum* (A. Massal.) Otálora *et al*. — Saxicolous on limestone on high beach, 0–6 m. **GB:** 867, S36445; **WA:** 389, S38571.
[*Scytinium cellulosum* (P. M. Jørg. & Tønsberg) Otálora *et al*. — Corticolous on dead *Alnus*, 20 m. **GUS:** 557, T41716. Several other fragments similar to *L. cellulosum* were encountered (for example on S39236 sub *Arctomia delicatula* and S39278 sub *Bacidia* sp.) but were either sterile or too small to be placed with confidence.]
*Scytinium gelatinosum* (With.) Otálora *et al*. — Terricolous in alpine heath with rock outcrops, 883 m. **EX:** 405, F10088.
*Scytinium imbricatum* (P. M. Jørg.) Otálora *et al*. — Terricolous or on bryophytes over rock outcrops, 10–100 m. **WA:** 101, F9903.
*Scytinium intermedium* (Arnold) Otálora *et al*. — Terricolous/muscicolous, 880 m. **EX:** 459, F10352.
*Scytinium lichenoides* (L.) Otálora *et al*. — Muscicolous or terricolous over organic accumulations, sometimes thin accumulations over rock, also in *Dryas* mats in glacial forelands, 0–43 m. **EA:** 869, F9772; 872, F9801, F9808, F9809; 438, F10224; **GB:** 863, F9684, F9686, F9689; 864, F9733, S36378; 867, S36454; N tip of Willoughby Island, F9709; S Sandy Cove, S40758; **GUS:** 880, S36826; **WA:** 101, F9901 (sub *Thalloidima sedifolium*); 318, S37918.
*Scytinium schraderi* (Ach.) Otálora *et al*. — Terricolous on soil over limestone, 10–100 m. **WA:** 101, F9903 (sub *Scytinium imbricatum*).
*Scytinium subtile* (Schrad.) Otálora *et al*. — Corticolous on *Salix*, 2 m. **EA:** near 438, S39311.
*Scytinium tenuissimum* (Hoffm.) Otálora *et al*. — Terricolous on sandy soil in uplifted beach meadows, 2–3 m. **GUS:** State Dock, F9877.
*Siphula ceratites* (Wahlenb.) Fr. — Terricolous in wet muskeg and into snowbed-like montane sedge turfs, 20–450 m. **DUN:** between 412 and 415, S38923; 413, F10140; 464, S39623; 222, M2690. A highly distinctive species found during the present survey only in the Dundas Basin, where it was abundant. DNA from one sample was used as isolate P110 in the molecular study of Resl *et al.* (2015).
*#*Skyttea caesii* Diederich & Etayo — Lichenicolous on *Mycoblastus caesius*, 22 m. **GUS:** A569, P2328.
*Solorina bispora* Nyl. — Terricolous over limestone, 0–20 m. **WA:** 104, F9970; 381, S38472. Ascospores 2 per ascus, 75–80 μm long.
*Solorina crocea* (L.) Ach. — Terricolous in alpine snowbed, 20–894 m. **EX:** 372, S38370; **GB:** slopes and ridges of mainland immediately NW of Sebree Cove and W of Caroline Point, 1968, *Worley* 10625 (UBC, n.v.); Mt Wright, 1974, *Noble & Sandgren* 336 (MIN, n.v.); **GUS:** semi-open sand rise along road from Bartlett Cove to Gustavus, 1968, *Worley* 10970 (UBC); **WA:** Russell Island, 1976, *Neher* s. n. (UC). The herbarium records of this species suggest that a well-developed alpine lichen biota is present above the glacial trimline in upper Glacier Bay, an area that for logistical reasons could not be included in the present survey. The 1968 record from the Bartlett Cove road is also remarkable as this area was thoroughly searched in 2011/2012 and no *S. crocea* was found, perhaps overtaken by succession. See also the entry for *Thamnolia vermicularis*.
*Solorina octospora* Arnold — Terricolous between boulders, 919 m. **EX:** 374, S38416.
*Solorina spongiosa* (Ach.) Anzi — Terricolous on bare mineral soil along recently constructed drive, 10 m. **GUS:** Housing Compound Bartlett Cove, S36790.
#*Sphaerellothecium araneosum* (Rehm ex Arnold) Zopf — Lichenicolous on *Ochrolechia* sp., 22–230 m. **EX:** 566, P2389; 567, P2144; **GUS:** A569, P2269.
*#*Sphaerellothecium contextum* Triebel — Lichenicolous on *Protoparmelia badia*, saxicolous, 618–903 m. **DUN:** 428, S39016 (sub *Rhizocarpon intersitum*); **EX:** 406, S38782 (sub *Lecidea swartzioidea*).
*Sphaerophorus globosus* (Huds.) Vain. — Saxicolous on rock and in talus, 618–922 m. **DUN:** 428, S39002; **EX:** 370, S38339; 373, S38380; 407, S38841.
*Sphaerophorus tuckermannii* Räsänen — Corticolous on *Alnus*, *Picea sitchensis* and *Pinus contorta*, 0–569 m. **DUN:** 132, F10451; 337, S38121; 462, S39732; 464, S39601; **EX:** [858, S36216]; 312, S37813 (sub *Sphaerophorus venerabilis*); 358, S38324; **GUS:** 100, F9887; 855, F9607, F9608, S36010; 876, S36737; **GB:** Marble Mtn beach, S38003. Both this and the following species have also been commonly seen on *Tsuga heterophylla*, but not collected.
*Sphaerophorus venerabilis* Wedin *et al*. — Corticolous on *Alnus*, *Picea sitchensis* and *Pinus contorta*, also seen on *Tsuga heterophylla*, 0–222 m. **DUN:** 338, S38134; 464, S39605; **EX:** 312, S37813; **GB:** 868, S36474, S36485; **GUS:** 100, F9888; 856, F9624; 862, S36284; 316, S37874; road from Bartlett Cove to Gustavus, 1968, *Worley* 11081 (UBC, det. C. R. Björk, 2014).
*Spilonema americanum* (Henssen & Tønsberg) T. Sprib. *et al*. — Corticolous on *Alnus* (9×), *Malus fusca* (2×), *Picea sitchensis* (1×), *Populus balsamifera* (1×), *Tsuga heterophylla* (3×) and *Viburnum edule* (1×), 2–217 m. **EA:** 440, S39200; 441, F10203, F10204; 233, M2790; **EX:** [858, S36202, S36217]; 398, S38717; [V431, S39051]; 432, S39064; **GUS:** 111, F10060; 857, S36091; 879, S36807 (sub *Leptogidium dendriscum*), S36813, S36816 (sub *Biatora subduplex*); Tower Rd, S38279; 533, T41559; [557, T41691]; 576, T41837; **WA:** Blue Mouse Cove plot BM1a (GLBA herbarium).
*Spilonema maritimum* T. Sprib. & Fryday — See ‘Descriptions of New Genera and Species’.
*Sporodictyon schaererianum* A. Massal. — Saxicolous in limestone crags, 0–20 m. **WA:** 104, F9977. The affinity of F9977 to *S. schaererianum* was confirmed by ITS rDNA (Table 1).
*Sporodictyon terrestre* (Th. Fr.) S. Savić & Tibell — Terricolous on recently deglaciated soils, 30–50 m, also saxicolous on semi-inundated rocks on creek bank, 225 m. **EA:** 869, S36569; V553, F10648; **EX:** 128, F10281 (as ‘cf.’).
*Staurothele discedens* (Nyl.) Zahlbr. — Saxicolous on limestone crags behind beach, 10–15 m. **WA:** 105, F10011.
**Staurothele septentrionalis Lynge — Saxicolous on granitic rock, from raised beaches to glacial forelands, 0–115 m. **EA:** 870, F9784, F9792 (sub *Ionaspis ventosa*); 871, F9794; N shore of Muir Inlet, F9798; **WA:** 101, F9918 (sub *Lecidea lactea*), F9922; 105, F9983, F9990. ITS rDNA has been obtained from F9784 and F9794. A single brown ascospore was found inside a perithecium of F9784 which has led us to consider whether the specimens represented an immature stage of *S. arctica.* The length of the single brown ascospore, however, was no different to the length of those without pigmentation in the same perithecium and those of other samples of *S. septentrionalis* from Glacier Bay (26–32 μm). The dimensions of the measured ascospores are nearly identical to the size of ascospores reported from the type material by Lynge (1928) and smaller compared to *S. arctica* which has an ascospore length of 32–65 μm (Thomson 1991). Stenroos *et al.* (2016) consider *S. septentrionalis* a synonym of *S. clopima* (Wahlenb.) Th.Fr., but in that species most ascospores turn brown soon and the width of the ascospores is smaller, although there is some overlap (11–15 μm, rarely up to 18 μm in *S. septentrionalis*; 15–25 μm in *S. clopima*). Lynge (1928) did not find intermediate forms between the two species when he studied both taxa in his collections from Novaya Zemlya and we prefer to keep *S. septentrionalis* as a separate species for now. ITS rDNA was obtained for specimens F9784 and F9794 (Table 1). Previously known from north-eastern Europe (Russia; Lynge 1928).
**Staurothele succedens (Rehm) Arnold — Saxicolous on semi-inundated, slightly basic rock, 225 m. **EX:** 128, F10283. Previously known from Europe and Asia (Orange *et al*. 2009*b*).
*Staurothele cf. *verruculosa* J. W. Thomson — Saxicolous on semi-inundated, slightly basic rock, with *Atla recondita*, 225 m. **EX:** 128, F10302. A dark red to red-brown colour is regarded as characteristic for the exciple of *S. verruculosa* (Thomson 1991). The exciple in F10302 does not show any red component. Although all other characters match the description of *S. verruculosa*, the collection from Glacier Bay would represent a significant range extension compared to its currently known distribution from Arizona to Baja California. The diagnostic power of the exciple colour in this species needs further examination.
*Steineropsis alaskana* T. Sprib. & Muggia — Saxicolous and partially going over onto organic matter, on granite and argillite, 20–918 m. **DUN:** 419, S38953; **EX:** 374, S38414 (sub *Atrophysma cyanomelanos*); 454, F10330, F10350, S39383; 455, S39437 (fertile); **WA:** 103, F9948; 545, T41619. Described as a new genus and species from KLGO (Spribille *et al.* 2010). The Dundas collection S38953 lacks isidia and was initially thought to constitute an undescribed species, but the ITS rDNA sequence is identical to that obtained from *S. alaskana* (isolate T1187, Fig. 9). See also ‘Other Species Treated in Detail’ (above).
*Steineropsis laceratula* (Hue) T. Sprib. & S. Ekman — Corticolous on *Alnus*, *Cupressus nootkatensis* and *Picea sitchensis*, in beach fringe or on coastal headlands, also saxicolous in rock crevices on sea stacks, 2–86 m. **DUN:** 129, F10364 (sub *Normandina acroglypta*); 333, S38092; 462, F10368, S39738 (as ‘cf.’); 463, F10385, F10393, F10397, F10401; S39526, S39568, S39570, S39598; 467, F10428, S39668 (as ‘cf.’), S39672; 562, T41745 (conf. P. M. Jørgensen); **GUS:** [557, T41695 (conf. P. M. Jørgensen)]. TLC: atr. A 28S rDNA sequence of the dactyloid form was published by Schneider *et al.* (2015) from isolate T1188 under the name *Fuscopannaria laceratula*, and data from two other loci from the same isolate are provided here (Fig. 9). See also ‘Other Species Treated in Detail’.
+*Stenocybe clavata* Tibell — Corticolous on *Tsuga* trunk, 124 m. **DUN:** 334, S38101.
*Stereocaulon alpestre* (Flot.) Dombr. — Terricolous, 33 m. **GUS:** Tower Rd, S37516. TLC: atr, stictic, menegazziaic acids.
*Stereocaulon alpinum* Laurer — Terricolous on recent glacial gravels, on road banks, and on thin soil over rock, 0–43 m. **EA:** 869, F9770, F9777, S36549, S36551, S36567; **GB:** 865, 36412; 868, F9756; 873, S36669; **GUS:** 880, S36827; **WA:** 383, S38500. TLC: atr, lobaric acid. Previously noted by Cooper (1923) in early post-glacial successional stages.
*Stereocaulon arenarium* (Savicz) I. M. Lamb — Terricolous on thin soil over rock and in snowbed, alpine zone, 872–907 m. **EX:** 373, S38378; 406, S38800; 456, S39459; 457, S39480 (sub *Brigantiaea fuscolutea*). TLC: atr, porphyrilic acid.
*Stereocaulon botryosum* Ach. — Saxicolous, 7 m. **WA:** 385, S38532. TLC: atr, porphyrilic acid.
*Stereocaulon coniophyllum* I. M. Lamb — Saxicolous on sea stacks, on small boulders in recent glacial forelands, and in crevices of outcrops along seashore, 0–115 m. **DUN:** 339, S38206; **EA:** 869, F9763, F9766, F9767, S36558; between 870 and 871, S36587; terminus of Riggs Glacier, S40800; **WA:** 319, S37937; 383, S38483 (sub *Stereocaulon rivulorum*); 549, T41636. TLC: atr, lobaric acid.
*Stereocaulon depressum* (Frey) I. M. Lamb — Terricolous, 922 m. **EX:** 407, S38844. TLC: atr, lobaric acid, bourgeanic acid (major).
*Stereocaulon glareosum* (Savicz) H. Magn. — Terricolous on 20 yr-old gravel surfaces, 0–43 m. **EA:** 869, S36563; Muir Inlet, S40785. TLC: atr, lobaric acid. Known so far only from Upper Muir Inlet.
*Stereocaulon grande* (H. Magn.) H. Magn. — Saxicolous and terricolous, 0–75 m. **DUN:** 415, S38939; **GB:** 868, F9758, F9759; N tip Willoughby Island, F9716; **WA:** 319, S37945; 323, S37978. TLC: atr, lobaric acid.
*Stereocaulon groenlandicum* (E. Dahl) I. M. Lamb — Saxicolous on small boulders in *c.* 20 yr-old gravels, and on rocks on raised beaches, 0–43 m. **EA:** 869, S36548, S36555; Muir Inlet, S40770; terminus of Riggs Glacier, S40807; **WA:** 326, S37985; 384, S38519 (sub *Stigmidium beringicum*). TLC: atr, perlatolic acid.
*Stereocaulon intermedium* (Savicz) H. Magn. — Saxicolous on rocky seashores, 9–27 m. **DUN:** 339, S38208; 463, S39562; **EA:** 438, F10223 (cf.). TLC: atr, lobaric acid.
*Stereocaulon leucophaeopsis* (Nyl.) P. James & Purvis — Saxicolous on alkaline argillite, alpine heath, 700 m. **EX:** 449, F10320 (sub *Rhizocarpon anaperum*).
*Stereocaulon octomerum* Müll. Arg. — Saxicolous on rocks at edge of lake, 127 m. **DUN:** 419, S38958. TLC: atr, lobaric acid.
*Stereocaulon paschale* (L.) Hoffm. — Terricolous, 922 m. **EX:** 407, S38847. TLC: atr, lobaric acid.
*Stereocaulon rivulorum* H. Magn. — Terricolous or closely attached to rock, 10–43 m. **EA:** 869, F9768; **GB:** Muir Glacier, *Trelease* 1298a (NY, det. I. M. Lamb, 1972); **WA:** 319, S37949; 383, S38483 (on rock). TLC: atr, perlatolic acid.
*Stereocaulon sasakii* Zahlbr. var. *tomentosoides* I. M. Lamb — Terricolous on beach ridges, rock outcrops, and exposed crags above the supralittoral zone, 0–125 m. **DUN:** 463, F10394, F10399; **EA:** 438, S39177; Muir Inlet, S40774; **WA:** 388, S38564, S38565. TLC: atr, lobaric acid.
*Stereocaulon saviczii* Du Rietz — Saxicolous on boulders in snowy basin, 149 m. **DUN:** below 416, S38947. TLC: atr, lobaric acid.
*Stereocaulon spathuliferum* Vain. — Saxicolous, firmly attached to alkaline argillite, 883 m. **EX:** 405, F10103 (sub *Porpidia flavicunda*). TLC: atr, stictic acid.
*Stereocaulon tomentosum* Fr. — Terricolous on metamorphic rock just back from shore, and in *Dryas* mats in glacial forelands, 0–43 m. **EA:** N shore Muir Inlet, F9800; 869, F9769; **GB:** N tip Willoughby Island, F9712; **WA:** 384, S38513, S38515. TLC: atr, stictic acid, ±menegazziaic acid. WA specimens belong to var. *compactum* Frey.
*Stereocaulon vesuvianum* Pers. var. *nodulosum* (Wallr.) I. M. Lamb — Saxicolous or on fine organic accumulations in rock crevices, from rocky seashore crags to alpine zone, 10–922 m. **DUN:** 339, S38207; 415, S38940; 423, S38972; 426, S38990; 428, S39010, S39012 (as ‘cf.’); 467, S39656; 468, F10440 (sub *Porpidia* sp.); **EX:** 407, F10109, S38824; 455, S39428; **WA:** 384, S38516. TLC: atr, stictic, menegazziaic acids.
*Sticta arctica* Degel. — Loosely saxicolous on thin soil over rock, 883–942 m, only near the highest ridgetop rocks. **EX:** 373, S38396; 405, F10091; 408, S38873; 218, M2622.
*Sticta fuliginosa* (Hoffm.) Ach. — Corticolous on *Alnus*, *Picea sitchensis*, *Populus balsamifera* and *Tsuga heterophylla*, mostly associated with beach fringe, 2–33 m. **EA:** 123, F10246; 440, S39230, S39247; 441, F10216, F10217; S39265; **GB:** 874, S36708; S Sandy Cove, S40750; **GUS:** 138, F10477 (as ‘*sylvatica*’); 857, S36108; 878, S36765; [V329, S38025, S38041]; **WA:** Blue Mouse Cove plot BM2a (GLBA herbarium). The GLBA material of this species needs to be re-examined in the light of recent results from Europe showing *S. fuliginosa* to be a complex of at least four microspecies (Magain & Sérusiaux 2015).
*Sticta limbata* (Sm.) Ach. — Corticolous on *Alnus* and *Picea sitchensis*, 8–33 m. **EA:** 440, S39196; 442, S39289; **GB:** 868, S36517; **GUS:** [V329, S38031]; 576, S41827. Rather uncommon in GLBA, known from Muir Point, Seebree Island and Tower Road.
**Sticta rhizinata Moncada & Lücking — Corticolous on *Alnus* and *Picea sitchensis*, always associated with beaches or edges of meadows, 4–46 m. **GUS:** 857, S36117; 879, S36814; 434, S39096; Bartlett Cove, 28 August 2008, *Dillman* s. n. (GLBA-9016!). It has been known for some time that the North American Pacific coastal species referred to as *Sticta weigelii* (Ach.) Vain. in the literature (Goward *et al.* 1994) does not correspond to the type of that species (Brodo *et al.* 2001), which is from the island of Martinique in the eastern Caribbean (McDonald *et al.* 2003). Harris (1984) and later Brodo *et al.* (2001) referred the eastern North American and Great Lakes material to *Sticta beauvoisii* Delise, but Brodo (2016) cautioned that northern and western specimens may represent another species. The foliose symbiotic morphotypes of *Sticta* have been recently studied using a combination of morphological and molecular markers, with emphasis on the *S. fuliginosa* group (Magain & Sérusiaux 2015) and all known species from Colombia (Moncada *et al.* 2014), including multiple specimens of both *S. beauvoisii* and *S. weigelii* that ostensibly correspond to the geographical origins and types of those two species. While the genus *Sticta* still awaits a comprehensive systematic treatment, the combination of DNA sequence data published in these two studies has enabled GLBA specimens to be tentatively placed in a broader context of molecular diversity within the genus *Sticta* (see Supplementary Material Fig. S1, available online). We used ITS sequences from every available species sequenced by Moncada *et al.* (2014), along with an ITS sequence of *Sticta torii* Simon & Goward, a species recently described from British Columbia (Simon *et al.* 2018) and combined these with the four-locus data set of Magain & Sérusiaux (2015). The resulting topology (see Supplementary Material Fig. S1) improves the support for a few species groups compared to a topology derived solely from ITS sequences, but most relationships remain unsupported. A specimen (*Stenroos* 4816 (TUR)) assigned to *S. weigelii* by Stenroos *et al.* (2003) and Högnabba *et al.* (2009) from Guyana resolves in a different place within the same broad clade that also harbours *S. beauvoisii*, perhaps reflecting inconsistency in identifying the species used as DNA vouchers in different studies. *Sticta* material from GLBA is recovered within this broader *beauvoisii*/*weigelii* clade but is clearly closest to the Colombian species *S. rhizinata* (one nucleotide difference in ITS1, a situation recalling *S. fuliginosa* and *S. limbata*; Magain & Sérusiaux 2015). Despite having three sequenced loci, *S. rhizinata* is, however, still not resolved in its relationships to other *Sticta* species owing to missing data; only nine of the 52 sequences included in the *beauvoisii*/*weigelii* clade that we obtained from our own and published data have sequences in addition to ITS. Initially we thought that the GLBA material differed morphologically, as Colombian *S. rhizinata* forms prominent rhizines and the thallus tends to form branches off a longer central thallus axis (R. Lücking, personal communication and shared specimen material). However, closer examination of multiple Alaskan specimens, by carefully extricating thalli from the moss cushions in which they are often embedded, revealed rhizines and a main axis-branching pattern similar to that of the type.
#*Stigmidium beringicum* Zhurb. & Triebel — Lichenicolous on *Stereocaulon* spp. (including *S. groenlandicum*), saxicolous, 10–406 m. **DUN:** 423, S38967; **EA:** 869, S36560; **WA:** 384, S38519; A570, P2265; A575, P2096, P2248.
#*Stigmidium conspurcans* (Th. Fr.) Triebel & R. Sant. — Lichenicolous on *Psora rubiformis*, thin soil over rock, 895–937 m. **EX:** 370, S38362 (sub *Psora rubiformis*); 407, S38823; 409, S38898.
#*Stigmidium peltideae* (Vain.) R. Sant. — Lichenicolous on *Peltigera* sp. in muskeg, 233 m. **EX:** 567, P2140.
*#*Stigmidium squamariae* (B. de Lesd.) Cl. Roux & Triebel — Lichenicolous on *Lecanora polytropa*, pebble beach, 2 m. **WA:** A576, P2186.
*Szczawinskia tsugae* A. Funk — Corticolous, mainly on *Tsuga* twigs, but also on *Alnus* (2×) and *Malus fusca* (3×), 21–50 m, often mixed in with other lichens and seen first when processing specimens in the laboratory. **DUN:** 560, T41736; **EX:** [858, S36194, S39196, S36197 (sub *Micarea peliocarpa*), S36204]; [V431, S39046]; 433, S39077; 211, M2566; [225, M2725]; [226, M2730]; **GUS:** 855, F9615, S36017; 878, S36770 (sub *Parmeliella parvula*), S36774 (sub *Micarea cinerea*), S36785; [V329, S38033]; [557, T41696].
*Tephromela atra* (Huds.) Hafellner — Saxicolous, populations found on gneiss just above high tide (0–5 m) and on soft argillite in the alpine zone (883–903 m). **DUN:** 339, S38202; **EX:** 370, S38350-B; 405, F10078; 406, S38786-B; 453, F10322. GLBA material acquired during this survey was included in a multilocus DNA study of this species (Muggia *et al.* (2014), isolates L1870 [S38786-B], L1871 [S38350-B] and L1886 [S38202]).
*Tetramelas* aff. *chloroleucus* (Körb.) A. Nordin — Corticolous on *Picea sitchensis*, *Populus balsamifera* and *Tsuga heterophylla*, 0–50 m. **EX:** [858, S36169 (cf.)]; **GUS:** 435, S39123 (sub *Buellia triseptata*), S39124 (sub *Scoliciosporum chlorococcum*); **WA:** 395, S38669. The ascospores in our material are (19–)24–29 × 9–12 μm, longer and wider than the values reported in *T. chloroleucus* from Fennoscandia (16.8–20.6 × 7.1–9.1 μm; Nordin 2000). It is not clear, however, whether Fennoscandian and central European specimens are conspecific (unpublished DNA studies), and the type is from central Europe (Koerber 1865; not 1860 as stated in Nordin 2000). Resolving the taxonomy of this group is beyond the scope of the present study.
*Tetramelas insignis* (Nägeli ex Hepp) Kalb — Muscicolous in alpine tundra, 895 m. **EX:** 370, S38338.
*Tetramelas papillatus* (Sommerf.) Kalb — Terricolous/muscicolous in alpine heath with rock outcrops, 922 m. **EX:** 407, F10128 (sub *Fuscopannaria praetermissa* aff.).
*Thalloidima sedifolium* (Scop.) Kistenich *et al.* — Muscicolous and terricolous on fine organic accumulations over limestone, 10–100 m. **EA:** 872, F9807; **GB:** 867, S36440; **WA:** 101, F9901; 318, S37915; 381, S38471.
*Thamnolia vermicularis* (Sw.) Schaer. s. lat. — Terricolous in heath and on alpine turf, sometimes sprawling over rock faces, 406–907 m. A low elevation record from *c*. 20 m also exists from 1968 (see below). **DUN:** 423, S38981; 428, S38999; **EX:** 370, S38359; 373, S38403; 406, S38794 (sub *Bryocaulon divergens*); 456, S39466, S39476; 457, S39477; **GB:** slopes and ridges of mainland immediately NW of Sebree Cove and W of Caroline Point, 1968, *Worley* 10820 (UBC, n.v.); Mt Wright, 1974, *Noble & Sandgren* 336 (MIN, n.v.); Beardsley Islands, *Stephens* 241 (UC); **GUS:** semi-open sand rise along road from Bartlett Cove to Gustavus, 1968, *Worley* 10981 (UBC). We are convinced this species no longer occurs on the Bartlett Cove moraine; see also *Solorina crocea*.
*Thelenella pertusariella (Nyl.) Vain. — Corticolous on *Salix*, 4 m. **EA:** E of Muir Point, M2816.
*Thelidium aff. *fontigenum* A. Massal. — Saxicolous on seashore limestone, 5 m. **EA:** 122, F10235, F10236. Exciple KOH+ magenta and ascospores 35–50 μm long with 1(–2) longitudinal septa.
*Thelidium incavatum Nyl. ex Mudd — Saxicolous on limestone crags behind beach, 10–15 m. **WA:** 105, F10008.
*Thelidium pyrenophorum* (Ach.) Mudd — Saxicolous on upper beach in *Plantago maritima* zone, 4 m. **EA:** 438, S39175.
**Thelidium submethorium (Vain.) Zschacke — Saxicolous on semi-inundated, slightly basic rock, with *Hymenelia lacustris*, 225 m. **EX:** F10285. Previously known from NE and central Europe (Thüs & Schultz 2008).
**Thelignya groenlandica (E. Dahl) Henssen — Saxicolous on limestone rocks on seashore, 0–5 m. **GB:** 864, F9729 (det. M. Schultz). Previously known only from the type collection, from Greenland (Dahl 1950).
*Thelignya lignyota* (Wahlenb.) P. M. Jørg. & Henssen — Saxicolous on rocks at the high tide line or on adjacent beaches (6×), in glacial foreland *Dryas* mats (1×) and in alpine heaths (1×), 0–115 m and again at 890 m. **EA:** 870, F9793; 438, S39174; **EX:** 438, F10225, F10185; 453, F10326 (det. M. Schultz; sub *Lecidea lapicida*); 218, M2636; **GB:** 865, F9738, F9742; 873, F9823; **WA:** 318, S37906; 322, S37966; 323, S37970; 325, S37982; 391, S38625; 392, S38650; 394, S38655; Scidmore Beach, S37999. A poorly known species globally that appears to be fairly common in GLBA; also reported from Lake Clark by McCune *et al.* (2018). This corresponds to the species treated under *Poroscyphus dispersus* Dahl by Thomson (1997), who highlighted its distinctive green epithecium (I. Brodo, personal communication).
**Thelocarpon depressellum Vain. — Lignicolous on *Cupressus nootkatensis*, 52–257 m. **DUN:** 219, M2678; **EX:** 213, M2570. This poorly known species was described from moist, rotten wood in Finland by Vainio (1883) and has otherwise been reported only from Slovakia and Sweden. It is characterized by its open, disc-shaped apothecia and spherical ascospores 1.5–2 μm diam. (Foucard 2001).
#*Thelocarpon epibolum* Nyl. var. *epithallinum* (Leight. ex Nyl.) G. Salisb. — Lichenicolous on *Lobaria pulmonaria*, 22–50 m. **EX:** [858, S36221]; **GUS:** A578, P2356, P2380.
*Thelocarpon immersum* Fryday — See ‘Descriptions of New Genera and Species’.
*Thelocarpon superellum Nyl. — Terricolous in *Dryas* mats in recent glacial forelands, 115 m. **EA:** 870, F9776 (sub *Verrucaria xyloxena*).
*Thelotrema lepadinum* (Ach.) Ach. — Corticolous on *Alnus* (10×), *Cupressus nootkatensis* (3×), *Malus fusca* (1×), *Menziesia ferruginea* (1×), *Picea sitchensis* (1×), *Tsuga heterophylla* (3×), *Vaccinium ovalifolium* (1×) and *Viburnum edule* (1×), 0–222 m. **DUN:** 114, F10138; 333, S38073, S38087, S38089, S38078; 334, S38096; 336, S38116; 412, S38915; 462, S39699; 466, S39635; 467, S39641; 562, T41753; **EX:** 126, F10253; [860, S36245]; 313, S37836; [V431, S39039, S39052]; 433, S39072; 211, M2565; **GUS:** 856, F9630; 855, S36036; [V329, S38021]; 341, S38233; 436, S39141. Thalli on *Cupressus* can be exceptionally large, develop a peculiar cracking around the ascomatal rim and possess ascomata larger than in typical *T. lepadinum.* However, ITS rDNA from a specimen from *Cupressus* (S39635: isolate T1344, Table 1) did not differ appreciably from that in a typical form (S38096; isolate P129, previously published by Resl *et al.* 2015).
*Thelotrema aff. *suecicum* (H. Magn.) P. James — Corticolous on *Alnus* along small mountain streams, 9–180 m. **DUN:** 333, S38072; nr 429, S39033; 462, S39699, S39705; 562, T41742; **EX:** [V431, S39052]; **GB:** 556, T41655; **GUS:** 855, S36036; 341, S38233 (cf.). New to western North America, previously known in North America from the maritime provinces of eastern Canada. Found in GLBA on the Taylor Peninsula, at Fern Harbor and in the Dundas Basin. The specimens are tentatively assigned to *T. suecicum* here, as although the ascospores are mostly in the range of 30–35 μm long, a few reach 43 μm, which is too long for *T. suecicum*.
*Tholurna dissimilis* (Norman) Norman — Corticolous on *Picea sitchensis* krummholz, 883–922 m. A herbarium record of this species, from Lituya Bay on the outer coast, has not been checked (*Lawrence* s. n., MIN). **EX:** 405, S38758; 407, S38807.
*Tingiopsidium elaeinum* (Wahlenb. ex Ach.) Hafellner & T. Sprib. — Saxicolous on recently exposed rocks, common in recently exposed glacial forelands and on raised beaches, 0–115 m. **EA:** 870, F9783, S36573, S36582; **GB:** 868, F9750; **WA:** 104, F9962 (sub *Rhizocarpon infernulum*); 319, S37935; 382, S38474; 385, S38534; 389, S38578; 391, S38630 (sub *Tingiopsidium isidiatum*); 205, M2493; 208, M2522. *Trelease* 988 (NY) from Muir Glacier, collected 9 June 1899, has been annotated as *Vestergrenopsis elaeina* by R. C. Harris. The same specimen was originally reported as *Parmelia olivacea* by Cummings (1904).
*Tingiopsidium isidiatum* (Degel.) Hafellner & T. Sprib. — Saxicolous on boulders, pebbles and snowbed rocks, 2–100 m. **WA:** 381, S38454; 385, S38535; 387, S38546; 391, S38630.
*Toensbergia blastidiata* T. Sprib. & Tønsberg — See ‘Descriptions of New Genera and Species’.
*Toensbergia geminipara* (Th. Fr.) T. Sprib. & Resl (syn. *Pertusaria geminipara* (Th. Fr.) C. Knight ex Brodo) — Terricolous on subalpine to alpine sod, or on thin, fine organic accumulations over rock, 406–918 m. **DUN:** 423, S38969, S38971; 428, S39027; **EX:** 372, S38371 (fertile), S38376 (sub *Lepra dactylina*); 454, F10339; **WA:** 383, S38484. For details on the new combination, see the discussion of *Toensbergia blastidiata* in ‘Descriptions of New Genera and Species’.
*Toensbergia leucococca* (R. Sant.) Bendiksby & Timdal — Corticolous on krummholz *Picea sitchensis*, 883–922 m. **EX:** 405, S38769; 407, S38813.
*Toninia squalescens* (Nyl.) Th. Fr. — Terricolous/saxicolous on fine organic accumulation over granitic rock, snowy area, 350 m. **DUN:** 119, F10156; 221, M2681.
*Toninia squalida* (Ach.) A. Massal. — Muscicolous on rocks at edge of lake, and over shallow soil accumulations, 10–127 m. **DUN:** 419, S38954; **WA:** 101, F9906.
*Toniniopsis aromatica* (Sm.) Kistenich *et al*. — Saxicolous on limestone crags, 10–15 m. **WA:** 105, F10015.
*Toniniopsis subincompta* (Nyl.) Kistenich *et al.* (syn. *Bacidia subincompta* (Nyl.) Arnold) — Corticolous on *Alnus* and *Populus balsamifera*, close to seashore, 7–33 m. **EA:** 123, F10250 (sub *Mycobilimbia tetramera*); 440, S39250; 233, M2789; no waypoint, M2818; **EX:** 236, M2828; **GB:** 866, S36419; **GUS:** Tower Rd, S37509, S37510, S37511; 210, M2546; **WA:** 395, S38664 (sub *Phaeophyscia ciliata*), S38678. S37511 is pigment-deficient in the apothecia.
*Trapelia coarctata* (Sm.) M. Choisy — Saxicolous on creekside rocks, in *Fauria-Vaccinium* snowbeds, and on gneiss rocks above the high tide line, 0–700 m. **DUN:** 462, F10370; **EX:** 310, S37806; 449, F10319, S39362.
*Trapelia corticola* Coppins & P. James — Corticolous on *Picea sitchensis*, on rain-exposed *Tsuga heterophylla* trunks, on stick of tip-up, also lignicolous on wood in bog, 0–155 m. **DUN:** 131, F10450; nr 334, S38109; 462, S39744; **EX:** [858, S36210]; 444, S39323; **GUS:** 855, S36019; 876, S36735; [557, T41718b (richly fertile)].
*Trapeliopsis gelatinosa* (Flörke) Coppins & P. James — Terricolous on tip-up and peat hags, 9–50 m. **DUN:** 463, S39596; **EA:** V553, F10647.
[*Trapeliopsis granulosa* (Hoffm.) Lumbsch — Terricolous on organic accumulations, 50 m. **EX:** 858, F9652, F9654.]
**Trapeliopsis glaucolepidea (Nyl.) Gotth. Schneid. — Terricolous on fine organic matter, granitic erratic in muskeg, 22 m. **DUN:** 468, F10440 (sub *Porpidia* sp.). A small collection originally identified as *T. percrenata*, which we recognize as a synonym of *T. glaucolepidea* (Resl *et al.* 2015). This is a widely disjunct, cosmopolitan species (Palice & Printzen 2004).
*Trapeliopsis pseudogranulosa* Coppins & P. James — Muscicolous on mossed-over rotten root table, also corticolous on *Picea sitchensis*, 0–40 m. **DUN:** 114, F10135; 564, T41760a; **GUS:** 855, S36048; 876, S36724. TLC (F10135): gyrophoric, lecanoric acids; KOH+ violet anthraquinone present. TLC (T41760a): gyrophoric acid only.
#*Tremella cetrariicola* Diederich & Coppins — Forming galls on *Tuckermannopsis chlorophylla*, 0–10 m. **DUN:** 467, F10433; **GB:** 868, S36544; **GUS:** 435, S39134.
#*Tremella cladoniae* Diederich & M. S. Christ. — Lichenicolous on *Cladonia* (including *C. pyxidata*), on stick in muskeg, or corticolous on *Alnus* or *Populus balsamifera*, 0–294 m. **EX:** 352, S38287; **GB:** 864, S36358; 868, S36478; **WA:** A573, P2156, P2368.
#*Tremella nephromatis* Diederich — Lichenicolous on *Nephroma* (including *N. parile*), corticolous on *Picea sitchensis* and *Ribes lacustre*, 0–33 m. **EA:** 123, F10249; **GUS:** 857, S36120; [V329, S38044]; 341, S38256.
*Tremolecia atrata* (Ach.) Hertel — Saxicolous on cobble on old beach, also on argillite rocks in the alpine zone, 0–937 m. **EX:** 407, S38835 (sub *Pertusaria* sp. S38786); 409, S38881; 218, M2626 (sub holotype of *Lecanora viridipruinosa*); **GUS:** 435, S39136.
*Trimmatothelopsis dispersa (H. Magn.) K. Knudsen & Lendemer — On metamorphic rock (hornblende augen gneiss), in the supralittoral zone, sea level. **DUN:** 462, F10371.
*Tuckermannopsis chlorophylla* (Willd. ex Humb.) Hale — Two morphs were observed. These were a) the typical morph: corticolous on *Alnus* and *Picea sitchensis* (including krummholz), lignicolous on dock and on beach logs, 0–883 m. **EX:** 405, S38764; 217, M2597; **GB:** Willoughby Island, S36319 (sub *Physcia tenella*); 864, S36341; **GUS:** 107, F10038; 138, F10470; NPS dock, S36166; 876, S36722; b) a pale, narrow-lobed morph: corticolous on *Alnus*, and on twigs of *Picea sitchensis* and *Tsuga heterophylla*, 0–27 m. **DUN:** 339, S38181, S38192; 467, F10432, F10433, F10434; 573, T41810; **EA:** nr 438, S39301; **GB:** 868, S36498; Marble Mtn beach, S38008; **GUS:** 857, S36113; 434, S39112; **WA:** Blue Mouse Cove plot BM1a, BM2a (GLBA herbarium). The pale morph tends to be found on thin branches far away from tree trunks and to have narrow, ribbon-shaped, light-coloured thalli. No genetic difference was found in the lecanoromycete fungus between pale morphs (isolates T1081, T1096, Table 1) and the typical morphs (T1080, T1211).
*Tuckermannopsis orbata* (Nyl.) M. J. Lai — Corticolous on *Picea sitchensis* in krummholz, 922 m. **EX:** 407, S38820 (sub *Japewia subaurifera*).
*Umbilicaria angulata* Tuck. — Saxicolous on smooth granite, 4 m. **EA:** terminus of Riggs Glacier, S40803.
*Umbilicaria cylindrica* (L.) Delise ex Duby — Saxicolous on smooth granitic rock, 4 m. **EA:** terminus of Riggs Glacier, S40802, S40805.
*Umbilicaria hyperborea* (Ach.) Hoffm. — Saxicolous on exposed rock, 10–907 m. **DUN:** 423, S38973; 428, S39019; **EX:** 373, S38391; **WA:** 102, F9940 (as var. *radicicola*). Also reported from Muir Glacier by Degelius (1937).
*Umbilicaria polyphylla* (L.) Baumg. — Saxicolous on smooth, bare rock, 4 m. **EA:** near terminus of Riggs Glacier, S40799.
*Umbilicaria proboscidea* (L.) Schrad. — Saxicolous. **GB:** Muir Glacier, 1897, *Kincaid* (UPS L-59596!), reported by Degelius (1937).
*Umbilicaria torrefacta* (Lightf.) Schrad. — Saxicolous on exposed rock, 10–883 m; also reported for Muir Glacier by Degelius (1937). **EA:** between 870 and 871, S36590; terminus of Riggs Glacier, S40804; **EX:** 405, F10086; 406, S38783; **GB:** Muir Glacier, 1897, *Kincaid* (UPS, n.v.); **WA:** 101, F9904, F9905.
*Umbilicaria virginis* Schaer. — Saxicolous. **WA:** Russell Island, 1976, *Neher* s. n. (UC, n.v.). A duplicate at WIS was seen by J. W. Thomson.
*Usnea* cf. *cylindrica* P. Clerc — Corticolous on *Picea sitchensis* on coastal headlands, 9 m. **DUN:** 462, S39743; 463, S39524, S39554 (all det. P. Clerc). TLC: usnic, salazinic acids. This species was reported as new to North America from SE Alaska by Dillman *et al.* (2012).
*Usnea* cf. *dasopoga* (Ach.) Nyl. — Corticolous on *Picea sitchensis* (5×) and *Tsuga heterophylla* (1×), 0–27 m. **DUN:** 339, S38157, S38220; 463, S39533, S39560; **EA:** 441, 39286; **GUS:** 436, S39143, S39147 (all det. P. Clerc). TLC: usnic, salazinic acids.
*Usnea longissima* Ach. — Corticolous on *Picea sitchensis*, *Pinus contorta* and *Tsuga heterophylla*, 0–233 m. **DUN:** 463, S39537; **EX:** 314, S37851; 432, S39068 (sorediate!); **GB:** 868, S36486; **GUS:** seen but not collected near Annie Mae Lodge, 2014 (all collected specimens det. P. Clerc). TLC: usnic acid, cf. diffractaic (2×) and usnic, barbatic acids (1×).
*Varicellaria rhodocarpa* (Körb.) Th. Fr. — Corticolous on *Alnus* trunk and on dead *Tsuga heterophylla*, 222–332 m. **DUN:** 338, S38128; 569, T41770; **EX:** 365, S38334. TLC: lichexanthone, lecanoric acid.
*Verrucaria aethiobola* Wahlenb. — Saxicolous on semi-inundated, slightly basic rock, with *Hydropunctaria alaskana, Verrucaria aquatilis* and *V. anziana*, 0–225 m. **EX:** 128, F10286; **DUN:** 133, F10455 (sub *Verrucaria anziana*), F10459 (sub *Verrucaria aquatilis*).
**Verrucaria anziana Garov. — Saxicolous on semi-inundated, slightly basic rock, with *Hydropunctaria alaskana* and *Verrucaria aethiobola*, 0–225 m. **EX:** 128, F10286 (sub *Verrucaria aethiobola*); **DUN:** 133, F10455. The identity of specimens F10286 and F10455 as *V. anziana* was confirmed by ITS rDNA (Table 1). Previously known from Europe (Orange *et al*. 2009*c*). *Verrucaria anziana* has long been seen as a synonym of *V. latebrosa* (Thüs & Schultz 2008), and North American vouchers of that species should be re-examined.
*Verrucaria aquatilis Mudd — Saxicolous on hornblende augen gneiss beside creek, with *Verrucaria aethiobola*, 0–5 m. **DUN:** 133, F10459.
*Verrucaria ditmarsica* Erichsen — Siliceous cobbles on shore, 0–5 m. **GB:** 866, F9745, F9746.
*Verrucaria epimaura* Brodo — Intertidal with sedimentary boulders, sea level. **GUS:** 875, F9836, F9837, F9838, F9839.
*Verrucaria halizoa* Leighton — Tidal flats with sedimentary rocks and boulders, sea level; pebbles in tidal creek, 3 m. **GB:** 873, F9819; 874, F9833.
*Verrucaria margacea* Wahlenb. — Saxicolous on semi-inundated, slightly basic rock, 225 m. **EX:** 128, F10293, F10294.
*Verrucaria xyloxena Norman — Tundra with bryophytes and *Dryas*, 115 m. **EA:** 870, F9776.
*Vezdaea acicularis* Coppins — Terricolous in recently deglaciated soils (20 yr), 0–43 m. **EA:** 869, S36562.
**Vezdaea aestivalis (Ohlert) Tscherm.-Woess & Poelt — Lichenicolous on *Nephroma* and partly corticolous on *Populus balsamifera*, 8–40 m. **DUN:** 114, F10136; **EA:** 440, S39256. We have also seen specimens from coastal British Columbia and KLGO: Canada, British Columbia, Nisga'a highway (113), Beaupre Falls trail, 55.16940°N, 129.01723°W, on moribund cyanolichen thalli in lower canopy of *Picea sitchensis*, 121 m, 28 October 2016, *Spribille* s. n. *& A. Simon* (CANL, UPS); USA, Alaska, Klondike Gold Rush National Historical Park, Chilkoot Trail, general area opposite confluence of West Creek/Taiya River, 59°31.816′N, 135°20.392′W, on moss tassels on *Populus balsamifera*, 14 m, 4 August 2008, *T. Spribille* 27291 *& S. Pérez-Ortega* (KLGO). Widely reported from Europe.
**Vezdaea cobria Giralt *et al*. — Muscicolous in underhang under stump, 8 m. **EA:** 234, M2803. Described from Austria and the United Kingdom (Giralt *et al.* 1993), and since reported only from the Czech Republic (Liška *et al.* 2008).
*Vulpicida pinastri* (Scop.) J. E. Mattsson & M. J. Lai — Uncommon, corticolous on krummholz *Picea sitchensis* branches, 830–883 m. **EX:** 405, S38761; 404, F10077.
*Wahlenbergiella mucosa* (Wahlenb.) Gueidan & Thüs — Saxicolous on seashore rocks, limestone, 0–5 m. **EA:** 122, F10230.
#*Xenonectriella nephromatis* Pérez-Ort. — See ‘Descriptions of New Genera and Species’.
*Xylographa bjoerkii* T. Sprib. — Lignicolous on beached log, 2 m. **DUN:** 462, S39752. Another specimen from DUN (568, T41768) contains confriesiic acid and has the goniocystose thallus of *X. bjoerkii*, but the ascomata are much larger and reminiscent of *X. erratica*. This specimen requires more detailed study.
*Xylographa erratica* T. Sprib. — Driftwood at high tide line, sea level. **DUN:** 571, T41775a, T41775b.
*Xylographa hians* Willey ex Tuck. — Lignicolous on logs, exposed dead wood of otherwise live trees, on snags and on driftwood, 0–213 m. **DUN:** 113, F10133; 337, S38126; **EX:** [125, F10270]; [858, S36222]; **GB:** 874, S36713; **GUS:** 107, F10029; 856, F9619, S36071-B; 316, S37886. TLC: stictic, norstictic acids. DNA from two specimens from GLBA was used for analysis by Spribille *et al.* (2014*b*).
*Xylographa opegraphella* Nyl. ex Rothr. — Lignicolous on beach logs and driftwood along seashore, 0–9 m. **DUN:** 462, S39750; **EA:** 124, F10251; 438, F10184, S39293; **GUS:** 876, S36740; **WA:** 396, S38689. TLC: stictic, norstictic acids.
*Xylographa parallela* (Ach. : Fr.) Fr. — Lignicolous on driftwood and interglacial wood, 0–43 m. **EA:** 869, S36553 (s. lat.); **WA:** Scidmore Beach, S37996 (sub *Xylographa vitiligo*), S37997. TLC: nil or stictic acid.
*Xylographa schofieldii* T. Sprib. — Lignicolous on logs, above the high tide line (1×), in open mixed forest and *Malus* scrub (1×), and on *Pinus contorta* wood in open muskeg (1×), 0–213 m. **DUN:** 462, S39751; **EX:** 312, S37817; 433, S39088; [125, F10275]. TLC: confriesiic acid. The type locality is near lower Falls Creek NE of Gustavus (Spribille *et al.* 2014*b*). Two of the collections reported here (F10275 and S37817) were overlooked in the original paper. Otherwise known only from Haida Gwaii (British Columbia).
*Xylographa trunciseda* (Th. Fr.) Minks ex Redinger — Lignicolous on snags and logs, 20–217 m. **EX:** 398, S38719; **GUS:** 856, S36071; 876, S36743 (sequenced: Clade I in Spribille *et al.* 2014*b*); 204, S38433. TLC: confriesiic acid.
*Xylographa vitiligo* (Ach.) J. R. Laundon — Lignicolous on beach logs, snags and exposed wood of otherwise live *Tsuga mertensiana*, 2–740 m. **EX:** [125, F10272]; 403, S38746; 410, S38911; **GUS:** 856, S36071-C; **WA:** Scidmore Beach, S37996. TLC: stictic acid, ±sats. One GLBA specimen was the source of a DNA isolate used by Spribille *et al.* (2014*b*).
*Xylopsora friesii* (Ach.) Bendiksby & Timdal — Corticolous on trunks of veteran *Tsuga heterophylla*, montane, 396–687 m. **EX:** 354, S38294 (sub *Chaenotheca ferruginea*), S38297; 358, S38309; 448, F10317, S39347 (sub *Arthonia arthonioides*).
*Zahlbrucknerella calcarea* (Herre) Herre — Saxicolous on limestone rock face near shore, 0–5 m. **GB:** 867, F9747 (det. M. Schultz).

## Supplementary material

For supplementary material accompanying this paper visit http://dx.doi.org/10.1017/S0024282920000079. click here to view supplementary material
