# Supplementary material for: Lichens and associated fungi from Glacier Bay National Park, Alaska
Source: Lichenologist (Lond). 2020 May 11;52(2):61–181. doi: 10.1017/S0024282920000079 (PMC7398404; doi:10.1017/S0024282920000079)
Supplement: Supplementary file 1 [file S0024282920000079sup001.zip › S0024282920000079sup001/Supplementary_Electronic_Appendices.docx]

**Supplementary Electronic Appendices**

Supplementary Table S1: Collectors of lichens in Glacier Bay 1899–2010.

Supplementary Table S2: GPS coordinates for localities cited in the text.

Supplementary Table S3: DNA accession table for sequences used in trees, from vouchers from which no new sequences are published here.

Supplementary Table S4: Data tables for species matrices used to construct Venn diagrams. Occurrence in sectors designated in Fig. 1A and described in text.

Supplementary Fig. S1: Majority-rule consensus tree of the genus *Sticta*, showing position of GLBA-derived specimen of *S. rhizinata*, based on four loci. Dots and dashes to the right of tip names indicate presence and absence of loci, respectively, in the following order: ITS, 28S, mtSSU, *RPB1*. Bold indicates the GLBA specimen. Values indicate percent bootstrap support. Alphanumeric codes in brackets are identifiers unique to this study. Voucher information and GenBank Accession numbers are outlined in Table 1 and Supplementary Material Table S3 (available online).
