# Supplementary figures and images for: Lichens and associated fungi from Glacier Bay National Park, Alaska
Source: Lichenologist (Lond). 2020 May 11;52(2):61–181. doi: 10.1017/S0024282920000079 (PMC7398404; doi:10.1017/S0024282920000079)

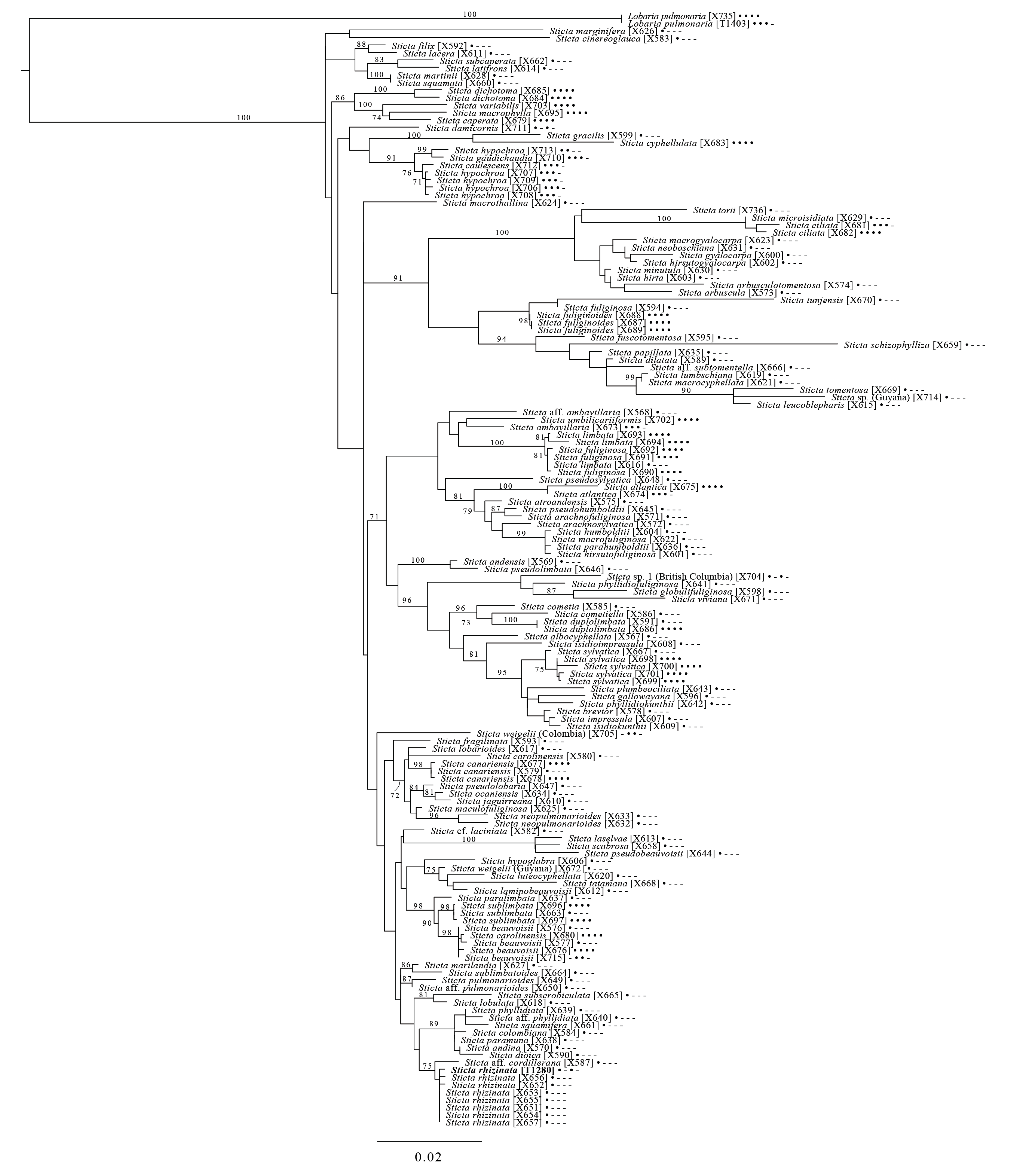

Supplement: Supplementary file 1 [file S0024282920000079sup001.zip › S0024282920000079sup001/Suppl_Figure_1_Sticta_tree_NEW-19.tif]
